# Supplementary material for: Patient-Related Risk Factors for Unplanned 30-Day Hospital Readmission Following Primary and Revision Total Knee Arthroplasty: A Systematic Review and Meta-Analysis
Source: J Clin Med. 2021 Jan 2;10(1):134. doi: 10.3390/jcm10010134 (PMC7795505; doi:10.3390/jcm10010134)

Contents

S1 – Amendments to Protocol 2
S2 - Search Strategies (MEDLINE(Ovid) and EMBASE(Ovid)) 4
S3 – Study cohort, geographical location, and type of study 7
S4 - Study inclusion and exclusion criteria, sample size, and patient characteristics 13
S5 - Outcome definition, missing data, and loss to follow-up 43
S6 - Prognostic factor selection 74
S7 - Adjustment for confounding 106
S8 –Summary of Findings: Revision-only studies 132
S9 - Results for all-cause readmission, adjusted analysis 138
S10 - Results for all-cause readmission, unadjusted and secondary analyses 167
S11 - Arroyo 2019 - Results from Exploratory Stratified Analysis 216
S12 – Results for other types of readmission, adjusted analysis 225
S13 – Results for other types of readmission, unadjusted and secondary analyses 233
S14 - Meta-analysis R code 235
S15 - Critical Appraisal 238
S16 - Summary of Findings – Comorbidities 244
S17 - Summary of Findings – Demographics 258
S18 - Summary of Findings – Other 262
S19 – Forest plots 265

S1 – Amendments to Protocol

Change to PICO framework:

In order to improve clarity, the PICO framework was changed to the following:

- P = Population = TKA recipients
- I = Predictor of Interest = Patient risk factors
- C = Comparator = TKA recipients who do not have the risk factor
- O = Outcome = 30-day readmission
- S = Study type = Case series were excluded. All other types of quantitative study design were eligible for inclusion, including retrospective and observational studies

Change to data extraction requirements:

It was originally decided that papers would be excluded from which number of patients and readmissions could not be obtained. Upon full-text screening, it became apparent that results from multivariate logistic regression analysis was the main way in which data were presented and that which was initially intended for capture was unavailable. It was subsequently decided that papers would not be excluded solely on the basis of being unable to obtain the number of patients and readmissions for each outcome. This is because the ultimate goal, as stated in the protocol, was to calculate effect estimates (risk ratios, but see below for separate amendment pertaining to risk ratios vs odds ratios)

Findings were also extracted from papers that did not conduct adjusted analyses, and this factored into the critical appraisal. It is still important to consider these findings, even though adjusted analyses are better.

Change from risk ratios to odds ratios:

It was originally planned to calculate risk ratios, but odds ratios were included because this was what was presented in most papers.

Regarding the inclusion of studies that only reported unadjusted effect estimates and/or univariate comparisons, it was decided to include these for the sake of completeness but rated at high risk of bias on critical appraisal. The protocol stated that the goal was to include/calculate risk ratios but did not specifically state that studies with unadjusted effect estimates and/or univariate comparisons would be excluded. 12 such studies were identified, ten of which presented only univariate comparisons between readmitted and non-readmitted patients.

Change from RevMan to R:

R was used instead of RevMan because it is flexible, and enables easy sharing and checking of code.

Change to modified GRADE approach and Summary of Findings tables:

The GRADE approach for systematic review of prognostic factors, developed by Huguet A et al 2013, was used [1]. The authors discovered this version of the GRADE framework after the protocol was written, therefore it was not specified in the protocol.

References:

1. Huguet A, Hayden JA, Stinson J, McGrath PJ, Chambers CT, Tougas ME, et al. Judging the quality of evidence in reviews of prognostic factor research: adapting the GRADE framework. Systematic reviews. 2013;2:71.

S2 - Search Strategies (MEDLINE(Ovid) and EMBASE(Ovid))

MEDLINE (Ovid) Search Strategy	
1	Exp *Arthroplasty, Replacement, Knee
2	(total knee replacement or total knee arthroplast* or TKR or TKA or TKJR or TKJA).ti
3	1 or 2
4	*risk/ or exp *protective factors/ or exp *risk assessment/ or exp *risk factors/
5	(risk factor* or risk assessment or body mass index or age or gender or comorbidit*).ab.
6	Exp Body Mass Index/
7	Exp *COMORBIDITY/
8	4 or 5 or 6 or 7
9	Exp *"length of stay"/ or exp *patient readmission/
10	(readmission or length of stay).ti. Or 30-day readmission.kw. or readmi*.ti
11	("30" adj4 day).mp. [mp=title, abstract, original title, name of substance word, subject heading word, floating sub-heading word, keyword heading word, protocol supplementary concept word, rare disease supplementary concept word, unique identifier, synonyms]

12	(thirty adj4 day).mp. [mp=title, abstract, original title, name of substance word, subject heading word, floating sub-heading word, keyword heading word, protocol supplementary concept word, rare disease supplementary concept word, unique identifier, synonyms]
13	9 or 10 or 11 or 12
14	3 and 8 and 13

**EMBASE (Ovid) Search Strategy**	
1	Exp *total knee arthroplasty/
2	(total knee replacement or total knee arthroplast* or TKR or TKA or TKJA or TKJA.ti.
3	1 or 2
4	Exp *risk factor/
5	(risk factor or risk assessment or body mass index or age or gender or comorbidit*.ab.
6	Exp *body mass/

7	Exp *comorbidity/ or exp *Charlson Comorbidity Index/ or exp *Elixhauser comorbidity index/ or exp *comorbidity assessment/
8	4 or 5 or 6 or 7
9	Exp *hospital readmission/ or exp *"length of stay"/
10	(readmission or length of stay).ti. or 30-day readmission.kw. or readmi*.ti.
11	("30" adj4 day).mp. [mp=title, abstract, heading word, drug trade name, original title, device manufacturer, drug manufacturer, device trade name, keyword, floating subheading word, candidate term word]
12	(thirty adj4 day).mp. [mp=title, abstract, heading word, drug trade name, original title, device manufacturer, drug manufacturer, device trade name, keyword, floating subheading word, candidate term word]
13	9 or 10 or 11 or 12
14	3 and 8 and 13
15	Exp time factor/
16	3 and 15
17	14 or 16
18	Limit 17 to embase

S3 – Study cohort, geographical location, and type of study

Study ID: Author and year of publication	Cohort (geographical location)	Type of study
**Abdulla 2020**	Alberta Bone and Joint Health Institute (ABJHI) registry (Alberta, Canada)	Retrospective cohort study
**Abola 2018**	*NSQIP (USA and various international sites)	Retrospective cohort study
**Ali 2019**	National Health Service (NHS) administrative database (UK)	Retrospective cohort study
**Alvi 2015**	NSQIP (USA and various international sites)	Retrospective cohort study
**Anderson 2020**	Veterans Affairs hospitals (USA)	Retrospective cohort study
**Anthony 2018**	National Readmissions Database (USA - 22 geographically dispersed states, accounting for 51.2% of the US resident population)	Retrospective cohort study
**Antoniak 2020**	NSQIP (USA and various international sites)	Retrospective cohort study
**Arroyo 2019**	State Inpatient Databases of the Healthcare Cost and Utilisation Project (HCUP)	Retrospective cohort study
**Belmont 2016**	NSQIP (USA and various international sites)	Retrospective cohort study
**Bovonratwet 2018**	NSQIP (USA and various international sites)	Retrospective cohort study
**Bovonratwet 2019**	NSQIP (USA and various international sites)	Retrospective cohort study

**Bovonratwet 2020**	NSQIP (USA and various international sites)	Retrospective cohort study
**Buitrago 2020**	Colombia’s contributory health care system (Colombia)	Retrospective cohort study
**Bullock 2003**	Single tertiary care facility (New Hampshire, USA)	Retrospective cohort study
**Charette 2019**	Single tertiary academic hospital (Pennsylvania, USA)	Retrospective cohort study
**Courtney 2018**	NSQIP (USA and various international sites)	Retrospective cohort study
**Curtis 2018**	NSQIP (USA and various international sites)	Retrospective cohort study
**Curtis 2019**	NSQIP (USA and various international sites)	Retrospective cohort study
**D'Apuzzo 2017**	Statewide Planning and Research Cooperative System (SPARCS) database from the New York Department of Health (New York, USA)	Retrospective cohort study
**George 2018**	NSQIP (USA and various international sites)	Retrospective cohort study
**Gwam 2020**	NSQIP (USA and various international sites)	Retrospective cohort study
**Hanly 2017**	Single tertiary referral centre (Brisbane, Australia)	Retrospective cohort study
**Hart 2016**	NSQIP (USA and various international sites)	Retrospective cohort study

**Jauregui 2015**	NSQIP - This was compared with a single prosthesis database (Sinai Hospital of Baltimore), but only the NSQIP data is pertinent to the current systematic review so there will be no further mention of the single prosthesis database (USA and various international sites)	Retrospective cohort study
**Jorgensen 2013**	Lundbeck Foundation Centre for Fast-Track Hip and Knee Replacement Collaboration (LCFC) database, cross-referenced with the Danish National Health Register (Denmark)	Retrospective cohort study
**Jorgensen 2017**	Lundbeck Foundation Centre for Fast-track Hip and Knee Replacement Collaboration (all patients having THA and TKA in the participating departments complete a questionnaire on comorbidity and other demographic information, which is subsequently merged with the Danish National Database on Reimbursed Prescriptions for additional information on dispensed drugs before and after surgery (Denmark)	Prospective cohort study
**Keeney 2015**	Single institution (Two groups were identified - Group A (before implementation of perioperative risk reduction efforts) = procedures from 1 January 2006 - 31 December 2009, Group B (after implementation of perioperative risk reduction efforts) = 1 January 2010 - 30 September 2013; Missouri, USA)	Retrospective cohort study
**Kester 2016**	NSQIP (USA and various international sites)	Retrospective cohort study
**Kheir 2014**	Urban hospital network (Philadelphia, USA)	Retrospective cohort study
**Kim 2019**	Medicare claims data (USA)	Retrospective cohort study
**Kuo 2017**	Single institution - Kaohsiung Chang Gung Memorial Hospital (Taiwan)	Case-control

**Kurtz 2016**	Medicare 100% national hospital claims database (USA)	Retrospective cohort study
**Lehtonen 2018**	NSQIP (USA and various international sites)	Retrospective cohort study
**Liao 2016**	National Health Insurance Research Database (Taiwan)	Nested case-control study
**Lovecchio 2014**	NSQIP (USA and various international sites)	Retrospective cohort study
**Miric 2014**	Kaiser Permanente National Total Joint Replacement Registry (USA)	Retrospective cohort study
**Mudumbai 2019**	Veterans Health Administration national database (USA)	Retrospective cohort study
**Nowak and Schemitsch 2019**	NSQIP (USA and various international sites)	Retrospective cohort study
**Ottesen 2018**	NSQIP (USA and various international sites)	Retrospective cohort study
**Patel 2020**	NSQIP (USA and various international sites)	Retrospective cohort study
**Patterson 2018**	NSQIP (USA and various international sites)	Nested case-control study
**Peskun 2012**	Single database containing data from one community hospital and one academic university-affiliated hospital (Toronto, Canada)	Retrospective cohort study
**Pugely 2013**	NSQIP (USA and various international sites)	Retrospective cohort study
**Ramos 2014**	Single hospital database – Hospital for Joint Diseases (New York, USA)	Retrospective cohort study

**Ricciardi 2017**	Single institution – Hospital for Special Surgery (New York, USA)	Retrospective cohort study
**Robinson 2017**	NSQIP (USA and various international sites)	Retrospective cohort study
**Ross 2020**	Canadian Joint Replacement Registry (Ontario, Canada)	Retrospective cohort study
**Roth 2019**	NSQIP (USA and various international sites)	Retrospective cohort study
**Rudasill 2019**	NSQIP (USA and various international sites)	Retrospective cohort study
**Runner 2017**	NSQIP (USA and various international sites)	Retrospective cohort study
**Saucedo 2014**	Single hospital database (USA)	Retrospective cohort study
**Schaeffer 2015**	Single academic centre (USA)	Retrospective cohort study
**Schairer 2014**	Single academic centre (San Francisco, USA)	Retrospective cohort study
**Singh 2013**	Pennsylvania Health Care Cost Containment Council (PHC4) database (169 hospitals in the state of Pennsylvania, USA)	Retrospective cohort study
**Siracuse 2017**	State Inpatient Database (SID) (USA – California, Florida, New York, Washington)	Retrospective cohort study
**Sloan 2020**	NSQIP (USA and various international sites)	Retrospective cohort study
**Sodhi and Anis et al 2019**	NSQIP (USA and various international sites)	Retrospective cohort study

**Sodhi and Mont et al 2019**	OrthoMiDaS Episode of Care (USA - The four Cleveland Clinic hospitals (Main Campus, Euclid Hospital, Lutheran Hospital, and Florida Campus))	Prospective cohort study
**Suleiman 2015**	NSQIP (USA and various international sites)	Retrospective cohort study
**Sutton 2016**	NSQIP (USA and various international sites)	Retrospective cohort study
**Tang 2019**	Administrative, clinical, and ancillary health care systems in Singapore (Singapore)	Retrospective nested case-control study
**Tay 2017**	Single large tertiary hospital (Singapore)	Retrospective cohort study
**Urish 2018**	National Readmission Database (USA)	Retrospective cohort study
**Webb 2017**	NSQIP (USA and various international sites)	Retrospective cohort study
**Weick 2018**	Truven Health MarketScan databases (USA)	Retrospective cohort study
**Welsh 2017**	Medicare Provider Analysis and Review (MedPAR) files linked with enrolment indicator files (USA)	Retrospective cohort study
**Workman 2019**	Single institution community non-academic centre (Pennsylvania, USA)	Retrospective cohort study
**Yohe 2018**	NSQIP (USA and various international sites)	Retrospective cohort study
**Zusmanovic 2018**	NSQIP (USA and various international sites)	Retrospective cohort study

***NSQIP = American College of Surgeons National Surgical Quality Improvement Program**

S4 - Study inclusion and exclusion criteria, sample size, and patient characteristics

Study ID	Sample size calculation:	Inclusion criteria and selection method:	Exclusion criteria:	Sample size:	Number of exclusions (with reasons, when available):	*Patient characteristics:<br>Age (A)<br>**Sex (S)<br>**BMI (B)<br>CCI (I) – if unavailable, report ASA class (C)
Abdulla 2020	Not reported	Primary TKA	Unicompartmental knee replacements; bilateral knee replacements	Number of patients = 16,485<br><br>Number of readmissions = Not reported	Not reported	Normal weight (defined according to WHO categorisation system for BMI):<br>A = 70.3<br>S = 30.7% male<br>Presurgical risk factors: 0 = 25.9%; 1 = 30.2%; ≥2 = 43.9%
						Overweight:<br>A = 69.2<br>S = 44.7% male<br>Presurgical risk factors: 0 = 28.6%; 1 = 30.1%; ≥2 = 41.3%
						Class I obese:<br>A = 67.6<br>S = 43.3% male<br>Presurgical risk factors: 0 = 28.0%; 1 = 28.5%; ≥2 = 43.5%
						Class II obese:<br>A = 65.7<br>S = 35.6% male<br>Presurgical risk factors: 0 = 25.8%; 1 = 29.2%; ≥2 = 45.0%
						Class III obese:<br>A = 62.7

						S = 29.0% male<br>Presurgical risk factors: 0 = 22.7%; 1 = 26.2%; ≥2 = 51.1%
Abola 2018	Not reported	Patients aged ≥18 who underwent TKA, identified using CPT code 27447	Patients with hypernatraemia (defined as serum sodium level >145 mEq/L)	Number of patients = 88,103<br><br>Number of readmissions = Not reported	833	A: <40 = 0.44%; 40-59 = 22.89%; 60-79 = 66.56%; 80+ = 10.11%<br>S = 37.62% male<br>B: <20 = 0.71%; 20-35 = 65.34%; >35 = 33.95%<br>C: 1 = 2.19%; 2 = 51.14%; 3 = 45.08%; 4/5 = 1.59%
Ali 2019	Not reported	Primary TKA patients, identified using Office of Population censuses and Surveys (OPCS) procedure codes (available in Appendix A of the paper)	Not reported	Number of patients = 566,323<br><br>Number of readmissions = 35,252 (6.2%) all-cause 19,095 (3.4%) surgical 2686 (0.5%) return-to-theatre (RTT)	Not reported, although the authors stated that all primary TKA patients were included	Only available for combined cohort (TKA + unicompartmental knee arthroplasty + patellofemoral arthroplasty)
Alvi 2015	Not reported	Primary TKA (CPT code 27447) for osteoarthritis (ICD-9 code 715)	Arthroplasty for indication other than osteoarthritis	Number of patients = Not reported for TKA patients – only reported for combined cohort (THA + TKA)<br><br>Number of readmissions = Not reported, and unable to calculate	Unclear for stated exclusion criteria.<br><br>After initial cohort was identified, 432 patients were excluded - 242 with BMI <18.5 and 190 with missing BMI data	Not reported for TKA patients – only reported for combined cohort (THA + TKA)
Anderson 2020	Not reported	Primary TKA (CPT code 27447)	Revisions or simultaneous bilateral procedures (CPT codes 27486, 27487, and 27488); BMI <12; surgery time <0.75 or >4 hours	Number of patients = 12,639<br><br>Number of readmissions = 609 (4.82%)	Not reported	A = 65.06 (8.49)<br>S = 93.20% male<br>B = 31.81 (5.24)<br>I (modified version created by authors) = 1.01 (1.56)

Anthony 2018	Not reported	Primary procedure code for TKA, which is the Healthcare Cost and Utilization Project (HCUP)'s Clinical Classification Software (CCS) code 152. Various ICD-9-CM codes were then used to identify patients readmitted for surgical site infection (SSI) specifically – these patients comprised the study population	Readmissions for causes other than SSI	Number of patients = 404,104 procedures<br><br>Number of readmissions = 5217	Not reported	A: <18 = 0.1%; [18, 30) = 0.2%; [30, 40) = 0.5%; [40, 50) = 4.4%; [50, 60) = 21.5%; [60, 70) = 37.5%; [70, 80) = 27.7%; 80+ = 8.1%<br>S = 38.7% male<br>B = 25% with positive "obesity flag"
Antoniak 2020	Not reported	Elective primary TKA >= 65yo, deduced from CPT code	End-stage kidney disease (ESKD), acute kidney injury (AKI), sepsis/septic shock/systemic inflammatory response syndrome (SIRS), urinary tract infection (UTI), pneumonia, ventilator dependence, BMI >100kg/m^2, missing creatinine	Number of patients = 104,580 available for analysis of 'Major Complication' (= composite outcome including readmission)<br><br>Number of readmissions = (calculated from %): 4375.98	312 non-elective<br>98 met multiple exclusion criteria<br><br>Number of patients excluded due to missing data in a particular predictor:<br>7850 (creatinine)<br>515 (ESKD)<br>38 (AKI)<br>220 (sepsis/septic shock/SIRS)<br>139 (UTI)<br>13 (pneumonia)<br>24 (ventilator dependence)<br>6 (BMI>100kg/m^2)<br>20,743 (missing data for ≥1 covariate)	Overall TKA cohort included in 'Major Complications' model:<br>A = 73 (6)<br>S = 37.1% male<br>B = 31.8 (6.3)<br>C: 1-2 = 46.4%; 3 = 51.5%; 4-5 = 2%<br><br>TKA patients excluded from 'Major Complications' model due to missing data:<br>A = 73.3 (6)<br>S = 38.7% male<br>B = 31.6 (6.2)<br>C: 1-2 = 53.2%; 3 = 45.4%; 4-5 = 1.4%

Arroyo 2019	Not reported	Primary TKA patients	Age <18y, missing demographic data (age, sex, primary insurance status, LOS, postoperative disposition, days to readmission), death during index hospitalisation, missing VisitLink data, or insufficient follow-up time after initial hospitalisation	Number of patients = 739,857:<br>UTKA = 709,929<br>BTKA = 32,928<br><br>Number of readmissions = 36,802<br>UTKA rate = 5%<br>BTKA rate = 4.5%	182,962	A = 67.31 ± 10.10<br>B (BMI unavailable, so proportion with obesity is reported): 21.2%<br>S = 37.0% male<br>I (CCI unavailable so Elixhauser Index (median (quartile 1 – quartile 3) is reported) = 0 (-1;0)
Belmont 2016	The authors stated that "Given the size of our sample (n = 1754), it was determined that this study was adequately powered to detect a 5% difference in readmission rates between cohorts." (page 5)	Revision TKA, identified using CPT code 27487	Not reported	Number of patients = 1754<br>Number of readmissions = 108	Not reported	A = 66.2 (10.9)<br>S = 41.1% male<br>B = 32.6 (7.5)<br>C: 1 = 1.2%; 2 = 42.8%; 3 = 52.8%; 4 = 3.2%; 5 = 0
Bovonratwet 2018	Not reported	Revision TKA, identified using CPT codes: 27486 (single-component revision) and 27487 (2-component revision)	Emergency surgery; ICD codes indicating fracture, trauma, neoplasm, infection, septic indications, or inflammatory	Number of patients = 9899, of whom 8769 had readmission data available due to readmission data being recorded in NSQIP database from 2011 onwards.	Not reported	Age <70 before propensity score matching (only available for total 2005-2015 cohort, not specifically for the 2011+ cohort for which readmission data were available):<br>A (average) = 59.0<br>S = 39.41%

			diagnoses were excluded	The following totals were thus calculated from the given number of readmissions in each category divided by the given readmission rate, then rounded down.<br><br>Age ≥80: 51/0.0623 = 818<br><br>Before propensity score matching:<br>Age <70: 299/0.052 = 5750<br>Age 70-79: 122/.0555 = 2198<br><br>After propensity score matching:<br>Age <70: 46/0.0545 = 844<br>Age 70-79: 837<br><br>Number of readmissions =<br>Age ≥80 = 51<br><br>Before propensity score matching:<br>Age <70 = 299<br>Age 70-79 = 122<br><br>After propensity score matching:<br>Age <70 = 46<br>Age 70-79 = 47		B: 18-25 = 8.95%; 25-30 = 22.18%; 30-35 = 28.32%; >35 = 40.55%<br>C: 1-2 = 48.17%; 3 = 49.69%; ≥4 = 2.15%<br><br>Age 70-79 before propensity score matching (only available for total 2005-2015 cohort, not specifically for the 2011+ cohort for which readmission data were available):<br>A (average) = 74.0<br>S = 39.78%<br>B: 18-25 = 10.44%; 25-30 = 30.25%; 30-35 = 29.73%; >35 = 29.57%<br>C: 1-2 = 37.27%; 3 = 58.95%; ≥4 = 3.79%<br><br>Age ≥80 before propensity score matching (only available for total 2005-2015 cohort, not specifically for the 2011+ cohort for which readmission data were available):<br>A (average) = 83.5<br>S = 40.33%<br>B: 18-25 = 19.85%; 25-30 = 38.35%; 30-35 = 25.18%; >35 = 16.61%<br>C: 1-2 = 27.69%; 3 = 67.50%; ≥4 = 4.81%
Bovonratwet 2019	Not reported	Primary TKA patients, identified using CPT code 27447, who underwent rapid recovery procedures (defined as length of stay ≤1 day)	Patients who underwent emergency surgery or had primary ICD diagnosis codes indicating fracture, trauma, neoplasm, infection, or	Number of patients = 18,196 split into two age groups: age <80 and age ≥80. Since readmission has only been recorded in NSQIP form 2011 onwards, 17,940 (98.6%) of these patients had readmission data available	Not reported for exclusion criteria. After applying eligibility criteria, 195 patients (1.06%) were excluded due to missing data for certain preoperative, procedural, or postoperative variables	Age <80 (only available for total 2005-2015 cohort, not specifically for the 2011+ cohort for which readmission data were available):<br>A = 64.0 (8.4)<br>S = 49.26% male<br>B: 18-25 = 9.68%; 25-30 = 29.72%; 30-35 = 30.47%; >35 = 30.13%

			inflammatory diagnoses.<br><br>Patients who underwent revision surgery	The following totals were thus calculated from the given number of readmissions in each category divided by the given readmission rate, then rounded down.<br><br>Age <80: 411/0.0243 = 16,913<br><br>Age ≥80: 44/0.0183 = 2404<br><br>Number of readmissions =<br>Age <80 = 411 (2.43%)<br>Age ≥80 = 44 (4.43%)		C: 1-2 = 59.44%; 3 = 39.72%; ≥4 = 0.84%
						Age ≥80 (only available for total 2005-2015 cohort, not specifically for the 2011+ cohort for which readmission data were available):<br>A = 82.8 (2.7)<br>S = 46.87% male<br>B: 18-25 = 25.57%; 25-30 = 41.39%; 30-35 = 24.08%; >35 = 8.96%<br>C: 1-2 = 46.77%; 3 = 51.44%; ≥4 = 1.79%
Bovonratwet 2020	Not reported	Primary TKA (CPT code 27447), unilateral, with length of stay (LOS) of 0, 1, and 2 days	Cases involving trauma, fracture, infectious diseases, neoplasms, or bilateralism; patients who had fatal complications on the day of surgery; cases with missing height, functional status prior to surgery, ASA classification, anaesthesia type, or readmission	Number of patients = 117,774:<br>Patients who had LOS 0 days = 3015<br>Patients who had LOS 1 day = 31,163<br>Patients who had LOS 2 days = 83,596<br><br>Number of readmissions = 78 for outpatient (LOS = 0) TKA patients. Not reported for other groups	Cases involving trauma, infection, neoplasm, bilateralism, revision, or fatal complications on day of surgery = 1874<br><br>Cases with missing height, functional status prior to surgery, ASA classification, anaesthesia type, or readmission = 1465	LOS = 0 (outpatient):<br>A = 65.2 (9.6)<br>S = 46.0% male<br>B = 32.1 (6.3)<br>C: 1-2 = 60.4%; ≥3 = 39.6%
						LOS = 1:<br>A = 65.4 (9.0)<br>S = 47.8% male<br>B = 32.2 (6.1)<br>C: 1-2 = 57.2%; ≥3 = 42.8%
						LOS = 2:<br>A = 65.5 (9.1)<br>S = 41.4% male<br>B = 32.9 (6.6)<br>C: 1-2 = 54.4%; ≥3 = 45.6%

Buitrago 2020	Not reported	Primary TKA	Not reported	Number of patients = 12,453<br><br>Number of readmissions = 533 (4.28%)	Not reported	A (median, p25, p75) = 68.1 (61.96; 74.83)<br>S = 28.37% male<br>I: 0 = 56.29%; 1-2 = 33.7%; ≥3 = 9.93
Bullock 2003	To achieve 80% power, with a two-sided type-I error of 0.05, estimated one-year mortality rates of 0.3% for the unilaterally treated patients and 3.3% for the bilaterally treated patients, and a study population with twice as many unilaterally treated patients as bilaterally treated patients, 500 patients treated with unilateral arthroplasty and 250 treated with bilateral arthroplasty were required.	Simultaneous bilateral TKA or unilateral TKA	Additional procedure performed under the same anaesthesia; additional complicating diagnosis; blood transfusions were refused; the procedure was a revision arthroplasty; component augmentation was require; a tourniquet was not used; arthroplasty was a two-stage procedure for septic arthritis	Number of patients =<br>Unilateral = 512<br><br>Bilateral = 255<br><br>Number of readmissions =<br>Unilateral = 12 (2.3%)<br><br>Bilateral = 9 (3.6%)	Unilateral:<br>44 (7.9%) due to exclusion criteria):<br>Second procedure = 17<br>Additional diagnosis = 8<br>Haemophilia = 2<br>Beta thalassaemia = 1<br>Essential thrombocytosis = 1<br>Osteogenesis imperfecta = 1<br>Synovial lymphoma = 1<br>Paget's disease = 1<br>Multiple Myeloma = 1<br>Refused transfusions = 6<br>Revision = 5<br>Component augmentation = 4<br>No tourniquet = 2<br>Multi stage for septic knee = 2<br><br>Two more patients (0.4%) were excluded from the 30-day (mortality) analysis due to loss to follow-up<br><br>Bilateral:<br>11:<br>Second procedure = 3<br>Additional diagnosis = 3<br>Haemophilia = 1<br>Paget's disease = 1<br>Chondrodysplasia = 1<br>Refused transfusions = 1<br>No tourniquet = 4	Unavailable
Charette 2019	Not reported	Primary TKA patients	TKA for aetiology other than osteoarthritis	Number of patients = 4259	92	Overall:<br>A (range) = 64 (18-94)<br>S = 32.2% male

				Number of readmissions = 298 (7.0%)		B: <30 = 26.7%; >30 = 73.3%<br>C: 1 = 44%; 2 = 53.1%; 3 = 45.0%; 4 = 0.9%
						Age <55:<br>A = 17.4% of the overall cohort<br>S = 32.9% male<br>B: <30 = 15.4%; >30 = 84.6%<br>I = 2.2
						Age ≥55:<br>A = 82.6% of the overall cohort<br>S = 32.1%<br>B: <30 = 29.1%; >30 = 70.9%<br>I = 4.6
Courtney 2018	Based on a prior published complication rate among patients undergoing revision TKA at 8%, with 20% of patients undergoing TKA revised for infection, to detect a 2% difference in complication rate, with a type I error rate of 0.05, required sample size was calculated	Revision TKA patients identified using CPT codes: 27486 (single-component revision), 27487 (both-component revision), 27488 (explantation and placement of a spacer)<br><br>Patients having a diagnosis of infection were identified based on principal ICD-9 diagnosis codes 996.6x and 711.xx. Patients undergoing	Primary diagnosis of malignancy (ICD-9 codes 170.7, 170.9, 171.8, or 198.5)	Number of patients = 10,844:<br>1999 for infection<br>8845 for aseptic revision<br><br>Number of readmissions = 692 total<br>Infection group = 199 (10%)<br>Aseptic group = 493 (6%)	Four patients were excluded due to primary diagnosis of malignancy	Aseptic revision:<br>A = 65.0 (11)<br>S = 39% male<br>B = 33.4 (8)<br>C: 1 = 1%; 2 = 40%; 3 = 55%; 4 = 3%; 5 = <0.1%
						Revision for infection:<br>A = 66.2 (11)<br>S = 54% male<br>B = 32.8 (8)<br>C: 1 = 0.4%; 2 = 26%; 3 = 66%; 4 = 8%; 5 = 0

	to be 10,584 to achieve power of 0.80<br><br>A statistical power calculation was not carried out specifically for 30-day readmission	revision for any other diagnosis code were considered as undergoing aseptic revisions				TKA explant with spacer:<br>A = 65.7 (11)<br>S = 52% male<br>B = 34.0 (10)<br>C: 1 = 1%; 2 = 27%; 3 = 65%; 4 = 7%; 5 = 0.1%
Curtis 2018	Not reported	TKA patients with osteoarthritis, identified using CPT code 27447 (OA identified using ICD-9 code 715.xx)	Patients with concurrent surgeries during admission; cases listed as emergency surgeries	Number of patients = 111,624, of which 3466 (3.1%) were chronic immunosuppressant users<br><br>Number of readmissions = Calculated from given % readmission rates and rounded down:<br>Non-immunosuppressant group (3.5%) = 3785<br>Immunosuppressant group (5.2%) = 180	Not reported	Control group:<br>A (range) = 67 (20-89)<br>S = 38% male<br>B = 33 (7)<br>C = 47% ≥3<br><br>Immunosuppressant group:<br>A = 66 (18-89)<br>S = 30% male<br>B = 32 (7)<br>C = 66% ≥3
Curtis 2019	N/A	Primary TKA identified using CPT code 27447	Patients with fractures; non-elective surgery; bilateral TKA; cases with unknown preoperative functional status	Number of patients = 188,172 total:<br>IND (i.e. able to perform ADLs without assistance) = 186,066<br>DEP (i.e. require assistance to perform ADLs) = 2166<br><br>Number of readmissions = Not reported	Patients with fractures = 177<br>Non-elective surgery = 2234<br>Bilateral TKA = 5483<br>Cases with unknown preoperative functional status = 1126	Independent function:<br>A = 67 (10)<br>S = 38% male<br>B = 33 (3)<br>C = 38% ≥3<br><br>Dependent function:<br>A = 69 (11)<br>S = 29% male<br>B = 34 (8)<br>C = 69% ≥3

D'Apuzzo 2017	Not reported	Primary elective TKA, identified using ICD-9-CM code 81.51 for diagnosis of osteoarthritis, inflammatory arthritis, or osteonecrosis	Non-elective cases (indication any of: neoplasm, fracture, congenital condition, infectious condition, or other); admission included concurrent THA, revision THA or TKA, or hip resurfacing procedure; >2 TKA procedures coded during the index hospitalisation; hospital discharge against medical advice; lack of complete information	Number of patients = 377,705<br><br>Number of readmissions = 22,076 (5.8%)	Not reported	All-cause readmission group:<br>A = 69.2 (10.5)<br>S = 36.9% male<br>B (% obesity) = 15.3%
						Non-readmitted group:<br>A = 66.8 (10.3)<br>S = 33.8% male<br>B (% obesity) = 16.7%
George 2018	Not reported	Primary TKA, identified using CPT code 27447	Missing BMI data; underweight BMI range (<18.5)	Number of patients = 150,934<br><br>Number of readmissions = 5189	750 (0.5% of initial sample of 151,684 TKAs):<br>403 missing BMI data; 347 underweight BMI range	Normal weight (BMI >18.5 to <25):<br>A = 70.6 (10.7)<br>S = 31.28% male<br>C: 1 = 4.35%; 2 = 60%; 3 = 34.33%; 4+ = 1.25%
						Overweight (BMI >25 to <30):<br>A = 69.0 (9.7)<br>S = 45.11% male<br>C: 1 = 3.5%; 2 = 60.46%; 3 = 34.71%; 4+ = 1.21%
						Obese (BMI >30 to <40):<br>A = 65.9 (9.1)<br>S = 38.83% male<br>C: 1 = 1.45%; 2 = 50.59%; 3 = 46.52%; 4+ = 1.38%
						Morbidly obese (BMI >40):<br>A = 61.9 (8.4)

						S = 26.72% male<br>C: 1 = 0.36%; 2 = 24.3%; 3 = 72.07%; 4+ = 3.19%
Gwam 2020	Not reported	Primary TKA (CPT code 27447)	Non-elective TKA	Number of patients = 224,376 total, from which 349 dialysis patients were propensity score matched to 349 non-dialysed patients<br><br>Number of readmissions = Not reported	17,287 (due to non-elective TKA status)	Total cohort from which dialysis patients were identified:<br>A = 66.6<br>S = 38% male
Hanly 2017	Not reported	Primary elective TKA	Not reported	Number of patients = 117 patients with morbid obesity were selected and then (BMI ≥40) compared with 94 controls (BMI 18.5 to <25)<br><br>Number of readmissions = 25	835 patients were excluded from the initial sample (1931) due to missing BMI data to arrive at 1096 patients from which the final study cohort was selected	Morbidly obese:<br>A = 62.8 (7.5)<br>B = 44.2 (3.6)<br>S = 26.5% male<br><br>Normal weight:<br>A = 73.9 (9.3)<br>B = 23.2 (1.7)<br>S = 37% male
Hart 2016	Not reported	Primary unilateral and bilateral simultaneous TKA, identified using CPT code 27447	Patients with a primary diagnosis code of infection, fracture, or malignancy were excluded<br><br>Other exclusions = patients with any of: incomplete demographic information, emergency cases, errant concomitant CPT codes, cases with a wound classification other than 'clean'	Number of patients = 8561: 1771 simultaneous bilateral TKA patients matched 1:4 with 6970 unilateral TKA patients<br><br>Number of readmissions = 64 in the bilateral TKA group 240 in the unilateral TKA group	Not reported	Unilateral:<br>A = 65.3 (8.3)<br>S = 43.7% male<br>B = 33.4 (7.1)<br>C: 1 = 3.4%; 2 = 57.8%; 3 = 37.8%; 4 = 1%<br><br>Bilateral:<br>A = 64 (8.6)<br>S = 42.1% male<br>B = 32.8 (6.7)<br>C: 1 = 3.4%; 2 = 57.4%; 3 = 38.1%; 4 = 1.1%

Jauregui 2015	Not reported	Primary TKA patients, identified using CPT code 27447	The authors only evaluated patients according to whether they were readmitted at any later time point for any reason in 2011<br><br>Planned readmissions were then excluded	Number of patients = 12,010<br><br>Number of readmissions = 523 (4.3%)	87.92% of patients in 2011 readmitted at any time (12,035/13,689)<br><br>Planned readmissions were then excluded (n=25)	NSQIP readmitted:<br>A (mean (range)) = 69 (30 to >90)<br>S = 43.4% male<br>B (mean (range)) = 33 (17-61)<br><br>NSQIP non-readmitted:<br>A (mean (range)) = 67 (18 to >90)<br>S = 37.2% male<br>B (mean (range)) = 33 (14-85)
Jorgensen 2013	The authors acknowledged that an RCT would be the best study design, but would require >9000 patients to exclude a 20% difference in readmissions between non-smoking/alcohol and smoking/alcohol patients in fast-track patients, with 80% power	All TKA patients age >18y in the hospitals participating in the LCFC	Patients with missing data regarding alcohol use or smoking	Number of patients = 1481<br><br>Number of readmissions = 94	71 (2.3%) from the combined overall THA + TKA cohort	N/A

Jorgensen 2017	Sensitivity analysis found that the study was powered to detect a minimal odds ratio of 1.9 for 30-day readmission using α of 0.05, β of 0.80, and R^2 of other variables of 0.9.	Primary TKA at one of five centres using methylprednisolone (MP)	Exclusions from control group (before introduction of MP): pre- or postoperative dispensed prescriptions for systemic glucocorticoids; reported type 1 diabetes or pre- or postoperative dispensed prescriptions for insulin<br><br>Exclusions from experimental group (after introduction of MP) = did not receive MP. Reasons: allergy;	Number of patients = 3927:<br>With MP = 1442<br>Without MP = 2485<br><br>Number of readmissions = 191:<br>With MP = 81 (5.6%)<br>Without MP = 110 (4.4%)	Initial sample = 4363 (2655 before introduction of MP, 1708 after).<br><br>Exclusions before introduction of MP = 170:<br>Pre- or postoperative dispensed prescriptions for systemic glucocorticoids = 94<br>Reported type 1 diabetes or pre- or postoperative dispensed prescriptions for insulin = 76<br><br>Exclusions after introduction of MP = 266:<br>Allergy = 6<br>Preoperative glucocorticoid treatment = 33<br>IDDM = 47<br>Active gastric ulcer = 27<br>Unspecified = 45<br>NIDDM = 36	MP patients:<br>A = 69 (62-75)<br>S = 60% male<br>B (median (IQR)) = 28.6 (25.5-32.0)

			preoperative glucocorticoid treatment; IDDM; active gastric ulcer; unspecified; NIDDM; cardiac comorbidity; cancer; previous gastric ulcer; included in other studies; converted from UKA; forgotten; surgeon preference; other		Cardiac comorbidity = 5<br>Cancer = 14<br>Previous gastric ulcer = 6<br>Included in other studies = 7<br>Converted from UKA = 2<br>Forgotten = 9<br>Surgeon preference = 19<br>Other = 4	Control:<br>A = 68 (62-75)<br>S = 62%<br>B (median (IQR)) = 28.2 (25.3-32.4)
Keeney 2015	Post-hoc power analysis was done to determine that a sample size twice as large as the present study would be required to detect a statistically significant difference for the magnitude of change in 30-day readmission rates for socially	Primary TKA	Medical payer status other than a commercial carrier, Medicare, or Medicaid; unidentifiable socioeconomic status	Number of patients = 3118:<br>2006-2009 group = 1337<br>2010-2013 group = 1772<br><br>Number of readmissions = 138 (4.1%) total	2006-2009 group = 106 (3.1%)<br>2010-2013 group = 157 (3.7%)	A = 63.4 (10.5)<br>S = 31.9% male<br>B = 33.0 (7.4)<br>I = 0.9 (1.2)

	disadvantaged minority patients					
Kester 2016	Not reported	Primary TKA, identified using CPT code 27447 (arthroplasty, knee, condyle and plateau; medial and lateral compartments with or without patellar resurfacing); post-traumatic patients identified with ICD-9 groups and concurrent CPT coding – Appendix A provides full details (available in full text of the article)	Cases with prior operation in the last 30 days; cases with missing (or "null") preoperative variables; unicompartmental arthroplasty; revision arthroplasty, unspecified site of concurrent operations; pathologic fractures; inflammatory arthropathies, and osteogenesis imperfecta. Full details in Appendix B (available in full text of the article)	Number of patients = 68,349, comprising:<br>67,675 non-traumatic<br>674 post-traumatic<br><br>Number of readmissions = Calculated from % readmission rates and rounded down:<br>Non-traumatic = 535<br>Post-traumatic = 10	Not reported	Non-traumatic:<br>A = 66.9<br>S = 37.5% male<br>B = 32.8<br>C = 46.1% ≥3<br><br>Post-traumatic:<br>A = 59.3<br>S = 48.7%<br>B = 31.5<br>C = 38.1% ≥3
Kheir 2014	Sample of convenience	Primary TKA, identified using ICD-9 procedure code 81.54	Planned readmissions (most commonly for in-house acute inpatient rehabilitation or skilled nursing facility); revision procedures	Number of patients = 3218<br><br>Number of readmissions = 165 patients – 178 readmissions	Not reported	A = 63.0 (10.9)<br>B = 32.8 (7.6)

Kim 2019	Not reported	TKA patients age ≥65 years with diagnosis of OA or rheumatoid arthritis at time of index TKA	Patients with no claims during the 360-day baseline period (i.e. Medicare eligible but may have been receiving care through alternate health insurance coverage)	Number of patients = 316,593<br><br>Number of readmissions = 16,786	730,065, comprising:<br>506,687 without continuous enrolment in Medicare Parts A, B, and D at baseline<br>66,094 younger than 65 years<br>26 without any claims at baseline<br>155,586 underwent hip replacement on or before the index date<br>1672 without OA or rheumatoid arthritis diagnosis at baseline	Continuous opioid users:<br>A = 72.7 (5.7)<br>S = 23.9% male<br>Combined comorbidity score = 1.9 (2.6)<br><br>Intermittent opioid users:<br>A = 73.7 (5.7)<br>S = 30.3% male<br>Combined comorbidity score = 1.3 (2.3)<br><br>Opioid-naïve patients:<br>A = 74.3 (5.8)<br>S = 35.9% male<br>Combined comorbidity score = 0.8 (1.8)
Kuo 2017	For 80% power to detect a difference at an overall significance level of 0.05, the authors calculated a required sample size of 84 per group (CKD and non-CKD). This was based on prior literature	Primary elective TKA – only minimally invasive surgery (MIS)	Revision surgery; unicompartmental TKA; conventional TKA procedures	Number of patients = 205 CKD patients matched 1:2 with 410 non-CKD patients<br><br>Number of readmissions = CKD group = 12<br><br>Non-CKD group (calculated from % readmission rate and rounded down) = 3	Unclear	CKD group:<br>A = 72.1 (8.2)<br>S = 14% male<br>B = 28.0 (4.2)<br><br>Non-CKD group:<br>A = 71.0 (5.8)<br>S = 18% male<br>B = 28.0 (5.1)

Kurtz 2016	Not reported	Primary TKA, identified with ICD-9-CM code 81.54	Patients younger than 65 years; patients enrolled in a Health Maintenance Organisation; patients residing outside the 50 US states	Number of patients = 952,593<br><br>Number of readmissions = 47,286	Unclear	A: 65-69 = 32.2%; 70-74 = 29.0%; 75-79 = 21.9%; 80-84 = 12.4%; 85+ 4%<br>S = 36.6% male<br>I: 0 = 54.5%; 1-2 = 37.5%; 2-4 = 6.7%; 5+ = 1.3%
Lehtonen 2018	Not reported	Elective primary TKA, identified using CPT code 27447	N/A	Number of patients = 137,209<br><br>Number of readmissions = 4668	Not reported	Not readmitted:<br>A = 66.53<br>S = 37.6% male<br>B: <18.5 = 0.2%; 18.5-25 = 9.8%; 25-30 = 27.0%; 30-35 = 28.6%; 35-40 = 19.2%; ≥40 = 15.3%<br>C: 1 = 2.1%; 2 = 50.6%; 3 = 45.6%; 4 = 1.5%; 5 = 0.00% (4 participants); not assigned = 0.08%
						Readmitted:<br>A = 68.49<br>S = 43.7%<br>B: <18.5 = 0.2%; 18.5-25 = 10.0%; 25-30 = 26.0%; 30-35 = 26.8%; 35-40 = 17.7%; ≥40 = 19.2%<br>C: 1 = 1.4%; 2 = 36.1%; 3 = 55.8%; 4 = 3.3%; 5 = 0.00% (0 participants); not assigned = 0.11%
Liao 2016	Not reported	Patients who underwent TKA surgery, identified as cases in which TKA was listed as the major procedure in the database	Patients <40 years old; patients diagnosed with cancer before TKA surgery	Number of patients = 3431 (including revision TKA patients):<br>358 with COPD<br>3073 without COPD<br><br>Number of readmissions = Calculated from % readmission rates and rounded down:<br>COPD group = 25 (7.0%)<br>Non-COPD = 122 (4.0%)	Unclear	A = 70.7 (8.12)<br>S = 25.4% male

Lovecchio 2014	Not reported	NSQIP database queried for all primary TKA cases with CPT code 27447	Revision TKA; aetiology other than osteoarthritis	Number of patients = 28,061 total:<br>No diabetes = 22,991<br>NIDDM = 3860<br>IDDM = 1165<br><br>Number of readmissions = 1200 total:<br>No diabetes = 939 (4.1%)<br>NIDDM = 177 (4.6%) IDDM = 83 (7.2%)	Not reported	No diabetes:<br>A = 66 (59, 74)<br>S = 38.6% male<br>B = 30.9 (6.2)<br>C: 1-2 = 58.7%, 3 = 40.0%, 4-5 = 1.3%<br><br>NIDDM:<br>A = 68 (62, 75)<br>S = 41.9% male<br>B = 34.0 (6.2)<br>C: 1-2 = 31.0%, 3 = 66.4%, 4-5 = 2.6%<br><br>IDDM:<br>A = 67 (61, 74)<br>S = 43.5% male<br>B = 35.1 (6.3)<br>C: 1-2 = 16.6%, 3 = 77.5%, 4-5 = 5.9%
Miric 2014	Not reported	Primary TKA for any indication	Not reported	Number of patients = Readmissions data were only available for a subset of patients from 2009-2011. Presented below are the number of patients in only that subsample:<br>90+ years old group= 74<br>80-89 years old group = 2868<br><80 years old group = 26,010<br><br>Number of readmissions = Total = 1047:<br>Age 90+ = 7<br>Age 80-89 = 203<br>Age <80 = 836	Not reported	A: <80 = 89.7%; 80-89 = 10.0%; 90+ = 0.3%<br>S = 37.5% male<br>B: <30 = 43.2%; ≥30 to <35 = 29.1%; ≥35 = 26.1%; unknown = 1.6%<br>C: 1&2 = 58.5%; ≥3 = 38.8%; unknown = 2.7%
Mudumbai 2019	Not reported	Primary and revision TKA using ICD-9 codes	Non-veterans; patients who had not used outpatient Veterans' Affairs (VA) pharmaceuticals	Number of patients = 5514 (4955 primary TKA, 599 revision TKA)<br><br>Number of readmissions = 531 (9.6%) overall – 9.5% primary, 11.1% revision	From the initial total Veterans Health Administration (VHA) sample for 2011 financial year (n=87,429), the following exclusions were made:<br>Non-veterans = 544	A: ≤54 = 14.96%; 55-65 = 52.14%; ≥66 = 32.90%<br>S = 94.25% male<br>I: 0 = 61.48%; 1 = 25.12%; 2+ = 13.40%

			within 180 days prior to admission; patients with metastatic cancer; patients with missing values		No outpatient VA pharmaceuticals within 180 prior to admission = 945<br>Metastatic cancer = 6522<br>Missing values = 5053<br>Did not undergo TKA = 68,851	
Nowak and Schemitsch 2019	Not reported	NSQIP database queried for all primary TKA cases with CPT code 27447.	LOS <0 or >4, to eliminate coding errors and potential outliers; patients whose time to complications was less than (or equal to) their LOS to eliminate patients with a prolonged LOS due to complication	Number of patients = 76,246 after propensity score-matching (from 187,907 that met inclusion criteria)<br><br>Number of readmissions = 2,398	Not reported	Not reported for patients with readmission data available (i.e. the cohort from 2011-2016)
Ottesen 2018	Not reported	Elective primary TKA, identified using CPT code 27447	Patients with ICD-9 or ICD-10 code for infection, tumour, or trauma	Number of patients = 250 dialysis-dependent<br>163,560 non-dialysis-dependent<br><br>Number of readmissions = 22 dialysis-dependent<br>4340 non-dialysis-dependent	Not reported	Non-dialysis-dependent:<br>A (median (IQR)) = 67 (60-74)<br>S: 37.73% male<br>B (median (IQR)) = 32 (28-37)<br>C (median (IQR)) = 2 (2-3)<br>Dialysis-dependent:<br>A (median (IQR)) = 68 (61-75)<br>S = 52.80% male<br>B (median (IQR)) = 32 (27-37)<br>C (median (IQR)) = 2 (2-3)
Patel 2020	Not reported	Elective primary TKA (CPT code 27447)	Not reported	Number of patients = 418,885<br><br>Number of readmissions = Not reported	Not reported	Not available for TKA-only cohort

Patterson 2018	Not reported	Elective primary TKA, identified using CPT code 27447	N/A	Number of patients = 339 dialysis-dependent, 213,666 non-dialysis-dependent<br><br>Number of readmissions = 26 (8.8%) of dialysis dependent patients, 6183 (3.4%) of non-dialysis dependent patients	Not reported	Dialysis-dependent:<br>A: <60 = 22.4%; 60-69 = 33.0%; ≥70 = 44.5%<br>S = 50.9% male<br>B: <18.5 = 12.7%; 18.5-25 = 0.3%; 25-30 = 29.5%; 30-40 = 44.8%; >40 = 12.7%
						Non-dialysis-dependent:<br>A: <60 = 23.7%; 60-69 = 38.0%; ≥70 = 38.3%<br>S = 37.9% male<br>B: <18.5 = 9.6%; 18.5-25 = 0.2%; 25-30 = 27.0%; 30-40 = 47.5%; >40 = 15.6%
Peskun 2012	Not reported	Patients treated with single anaesthetic bilateral TKA for osteoarthritis, match to patients treated with staged procedures	Aetiology other than osteoarthritis	Number of patients = 156 patients treated with single anaesthetic bilateral TKA matched to 78 staged bilateral TKA patients<br><br>Number of readmissions = 7 (2 single-anaesthetic, 5 staged procedure)	Not reported	Single-anaesthetic:<br>A = 67.73 ± 8.101<br>S = 39.1% male
						Staged procedures:<br>A = 66.20 ± 9.74<br>S = 32.5% male
Pugely 2013	Not reported	Elective primary TKA, identified using CPT code 27447	Patients with emergency treatment (NSQIP-defined); evidence of prior infection (wound class = 1, 2, 3, 4); patients missing readmissions data	Number of patients = 11,814<br><br>Number of readmissions = 544	1875 (from 13,689 identified in the initial cohort)	A = 66.85 (10.04)<br>S = 37.52% male<br>B = 68.45% <35kg/m^2<br>C: 1-2 = 52.33%; 3 = 46.00%; 4 = 1.66%
Ramos 2014	Not reported	Elective primary TKA	Patients transferred to another medical facility; patients who left against medical advice; patients who died during their index admission	Number of patients = 1668<br><br>Number of readmissions = 53	Not reported	Discharge to inpatient acute rehabilitation facility (IRF):<br>A = 69.0 (10.3)<br>S = 27% male<br>Comorbidity (surrogate marker based on MS-DRG code) = 3.9%
						Discharge to skilled nursing facility (SNF):<br>A = 67.9 (9.2)<br>S = 22% male

						Comorbidity (surrogate marker based on MS-DRG code) = 3.4%
						Discharge to home with health services:<br>A = 60.7 (10.2)<br>S = 39% male<br>Comorbidity (surrogate marker based on MS-DRG code) = 3.1%
Ricciardi 2017	Not reported	Primary unilateral TKA patients with a diagnosis of osteoarthritis	Revision arthroplasty; TKA for diagnosis of hip dysplasia, avascular necrosis, inflammatory disease, inflammatory arthropathy, rheumatoid arthritis, posttraumatic arthritis, or fracture; planned readmissions for unrelated surgery	Number of patients = After matching = 46 non-readmitted patients matched to 23 readmitted patients (from an initial sample of 10,759 primary TKA patients)<br><br>Number of readmissions = 23	Not reported	Only ASA class is available for the TKA-only cohort.<br>Non-readmitted group:<br>C: 1-2 = 69.6%; 3-4 = 30.4%
						Readmitted group:<br>C: 1-2 = 82.9%; 3-4 = 17.4%
Robinson 2017	Not reported	TKA patients aged ≥18 years, identified by CPT code 27447	BMI <18.5, hospital stay >365 days, emergency cases, incomplete patient demographics data	Number of patients = 87177 overall:<br>32,848 males and 54,329 females<br><br>Number of readmissions = Calculated from % readmission rates and rounded down:<br>1313 males<br>1684 females	Not reported	Males (37.7% of the overall cohort):<br>A = 66.5 (9.67)<br>B = 32.2 (6.17)<br>C = 47.9% ASA class >2
						Females (62.3% of the overall cohort):<br>A = 66.8 (9.82)<br>B = 33.5 (7.42)<br>C = 46.0% ASA class >2

Ross 2020	Not reported	Primary and revision TKAs, any indication. If two procedures, only the first was analysed	Age ≤ 18	Number of patients = 210,145<br><br>Number of readmissions = 7338	Not reported	A = 67.59 ± 9.69<br>S = 38.8% male<br>B = N/A<br>I: 0 = 76.2%; 1 = 17.0%; 2 = 5.0%; 3+ = 1.8%
Roth 2019	Not reported	Revision TKA patients, selected using CPT codes	Infected joints (CPT code 996.66 – infection and inflammatory reaction due to internal joint prosthesis), missing BMI values, underweight (BMI ≤18.5kg/m^2)	Number of patients = 9773<br><br>Number of readmissions = 691	Infected joints = 2004 (9.3% of initial sample)<br><br>Missing BMI values = 240 (1.1% of initial sample)<br><br>Underweight = 210 (0.98% of initial sample)	Normal weight (18.5-25; 10.5% of the overall cohort):<br>A = 67 (13)<br>S = 31.7% male<br>C: 1 = 2.6%; 2 = 49.2%; 3 = 44.4%; 4+ = 3.7%<br><br>Overweight (25-30; 29.5% of the overall cohort):<br>A = 67 (11)<br>S = 46% male<br>C: 1 = 2.3%; 2 = 49.7%; 3 = 45.5%; 4+ = 2.4%<br><br>Obese (30-40; 46.7% of the overall cohort):<br>A = 65 (10)<br>S = 41.6% male<br>C: 1 = 0.9%; 2 = 25.2%; 3 = 55%; 4+ = 2.8%<br>The proportions in the ASA class categories do not sum to 100%<br><br>Morbidly obese (40+; 17.0% of the overall cohort):<br>A = 62 (9)<br>S = 29% male<br>C: 1 = 0.1%; 2 = 3.7%; 3 = 73.1%; 4+ = 6%<br>The proportions in the ASA class categories do not sum to 100%
Rudasill 2019	Not reported	Primary TKA	<18y, no INR recorded within 1 day before the TKA, those	Number of patients = 21,239:<br>12,149 (INR <= 1)<br>8095 (INR >1-1.25)<br>834 (INR >1.25-1.5)	78,855 missing preoperative INR<br>92 missing time of recorded INR	INR <= 1:<br>A = 66.9 ± 9.8<br>S = 35.4% male<br>B = 32.8 ± 7.2<br>I = 0.4 ± 0.9

			missing the day of INR measurement	161 (INR >1.5)<br><br>Number of readmissions = (readmission due to any cause):<br>403 (3.3%) (INR <= 1)<br>364 (4.5%) (INR >1-1.25)<br>67 (8.0%) (INR >1.25-1.5)<br>17 (10.6%) (INR >1.5)<br><br>TKA-related:<br>289 (2.4%) (INR <= 1)<br>255 (3.2%) (INR >1-1.25)<br>46 (5.5%) (INR >1.25-1.5)<br>12 (7.5%) (INR >1.5)	120,581 preoperative INR recorded > one day before surgery	INR >1-1.25:<br>A = 69.9 ± 9.8<br>S = 50.7% male<br>B = 32.7 ± 7.4<br>I = 0.5 ± 0.9<br>INR >1.25-1.5:<br>A = 72.7 ± 9.6<br>S = 57.1% male<br>B = 33.0 ± 7.8<br>I = 0.6 ± 1.0<br>INR >1.5:<br>A = 71.5 ± 9.2<br>S = 52.8% male<br>B = 34.2 ± 7.8<br>I = 0.8 ± 1.1
Runner 2017	Not reported	Primary TKA, age ≥60. Selection based on CPT codes (specific codes not reported)	Not reported	Number of patients = 90,260<br><br>Number of readmissions = Unable to calculate	Not reported	N/A
Saucedo 2014	Not reported	Primary TKA identified using ICD-9 code 8154	Planned readmissions	Number of patients = 3890<br><br>Number of readmissions = 182 (calculated from % readmission rate and rounded down)	Unclear – cannot clearly distinguish THA from TKA exclusions	N/A
Schaeffer 2015	Not reported	All TKA patients during the study period	Nil	Number of patients = 662<br><br>Number of readmissions = 12	N/A	N/A
Schairer 2014	Not reported	Primary TKA (ICD-9 code 81.54, CPT code 27447), revision TKA (ICD-9 codes 00.80-00.84 and 81.55; CPT codes 27486 and 27487), and	Not reported	Number of patients = 1032 primary TKA patients (912 unilateral, 120 bilateral)<br>262 revision non-infective TKA<br>113 revision for infective TKA	294 – insufficient follow-up	Primary:<br>A = 63.6 (13.1)<br>S = 36.7% male<br>Revision (non-infective):<br>A = 62.1 (12.9)<br>S = 43.9% male

		antibiotic spacer implantation/removal (ICD-9 codes 84.56 and 84.57; CPT code 27448)		Number of readmissions = 118 (8.9%)		Revision (infective):<br>A = 62.9 (11.8)<br>S = 46% male
Singh 2013	Not reported	ICD-9-CM code 81.54 for primary TKA	Patients who had undergone primary THA, or revision TKA or THA, during the same hospitalisation were excluded from the analysis.<br><br>Patients with a documented prior TKA, using ICD-9-CM code v43.65	Number of patients = 17,994 (11,669 women, 6325 men)<br><br>Number of readmissions = 1061 (635 women, 426 men)	From Figure 1:<br>Missing ID = 23<br>Age < 18 = 5<br>Died in the same day = 2<br>Duplicated index = 2<br>Missing gender = 1<br>Revision TKA = 25<br>Hip procedure = 7<br>Prior TKA = 1323<br>LOS ≥15 = 63<br>No teaching hospital status = 26	Women (64.8% of overall cohort):<br>A (median (IQR)) = 69 (60-76)<br><br>Men (35.2% of overall cohort):<br>A (median (IQR)) = 69 (60-75)
Siracuse 2017	Not reported	Primary TKA (ICD-9 code 815.4)	Patients with a missing numeric identifier variable, used to track repeated visits, were excluded	Number of patients = 433,638 in derivation cohort<br><br>Number of readmissions = 22,158 (calculated from % readmission rate and rounded down)	Not reported	A = 67.3 (10.0)<br>S = 36.0% male
Sloan 2020	Post-hoc power analysis demonstrated that the study was over 95% powered to detect the difference in observed readmission rates between normal weight and obesity class III patients	Primary TKA (CPT code 27447)	Patients without BMI or albumin data	Number of patients = 101,474 with readmission data available<br><br>Number of readmissions = 3508 (3.46%)	Unable to access supplementary materials	Overall cohort:<br>A = 66.4 (9.6)<br>S = 37.5% male

Sodhi and Anis et al 2019	Not reported	NSQIP database queried for all primary TKA cases with current procedural terminology (CPT) code 27447.	BMI <15 or >70kg/m^2; operative time <30min or >500min; missing data on surgery type (elective or non-elective); missing data on length of stay	Number of patients = 209,178 (206,655 elective; 2523 non-elective)<br><br>Number of readmissions = 4578 (4514 elective; 64 non-elective)	1838	Elective:<br>A = 67 ± 10<br>S = 38% male<br>B = 33 ± 7<br>C = 2 ± 1<br>Non-elective:<br>A = 66 ± 10<br>S = 34% male<br>B = 34 ± 7<br>C = 3 ± 1
Sodhi and Mont et al 2019	No power analysis was performed because all patients were included for whom data were available from the data source	Elective primary or revision TKA	TKA status non-elective or performed by a non-participating surgeon	Number of patients = 584<br><br>Number of readmissions = 12	From Figure 1 (Strobe diagram) – initial sample (patients who underwent TKA) comprised 1821 patients – exclusions:<br>Non-elective surgery = 50<br>Non-participating surgeon = 179<br>Not sampled by Press Ganey = 607<br>Sampled by Press Ganey but did not respond to survey = 401	No 30-day readmission:<br>A = 65 (range = 29-88)<br>S = 40.5% male<br>B = 32 (range = 17-75)<br>30-day readmission:<br>A = 66 (range 51-86)<br>S = 33.33% male<br>B = 32 (range 23-46)
Suleiman 2015	Not reported	Unilateral or simultaneous bilateral (SB) primary TKA, identified by CPT code 27447	Not stated	Number of patients = 973 SB TKA matched to 973 unilateral TKA<br><br>Number of readmissions = 43 in unilateral TKA group<br>9 in SB TKA group	1105 of the 44,393 TKAs in the NSQIP 2010-2012 dataset were SB TKAs, and 973 of these were matched, therefore 132 exclusions	Unilateral TKA:<br>A = 64.3 (10.1)<br>B: underweight = 0.4%; healthy weight = 9.4%; overweight = 28.5%; obese class I = 28.7%; obese class II = 18.5%; obese class III = 14.4%<br>C: 1 = 3.2%; 2 = 54.9%; 3 = 40.2%; 4 = 1.4%

						SB TKA:<br>A = 64.0 (8.6)<br>B: underweight = 0.3%; healthy weight = 8.6%; overweight = 29.4%; obese class I = 28.7%; obese class II = 18.9%; obese class III = 14.1%<br>C: 1 = 3.4%; 2 = 54.7%; 3 = 40.1%; 4 = 1.3%
Sutton 2016	N/A	Elective primary TKA identified using CPT code 27447	Emergency procedures, fractures, bilateral procedures, revisions, cases with operative time <30 or >300 mins, patients with incongruent data, patients with length of stay ≥5 days	Number of patients = 31,044:<br>7044 early discharge (length of stay 0-2 days)<br>24,000 standard length of stay (3-4 days)<br><br>Number of readmissions = 209 early discharge, 922 standard length of stay	After other exclusion criteria were applied, 33,932 patients remained in the sample. 2888 patients were then excluded due to length of stay ≥5 days.	Early discharge:<br>A = 64.4 (9.3)<br>S = 48.3% male<br>B = 32.2 (6.6)<br>C: 1 = 2.8%; 2 = 58.8%; 3 = 37.4%; 4 = 1.0%
						Standard length of stay:<br>A = 67.4 (9.8)<br>S = 34.2% male<br>B = 32.8 (7.0)<br>C: 1 = 2.2%; 2 = 51.1%; 3 = 45.2%; 4 = 1.5%
Tang 2019	Not reported	TKA patients	Not reported	Number of patients = 2621<br><br>Number of readmissions = 44 considered after 1:3 propensity score matching. The total number of readmissions in the overall cohort was not reported	One patient, due to BMI that deviated significantly from the normal level	Readmitted due to complication:<br>A = 66.3 (7.7)<br>S = 36.4% male<br>B: <25 = 31.8%; 25-29.9 = 34.1%; 30-34.9 = 20.5%; ≥35 = 13.6%<br>C (ASA Physical Status): 1 = 6/8%; 2 = 84.1%; 3 = 9.1%
						No complication (not readmitted):<br>A = 66.8 (8.9)<br>S = 37.7% male<br>B: <25 = 34.6%; 25-29.9 = 25.4%; 30-34.9 = 28.5%; ≥35 = 11.5%<br>C (ASA Physical Status): 1 = 10.8%; 2 = 83.8%; 3 = 5.4%

Tay 2017	N/A	Age ≥80, primary unilateral TKA, diagnosis of primary osteoarthritis, and minimum follow-up of two years	Revision arthroplasty, bilateral TKA, previous high tibial osteotomy, diagnosis other than primary osteoarthritis	Number of patients = 209 OG matched to 209 YG from an initial sample (OG and YG combined) of 7532<br><br>Number of readmissions = (calculated from % values in Table 3 and rounded down):<br>YG = 3<br>OG = 10	133	Younger controls (YG, 60-79y):<br>A = 66.1<br>S = 19.6% male<br>B = 26.6<br>I: 0 = 65.1%; 1 = 25.8%; 2 = 8.1%; ≥3 = 1.0%<br><br>Octogenarians (OG):<br>A = 82.1<br>S = 18.7% male<br>B = 26.4<br>I: 0 = 49.8%; 1 = 32.1%; 2 = 13.4%; ≥3 = 4.8%
Urish 2018	N/A	Elective TKA identified using ICD-9-CM procedure code 8154	Age <18y. TKA for trauma or neoplasm, identified using ICD-9 codes 8151, 8006, 8155, 0080, 0081, 0082, 0083, 0084, 27486, 27487, 274888 and diagnosis codes 73310, 73314, 73315, 73316, 808, 820, 821, 827, 828	Number of patients = 224,465<br><br>Number of readmissions = 7816	Not reported	Non-readmitted:<br>A = 67 (9)<br>S = 37% male<br><br>Readmitted:<br>A = 66 (10)<br>S = 43% male
Webb 2017	Not reported	TKA identified by CPT code 27447	Trauma, revision	Number of patients = 99,508 (this represents 87.2% of all patients included in the study because readmission was only collected in the NSQIP from 2011 onwards)<br><br>Number of readmissions = Unable to calculate accurately	Not reported	(demographic data for the 87.2% of patients with readmission data were not available, so overall demographic data is given)<br>Without diabetes mellitus:<br>A: 15-54 = 11.5%; 55-64 = 29.9%; 65-74 = 35.3%; ≥75 = 23.3%<br>S = 36.6% male<br>B: 18-25 = 11.6%; 25-30 = 29.7%; 30-35 = 28.4%; >35 = 30.3%<br>I: 0-2 = 23.5%; 3 = 35.8%; ≥4 = 40.8%

						Non-insulin-dependent diabetes mellitus:<br>A: 15-54 = 7.4%; 55-64 = 29.3%; 65-74 = 41.0%; ≥75 = 22.3%<br>S = 40.1% male<br>B: 18-25 = 4.9%; 25-30 = 19.7%; 30-35 = 29.0%; >35 = 46.4%<br>I: 0-2 = 17.8%; 3 = 38.6%; ≥4 = 43.6%
						Insulin-dependent diabetes mellitus:<br>A: 15-54 = 8.8%; 55-64 = 30.0%; 65-74 = 41.7%; ≥75 = 19.6%<br>S = 43.2% male<br>B: 18-25 = 3.3%; 25-30 = 14.7%; 30-35 = 26.7%; >35 = 55.3%<br>I: 0-2 = 19.0%; 3 = 38.2%; ≥4 = 42.8%
Weick 2018	Not reported	CPT code for primary TKA (27447) and enrolled in the database continuously for at least six months prior to the index procedure. Two populations were studied from this sample: one with 12 months continuous post-operative follow-up, and a subset of this population that had at least three years continuous post-operative follow-up	Not specified	Number of patients = 232,694<br><br>Number of readmissions = 12,123	Unable to calculate (Figure 1 does not enable the reader to distinguish between THA and TKA exclusions from the total combined cohort in the initial sample)	12-month follow-up population:<br>A = 65.2<br>S = 38.8%<br>I = 0.080
						3-year follow-up population:<br>A = 66.0<br>S = 61.0%<br>I = 0.088

Welsh 2017	Not reported	Direct quote: “Primary TKA (unilateral or bilateral) and were discharged from an acute care hospital between January 1, 2009 and September 30, 2011 were included. We included only Medicare beneficiaries aged ≥ 66 years at the index hospitalization and those enrolled in a fee-for-service plan. TKA procedures were identified using the International Classification of Diseases, Ninth revision, Clinical Modification (ICD-9-CM) procedure code of 81.54.24 Bilateral procedures were identified using the TKA 81.54 procedure code listed under two surgical procedure code columns for a single stay in the MedPAR files.”	Direct quote: “Patients who were enrolled in a Health Maintenance Organization (HMO) at any time during the study period, died during initial hospitalization, had missing data on patient or clinical characteristics, had other than elective or traumatic reasons for admission or were discharged to a setting other than the three post-acute settings (IRF, SNF, and HHA) or community”	Number of patients = 607,169<br><br>Number of readmissions = 32,226	Total exclusions = 356,269. Reasons taken from Figure 1:<br><66 years = 119,456<br>HMO = 215,473<br>Died in hospital = 638<br>Non-trauma or elective admission = 952<br>Other discharge setting = 18,418<br>Died within 30 days = 862	A: 66-70y = 31.92%; 71-80y = 50.67%; 81+y = 17.41%<br>S = 36.12% male<br>I: (number of conditions) 0 = 57.22%; 1 = 32.08%; 2+ = 10.70%
Workman 2019	Not reported	Primary TKA (CPT code 27447)	Readmissions within 30 days after TKA were excluded if the patient underwent	Number of patients = 7482<br><br>Number of readmissions = 210	Five patients were excluded due to subsequent contralateral TKA within 30 days, and three were excluded due to	Readmit group:<br>A = 68.5 (10.9)<br>S = 40.48% male

			subsequent contralateral elective TKA or THA		contralateral THA within 30 days	Non-readmit group:<br>A = 64.9 (9.8)<br>S = 35.13% male
Yohe 2018	Not reported	Patients older than 80 years who underwent TKA (identified by CPT code 27447)	Patients who underwent a TKA for conditions such as acute trauma, infection, or malignancy as identified by International Classification of Diseases (ICD) Ninth or 10th Revision codes	Number of patients = 12,026<br><br>Number of readmissions = 566	Not reported	A: 80-84 years (70.95%), 85+ years (29.05%)<br>S = 35.36% male
Zusmanovic 2018	Not reported	Primary TKA<br>(CPT codes: CPT 27446, primary TKA without patella resurfacing; CPT 27447, primary TKA with<br>patella resurfacing)	All cases involving a diagnosis coding of infection, fracture, or malignancy were excluded	Number of patients = 167,703<br><br>Number of readmissions = Unable to calculate:<br>Normal weight = not reported<br>Overweight = not reported<br>Class I obesity = 1434<br>Class II obesity = 929<br>Class III obesity = 990	Not reported	N/A (only available for combined (THA & TKA) cohort)
*Wherever possible, patient characteristics for the entire cohort were obtained. If this was not possible, patient characteristics for the reported subgroups were obtained;<br>**presented as mean (standard deviation) unless stated otherwise						

S5 - Outcome definition, missing data, and loss to follow-up

Study ID	Definition of outcome (and was it a primary or secondary):	Method of measurement:	Approach to missing data		Loss to follow-up:	
			**Method:**	**Number with, or excluded due to, missing data (per prognostic factor):**	**Strategy to deal with it:**	**Proportion or likelihood:**
Abdulla 2020	30-day readmission primary outcome (or part of primary outcome) = one of the primary outcomes<br><br>From date of index procedure or date of discharge = unclear<br><br>Planned or unplanned readmission or both = both<br><br>All-cause or cause-specific = readmission due to any cause	Data cross-linked between Discharge Abstract Database (DAD) and National Ambulatory Care Reporting System (NACRS)	Not reported	Not reported	Use of the ABJHI database ensured all readmissions within the province of Alberta, Canada, were included	Proportion = Not reported<br><br>Likelihood of substantial loss to follow-up = Moderate
Abola 2018	30-day readmission primary outcome (or part of primary outcome) = one of the primary outcomes<br><br>From date of index procedure or date of discharge = index procedure<br><br>Planned or unplanned readmission or both = unclear<br><br>All-cause or cause-specific = all-cause	As per NSQIP protocols, described and referenced in the Materials and Methods section of the paper	Multiple chained imputation based on a series of 10 multivariable logistic regression equations.	Not reported	Not reported	Proportion = Not reported<br><br>Likelihood of substantial loss to follow-up = Unlikely.

Ali 2019	30-day readmission primary outcome (or part of primary outcome) = primary outcome<br><br>From date of index procedure or date of discharge = discharge<br><br>Planned or unplanned readmission or both = unplanned<br><br>All-cause or cause-specific = all-cause, surgical, and return-to-theatre (RTT) readmissions analysed separately	As per Hospital Episode Statistics (HES) data collection practices for the NHS, referenced in the paper	Not reported	Not reported	Use of comprehensive database, and ICD-10 codes	Proportion = Not reported<br><br>Likelihood of substantial loss to follow-up = Unlikely.
Alvi 2015	30-day readmission primary outcome (or part of primary outcome) = one of the primary outcomes<br><br>From date of index procedure or date of discharge = unclear<br><br>Planned or unplanned readmission or both = unplanned<br><br>All-cause or cause-specific = only SSI-related readmissions	As per NSQIP protocols, described and referenced in the Methods section of the paper	190 cases were excluded from analysis due to missing BMI data<br><br>Otherwise no mention of general approach to missing data<br><br>No mention of the fact that readmission is only captured in the NSQIP from 2011, which is the last year of analysis in this study	191	Not reported	Proportion = Not reported<br><br>Likelihood of substantial loss to follow-up = Unlikely.
Anderson 2020	30-day readmission primary outcome (or part of primary outcome) = one of the primary outcomes	Veterans Affairs (VA) administrative database.<br>This was supplemented by	Not reported	Not reported	All readmissions to VA facilities are recorded, and the authors supplemented these data (which comprised	Proportion = Not reported<br><br>Likelihood of substantial loss to follow-up = Low likelihood

	From date of index procedure or date of discharge = discharge<br><br>Planned or unplanned readmission or both = unclear<br><br>All-cause or cause-specific = unclear	data from the fee-bases table including visits to civilian hospitals, which are recorded when reimbursement is requested from a non-VA facility. An outside inpatient stay was included if the purposes of the visit was listed as civilian hospital without the following words: outpatient, PT, nursing, physical, emergency (if emergency was listed, this was documented as the purpose of the visit)			the majority of readmissions) with data from civilian facilities	
Anthony 2018	30-day readmission primary outcome (or part of primary outcome) = one of the primary outcomes<br><br>From date of index procedure or date of discharge = discharge<br><br>Planned or unplanned readmission or both = unplanned<br><br>All-cause or cause-specific = only SSI-related readmissions	Unique patient identifiers within the NRD enabled authors to track readmission for each patient discharge, and ICD-9 codes were used to identify SSI cases	An “other” category for primary payer was created, and missing values for the variable were contained in this category<br><br>Otherwise, univariate median imputation was carried out	The missingness in covariate data across the entire cohort (THA and TKA combined – 760,238 patients) was sparse (n = 1792 missing) and univariate median imputation had minimal impact	Patients have a unique identifier, enabling linkage across visits in 22 US states comprising 49.3% of all US hospitalisations	Proportion = Not reported<br><br>Likelihood of substantial loss to follow-up = Unlikely.
Antoniak 2020	30-day readmission primary outcome (or part of primary	As per NSQIP documentation	Potential predictors with >40% missing data were excluded.	See ‘Participants’ table – ‘Number of exclusions’	Not reported	Proportion = Not reported

	outcome) = one of the primary outcomes<br><br>From date of index procedure or date of discharge = index procedure<br><br>Planned or unplanned readmission or both = unplanned<br><br>All-cause or cause-specific = unclear	referenced in the paper	Patients with missing values for any variables included in a model were excluded from that specific model.<br><br>No mention of readmission data only being included in NSQIP from 2011 onwards	column – for number of patients excluded from the entire study, i.e. all models, on account of missing data<br><br>The number of patients excluded specifically from the readmission model was not reported		Likelihood of substantial loss to follow-up = Unlikely
Arroyo 2019	30-day readmission primary outcome (or part of primary outcome) = primary<br><br>From date of index procedure or date of discharge = discharge<br><br>Planned or unplanned readmission or both = unclear<br><br>All-cause or cause-specific = unclear	Data are coded such that each inpatient hospital discharge corresponds to 1 individual record; readmission records are linked to the initial inpatient discharge using a unique identifier and provide post-discharge days to readmission (VisitLink).	Complete case analysis on variables listed in Participants table, and 'missing' category generated for other variables (i.e. the variables with missing data that did not comprise part of the patient exclusion criteria)	N/A	Readmissions are documented if they occur in the same state as the original surgery.	Proportion = Not reported<br><br>Likelihood of substantial loss to follow-up = Unlikely
Belmont 2016	30-day readmission primary outcome (or part of primary outcome) = primary outcome<br><br>From date of index procedure or date of discharge = index procedure	As per NSQIP protocols, described and referenced in the Materials and Methods section of the paper	Variables missing >20% of the cohort were excluded from the multivariate logistic regression model to avoid model distortion	Not reported	As per NSQIP data collection practices, patients are followed up for 30 days postoperatively regardless of discharge status	Proportion = Not reported<br><br>Likelihood of substantial loss to follow-up = Unlikely.

	Planned or unplanned readmission or both = unplanned<br><br>All-cause or cause-specific = all-cause					
Bovonratwet 2018	30-day readmission primary outcome (or part of primary outcome) = one of the primary outcomes, along with other complications<br><br>From date of index procedure or date of discharge = index procedure<br><br>Planned or unplanned readmission or both = unclear<br><br>All-cause or cause-specific = unclear	As per NSQIP protocols, described and referenced in the Materials and Methods section of the paper	Not reported	Not reported	As per NSQIP data collection practices, patients are followed up for 30 days postoperatively regardless of discharge status	Proportion = Not reported<br><br>Likelihood of substantial loss to follow-up = Unlikely.
Bovonratwet 2019	30-day readmission primary outcome (or part of primary outcome) = one of the primary outcomes, along with other complications<br><br>From date of index procedure or date of discharge = index procedure<br><br>Planned or unplanned readmission or both = unclear<br><br>All-cause or cause	As per NSQIP protocols, described and referenced in the Methods section of the paper	Patients were excluded due to missing data for certain preoperative, procedural, or postoperative variables.	195 in total. Per-prognostic factor exclusions not reported. The authors cited prior literature indicating that this low proportion (1.06%) of patients excluded due to missing data was not expected to bias results	As per NSQIP data collection practices, patients are followed up for 30 days postoperatively regardless of discharge status	Proportion = Not reported<br><br>Likelihood of substantial loss to follow-up = Unlikely.

Bovonratwet 2020	30-day readmission primary outcome (or part of primary outcome) = one of the primary outcomes<br><br>From date of index procedure or date of discharge = index procedure<br><br>Planned or unplanned readmission or both = both<br><br>All-cause or cause-specific = all-cause (but specific causes recorded separately)	NSQIP follows patients for the occurrence of hospital readmission	Following inclusion and exclusion criteria, ~1% of patients had missing data for certain perioperative characteristics, including height, functional status prior to surgery, anaesthesia type, ASA classification, readmission. These patients were excluded.<br>The only variables for which there was >1% missing data were preoperative haematocrit and INR (6% and 37% missing, respectively) – missing data category/indicator variable was created for these	119,239 minus 117,774 = 1465	NSQIP follows patients for the occurrence of hospital readmission	Proportion = Not reported<br><br>Likelihood of substantial loss to follow-up = Low likelihood
Buitrago 2020	30-day readmission primary outcome (or part of primary outcome) = one of the secondary outcomes<br><br>From date of index procedure or date of discharge = unclear<br><br>Planned or unplanned readmission or both = unclear<br><br>All-cause or cause-specific = all-cause	Data collection from Colombia's Integrated Social Protection Information System (SISPRO), which contains information from all health care providers for all those enrolled in the system	Not reported	Not reported	Use of a comprehensive national database	Proportion = Not reported<br><br>Likelihood of substantial loss to follow-up = Low likelihood

Bullock 2003	30-day readmission primary outcome (or part of primary outcome) = one of the primary outcomes<br><br>From date of index procedure or date of discharge = unclear<br><br>Planned or unplanned readmission or both = unclear<br><br>All-cause or cause-specific = unclear	Chart review	A patient was excluded from a given category if the data field for that category was incomplete for that patient	No patients were excluded due to incomplete data for 30-day readmission<br><br>Two patients were excluded from the 30-day (mortality) analysis due to loss to follow-up. They were also excluded from the readmission analysis as a result of this exclusion	None	Proportion = Not reported<br><br>Likelihood of substantial loss to follow-up = High likelihood
Charette 2019	30-day readmission primary outcome (or part of primary outcome) = one of the primary outcomes<br><br>From date of index procedure or date of discharge = index procedure<br><br>Planned or unplanned readmission or both = unclear<br><br>All-cause or cause-specific = unclear	Unclear. Some sort of extraction from an institutional electronic database	Unclear	Complete 2-year clinical data were lacking in 5.3% of patients (5.4% in the older group and 4.6% in the younger group)	None	Proportion = Not reported<br><br>Likelihood of substantial loss to follow-up = High likelihood
Courtney 2018	30-day readmission primary outcome (or part of primary outcome) = one of the primary outcomes<br><br>From date of index procedure or date of	As per NSQIP documentation briefly described and referenced in the paper	Not reported	Not reported	Not reported	Proportion = Not reported<br><br>Likelihood of substantial loss to follow-up = Unlikely.

	discharge = index procedure<br><br>Planned or unplanned readmission or both = unclear<br><br>All-cause or cause-specific = unclear					
Curtis 2018	30-day readmission primary outcome (or part of primary outcome) = one of the primary outcomes<br><br>From date of index procedure or date of discharge = index procedure, as per NSQIP documentation<br><br>Planned or unplanned readmission or both = unclear<br><br>All-cause or cause-specific = unclear	As per NSQIP documentation referenced in the paper	Variables that were <80% complete were excluded from analysis. Otherwise there is no mention of missing data<br><br>No mention of readmission data only being included in NSQIP from 2011 onwards	Not reported	Not reported	Proportion = Not reported<br><br>Likelihood of substantial loss to follow-up = Unlikely.
Curtis 2019	30-day readmission primary outcome (or part of primary outcome) = one of the primary outcomes<br><br>From date of index procedure or date of discharge = index procedure, as per NSQIP documentation<br><br>Planned or unplanned readmission or both = unclear	As per NSQIP documentation referenced in the paper	Patients with unknown preoperative functional status were excluded	1126 (0.571% of initial cohort before exclusions)	Not reported	Proportion = Not reported<br><br>Likelihood of substantial loss to follow-up = Unlikely.

	All-cause or cause-specific = unclear					
D'Apuzzo 2017	30-day readmission primary outcome (or part of primary outcome) = primary<br><br>From date of index procedure or date of discharge = index procedure<br><br>Planned or unplanned readmission or both = unclear<br><br>All-cause or cause-specific = all-cause (due to any diagnosis); TKA-specific (any of the 8 diagnoses the CMS considers to be TKA-specific), expanded TKA-specific (22 additional diagnoses considered to be TKA-specific according to REFERENCE (Pierce 2015 – How we measured surgical complications))	SPARCS collects hospital discharge information for every non-federal-hospital discharge, ambulatory surgery case, and emergency department visit in NY State. State regulations require that inpatient data be submitted according to a designated format and schedule by all facilities certified for inpatient care	Missing category created for race/ethnicity<br><br>'Study subjects' section states that patients were excluded where there was a 'lack of complete information' but this was not elaborated on further	Not reported	The 377,705 patients included in the study comprised only those who were eligible for 30-day follow-up, however the authors acknowledged in the Discussion section that patients readmitted to hospitals outside of New York State would not have been captured in SPARCS.<br>Also, according to the information reported in the 'Method of measurement' column of this table, patients readmitted to federal hospitals would not have been captured either.	Proportion = Not reported<br><br>Likelihood of substantial loss to follow-up = Unlikely.
George 2018	30-day readmission primary outcome (or part of primary outcome) = primary<br><br>From date of index procedure or date of discharge = index procedure	As per NSQIP protocol described and referenced in the paper: trained clinical reviewers prospectively collect information on 30-day outcomes for patients in NSQIP hospitals	Excluded patients with missing BMI data entirely from the study<br><br>Variables with missing values were excluded from the multivariate model because less than	403 excluded due to missing BMI data.<br><br>The following variables had missing values: gender (105), race (180), anaesthesia (15), ASA class (123),	Not reported	Proportion = Not reported<br><br>Likelihood of substantial loss to follow-up = Unlikely.

	Planned or unplanned readmission or both = unclear<br><br>All-cause or cause-specific = unclear		2% of the patients had missing values	functional status (1000), readmission (2416), reoperation (488), mortality (1)		
Gwam 2020	30-day readmission primary outcome (or part of primary outcome) = one of the primary outcomes<br><br>From date of index procedure or date of discharge = index procedure<br><br>Planned or unplanned readmission or both = unclear<br><br>All-cause or cause-specific = related to index procedure	As per NSQIP protocols, described and referenced in the Methods section of the paper	Multiple imputation	Not reported	Not reported	Proportion = Not reported<br><br>Likelihood of substantial loss to follow-up = Low likelihood
Hanly 2017	30-day readmission primary outcome (or part of primary outcome) = primary<br><br>From date of index procedure or date of discharge = unclear<br><br>Planned or unplanned readmission or both = unclear<br><br>All-cause or cause-specific = all-cause	Not reported	Patients with missing BMI data were excluded<br><br>Otherwise no mention of other missingness	Excluded due to missing BMI data = 835	Not reported	Proportion = Not reported<br><br>Likelihood of substantial loss to follow-up = High likelihood
Hart 2016	30-day readmission primary outcome (or part of primary outcome) = primary	As per NSQIP documentation referenced in the paper	Patients with missing demographic	Not reported	Not reportedotte	Proportion = Not reported<br><br>Likelihood of substantial loss to follow-up = Unlikely.

	From date of index procedure or date of discharge = index procedure for NSQIP<br><br>Planned or unplanned readmission or both = unplanned<br><br>All-cause or cause-specific = all-cause, but the authors also gathered data on the specific cause for readmission		information were excluded. Otherwise approach not stated			
Jauregui 2015	30-day readmission primary outcome (or part of primary outcome) = primary<br><br>From date of index procedure or date of discharge = index procedure for NSQIP<br><br>Planned or unplanned readmission or both = unplanned<br><br>All-cause or cause-specific = all-cause, but the authors also gathered data on the specific cause for readmission	Not reported	Not reported	N/A	Not reported	Proportion = Not reported<br><br>Likelihood of substantial loss to follow-up = Unlikely.
Jorgensen 2013	30-day readmission primary outcome (or part of primary outcome) = both (all-cause readmission was the primary outcome; readmission possibly	Utilising each patient's unique social security number, cross-referencing between LCFC database (LCDB)	Excluded patients with missing data regarding alcohol use or smoking.	71 (2.3%) from the combined overall THA + TKA cohort	Since the DNHR registers all hospitalisations, readmissions, and surgical procedures performed anywhere in Denmark, regardless of	Proportion = Not reported<br><br>Likelihood of substantial loss to follow-up = Unlikely.

	related to smoking or alcohol use was the secondary outcome)<br><br>From date of index procedure or date of discharge = index procedure<br><br>Planned or unplanned readmission or both = unclear<br><br>All-cause or cause-specific = unclear	and the Danish National Health Register (DNHR)			hospital localisation, and all Danish hospitals need to report to DNHR to receive reimbursement, it is possibly to achieve 100% follow-up	
Jorgensen 2017	30-day readmission primary outcome (or part of primary outcome) = secondary outcome<br><br>From date of index procedure or date of discharge = index procedure<br><br>Planned or unplanned readmission or both = unclear<br><br>All-cause or cause-specific = unclear	Readmission requiring an overnight stay in hospital obtained from the Danish National Patient Registry (DNPR)	Cases with missing covariates were excluded from the logistic regression model	Only reported for the following (presumably no missingness in other variables):<br>Living in institution: MP = 10; non-MP = 0<br>Use of walking aids: MP = 21; non-MP = 30<br>BMI: MP = 13; non-MP = 5<br>Smoking status: MP = 7; non-MP = 0<br>Alcohol use: MP = 15; non-MP = 19<br>NIDDM: MP = 7; non-MP = 14<br>Preoperative anaemia: MP = 26; non-MP = 50<br>Pharmacologically treated cardiac disease: MP = 7; non-MP = 18	Virtually guaranteed 100% follow-up, because reporting to the DNPR is required to receive reimbursement from the Danish government	Proportion = Not reported<br><br>Likelihood of substantial loss to follow-up = Unlikely.

				Pharmacologically treated pulmonary disease: MP = 2; non-MP = 0<br>Pharmacologically treated psychiatric disorder: MP = 4; non-MP = 7<br>Anticoagulant therapy: MP = 0; non-MP = 0		
Keeney 2015	30-day readmission primary outcome (or part of primary outcome) = primary outcome<br><br>From date of index procedure or date of discharge = index procedure<br><br>Planned or unplanned readmission or both = unclear<br><br>All-cause or cause-specific = unclear	Unclear	Only reference made to missingness is exclusion of patients whose socioeconomic status could not be determined. Otherwise not reported	Not reported	This is a single-institution study, but Medicare readmissions are reported to the hospital if the patient is readmitted to a different institution	Proportion = Not reported<br><br>Likelihood of substantial loss to follow-up = Moderate. Medicare readmissions are reported to the hospital, however the authors acknowledged that there may be higher rates of readmission among commercially insured patients that may be under-reported and would decrease the differences in readmission between socioeconomically disadvantaged and minority groups in the study
Kester 2016	30-day readmission primary outcome (or part of primary outcome) = one of the secondary outcomes<br><br>From date of index procedure or date of discharge = index procedure<br><br>Planned or unplanned readmission or both = unplanned	As per NSQIP protocol for trained data collectors, patients are followed for 30 days after index operation and postoperative complications are collected regardless of whether the patient has been readmitted to another hospital	Complete case analysis – excluded all patients with missing or “null” preoperative variables	Not reported	See ‘method of measurement’ column	Proportion = Not reported<br><br>Likelihood of substantial loss to follow-up = Unlikely

	All-cause or cause-specific = unclear					
Kheir 2014	30-day readmission primary outcome (or part of primary outcome) = yes, it was the primary outcome<br><br>From date of index procedure or date of discharge = index procedure<br><br>Planned or unplanned readmission or both = unplanned<br><br>All-cause or cause-specific = 'unforeseen causes'	Obtained from each institution's clinical data warehouse, supplemented by chart review	Not reported	Not reported	None, but the authors acknowledged this as a limitation and made a comment about the fact that the hospital does service most of the local community but still it is those patients outside of the local community who came to the hospital for their TKA that would be missed if readmitted to a separate hospital	Proportion = Not reported<br><br>Likelihood of substantial loss to follow-up = Unlikely
Kim 2019	30-day readmission primary outcome (or part of primary outcome) = yes, it was one of the primary outcomes<br><br>From date of index procedure or date of discharge = index procedure<br><br>Planned or unplanned readmission or both = both<br><br>All-cause or cause-specific = all-cause	Not reported	Only reported for the following:<br>Patients without continuous enrolment in Medicare Parts A, B, and D at baseline were excluded<br><br>Patients without claims data at baseline were excluded	506687 patients without continuous enrolment in Medicare Parts A, B, and D at baseline<br><br>26 patients without claims data at baseline were excluded	Not reported	Proportion = Not reported<br><br>Likelihood of substantial loss to follow-up = Unlikely
Kuo 2017	30-day readmission primary outcome (or part of primary outcome) = yes, it was	Not reported	Not reported	Not reported	Not reported	Proportion = Not reported<br><br>Likelihood of substantial loss to follow-up = High likelihood

	one of the primary outcomes<br><br>From date of index procedure or date of discharge = unclear<br><br>Planned or unplanned readmission or both = unclear<br><br>All-cause or cause-specific = unclear					
Kurtz 2016	30-day readmission primary outcome (or part of primary outcome) = yes, it was the primary outcome<br><br>From date of index procedure or date of discharge = date of discharge<br><br>Planned or unplanned readmission or both = unclear, but seems to be both considering the method of measurement<br><br>All-cause or cause-specific = unclear, but seems to be both considering the method of measurement	Readmission was determined as the appearance of a new hospital claims record for the patient within 30 days of the patient's discharge date.<br>Patients who died within 30 days of discharge were considered to be censored.	Not reported	Not reported	Not reported	Proportion = Not reported<br><br>Likelihood of substantial loss to follow-up = Unlikely
Lehtonen 2018	30-day readmission primary outcome (or part of primary outcome) = yes, it was the primary outcome	As per NSQIP documentation briefly described and referenced in the paper	Variables with less than 80% data completion were excluded from the multivariate analysis	Not reported	Not reported	Proportion = Not reported<br><br>Likelihood of substantial loss to follow-up = Unlikely

	From date of index procedure or date of discharge = unclear<br><br>Planned or unplanned readmission or both = unclear<br><br>All-cause or cause-specific = all-cause					
Liao 2016	30-day readmission primary outcome (or part of primary outcome) = yes, it was one of the primary outcomes<br><br>From date of index procedure or date of discharge = index procedure<br><br>Planned or unplanned readmission = unclear<br><br>All-cause or cause-specific = unclear	Unclear	Not reported	Not reported	Not reported	Proportion = Not reported<br><br>Likelihood of substantial loss to follow-up = Unlikely
Lovecchio 2014	30-day readmission primary outcome (or part of primary outcome) = one of the primary outcomes<br><br>From date of index procedure or date of discharge = unclear<br><br>Planned or unplanned readmission or both = unclear<br><br>All-cause or cause-specific = unclear	As per NSQIP documentation referenced in the paper	Not reported<br><br>No mention of readmission data only being included in NSQIP from 2011 onwards	Not reported	Not reported	Proportion = Not reported<br><br>Likelihood of substantial loss to follow-up = Unlikely

Miric 2014	30-day readmission primary outcome (or part of primary outcome) = yes, it was one of the primary outcomes<br><br>From date of index procedure or date of discharge = index procedure<br><br>Planned or unplanned readmission or both = unclear<br><br>All-cause or cause-specific = unclear	Described previously (see reference 10 of the paper)	Unclear	Unclear	Not reported	Proportion = Not reported<br><br>Likelihood of substantial loss to follow-up = Unlikely
Mudumbai 2019	30-day readmission primary outcome (or part of primary outcome) = yes, it was the primary outcome<br><br>From date of index procedure or date of discharge = discharge<br><br>Planned or unplanned readmission or both = from discussion section: “not able to account for…planned admissions for staged procedures”<br><br>All-cause or cause-specific = All-cause	VHA electronic medical records, including VHA National Patient Care Database and VHA Managerial Cost Accounting pharmacy files. Exact process by which readmissions were identified is unclear.	Complete case analysis – all patients with any missing data were excluded	Unclear. Figure 1 shows that 5053 (6.36%) were excluded due to missing data from the 79,418 patients remaining in the sample prior to applying the final exclusion criteria (i.e. patients who did not undergo TKA) – therefore it is unclear how many TKA patients were among those excluded	Not reported	Proportion = Not reported<br><br>Likelihood of substantial loss to follow-up = Moderate. Readmissions to any VHA facility were captured, but from patients readmitted to non-VHA facilities were not. Therefore loss to follow up is likely if patients who undergo TKA at a VHA facility are likely to be readmitted to non-VHA facilities but this is unclear
Nowak and Schemitsch 2019	30-day readmission primary outcome (or part of primary outcome) = secondary	As per NSQIP documentation referenced in the paper	As readmission was only included in the NSQIP database from 2011 onwards, for this model we	Not reported (for any variables, nor for pre-2011 population)	Not reported	Proportion = Not reported<br><br>Likelihood of substantial loss to follow-up = Unlikely

	From date of index procedure or date of discharge = index procedure<br><br>Planned or unplanned readmission or both = unclear<br><br>All-cause or cause-specific = unclear		included only patients between 2011 and 2016.<br>No mention of approach to missing data in other variables			
Ottesen 2018	30-day readmission primary outcome (or part of primary outcome) = yes, one of the primary outcomes<br><br>From date of index procedure or date of discharge = unclear<br><br>Planned or unplanned readmission or both = unclear<br><br>All-cause or cause-specific = unclear	As per NSQIP documentation briefly described and referenced in the paper	Complete cohort analysis to address all missing data	Unclear, but authors stated that less than 5% of data were missing from all variables	Not reported	Proportion = Not reported<br><br>Likelihood of substantial loss to follow-up = Unlikely
Patel 2020	30-day readmission primary outcome (or part of primary outcome) = one of the primary outcomes<br><br>From date of index procedure or date of discharge = discharge<br><br>Planned or unplanned readmission or both = unclear<br><br>All-cause or cause-specific = all-cause	Not reported	Not reported	Not reported	Not reported	Proportion = Not reported<br><br>Likelihood of substantial loss to follow-up = Low likelihood

Patterson 2018	30-day readmission primary outcome (or part of primary outcome) = no, it was a secondary outcome<br><br>From date of index procedure or date of discharge = index procedure<br><br>Planned or unplanned readmission or both = unclear<br><br>All-cause or cause-specific = unclear	As per NSQIP documentation briefly described and referenced in the paper	Not reported	Not reported	No strategy reported	Proportion = Not reported<br><br>Likelihood of substantial loss to follow-up = Unlikely
Peskun 2012	30-day readmission primary outcome (or part of primary outcome) = primary outcome<br><br>From date of index procedure or date of discharge = unclear<br><br>Planned or unplanned readmission or both = unclear<br><br>All-cause or cause-specific = unclear	Data abstraction form applied to hospital charts	Not reported	Not reported	Not reported	Proportion = Not reported<br><br>Likelihood of substantial loss to follow-up = High likelihood
Pugely 2013	30-day readmission primary outcome (or part of primary outcome) = yes, it was the primary outcome<br><br>From date of index procedure or date of discharge = unclear	As per NSQIP documentation briefly described and referenced in the paper	Due to statistical limitations, patients with missing data could not be included in the multivariate logistic regression model	Not reported	All readmissions, regardless of treating hospitals, were captured	Proportion = Not reported<br><br>Likelihood of substantial loss to follow-up = Unlikely

	Planned or unplanned readmission or both = planned readmissions were included (e.g. for oncologic therapies)<br><br>All-cause or cause-specific = unclear		Patients missing readmissions data were excluded<br><br>Variables with chart completion rates <80% (e.g. albumin) were excluded from the multivariate logistic regression model to avoid over-fitting			
Ramos 2014	30-day readmission primary outcome (or part of primary outcome) = yes, it was the primary outcome<br><br>From date of index procedure or date of discharge = index procedure<br><br>Planned or unplanned readmission or both = unplanned<br><br>All-cause or cause-specific = surgical (such as wound infection or fracture) or medical (such as pulmonary embolus or deep vein thrombosis)	Unclear	Not reported	Not reported	Not reported	Proportion = Not reported<br><br>Likelihood of substantial loss to follow-up = High likelihood
Ricciardi 2017	30-day readmission primary outcome (or part of primary outcome) = yes, it was the primary outcome<br><br>From date of index procedure or date of	Identified from administrative claims data and confirmed through an institutional registry for TKA. Data regarding the readmission were obtained from post-	Not recorded	Not recorded	None, but the authors acknowledge the potential for readmission to non-index institutions, and suggest their data are more reflective of surgical complications rather than medical	Proportion = Not reported<br><br>Likelihood of substantial loss to follow-up = High likelihood

	discharge = index procedure<br><br>Planned or unplanned readmission or both = unplanned<br><br>All-cause or cause-specific = surgical (such as wound infection or fracture) or medical (such as pulmonary embolus or deep vein thrombosis)	discharge records. For patients who had more than one operation and had readmission for one of the operations, the second operation was excluded if the readmission was associated with the first operation. For patients who had more than one operation without subsequent readmissions, both operations were included in the cohort. If the readmission was associated with the second operation, the first operation was not excluded.			complications. They provide an in-depth discussion of this, albeit without any confirmatory statistical analyses, in the section of the Discussion pertaining to study limitations (page 1079, final paragraph before Conclusion).	
Robinson 2017	30-day readmission primary outcome (or part of primary outcome) = no, secondary outcome<br><br>From date of index procedure or date of discharge = index procedure<br><br>Planned or unplanned readmission or both = unplanned<br><br>All-cause or cause-specific = unclear	Abstracted as per NSQIP protocol from medical records, operative reports, and patient interviews	Patients with incomplete demographics data were excluded	Not reported	Not reported	Proportion = Not reported<br><br>Likelihood of substantial loss to follow-up = Unlikely

Ross 2020	30-day readmission primary outcome (or part of primary outcome) = primary outcome<br><br>From date of index procedure or date of discharge = discharge<br><br>Planned or unplanned readmission or both = both<br><br>All-cause or cause-specific = all-cause	Data from a provincially held and validated registry, the Institute for Clinical and Evaluative Sciences (IC/ES), were used for this study. Reporting to the Canadian Joint Replacement Registry is mandatory for TKAs performed in Ontario	Not reported	Not reported	Not reported	Proportion = Not reported<br><br>Likelihood of substantial loss to follow-up = Moderate
Roth 2019	30-day readmission primary outcome (or part of primary outcome) = yes, it was the primary outcome<br><br>From date of index procedure or date of discharge = unclear<br><br>Planned or unplanned readmission or both = unclear<br><br>All-cause or cause-specific = unclear	As per NSQIP documentation briefly described and referenced in the paper	Patients with missing BMI data were excluded<br><br>Otherwise there is no mention of missing data in other variables<br><br>No mention of readmission data only being included in NSQIP from 2011 onwards	Missing BMI values = 240 (1.1% of initial sample)	Not reported	Proportion = Not reported<br><br>Likelihood of substantial loss to follow-up = Unlikely
Rudasill 2019	30-day readmission primary outcome (or part of primary outcome) = secondary<br><br>From date of index procedure or date of discharge = discharge	Unclear	Excluded cases with missing INR data. No mention of approach to missing data in other variables<br><br>No mention of readmission data only being included	Only reported for INR (see exclusion criteria)	Not reported	Proportion = Not reported<br><br>Likelihood of substantial loss to follow-up = Unlikely

	Planned or unplanned readmission or both = unclear<br><br>All-cause or cause-specific = all-cause and TKA-specific		in NSQIP from 2011 onwards			
Runner 2017	30-day readmission primary outcome (or part of primary outcome) = no, secondary outcome<br><br>From date of index procedure or date of discharge = unclear<br><br>Planned or unplanned readmission or both = unclear<br><br>All-cause or cause-specific = unclear	As per NSQIP documentation briefly described and referenced in the paper	Not reported<br><br>No mention of readmission data only being included in NSQIP from 2011 onwards	Not reported	Not reported	Proportion = Not reported<br><br>Likelihood of substantial loss to follow-up = Unlikely
Saucedo 2014	30-day readmission primary outcome (or part of primary outcome) = yes, primary outcome<br><br>From date of index procedure or date of discharge = discharge<br><br>Planned or unplanned readmission or both = unplanned<br><br>All-cause or cause-specific = all-cause	Not reported	Not reported	Not reported	None, but the authors compared their readmission rates to those reported in other studies and found them to be comparable. They also noted that it is common practice that patients admitted to other facilities are transferred back to their institution for definitive care	Proportion = Not reported<br><br>Likelihood of substantial loss to follow-up = High likelihood
Schaeffer 2015	30-day readmission primary outcome (or part of primary	Unclear method of measurement.	Not reported	Not reported	None, and the authors acknowledged this as a limitation of single-institution studies	Proportion = Not reported<br><br>Likelihood of substantial loss to follow-up = High likelihood

	outcome) = yes, primary outcome<br><br>From date of index procedure or date of discharge = unclear<br><br>Planned or unplanned readmission or both = unclear<br><br>All-cause or cause-specific = unclear					
Schairer 2014	30-day readmission primary outcome (or part of primary outcome) = secondary outcome<br><br>From date of index procedure or date of discharge = discharge<br><br>Planned or unplanned readmission or both = planned (i.e. predetermined at the time of original procedure, e.g. staged antibiotic spacer exchange for revision), and unplanned otherwise<br><br>All-cause or cause-specific = grouped into surgical and medical causes	Hospital administrative claims data with confirmatory medical record review	294 patients were excluded for insufficient follow-up. Otherwise an overall approach to missing data was not reported	294 excluded	None. Authors acknowledged that this is a particular concern for tertiary referral centres such as theirs, as patients often travel long distances to undergo TKA and therefore are more likely to be readmitted to a different institution.	Proportion = Not reported<br><br>Likelihood of substantial loss to follow-up = High likelihood
Singh 2013	30-day readmission primary outcome (or part of primary outcome) = yes, one of the primary outcomes	Unclear	Not reported, apart from 'missing' class created for insurance type (see	Not reported	Not reported	Proportion = Not reported<br><br>Likelihood of substantial loss to follow-up = Unlikely

	From date of index procedure or date of discharge = index procedure<br><br>Planned or unplanned readmission or both = both<br><br>All-cause or cause-specific = all-cause		Table 2 of the paper)			
Siracuse 2017	30-day readmission primary outcome (or part of primary outcome) = yes, primary outcome<br><br>From date of index procedure or date of discharge = index procedure<br><br>Planned or unplanned readmission or both = both, excepted visits for rehabilitation as identified using ICD-9 codes<br><br>All-cause or cause-specific = all-cause	Unclear	Patients with a missing numeric identifier variable used to track repeated visits were excluded.<br><br>Otherwise, no mention of how missing data were handled	Not reported	Not reported	Proportion = Not reported<br><br>Likelihood of substantial loss to follow-up = Unclear
Sloan 2020	30-day readmission primary outcome (or part of primary outcome) = one of the primary outcomes<br><br>From date of index procedure or date of discharge = index procedure	Not reported	Not reported	Not reported	Not reported	Proportion = Not reported<br><br>Likelihood of substantial loss to follow-up = Low likelihood

	Planned or unplanned readmission or both = unclear<br><br>All-cause or cause-specific = all-cause					
Sodhi and Anis et al 2019	30-day readmission primary outcome (or part of primary outcome) = secondary<br><br>From date of index procedure or date of discharge = unclear<br><br>Planned or unplanned readmission or both = unclear<br><br>All-cause or cause-specific = unclear	Unclear	Excluded cases with missing data (0.9% of all cases extracted)	Not reported	Not reported	Proportion = Not reported<br><br>Likelihood of substantial loss to follow-up = Unlikely
Sodhi and Mont 2019	30-day readmission primary outcome (or part of primary outcome) = one of the primary outcomes<br><br>From date of index procedure or date of discharge = unclear<br><br>Planned or unplanned readmission or both = unclear<br><br>All-cause or cause-specific = unclear	Unclear	Not reported	Not reported	Not reported	Proportion = Not reported<br><br>Likelihood of substantial loss to follow-up = Moderate
Suleiman 2015	30-day readmission primary outcome (or part of primary outcome) = one of the primary outcomes	According to the NSQIP User Guide (referenced in the article)	Variables with over 50% missing data were excluded<br><br>No mention of readmission data	Unclear	Not reported	Proportion = Not reported<br><br>Likelihood of substantial loss to follow-up = Unlikely

	From date of index procedure or date of discharge = index procedure<br><br>Planned or unplanned readmission or both = unclear<br><br>All-cause or cause-specific = unclear		only being included in NSQIP from 2011 onwards			
Sutton 2016	30-day readmission primary outcome (or part of primary outcome) = secondary<br><br>From date of index procedure or date of discharge = index procedure<br><br>Planned or unplanned readmission or both = both<br><br>All-cause or cause-specific = all-cause	According to the NSQIP User Guide (referenced in the article)	Variables with a medical record completion rate of <85% were excluded<br><br>Patients were excluded from the multivariate logistic regression model due for missing values	3367 patients were excluded from the multivariate logistic regression model due for missing values	Not reported	Proportion = Not reported<br><br>Likelihood of substantial loss to follow-up = Unlikely
Tang 2019	30-day readmission primary outcome (or part of primary outcome) = primary outcome<br><br>From date of index procedure or date of discharge = index procedure<br><br>Planned or unplanned readmission or both = unplanned (all due to complications)	Not reported	Multiple imputation	Not reported. However, the authors noted (second page of article) that "a lot" of missing data were present variables such as the following required multiple imputation: race, BMI, type of anaesthesia, elevated preoperative creatinine	Not reported	Proportion = Not reported<br><br>Likelihood of substantial loss to follow-up = Moderate

	All-cause or cause-specific = complication-specific (i.e. complications that led to readmission were included)			>2mg/dL, and diabetes mellitus on insulin		
Tay 2017	30-day readmission primary outcome (or part of primary outcome) = secondary<br><br>From date of index procedure or date of discharge = discharge<br><br>Planned or unplanned readmission or both = both<br><br>All-cause or cause-specific = all-cause	Medical records were extensively reviewed to ensure accurate capture of information, especially with regards to complications and comorbidities. All data were collected by independent observers not participating in the care of the patients.	Not reported	Unclear	Not reported	Proportion = Not reported<br><br>Likelihood of substantial loss to follow-up = High likelihood
Urish 2018	30-day readmission primary outcome (or part of primary outcome) = secondary<br><br>From date of index procedure or date of discharge = index procedure<br><br>Planned or unplanned readmission or both = both<br><br>All-cause or cause-specific = all-cause	NRD is part of the Healthcare Cost and Utilization Project (HCUP), sampled from the State Inpatient Database. Linkage is used to follow patients such that readmissions across different hospitals are tracked	Not reported	Unclear	NRD was designed specifically to track readmissions	Proportion = Not reported<br><br>Likelihood of substantial loss to follow-up = Unlikely
Webb 2017	30-day readmission primary outcome (or part of primary	According to NSQIP documentation	The authors analysed only those cases for whom readmission data	Unclear	Not reported	Proportion = Not reported<br><br>Likelihood of substantial loss to follow-up = Unlikely

	outcome) = one of the primary outcomes<br><br>From date of index procedure or date of discharge = index procedure<br><br>Planned or unplanned readmission or both = both<br><br>All-cause or cause-specific = all-cause	(referenced in the article)	was available (i.e. those who underwent surgery in 2011+), but otherwise there is no mention of missing data			
Weick 2018	30-day readmission primary outcome (or part of primary outcome) = one of the primary outcomes<br><br>From date of index procedure or date of discharge = index procedure<br><br>Planned or unplanned readmission or both = unclear<br><br>All-cause or cause-specific = all-cause	From 'Data Source' section of Materials and Methods: ICD-9 and CPT-4 codes	Not reported	Unclear	Not reported	Proportion = Not reported<br><br>Likelihood of substantial loss to follow-up = Unclear
Welsh 2017	30-day readmission primary outcome (or part of primary outcome) = one of the primary outcomes<br><br>From date of index procedure or date of discharge = discharge	Direct quote: "Unplanned readmissions were coded using the methodology from CMS' hospital-wide readmission measure.25 The variable was coded as a dichotomous (yes/no) variable for the 30-day	Patients with missing data on patient or clinical characteristics were excluded	461 (per-prognostic factor information not available)	Not reported	Proportion = Not reported<br><br>Likelihood of substantial loss to follow-up = Unlikely

	Planned or unplanned readmission or both = unplanned<br><br>All-cause or cause-specific = all-cause	readmission logistic regression analysis." "Reasons for hospital readmission were obtained using the Medicare Severity-Diagnosis Related Groups (MS-DRG) diagnostic codes in the MedPAR files."				
Workman 2019	30-day readmission primary outcome (or part of primary outcome) = primary outcome<br><br>From date of index procedure or date of discharge = index procedure<br><br>Planned or unplanned readmission or both = unplanned<br><br>All-cause or cause-specific = all-cause, but specific causes were documented	Patient readmission within 30 days from index procedure was confirmed by electronic record review and chart exploration.	Not reported	Not reported	None, although the authors do acknowledge this as a limitation in the discussion section.	Proportion = Not reported<br><br>Likelihood of substantial loss to follow-up = High likelihood
Yohe 2018	30-day readmission primary outcome (or part of primary outcome) = one of the primary outcomes<br><br>From date of index procedure or date of discharge = unclear<br><br>Planned or unplanned readmission or both = unplanned	Not reported	Not reported<br><br>No mention of readmission data only being included in NSQIP from 2011 onwards	Not reported	Not reported	Proportion = Not reported<br><br>Likelihood of substantial loss to follow-up = Unlikely

	All-cause or cause-specific = unclear					
Zusmanovic 2018	30-day readmission primary outcome (or part of primary outcome) = one of the primary outcomes<br><br>From date of index procedure or date of discharge = unclear<br><br>Planned or unplanned readmission or both = unplanned<br><br>All-cause or cause-specific = unclear	As per NSQIP documentation referenced in the paper	Not reported<br><br>No mention of readmission data only being included in NSQIP from 2011 onwards	Not reported	Not reported	Proportion = Not reported<br><br>Likelihood of substantial loss to follow-up = Unlikely

S6 - Prognostic factor selection

Study ID	**Method of prognostic factor measurement:**	Prognostic factors (reference category – if applicable):	**Events per predictor variable (or mean value for readmitted group, for continuous predictor variables):**	**Categorisation of continuous predictor variables or covariates:**
Abdulla 2020	Not reported	BMI categories:<br>Normal weight (18.5-24.9)<br>Overweight (25-29.9)<br>Obese class I (30-34.9)<br>Obese class II (35-39.9)<br>Obese class III (≥40)	Not reported	BMI was categorised according to the WHO system<br><br>Presurgical risk factors were categorised as present or absent into 0, 1-2, and ≥3
Abola 2018	As per NSQIP protocols, described and referenced in the paper	Hyponatraemia = sodium <135 mEq/L (normonatraemia)<br>Age (<40)<br>BMI (20-35)<br>Non-elective operation<br>Emergency operation<br>Female sex<br>Non-white race<br>ASA class (1)<br>Bleeding disorders<br>COPD<br>CHF<br>Diabetes<br>Dialysis – current<br>Disseminated cancer<br>Dyspnoea<br>Functional dependence<br>Hypertension<br>Renal failure (acute, preoperative)<br>Smoking<br>SIRS/sepsis/septic shock<br>Steroids for chronic condition<br>Weight loss<br>Preoperative wound infection/open wound	Not reported	Serum sodium level was categorised (hyponatraemia = serum sodium <135; normonatraemia = 135-145; hypernatraemia = >145), and patients with hypernatraemia were excluded<br><br>Age was categorised (<40, 40-59, 60-79, 80+)<br><br>BMI was categorised (<20, 20-35, >35)<br><br>Comorbidities were dichotomised as present or absent
Ali 2019	ICD-10 codes, assigned by trained clinical coders	Age group (60-64)<br>Male sex<br>SES quintile (1 (least deprived))	Not available for TKA-only cohort (only reported for combined cohort: TKA + unicompartmental knee arthroplasty + patellofemoral arthroplasty)	Age was categorised (0-39, 40-44, 45-49, 50-54, 55-59, 60-64, 65-

		Number of prior emergency admissions (0)<br>Ethnicity (white)<br>Diabetes mellitus<br>Hypertension<br>Arrhythmias<br>Valvular heart disease<br>Congestive heart failure<br>Peripheral vascular disease<br>Chronic pulmonary disease<br>Pulmonary circulatory disease<br>Metastases<br>Renal disease<br>Dementia<br>Psychoses<br>Alcohol abuse<br>Drug abuse<br>Depression<br>Other mental health disorder<br>Living alone (reference category not reported)<br>Liver disease<br>Peptic ulcer disease<br>Paraplegia<br>Anemia due to blood loss<br>Iron deficiency anemia<br>Coagulopathy<br>Recent weight loss<br>Fluid balance abnormality<br>Hypothyroidism<br>Obesity (definition not given; reference category not reported)<br>Other neurological disorder<br>Rheumatological disorder<br>Previous pneumonia<br>Previous stroke<br>Previous AMI<br>Cancer diagnosis		69, 70-74, 75-79, 80-84, 85-89, 90+)<br><br>Comorbidities were dichotomised as present or absent<br><br>Socioeconomic status was measured as quintiles<br><br>Number of prior emergency admissions was categorised as 0, 1, 2, 3+
Alvi 2015	As per NSQIP protocols, described and referenced in the paper	BMI categories (18.5-25)	Not reported	BMI was categorised (18.5-25, 25-30, 30-35, 35-40, 40+)

				Comorbidities were dichotomised as present or absent<br><br>Age was dichotomised as <75 and ≥75
Anderson 2020	Not reported	Use of medications on the Beers List of Drugs in the 180 days prior to the surgical procedure	Not reported	Use of Beers List medications was dichotomised as present or absent based on prescription fill in the 180 days prior to surgical procedure
Anthony 2018	Not reported	Age group (<18)<br>Primary payer (Medicare)<br>Sex (male)<br>Diabetes<br>Hypertension<br>Obesity (definition not provided; reference category not reported)<br>Number of diagnoses (continuous)<br>Number of procedures (continuous)	Not reported	Comorbidities were dichotomised as present or absent<br><br>Age was categorised (<18, [18,30), [30,40), [40,50), [50,60), [60,70), [70,80), 80+)
Antoniak 2020	As per NSQIP protocols referenced in the paper<br><br>eGFR calculated using Chronic Kidney Disease Epidemiology Collaboration equation (with the Cr value available from NSQIP)	Variables necessary for calculation eGFR (age, gender, race, single preoperative Cr value), and "all variables reported by the ACS NSQIP at any time between 2006 and 2016"	Only available for CKD stage, calculated from % values given in Table 3 and rounded down:<br>No CKD = 330<br>Stage 2 = 1490<br>Stage 3a = 538<br>Stage 3b = 288<br>Stage 4 = 67	eGFR was categorised based on prior research into meaningful risk categories (see the paper for more details) for the analysis of readmission. It was analysed as a continuous variable for the composite outcome, major complications, but not for readmission alone
Arroyo 2019	Identified by ICD-9 codes	Number of knee procedures<br>Age<br>Female<br>Race (white)<br>Payer status (private insurance)<br>Median income level<br>Elixhauser Index	Age = 69.23 (10.81)<br>Male = 15,456; female = 21,446<br>Race: White = 27,477; black = 3489; Hispanic = 3621; Other = 1713; Missing = 502<br>Payer: Medicare = 25,325; Medicaid = 1492; Private insurance = 8439; Other = 1400; Self-pay/No charge = 146	BMI was not analysed. Instead, 'obesity' (without clear definition) was included as a predictor

			Median income quartile of postal (ZIP code): first = 7990, second = 9207, third = 9709, fourth = 9206, missing = 690<br>Elixhauser Index (van Walraven score) = 0 (-1; 2. = median (Q1; Q3))<br>Elixhauser comorbidities: CHF = 1530, valvular disease = 1738, peripheral vascular disorders = 1252, hypertension (uncomplicated) = 23,596, hypertension (complicated) = 3089, other neurological disorders = 1093, chronic pulmonary disease = 6754, diabetes (uncomplicated) = 8214, diabetes (complicated) = 1102, hypothyroidism = 5646, renal failure = 2933, liver disease = 526, solid tumour without metastasis = 331, rheumatoid arthritis/collagen vascular diseases = 1903, coagulopathy = 705, obesity = 8153, fluid and electrolyte disorders = 1352, blood loss anaemia = 201, deficiency anaemia = 3438, alcohol abuse = 445, drug abuse = 317, psychoses = 963, depression = 4440	
Belmont 2016	As per NSQIP protocols, described and referenced in the paper	Age (continuous and categorical – reference = <60): <60, 60-69, 70-79, ≥80<br>Sex (male)<br>BMI (continuous and categorical – reference = <30): ≤29.9, 30.0-39.9, ≥40<br>Functional status (dependent): independent vs partially/totally dependent<br>Wound classification (clean): clean vs clean contaminated/contaminated/ dirty/infected<br>ASA class (1/2): 1/2 vs 3/4<br><br>Preoperative laboratory values (all continuous):<br>White blood cell count<br>Haematocrit<br>Platelets<br>Creatinine<br>Serum albumin	Age categories:<br>≤59.9 = 22<br>60-69 = 37<br>70-79 = 36<br>≥80<br><br>Sex:<br>Male = 57<br>Female = 51<br><br>BMI categories:<br>≤29.9 = 41<br>30.0-39.0 = 45<br>≥40 = 22<br><br>Functional status (data only available for 106 of the total 108 readmissions that occurred in the study population):<br>Independent = 102<br>Partially dependent = 3<br>Totally dependent = 1<br><br>Wound classification:	Comorbidities were dichotomised as present or absent<br><br>Age (<60, 60-69, 70-79, ≥80)<br><br>BMI (<30, 30.0-39.9, ≥40)<br><br>Functional status was dichotomised as independent or partially/totally dependent

		INR<br><br>Individual comorbidities:<br>Recent weight loss - >10% of body weight in last 6 months<br>Smoking – current within 1 year<br>Regular alcohol use – more than 2 drinks per day in the 2 weeks prior to admission<br>All diabetes<br>IDDM<br>NIDDM<br>Dyspnoea<br>COPD<br>Hypertension<br>Cardiac disease<br>CHF<br>Previous myocardial infarction within 6 months<br>PCI<br>Previous cardiac surgery<br>History of angina within 1 month<br>History of revascularisation/amputation for PVD/rest pain/gangrene<br>Impaired sensorium<br>Previous TIA/CVA/stroke with neurologic deficit/CVA/stroke without neurologic defect<br>Dialysis use/renal failure<br>Bleeding disorder<br>Preoperative open wound or wound infection<br>Systemic sepsis<br>Steroid use for chronic infection<br>Chemotherapy for <30 days<br>Radiation therapy for <90 days<br>Prior operation within 30 days<br><br>Overall complications<br><br>Major systemic complications:<br>Pulmonary embolism	Clean = 98<br>Clean/contaminated = 3<br>Contaminated = 2<br>Dirty/infected = 5<br><br>ASA class:<br>1 = 1<br>2 = 34<br>3 = 67<br>4 = 6<br>5 = 0<br><br>Preoperative laboratory values (mean non-readmitted vs mean readmitted):<br>White blood cell count (x103/µL) = 7.2 vs 7.3<br>Haematocrit (%) = 39.3 vs 39.1<br>Platelets (x103/µL) = 249.2 vs 244.6<br>Creatinine = 1.0 vs 1.0<br>Serum albumin (g/dL) = 4.0 vs 3.8<br>INR = 1.1 vs 1.1<br><br>Comorbidities:<br>Recent weight loss - >10% of body weight in last 6 months = 2<br>Smoking – current within 1 year = 12<br>Regular alcohol use – more than 2 drinks per day in the 2 weeks prior to admission = 0<br>All diabetes = 25<br>IDDM = 7<br>NIDDM = 18<br>Dyspnoea = 9<br>COPD = 8<br>Hypertension = 64<br>Cardiac disease = 8<br>CHF = 2<br>Previous myocardial infarction within 6 months = 0<br>PCI = 5<br>Previous cardiac surgery = 2<br>History of angina within 1 month = 1<br>History of revascularisation/amputation for PVD/rest pain/gangrene = 0<br>Impaired sensorium = 0	

		Unplanned intubation<br>Ventilator >48hrs<br>Post-operative sepsis/septic shock combined<br>Stroke/CVA<br>Acute renal failure<br>Cardiac arrest requiring CPR<br>Myocardial infarction<br>Coma<br><br>Minor systemic complications:<br>UTI<br>DVT<br>Pneumonia<br>Progressive renal insufficiency<br><br>Major local complications:<br>Deep wound infection/organ or space SSI combined<br>Peripheral nerve injury<br>Graft/prosthesis failure<br><br>Minor local complications:<br>Superficial wound infection<br>Wound disruption<br><br>Mortality or major complication	Previous TIA/CVA/stroke with neurologic deficit/CVA/stroke without neurologic defect = 6<br>Dialysis use/renal failure = 0<br>Bleeding disorder = 7<br>Preoperative open wound or wound infection = 2<br>Systemic sepsis = 2<br>Steroid use for chronic infection = 5<br>Chemotherapy for <30 days = 0<br>Radiation therapy for <90 days = 0<br>Prior operation within 30 days = 0<br><br>Overall complications = 41<br><br>Major systemic complications (14 total):<br>Pulmonary embolism = 3<br>Unplanned intubation = 0<br>Ventilator >48hrs = 0<br>Post-operative sepsis/septic shock combined = 5<br>Stroke/CVA = 3<br>Acute renal failure = 2<br>Cardiac arrest requiring CPR = 0<br>Myocardial infarction = 1<br>Coma = 0<br><br>Minor systemic complications (11 total):<br>UTI = 4<br>DVT = 4<br>Pneumonia = 1<br>Progressive renal insufficiency = 3<br><br>Major local complications (10 total):<br>Deep wound infection/organ or space SSI combined = 10<br>Peripheral nerve injury = 0<br>Graft/prosthesis failure = 0<br><br>Minor local complications (14 total):<br>Superficial wound infection = 9<br>Wound disruption = 5<br><br>Mortality or major complication = 22	

Bovonratwet 2018	As per NSQIP protocols, briefly described and referenced in the paper	Age ≥80 compared to age <70 and age 70-79	Age ≥80 = 51<br><br>Before propensity score matching:<br>Age <70 = 299<br>Age 70-79 = 122<br><br>After propensity score matching:<br>Age <70 = 46<br>Age 70-79 = 47	Age was categorised (<70, 70-79, ≥80)<br><br>BMI was categorised (18-25, 25-30, 30-35, >35)<br><br>Diabetes mellitus was categorised as: none, NIDDM, IDDM<br><br>Smoking status was dichotomised as yes/no<br><br>Functional status was categorised as dependent/independent
Bovonratwet 2019	As per NSQIP protocols, briefly described and referenced in the paper	Age ≥80 (reference = age <80)	Age <80 = 411 (2.43%)<br>Age ≥80 = 44 (4.43%)	Age was dichotomised as <80 and ≥80<br><br>BMI was categorised (18-25, 25-30, 30-35, >35)<br><br>Diabetes mellitus was categorised as: none, NIDDM, IDDM<br><br>Smoking status was dichotomised as yes/no<br><br>Functional status was categorised as dependent/independent<br><br>Operative duration (minutes) was categorised (<69, 70-89, ≥90)

Bovonratwet 2020	As per NSQIP protocols, described and referenced in the paper	(taken from Table 1):<br>Age<br>Sex<br>BMI<br>Functional status prior to surgery<br>ASA classification<br>Smoker<br>Anaemia<br>Diabetes mellitus<br>Chronic steroid use<br>Hypertension<br>Dyspnoea on exertion<br>COPD<br>Preoperative INR<br>Bleeding disorder	Not reported	Comorbidities were dichotomised as present or absent<br><br>It is unclear whether age was included as a categorised or continuous variable in the multivariate Poisson regression model<br><br>It is unclear whether BMI was included as a categorised or continuous variable in the multivariate Poisson regression model<br><br>Preoperative INR was dichotomised at 1.2 (unit of measurement not reported)
Buitagro 2020	Not reported	Age<br>CCI<br>Geographical region<br>('Insurer' was included in the study, but not analysed in this review because the categories (A to G + others) were not named, therefore are not identifiable)	Age:<br>≤49 = 14/395 (3.54%)<br>50-59 = 63/1981 (1.67%)<br>60-69 = 154/4553 (3.38%)<br>70-79 = 218/4488 (4.86%)<br>≥80 = 34/1036 (8.11%)<br><br>CCI:<br>0 = 408/7010 (5.82%)<br>1-2 = 328/4206 (7.80%)<br>≥3 = 150/1237 (1.7%)<br><br>Geographical region:<br>Atlantic = 54/1488 (3.63%)<br>Bogota = 126/2911 (4.33%)<br>Central = 157/3772 (4.16%)<br>Eastern = 87/1710 (5.09%)<br>Pacific = 104/2534 (4.10%)<br>Other Departments = 5/38 (13.16%)	Age was categorised (≤49, 50-59, 60-69, 70-79, ≥80)
Bullock 2003	Routine documentation of surgery type (unilateral vs bilateral)	Bilateral TKA (reference = unilateral)	Unilateral = 12 (2.3%)	N/A

			Bilateral = 9 (3.6%)	
Charette 2019	Extraction from institutional electronic database<br><br>Data were collected prospectively, but the sample for this study was extracted and analysed retrospectively	Age <55 vs age ≥55	Age <55 = 45 readmissions (6.1%)<br>Age ≥55 = 253 readmissions (7.2%)	Age was dichotomised as <55 and ≥55<br><br>BMI was dichotomised as <30 and >30. It is unclear whether BMI was included as a covariate in the logistic regression model.<br><br>Comorbidities were dichotomised as present or absent. t is unclear which comorbidities were included as a covariate in the logistic regression model.
Courtney 2018	As per NSQIP documentation, referenced and (briefly) described in the paper	Primary predictor variable of interest:<br>TKA revision for infection<br><br>Variables included in the multivariate logistic regression model:<br>Male sex<br>Minority ethnicity<br>Age >70 (reference category not reported)<br>BMI >35 (reference category not reported)<br>Diabetes mellitus<br>Smoking history<br>CHF<br>Hypertension<br>Dialysis<br>Preoperative creatinine >1.5 mg/dL<br>Preoperative albumin <3.5 g/dL<br>ASA ≥4	Infection group = 199 (10%)<br>Aseptic group = 493 (6%)<br><br>Not reported for any other variables	Comorbidities were dichotomised as present or absent<br><br>Age was dichotomised as ≤70 and >70<br><br>BMI was dichotomised as ≤35 and >35<br><br>Preoperative creatinine was dichotomised as ≤1.5 and >1.5 mg/dL<br><br>Preoperative albumin was dichotomised as <3.5 and ≥3.5 g/dL
Curtis 2018	As per NSQIP documentation, referenced and (briefly) described in the paper	Chronic immunosuppressant use, defined as per NSCIP documentation as “the need for	180 readmissions (5.2%) in the immunosuppressant user group	Immunosuppressant use was dichotomised

		oral/IV corticosteroids, or immunosuppressant drugs, for a chronic medical condition within 30 days prior to the surgery or at the time the surgery was scheduled		as present (chronic) or absent<br><br>Comorbidities were dichotomised as present or absent<br><br>ASA class was dichotomised as <3 and ≥3
Curtis 2019	As per NSQIP documentation, referenced and (briefly) described in the paper	Dependent functional status (DEP)	Not reported	Age was dichotomised as ≤75 and >75<br><br>Comorbidities were dichotomised as present or absent<br><br>BMI was dichotomised as ≥40 and <40<br><br>ASA class was dichotomised as <3 and ≥3
D'Apuzzo 2017	Diagnosis codes used to identify prognostic factors coded in the SPARCS database	Age (65-75, 76-85, >85; reference = <65y)<br>Sex (reference = female)<br>Race/ethnicity (Black, Hispanic, Asian, Other, Missing; reference = white))<br>Insurance Status (Medicare, Medicaid, Workers' Compensation, Other; reference = private)<br>Bilateral TKA (reference = unilateral)<br>In-hospital complications<br><br>Comorbidities:<br>CHF, valvular disease, pulmonary circulation disorder, pulmonary vascular disorder, paralysis, other neurological disorder, chronic pulmonary disease, diabetes, renal failure, liver disease, peptic ulcer	All-cause:<br><br>Mean age (SD) = 69.2 (10.5)<br>Sex: male = 8,154 (36.9%); female = 13,922 (63.1%)<br>Race/ethnicity: White = 16,619 (75.3%); Black = 1,983 (9.0%); Hispanic = 1,122 (5.1%); Asian = 197 (0.9%); Other = 876 (4.0%); Missing = 1,279 (5.8%)<br>Insurance status: Medicare = 14,233 (64.5%); Medicaid = 856 (3.9%); Private = 5,975 (27.1%); Worker's compensation = 651 (2.9%); Other = 361 (1.6%)<br>Surgical indication: osteoarthritis = 20,705 (93.8%); inflammatory arthritis = 1,205 (5.5%); Osteonecrosis = 166 (0.8%)<br>Bilateral TKA = 2,291 (10.4%)<br>In-hospital complication: medical = 1,413 (6.4%); surgical = 798 (3.6%)	Comorbidities were dichotomised as present/absent<br><br>Age was categorised (<65, 65-75, 76-85, >85)

		disease excluding bleeding, lymphoma, solid tumor without metastasis, rheumatoid arthritis/collagen vascular disease, coagulopathy, obesity (not define; reference category not reported), weight loss, fluid and electrolyte disorders, deficiency anaemias, alcohol abuse, psychoses, depression, hypertension (uncomplicated and complicated combined), drug abuse, metastatic cancer, chronic blood loss anaemia, hypothyroidism	Comorbidities:<br>CHF = 776 (3.5%)<br>Valvular disease = 1,395 (6.3%)<br>Pulmonary circulation disorder = 205 (0.9%)<br>Pulmonary vascular disorder = 510 (2.3%)<br>Paralysis = 47 (0.2%)<br>Other neurological disorder = 610 (2.8%)<br>Chronic pulmonary disease = 3,568 (16.2%)<br>Diabetes = 4,560 (20.7%)<br>Hypothyroidism = 2,872 (13.0%)<br>Renal failure = 716 (3.2%)<br>Liver disease = 205 (0.9%)<br>Peptic ulcer disease excluding bleeding = 120 (0.5%)<br>Lymphoma = 41 (0.2%)<br>Metastatic cancer = 10 (0.0%)<br>Solid tumor without metastasis = 694 (3.1%)<br>Rheumatoid arthritis/collagen vascular disease = 1,130 (5.1%)<br>Coagulopathy = 279 (1.3%)<br>Obesity = 3,388 (15.3%)<br>Weight loss = 21 (0.1%)<br>Fluid and electrolyte disorders = 579 (2.6%)<br>Chronic blood loss anaemia = 73 (0.3%)<br>Deficiency anaemias = 1,628 (7.4%)<br>Alcohol abuse = 160 (0.7%)<br>Drug abuse = 93 (0.4%)<br>Psychoses = 316 (1.4%)<br>Depression = 2,057 (9.3%)<br>Hypertension (uncomplicated and complicated combined) = 14,185 (64.3%)	
			TKA-specific:<br>Mean age (SD) = 67.6 (11.0)<br>Sex: male = 965 (39.4%); female = 1,484 (60.6%)<br>Race/ethnicity: White = 1,814 (74.1%); Black = 265 (10.8%); Hispanic = 139 (5.7%); Asian = 20 (0.8%); Other = 129 (5.3%); Missing = 82 (3.3%)<br>Insurance status: Medicare = 1,472 (60.1%); Medicaid = 147 (6.0%); Private = 685 (28.0%); Worker's Compensation = 91 (3.7%); Other = 54 (2.2%)	

			Surgical indication: osteoarthritis = 2,282 (93.2%); inflammatory arthritis = 146 (6.0%); osteonecrosis = 21 (0.9%)<br>Bilateral TKA = 144 (5.9%)<br>In-hospital complications: medical = 226 (9.2%); surgical = 164 (6.7%)<br><br>Comorbidities:<br>CHF = 94 (3.8%)<br>Valvular disease = 131 (5.3%)<br>Pulmonary circulation disorder = 37 (1.5%)<br>Pulmonary vascular disorder = 58 (2.4%)<br>Paralysis = 7 (0.3%)<br>Other neurological disorder = 73 (3.0%)<br>Chronic pulmonary disease = 418 (17.1%)<br>Diabetes = 558 (22.8%)<br>Hypothyroidism = 333 (13.6%)<br>Renal failure = 108 (4.4%)<br>Liver disease = 23 (0.9%)<br>Peptic ulcer disease excluding bleeding = 9 (0.4%)<br>Lymphoma = 5 (0.2%)<br>Metastatic cancer = 0 (0.0%)<br>Solid tumor without metastasis = 50 (2.0%)<br>Rheumatoid arthritis/collagen vascular disease = 137 (5.6%)<br>Coagulopathy = 42 (1.7%)<br>Obesity = 492 (20.1%)<br>Weight loss = 5 (0.2%)<br>Fluid and electrolyte disorders = 70 (2.9%)<br>Chronic blood loss anaemia = 9 (0.4%)<br>Deficiency anaemias = 181 (7.4%)<br>Alcohol abuse = 20 (0.8%)<br>Drug abuse = 17 (0.7%)<br>Psychoses = 36 (1.5%)<br>Depression = 303 (12.4%)<br>Hypertension (uncomplicated and complicated combined) = 1,633 (66.7%)	
			Expanded TKA-specific:<br>Mean age (SD) = 67.4 (11.1)<br>Sex: male = 2,748 (40.5%); female = 4,036 (59.5%)	

			Race/ethnicity: White = 4,965 (73.2%); Black = 736 (10.8%); Hispanic = 456 (6.7%); Asian = 72 (1.1%); Other = 348 (5.1%); Missing = 207 (3.1%)<br>Insurance status: Medicare = 4,022 (59.3%); Medicaid = 429 (6.3%); Private = 1,920 (28.3%); Worker's Compensation = 272 (4.0%); Other = 141 (2.1%)<br>Surgical indication: osteoarthritis = 6,364 (93.8%); inflammatory arthritis = 370 (5.5%); osteonecrosis = 50 (0.7%)<br>Bilateral TKA = 269 (4.0%)<br>In-hospital complications: medical = 470 (6.9%); surgical = 336 (5.0%)<br><br>Comorbidities:<br>CHF = 259 (3.8%)<br>Valvular disease = 331 (4.9%)<br>Pulmonary circulation disorder = 69 (1.0%)<br>Pulmonary vascular disorder = 163 (2.4%)<br>Paralysis = 15 (0.2%)<br>Other neurological disorder = 218 (3.2%)<br>Chronic pulmonary disease = 1,257 (18.5%)<br>Diabetes = 1,505 (22.2%)<br>Hypothyroidism = 893 (13.2%)<br>Renal failure = 258 (3.8%)<br>Liver disease = 88 (1.3%)<br>Peptic ulcer disease excluding bleeding = 28 (0.4%)<br>Lymphoma = 13 (0.2%)<br>Metastatic cancer = 0 (0.0%)<br>Solid tumor without metastasis = 126 (1.9%)<br>Rheumatoid arthritis/collagen vascular disease = 353 (5.2%)<br>Coagulopathy = 110 (1.6%)<br>Obesity = 1,251 (18.4%)<br>Weight loss = 10 (0.1%)<br>Fluid and electrolyte disorders = 187 (2.8%)<br>Chronic blood loss anaemia = 23 (0.3%)<br>Deficiency anaemias = 499 (7.4%)<br>Alcohol abuse = 63 (0.9%)<br>Drug abuse = 50 (0.7%)<br>Psychoses = 119 (1.8%)<br>Depression = 788 (11.6%)	

			Hypertension (uncomplicated and complicated combined) = 4,442 (65.5%)	
George 2018	As per NSQIP documentation, referenced and (briefly) described in the paper	Reference category for all of the following = normal weight (BMI >18.5 to <25):<br>Overweight (BMI >25 to <30)<br>Obese (BMI >30 to <40)<br>Morbidly obese (BMI >40)	Calculated from given % readmission rates and rounded down:<br>Normal weight = 530 (3.54%)<br>Overweight = 1366 (3.32%)<br>Obese = 2316 (3.23%)<br>Morbidly obese = 976 (4.23%)	BMI was categorised as per the 'Predictor variables' column of this table<br><br>Comorbidities were dichotomised as present/absent<br><br>Weight loss was dichotomised as present/absent<br><br>Functional status was dichotomised as independent/non-independent
Gwam 2020	As per NSQIP protocols, described and referenced in the paper	Dialysis dependence (defined as dialysis use within two weeks prior to the procedure)	Not reported	Weight loss was dichotomised present or absent based on at least 10% body weight lost<br><br>Comorbidities were dichotomised as present or absent
Hanly 2017	All operations performed at the centre are recorded prospectively using SOCRATES (Standardised Orthopaedic Clinical Research and Treatment Evaluation Software, V6.9, Ortholink Pty, Sydney, Australia). Age, sex,	Morbidly obese (normal weight)	Morbidly obese = 17 (14.5%)<br>Normal weight = 8 (8.5%)	BMI was categorised (morbidly obese vs normal weight, according to WHO guidelines)

	comorbidities, and BMI were routinely collected.			
Hart 2016	As per NSQIP protocol described and referenced in the paper	Main predictor of interest = bilateral vs unilateral TKA<br><br>Others:<br>Age (continuous)<br>Sex (female)<br>Smoker (non-smoker)<br>Hypertension<br>COPD<br>Steroid use<br>Blood urea nitrogen<br>ASA classification (1)	Only available for main predictor of interest:<br>Bilateral group = 64 (3.6%)<br>Unilateral group = 120 (3.5%)	Comorbidities were dichotomised as present/absent
Jauregui 2015	Not reported	Age<br>Gender<br>BMI<br>Comorbidities (e.g. COPD, percutaneous coronary intervention, cardiac surgery, hypertension requiring medication, bleeding disorders)<br>ASA grade	Not reported	No
Jorgensen 2013	Patients completed a pre-operative questionnaire regarding smoking (smoking daily: yes/no), alcohol use (daily use >2 units of alcohol: yes/no), functional level (daily use of mobility aids pre-operatively), housing condition (living with spouse/partner, living alone or in institution (nursing home, sheltered care facility, etc)), pharmacological treatment for cardiopulmonary disease (atrial flutter/fibrillation, angina pectoris, COPD, asthma, etc)	Smoking<br>Alcohol use<br><br>Others:<br>Cardiopulmonary disease<br>Use of walking aids<br>Housing condition<br>Male sex	Obtained upon request from corresponding author:<br>Smoking = 13 (6.5%)<br>Cardiopulmonary disease = 27 (10.1%)<br>Use of walking aids = 39 (10.9%)<br>Alcohol use = 6 (6.1%)<br>Living alone = 39 (7.6%)<br>Living with others = 55 (5.7%)<br>Living in institution = 0<br>Male sex = 43 (7.2%)<br>Female sex = 43 (4.9%)	Smoking, alcohol use, cardiopulmonary disease, and functional status were dichotomised
Jorgensen 2017	Prior to surgical procedure, all patients complete a questionnaire on comorbidity and other demographic information, which is then merged with the Danish	Age (66-70)<br>BMI (18.5-25)<br>Smoking<br>Alcohol use<br>Use of walking aids	Age:<br><50 = 2<br>50-60 = 21<br>61-65 = 31<br>66-70 = 27	Age (<50; 50-60; 61-65; 66-70; 71-75; 76-80; 81-85; >80)

	National Database on Reimbursed Prescriptions for additional information on dispensed drugs before and after surgery	Living alone (living with spouse or relatives)<br>Living in an institution (living with spouse or relatives)<br>Anaemia<br>NIDDM<br>Pharmacologically treated pulmonary disease<br>Pharmacologically treated Cardiac disease<br>Psychiatric disorder<br>Anticoagulant therapy	71-75 = 37<br>76-80 = 43<br>81-85 = 18<br>>85 = 12<br><br>BMI:<br><18.5 = 0<br>18.5-25 = 50<br>25.1-29.9 = 65<br>30-39.9 = 24<br>≥40 = 6<br><br>Living alone = 74<br>Living in institution = 6<br><br>NIDDM = 23<br><br>Cardiac disease = 45<br><br>Pulmonary disease = 27<br><br>Alcohol use = 17<br><br>Smoking = 23<br><br>Psychiatric disorder = 41<br><br>Anaemia = 32<br><br>Anticoagulant use = 16<br><br>Use of walking aid = 60	BMI (<18.5; 18.5-25; 25.1-29.9; 30-39.9; ≥40)<br><br>Comorbidities were dichotomised as present/absent
Keeney 2015	Extracted from hospital admission database, but unclear method of recording socioeconomic status	Socioeconomic status and minority status	Not reported	Socioeconomic status was categorised, with socioeconomic disadvantage being define as: Medicaid insurance, or Medicare insurance and age <65 years<br><br>Minority status was dichotomised, with

				minority status being defined as all categories other than Caucasian (non-Hispanic)
Kester 2016	As per NSQIP protocol, trained and certified surgical reviewers at each surgical site abstract data from medical records and by contacting patients directly	Post-traumatic TKA as a predictor of readmissions	10 readmissions in the post-traumatic group	Age (dichotomised at 50)<br><br>BMI (dichotomised at 40)
Kheir 2014	Not reported	Age<br>Sex<br>Race<br>BMI<br>Medical Severity Diagnosis-Related Group (MS-DRG)<br>Whether the TKA was the second episode of a staged bilateral procedure	Average MS-DRG in readmitted group = 2.57<br><br>Age of readmitted group = 63.9 (12.6) years<br><br>BMI of readmitted group = 33.4 (8.2) kg/m^2<br><br>Number of readmissions:<br>Sex:<br>Female = 112<br>Male = 53<br><br>Race:<br>White = 100<br>Black = 55<br>Native American = 0<br>Asian = 2<br>Other = 4<br>Unknown = 4<br><br>Staged bilateral procedures = 5	Age (≤55, 56-65, 66-75, ≥76)<br><br>BMI (<25, 25-30, 30 to <35, ≥35)
Kim 2019	ICD-9 codes for medical diagnoses, National Drug Codes for medication dispensing	Preoperative opioid use (continuous use vs intermittent use vs opioid-naïve patients)	Continuous opioid users = 1672<br><br>Intermittent opioid users = 9027<br><br>Opioid-naïve patients = 6087	Individual comorbidities were dichotomised as present/absent
Kuo 2017	Not reported	CKD (non-CKD)	CKD group = 12<br><br>Non-CKD group (calculated from % readmission rate and rounded down) = 3	eGFR cut-off for CKD definition was 60 mL/min/1.73m^2 (CKD = eGFR <60)<br><br>Individual comorbidities were dichotomised as present/absent

Kurtz 2016	1-year look-back period before index TKA<br><br>Unclear method of measurement	Lymphoma<br>CCI<br>Secondary tumour<br>Drug/alcohol abuse<br>Obesity<br>Transfusion<br>Diabetic<br>Depression<br>Age<br>Anaemia<br>Pulmonary disease<br>Renal failure<br>Sex<br>Heart disease	Number of events per predictor variable were only available for the following variables.<br><br>Age: 65-69 = 11,747; 70-74 = 12,443; 75-79 = 11,553; 80-84 = 7957; 85+ = 3568<br><br>CCI: 00 = 20,282; 1-2 = 19,891; 3-4 = 5475; 5+ = 1621<br><br>Sex: female = 27,431; male = 19,837<br><br>Race: Black = 2987; Other/Unknown = 1734; White = 42,547	Categorical age was presented for baseline demographics, but appears to have been included in the multivariate logistic regression as a continuous variable<br><br>Comorbidities were dichotomised as present or absent
Lehtonen 2018	According to the NSQIP User Guide (referenced in the article)	Univariate (reference category not applicably):<br>Age, sex, race, BMI category, recent weight loss, smoking, diabetes, dialysis use, hypertension, CHF, dyspnoea, COPD, bleeding disorder, open/infected wound, sepsis, SIRS, septic shock, corticosteroid use, WBC count ($x10^3$/L), haematocrit (%), platelets ($x10^3$/L), creatinine (mg/dL), serum albumin (g/dL), INR, ASA class<br><br>Multivariate:<br>Age (continuous – per year)<br>Sex (male)<br>Race (white)<br>BMI category (overweight)<br>≥1 Comorbidity (absence of comorbidities)<br>ASA class (2)<br><br>Medical complications:<br>Pneumonia<br>Urinary tract infection<br>Stroke or CVA<br>Acute renal failure<br>Cardiac arrest<br>Myocardial infarction	Age = 66.53 years old (non-readmitted group) vs 68.49 years old (readmitted group)<br>Sex: male = 2039; female = 2629<br>Race: White = 3691; Black = 440; Asian = 63; American Indian = 15; Native Hawaiian = 9; Unreported = 450<br>BMI category: <18.5 = 10; 18.5-25 = 466; 25-30 = 1211; 30-35 = 1246; 35-40 = 824; ≥40 = 894<br>Recent weight loss = 6<br>Smoking = 482<br>Insulin-dependent diabetes = 345<br>Non-insulin-dependent diabetes = 706<br>Dialysis use = 22<br>Hypertension = 3460<br>CHF = 44<br>Dyspnoea = 456<br>COPD = 373<br>Bleeding disorder = 241<br>Open/infected wound = 35<br>Sepsis = 0<br>Septic shock = 0<br>SIRS = 15<br>Corticosteroid use = 260<br>WBC count = 7.05 (2.75-11.35) non-readmitted group vs 7.26 (1.64-12.88) readmitted group<br>Haematocrit = 40.82 (32.74-48.9) non-readmitted group vs 40.29 (31.51-49.07) readmitted group<br>Platelets = 244.12 (111.52-376.72) non-readmitted group vs 239.44 (97.78-381.1) readmitted group	BMI (<18.5; 18.5-25; 25-30; 30-35; 35-40; ≥40)<br><br>Comorbidities were dichotomised as present or absent for univariate analysis<br><br>For multivariate analysis, a dummy variable was created for presence or absence of at least one comorbidity

		Surgical complications:<br>Superficial surgical site infection<br>Pulmonary embolism<br>Deep venous thrombosis	Creatinine = 0.91 (0.11-1.71) non-readmitted group vs 1.01 (9.21-1.81) readmitted group<br>Serum albumin = 4.10 (3.34-4.86) non-readmitted group vs 4.02 (3.2-4.84) readmitted group<br>INR = 1.02 (0.52-1.52) non-readmitted group vs 1.06 (0.56-1.56) readmitted<br>ASA class 1 = 66<br>ASA class 2 = 1683<br>ASA class 3 = 2745<br>ASA class 4 = 169<br>ASA class 5 = 0<br>ASA class not assigned = 5	
Liao 2016	ICD-9 codes<br><br>For comorbidities, each patient was traced back from 2 years to the<br>index date, which included cardiovascular disease (CVD), CVAs,<br>chronic kidney disease (CKD), diabetes mellitus (DM), and rheumatoid arthritis (RA)	The primary predictor of interest was COPD.<br><br>Other predictors analysed:<br>Age (dichotomised at 60 – reference category not applicable)<br>Sex<br>Hypertension<br>Cardiovascular disease<br>Cerebrovascular accident<br>Chronic kidney disease<br>Diabetes mellitus	Calculated from % values and rounded down:<br>COPD: yes = 25 (7.0%); no = 122 (4.0%)<br>Age: <60 = N not reported (3.8%); ≥60 = N not reported (4.4%)<br>Sex: male = 53 (6.1%); female = 94 (3.7%)<br>Hypertension: yes = 92 (4.1%); no = 55 (4.7%)<br>CVD: yes = 20 (7.3%); no = 126 (4%)<br>CVA: yes = 26 (5.5%); no = 120 (4.1%)<br>CKD: yes = 17 (6.4%); no = 123 (3.9%)<br>DM: yes = 53 (6%); no = 93 (3.7%)	Comorbidities were dichotomised as present or absent<br><br>Age was dichotomised at 60 years
Lovecchio 2014	As per NSQIP documentation referenced in the paper	NIDDM (no diabetes)<br>IDDM (no diabetes)	NIDDM = 177 (4.6%)<br>IDDM = 83 (7.2%)	Nil
Miric 2014	Described previously (reference 10 of the paper)	Age category – the primary predictor of interest was age >90 compared to age younger patients	Age 90+ = 7<br>Age 80-89 = 203<br>Age <80 = 836	Age
Mudumbai 2019	The following methods were utilised: administrative data review for inpatient and outpatient instances; ICD-9 diagnosis codes; electronic medical records<br><br>Measured within the year prior to TKA for 'preoperative chronic pain' and 'illness and care severity' and comorbidities	Reference categories not applicable.<br>Predictors:<br>Age<br>Sex<br>Surgery type<br>Post-discharge opioid status as of 30 d<br>Preoperative outpatient opioids – status for the 180 d preoperatively<br>Preoperative adjunctive pharmacotherapy for the 180 d preoperatively<br>DRG weight	Age: ≤54 = 55; 55-65 = 266; ≥66 = 210<br>Sex: female = 35; male = 496<br>Surgery type: primary = 469; revision = 62<br>Post-discharge opioid status as of 30 d: tramadol only = 16; short-acting only = 311; any long-acting = 18; no opioids = 186<br>Preoperative outpatient opioids – status for the 180 d preoperatively: tramadol only = 55; short-acting acute = 145; short-acting chronic = 112; any long-acting = 21; no opioids = 198<br>Preoperative adjunctive pharmacotherapy for the 180 d preoperatively: analgesics = 255; SNRIs = 65; anticonvulsants = 119; TCAs = 38; sedatives = 113	Age (≤54, 55-65, ≥66)<br><br>Comorbidities were dichotomised as presence or absence<br><br>Pharmaceutical use was dichotomised as presence or absence within specified time frames

		CCI<br>Diagnoses in the 365 d before admission<br>Had chronic pain in the 365 d before admission<br>Had a prior inpatient surgery in the 180 d before admission<br>Had >30 outpatient visits in the 365 d before admission<br>Urban/rural status<br>Homeless<br>Currently married<br>Race	DRG weight: ≤2 = 9; 2-3 = 479; 3+ = 43<br>CCI: 0 = 289; 1 = 142; 2+ = 100<br>Diagnoses in the 365 d before admission: substance use disorder (excludes nicotine) = 50; nicotine = 56; bipolar disorder = 17; major depression = 39; PTSD = 100; generalised anxiety disorder = 8<br>Had chronic pain in the 365 d before admission = 505<br>Had a prior inpatient surgery in the 180 d before admission = 10<br>Had >30 outpatient visits in the 365 d before admission = 230<br>Urban/rural status: highly rural = 7; rural = 165; urban = 359<br>Homeless = 32<br>Currently married = 306<br>Race: Caucasian/white = 407; American Indian = 7; Asian = 2; African American/black = 110	
Nowak and Schemitsch 2019	As per NSQIP documentation referenced in the paper	Bleeding disorder (reference = no bleeding disorder)<br>CHF (reference = no CHF)<br>COPD (reference = no CHF)<br>Hypertension (reference = no hypertension)	Not reported	Nil
Ottesen 2018	Dialysis dependence was defined in NSQIP as any patient who received peritoneal dialysis, haemodialysis, haemofiltration, haemodiafiltration, or ultrafiltration within two weeks before their surgical procedure	Dialysis dependence (non-dialysis dependence). Defined as dialysis within two weeks prior to TKA	22 readmissions in dialysis-dependent group<br>4340 readmissions in non-dialysis-dependent group	Age (≤40, 41-50, 51-60, 61-70, 71-80, 81-90, >90)<br><br>BMI (<25, 25-30, 30-35, 35-40, ≥40)

Patel 2020	Not reported	Sex	Not reported	Comorbidities were dichotomised as present or absent<br><br>Procedure length was dichotomised at 100 minutes
Patterson 2018	As per NSQIP data collection practices, the authors were able to obtain from the database information regarding type of dialysis (peritoneal dialysis, haemodialysis, haemofiltration, or ultrafiltration) within two weeks prior to primary TKA procedure, and indication (acute or chronic renal failure)	Dialysis dependence (non-dialysis-dependence). Defined as dialysis within two weeks prior to TKA	17 readmissions in dialysis-dependent group<br><br>3956 readmissions in non-dialysis-dependent group	BMI (<18.5, 18.5-25, 25-30, 30-40, >40)<br><br>Comorbidities were dichotomised as present or absent
Peskun 2012	All patients were preoperatively assessed by a general internist and/or anaesthetist and medical co-morbidities, including: previous myocardial infarction(MI)/coronary artery disease(CAD), diabetes mellitus (DM), chronic obstructive pulmonary disease(COPD), and hypertension (HTN), were noted.	Age (no reference - continuous variable)<br>Gender (reference not stated)<br>Hypertension (reference = no hypertension)<br>T2DM (reference = no diabetes)<br>MI/CAD (reference = no MI/CAD)<br>COPD (reference = no COPD)	Not reported	Nil
Pugely 2013	Refer to the final paragraph of the “Data” subsection of the Methods section of the paper – page 1500 – for a description of the NSQIP data collection process	Age (56-65)<br>Sex (female)<br>Race<br>BMI<br>ASA class (1 or 2)<br>Functional status independent<br>Functional status totally or partially dependent	Calculated from % readmission rates and rounded down:<br>Age <45 = 16; Age 46-55 = 57; Age 56-65 = 133; Age 66-75 = 176; Age 76-85 = 138; Age >85 = 23<br>Male sex = 239; Female sex = 304<br>Black race = 31; white race = 449; other = 62<br>BMI <35 = 375; BMI >35 = 168	Age was categorised (<45, 46-55, 56-65, 66-75, 76-85, >85)<br><br>BMI was dichotomised at 35kg/m^2

		Comorbidities (defined dichotomously as present or absent, with absent as the reference category for variables included in the multivariate logistic regression model):<br>Current alcohol abuse<br>Current smoker<br>Recent weight loss<br>Dyspnoea<br>COPD<br>CHF<br>Hypertension<br>Diabetes<br>PVD<br>Disseminated cancer<br>Steroid use<br>Bleeding disorder<br>Dialysis<br>Chemotherapy within 30 days<br>Radiation therapy within 90 days<br>Prior operation within 30 days<br>Mean pre-op sodium<br>Mean pre-op BUN<br>Mean pre-op albumin<br>Mean pre-op WBC<br>Mean pre-op haematocrit<br>Mean pre-op platelets<br>Mean pre-op INR<br>Mean pre-op creatinine	Current alcohol abuse = 10; current smoker = 53; recent weight loss = 2; dyspnoea = 51; COPD = 29; CHF = 4; hypertension = 401; diabetes = 118; PVD = 7; disseminated cancer = 2; steroid use = 16; bleeding disorder = 35; dialysis = 2; chemotherapy within 30 days = 5; radiation therapy within 90 days = 0; prior operation within 30 days = 0<br><br>ASA: 1 or 2 = 215; 3 = 316; 4 = 12<br><br>Functional status: independent = 529; totally or partially dependent = 14<br><br>Mean values in readmitted patients:<br>Pre-op sodium = 139.3<br>Pre-op BUN = 20.18<br>Pre-op albumin = 4.04<br>Pre-op WBC = 7.18<br>Pre-op haematocrit = 40.42<br>Pre-op platelets = 238.9<br>Pre-op INR = 1.06<br>Pre-op creatinine = 1.02	All comorbidities were dichotomised as present or absent
Ramos 2014	Comorbidities were identified using MS-DRG codes in the institution's administrative database. Otherwise the method of measurement is unclear	Age (continuous)<br>Sex (male)<br>Comorbidity (no comorbidity – MS-DRG codes were used to derive a surrogate marker for comorbidities according to an unclear methodology)	Not reported	No
Ricciardi 2017	Patient factors were retrieved from electronic health records. Perioperative factors at index procedure were retrieved from the	Patient characteristics:<br>Sex (reference not applicable)<br>Race (reference not applicable)<br>BMI (reference not applicable)	Only available in the TKA-only cohort for ASA class and comorbidities.<br>ASA class: 1-2 = 19; 3-4 = 4	No

	hospital's electronic medical record.	Perioperative factors:<br>ASA class (reference not applicable)<br><br>Comorbidities:<br>List of Elixhauser comorbid conditions<br>CHF<br>Valvular disease<br>PVD<br>Neurological disorders<br>Long-term pulmonary disease<br>Diabetes (with and without long-term complications)<br>Hypothyroidism<br>Renal failure<br>Liver disease<br>Coagulopathy<br>Obesity<br>Fluid and electrolyte disorders<br>Anaemia<br>Depression<br>Hypertension	Comorbidities (from the full list given in the 'Predictor variables (reference)' column of this table, which were identified a priori for the combined THA and TKA cohort prior to study commencement, only the following comorbidities were present at index procedure for the TKA cohort (see Table 8 of the study)):<br>Valvular disease = 4<br>PVD = 0<br>Other neurological disorders = 1<br>Long-term pulmonary disease = 2<br>Diabetes without long-term complications = 4<br>Liver disease = 1<br>Obesity = 4<br>Fluid and electrolyte disorders = 2<br>Deficiency anaemias = 3<br>Depression = 8<br>Hypertension = 13	
Robinson 2017	Direct quote: "Demographic data, comorbid conditions, Current Procedural Terminology (CPT) codes, hospital length of stay (LOS), and 30-day postoperative adverse events, including unplanned<br>reoperation or readmission are abstracted from medical records, operative reports, and patient interviews."	Sex	Males = 1313<br>Females = 1684	Obesity classification defined by World Health Organization was used: non-obese = 18.5-29.9kg/m^2; obesity class I = 30.0-34.9; obesity class II = 35.0-39.9; obesity class III = ≥40
Ross 2020	Not reported	Age (per 10 years)<br>Female (reference = male)<br>Income quintile (reference = 5: highest)<br>Visit with GP between surgery and readmission/ED visit (reference = no visit)<br>CCI (reference = 0)	Mean age of readmitted patients = 70.38 ± 10.16<br>Female = 4030<br>Income quintile: 1 = 1550; 2 = 1498; 3 = 1413; 4 = 1456; 5 = 1421<br>Visit to GP during follow-up = 3628<br>CCI: 0 = 4960; 1 = 1456; 2 = 578; 3 = 344<br>Revision index surgery = N/A (not reported)	Age was categorised into 10-year intervals with unclear cut-points<br><br>Income was analysed in quintiles

		Revision index surgery (reference = primary)		
Roth 2019	Authors refer the reader to NSQIP documentation for specific details on the data collection process used by NSQIP clinical reviewers	(reference = normal weight)<br>Overweight<br>Obese<br>Morbidly obese	Calculated from % readmission rate and rounded down:<br>Normal weight = 40<br>Overweight = 123<br>Obese = 250<br>Morbidly obese = 134	BMI was analysed as continuous variable, but initial comparisons were made between patients in the pre-defined BMI categories (normal weight = 18.5-25, overweight = 25-30, obese = 30-40, morbidly obese = 40+)
Rudasill 2019	NSQIP database routinely collects demographic, laboratory, and perioperative data. Patients who had an INR recorded within one day prior to TKA were included for analysis	INR levels (reference <= 1)	851:<br>403 (INR <= 1)<br>364 (INR >1-1.25)<br>67 (INR >1.25-1.5)<br>17 (INR >1.5)	INR levels:<br>INR <= 1<br>INR >1-1.25<br>INR >1.25-1.5<br>INR >1.
Runner 2017	Authors refer the reader to NSQIP documentation for specific details on the data collection process used by NSQIP clinical reviewers	Main predictor of interest = Modified Frailty Index (MFI)<br>Other predictor variables:<br>ASA class (1)<br>Age ≥60 (unclear reference category considering only patients aged ≥60 were included in the study)<br>Wound class (class I)<br>Male sex<br>BMI >40<br>Any occurrence (of adverse events other than mortality, during hospital stay)	Unable to calculate	Age was dichotomised at 60 years<br><br>BMI was dichotomised at 40kg/m^2
Saucedo 2014	Extracted from an electronic database (Enterprise Data Warehouse, Chicago, IL)	Age (50-79)<br>BMI (28.5-24.9)<br>Ethnicity – African-American, Caucasian, other (reference unavailable)<br>Sex (reference unavailable)<br>Payer group – preferred provider organisation (PPO), health maintenance organisation (HMO), Medicare, self-pay, other (reference unavailable)	Not reported	Age was categorised (20-49, 50-79, ≥80)<br><br>BMI was categorised (>30 vs 18.5-24.9. Other categories may have been created but only significant risk factors on multivariate analysis were reported so any other potential

		Comorbidities:<br>CHF<br>Coronary artery disease (CAD)<br>Diabetes mellitus<br>COPD<br>History of pulmonary embolus		categories are not clearly stated)
Schaeffer 2015	Direct quote: “ASA status was obtained by the anesthesia summary and was determined by the anesthesia team, led by the attending<br>anesthesia provider	Only ASA class was of interest	ASA 1 = 0 readmissions (2 discharges)<br>ASA 2 = 3 readmissions (283 discharges)<br>ASA 3 = 8 readmissions (290 discharges)<br>ASA 4 = 0 readmissions (4 discharges)<br>ASA not available = 1 readmission (83 discharges)	N/A
Schairer 2014	Not reported	30-day readmission data only available for TKA group: primary, revision, revision for infection	TKA:<br>Primary = 64 (6.2% of readmitted patients)<br>Revision (non-infective) = 34 (13.0% of readmitted patients)<br>Revision (for infective causes) = 20 (17.0% of readmitted patients)	N/A
Singh 2013	As per PHC4 documentation, described and referenced in the paper	Sex	1061 (635 women, 426 men)	Hospital procedure volume was dichotomised into two levels (1-100 and >100 TKAs performed) based on a priori consideration of the potential impact of hospital volume balanced with a consideration of the relatively small number of very high volume facilities.

				Age was categorised (18-64, ≥65)
Siracuse 2017	Demographic and clinicopathologic variables were abstracted from SID records	Age (51-60)<br>Sex (female)<br>Race/ethnicity (Caucasian)<br>Income quartile (1st)<br>Primary payer (private insurance)<br>Anaemia<br>Chronic obstructive pulmonary disease<br>Coagulopathy<br>Congestive heart failure Diabetes<br>Fluid and electrolyte disorder<br>Hypertension<br>Liver disease<br>Obesity<br>Renal failure<br>Rheumatoid arthritis	Not reported	Income was categorised into quartiles<br><br>Age was categorised (41-50, 51-60, 61-70, 71-80, 81-90)
Sloan 2020	Not reported	BMI categories (as per WHO system):<br>Underweight (<18.5)<br>Normal weight (18.5 to <25)<br>Overweight (25 to <30)<br>Obese Class I (30 to <35)<br>Obese Class II (35 to <40)<br>Obese Class III (>40)<br><br>Albumin level:<br>Continuous variable<br>Dichotomised (hypoalbuminaemia vs normal albumin)	BMI categories:<br>Underweight = 8 (4.06%)<br>Normal weight = 337 (3.59%)<br>Overweight = 918 (3.38%)<br>Obese I = 960 (3.33%)<br>Obese II = 615 (3.11%)<br>Obese III = 670 (4.15%)<br><br>Albumin level:<br>Normal albumin = 3254 (3.34%)<br>Hypoalbuminaemia (<35g/L as per Table II) = 254 (6.10%)	Comorbidities were dichotomised as present or absent<br><br>Albumin level was dichotomised into two groups (<35g/L (hypoalbuminaemia group) and ≥35g/L)
Sodhi and Anis et al 2019	As per NSQIP documentation referenced in the paper	Elective procedure (reference = non-elective)	4514 (2.3%) readmissions in elective group vs 64 (2.8%) in non-elective group	N/A
Sodhi and Mont et al 2019	Patient-reported baseline health status measures were collected from the point of care at preoperative check-in and, in one hospital, from the preoperative visit with the orthopaedic surgeon.	Elements of the Hospital Consumer Assessment of Healthcare Providers and Systems (HCAHPS) patient experience of care survey:<br>Communication with physicians and nurses<br>Responsiveness of hospital staff	Readmissions per number of responders for each component, among the readmitted patients, are reported below.<br>Communication with nurses:<br>Nurses “always” treated you with courtesy and respect = 11/12<br>Nurses “always” listened carefully to you = 10/12	N/A

	Other baseline variables (demographics, comorbidities): Unclear<br><br>Timing of measurement was specified:<br>For patient-reported baseline health status: Preoperative check-in and, in one hospital<br><br>For other baseline variables (demographics, comorbidities): Unclear	Pain management<br>Cleanliness<br>Quietness of hospital environment<br>Discharge information	Nurses “always” explained things in a way you could understand = 9/12<br><br>Communication with doctors:<br>Doctors “always” treated you with courtesy and respect = 9/12<br>Doctors “always” listened carefully to you = 8/12<br>Doctors “always” explained things in a way you could understand = 7/12<br><br>Cleanliness and quietness of the hospital environment:<br>Your room and bathroom were “always” kept clean = 9/12<br>The area around your room was “always” quiet at night = 5/12<br><br>Responsiveness of hospital staff:<br>You “always” got help in getting to the bathroom or using a bedpan as soon as you wanted it = 5/8<br>After you pressed the call button, you “always” got help as soon as you wanted it = 6/11<br><br>Pain management:<br>Your pain was “always” well controlled = 6/10<br>The hospital staff “always” did everything they could to help you with your pain = 8/10<br><br>Communication about medicines:<br>Before giving you any new medicine, hospital staff “always” described possible side effects in a way you could understand = 2/7<br>Before giving you any new medicine, hospital staff “always” told you what the medicine was for = 6/8<br><br>Discharge information:<br>Hospital staff talked with you about whether you would have the help you needed when you left the hospital = 9/9<br>You got information in writing about what symptoms or health problems to look out for after you left the hospital = 7/8	

			Staff took your preferences and those of your family into account in deciding what your health care needs would be when you left the hospital = 4/12<br><br>Transition of care:<br>You had a good understanding of the things you were responsible for in managing your health = 4/12<br>You clearly understood the purpose for taking each of your medications = 7/10<br><br>Overall rating of a hospital:<br>Rated this hospital as a “9 or 10” out of 10 overall = 8/12<br>Would “definitely” recommend this hospital to friends and family = 7/12	
Suleiman 2015	According to the NSQIP User Guide (referenced in the article)	SB TKA (unilateral TKA)	9 readmissions in SB TKA group	BMI was categorised in a manner that resembles the WHO’s system
Sutton 2016	As per NSQIP protocols, referenced in the paper	Age (per year)<br>Sex (female)<br>BMI continuous (per kg/m^2)<br>Race<br>Smoking status (non-smoker)<br>Hypertension<br>Diabetes<br>COPD<br>CHF & Dyspnoea<br>History of corticosteroid use<br>Preop lab values: creatinine (mg/dL), BUN (mg/dL), hematocrit (%), platelets (x 10^9/L)<br>ASA classification (1)	Not reported	N/A
Tang 2019	Not reported	Age (continuous)<br>BMI (continuous)<br>Sex (male)<br>Bilateral procedure (unilateral)<br>Revision procedure – no cases<br>Smoking<br>Diabetes mellitus<br>Ischaemic heart disease (IHD)	Age = 66.3 (7.7) readmitted vs 66.8 (8.9) non-readmitted<br><br>Race:<br>Chinese = 34<br>Indian = 5<br>Malay = 4<br>Others = 1	Comorbidities were dichotomised as present or absent<br><br>Age was categorised (<60, 60-64, 64-69, ≥70)

		Previous CVA or TIA<br>CHF<br>Race (Chinese)<br>ASA-PS (1)	BMI:<br><25 = 14<br>25-29.9 = 15<br>30-34.9 = 9<br>≥35 = 6<br><br>Male sex = 16<br>Female sex = 28<br><br>Diabetes mellitus = 8<br>Anaemia: mild = 17; moderate/severe = 3<br>Smoking = 7<br>Previous CVA or TIA = 4<br>IHD = 3<br>CHF = 1<br>ASA-PS (physical score): 1 = 3; 2 = 37; 3 = 4	BMI was categorised (<25, 25-29.9, 30-34.9, ≥35)<br><br>Anaemia was categorised (none, mild, moderate/severe)
Tay 2017	Direct quote: "Medical records were extensively reviewed to ensure accurate capture of information, especially with regards to complications and comorbidities. All data were collected by independent observers not participating in the care of the patients"	Age (OG vs YG)<br>CCI	YG = 3<br>OG = 10<br>CCI (unable to calculate accurately, therefore readmission rate is presented here instead of number of events per category): 0 = 2.9%; 1 = 4.1%; 2 = 2.2%; ≥3 = 8.3%	Age was dichotomised at 80 years
Urish 2018	ICD-9 codes	Age (45-54): 55-64; 65-74; 75-84; 85 or older<br>Sex (female)<br>Median household income, dollars (37,999 or less): 38,000-47,999; 48,000-63,999; 64,000 or more<br>Diabetes without complications<br>Diabetes with complications<br>Chronic pulmonary disease<br>Rheumatologic disease<br>Renal disease<br>Congestive heart failure<br>Obese or overweight (defined according to ICD-9 codes, but the exact codes used were not specified nor was a BMI range	Age 45-54 = 662; 55-64 = 1791; 65-74 = 2748; 75-84 = 2178; 85 or older = 437<br>Sex: female = 4456; male = 3360<br>Median household income, dollars: 37,999 or less = 1781; 38,000-47,999 = 2034; 48,000-63,999 = 2082; 64,000 or more = 1787<br>Diabetes without complications = 1858<br>Diabetes with complications = 269<br>Chronic pulmonary disease = 1507<br>Rheumatologic disease = 376<br>Renal disease = 760<br>Congestive heart failure = 396<br>Obese or overweight = 1882	Age was categorised (45-54, 55-64, 65-74, 85 or older)<br><br>BMI was categorised (obese or overweight, according to ICD-9 code)

		given; reference category not reported)		
Webb 2017	As per NSQIP protocols, referenced in the paper	Insulin-dependent diabetes mellitus (non-diabetic)<br>Non-insulin-dependent diabetes mellitus (non-diabetic)	Cannot calculate accurately	Covariates:<br>Age was categorised into distinct categories of 15-54, 55-64, 65-74, and 75+<br>BMI was categorised into 18-25, 25-30, 30-35, and >35. This is similar, but not identical to, the WHO BMI categorisation system
Weick 2018	Quote from paper: "Prescription drug information was available for all patients included in the study. Specific opioid NDCs were identified in the databases using generic opioid drug names (hydrocodone, oxycodone, codeine phosphate, morphine, hydromorphone, fentanyl, methadone, meperidine, and tramadol). Matching the NDCs with the study subject allowed for the identification of the number and duration of preoperative prescriptions." – NDCs = National Drug Codes	No preoperative opioid use vs 1-30 days vs >30-60 days vs >60 days	Preoperative opioid use (calculated from % values and rounded down):<br>0-60 days = 14,571<br>>60 days = 3364	Preoperative opioid use was categorised into no preoperative use, 1-30 days, >30-60 days, and >60 days
Welsh 2017	Age and sex from MedPAR; race/ethnicity from enrolment indicator files; CCI based on ICD codes; traumatic admissions coded using MedPAR variables; disability entitlement from enrolment indicator files	Male sex<br>Race/ethnicity (white)<br>Disability entitlement<br>Surgery type (unilateral)<br>Admission type (elective)<br>Number of previous admissions in the past year<br>CCI	Calculated from % values given in Table 1 and rounded down (based on N and 30-day Readmission % value):<br>Age: 66-70y = 7762; 71-80y = 16,328; 81+y = 8258<br>Sex: female = 19424; male = 13172<br>Race/ethnicity: White = 28711; Black = 1927; Hispanic = 1292; Other = 634<br>Disability: yes = 3564; no = 29146<br>Surgery type: unilateral = 30985; bilateral = 1451<br>Admission type: elective = 30344; traumatic = 2129<br>Prior acute stays: 0 = 23497; 1 = 6183; 2+ = 2745	Age was categorised into 66-70y, 71-80y, and 81+y

			CCI: (number of conditions) 0 = 14959; 1 = 11510; 2+ = 5788	
Workman 2019	A combination of administrative data review using CPT codes and retrospective chart exploration was used to confirm readmission diagnosis, patient demographics, and clinical comorbidities. The parameters obtained from administrative data review were body mass index (BMI), discharge status (rehab versus home), age, gender, race, and LOS. Medical comorbidities were assessed via diagnosis codes from the hospitals administrative data but supplemented and modified as necessary based on history and physical exam during chart review	Ethnicity (unclear reference group)<br>Sex (female)<br>Age (<65 years)<br>CKD<br>Chronic airway obstruction disease<br>Obesity and morbid obesity (BMI 30 used as threshold)<br>Atrial fibrillation<br>Coronary atherosclerosis	Only available for the following predictors (derived from Table 2 in the study)<br>Sex = 86 male<br>Ethnicity: Caucasian = 184; African American = 4; Biracial = 22	Age was dichotomised at 65 years<br><br>BMI was dichotomised at 30kg/m^2
Yohe 2018	Trained clinical reviewers extract information as per NSQIP protocols	ASA 3/4/5 (1/2)<br>Functional status partially or totally dependent (independent)<br>COPD<br>CHF<br>Age 85+ (81-84)<br>Male sex<br>Hispanic ethnicity (non-Hispanic)<br>BMI (ref = normal): underweight (<18.5); overweight (≤25.0 to <30.0); obese (≥30.0)	Not reported	Age categorised into 81-84 years vs 85+ years.<br><br>BMI categorised into normal (18.5 to < 25.0), underweight (<18.5), overweight (≤25.0 to <30.0), and obese (≥30.0)
Zusmanovic 2018	Direct quote from the paper: "Data are abstracted at each site by surgical certified reviewers using clinical records, physician charts, and by contacting patients directly. Surgical certified reviewers are intensively trained with continuing education courses to standardize data collection. Data definitions are rigorous and standardized across all participating institutions. Data consistency and reliability	Obesity categories (reference = normal weight (18.5-24.9kg/m^2):<br>Overweight = 25.0-29.9<br>Obese class I (O1) = 30-34.9<br>Obese class II (O2) = 35-39.9<br>Obese class III (O3) = >40	See 'Participants' table	BMI was categorised into obesity categories approximating the WHO categorisation system, although the authors did not state that they adhered to this system

	are assessed at each hospital through an on-site interrater reliability audit program [10].”			

S7 - Adjustment for confounding

Adjustment for confounding						
Study ID	**Matching procedure:**	**Predictor variable selection method:**	**Type of multivariate regression model:**	**Covariates:**		**Model performance metric (result):**
				**Method of selection for adjusted covariates:**	**Covariates included:**	
Abola 2018	N/A	The main predictor of interest was hyponatraemia, based on prior literature. Each covariate included in the multivariate logistic regression model was also analysed independently for its impact on readmission risk	Logistic regression	Based on a priori clinical significance	Age, sex, race, BMI, history of diabetes, smoking, dyspnoea, functional status, history of CHF, history of COPD, dialyses, hypertension requiring medication, disseminated cancer, chronic steroid use, ASA class, bleeding disorders, weight loss, evidence of wound infection, preoperative blood transfusion, sepsis.	Not reported
Abdulla 2020	N/A	Based on prior literature and clinical reasoning	Logistic regression	Selected a priori	Age, sex, presurgical risk factors (presence of any of: history of thromboembolic disease, cardiac disease, dementia, moderate or severe mental illness, chronic pulmonary dysfunction, chronic hepatic dysfunction, renal dysfunction, diabetes with complications, history of stroke, malignancy)	Not reported
Ali 2019	N/A	The primary aim of the study was to assess and compare patient-related predictors of 30-day readmission after elective primary TKA, therefore a broad range of predictors were selected a priori	Multiple logistic regression	Each predictor variable in the logistic regression model was treated in turn as a covariate in order to estimate the independent contribution of each predictor to readmission risk	See earlier 'predictor variables' column	Not reported
Alvi 2015	Patients in the overall (THA +	Based on prior literature regarding	Logistic regression	After matching, the overall (THA + TKA)	ASA class, smoking status, steroid use, hypertension medications, history of	Not reported

	TKA) cohort were randomly matched 1:1:1:1:1 into BMI categories (18.5-25, 25-30, 30-35, 35-40, 40+) on the basis of equal proportions in age (<75 or ≥ 75 years), gender, procedure type (THA or TKA), and ASA class	the impact of BMI on total joint arthroplasty outcomes		cohort BMI groups were compared for differences in weight loss, diabetes, active smoker, steroid use, emergency case, COPD, CHF <30 days, dyspnoea, hypertension, dialysis, disseminated cancer, open wound/wound infection, bleeding disorders, and sepsis	COPD, creatinine, platelet counts, WBC counts, anaesthesia type, race, age, gender	
Anderson 2020	N/A	Based on prior literature and clinical reasoning	Logistic regression	Selected a priori	Sex, race, age at surgery, BMI, ASA class, CCI, length of surgery, anaesthesia method, and VA centre	Not reported
Anthony 2018	N/A	Selected a priori. The main predictor of interest was seasonality, which is not patient-related. Additional predictors were selected as covariates and their independent association with readmission was assessed	Logistic regression	Selected a priori	Age, sex, length of stay, Elixhauser Comorbiditiy Index, , number of diagnoses on the record, number of procedures on the record, patient location<br><br>Hospital-level: size, teaching/metropolitan status	Not reported
Antoniak 2020	N/A	N/A (only the primary predictor of interest was analysed)	N/A for readmission (multivariate logistic regression for the composite endpoint, major complications)	N/A for readmission	N/A for readmission	N/A for readmission
Arroyo 2019	N/A	Variables selected a priori: age, race, sex, insurance	Logistic regression	The authors selected some covariates a priori and the rest	Variables included as covariates because they had p <0.05 on bivariate analysis: Age in years, Gender, Race (White, Black,	N/A, but model assumptions of normality and

		type, median household income of patient's zip code, procedure state, procedure year, UTKA/BTKA		based on p <0.05 on bivariate analysis	Hispanic, Other, Missing), Payer (Medicare, Medicaid, Private insurance (self-pay/no charge or other)), year of surgery, stata, median income quartile of postal (ZIP) code, Elixhauser Index - the van Walraven Score, Elixhouser Comorbidities = CHF, valvular disease, PVD, hypertension uncomplicated, hypertension complicated, other neurological disorders, chronic pulmonary disease, diabetes uncomplicated, diabetes complicated, renal failure, liver disease, solid tumour without metastasis, rheumatoid arthritis/collagen vascular diseases, coagulopathy, obesity, fluid and electrolyte disorders, blood loss anemia, deficiency anemia, alcohol abuse, drug abuse, psychoses, depression, LOS, total charges in 2016 dollars, hospital volume	linearity were assessed graphically and statistically
Belmont 2016	N/A	Selected a priori to analyse patient-based factors that influence 30-day readmission in revision TKA	Logistic regression	Variables that differed on bivariate testing (p<0.2), that also maintained event frequencies greater than 10, were included in the multivariate logistic regression model	Sex, hypertension, previous TIA/CVA/stroke with neurologic deficit/CVA/stroke without neurologic deficit, type of anaesthesia, UTI, deep venous thrombosis, deep wound infection/organ or space SSI combined, superficial wound infection	C-statistic = 0.75 for final multivariate logistic regression model<br><br>Hosmer-Lemeshow goodness of fit test was used to assess model calibration, which showed no statistically significant lack of fit
Bovonratwet 2018	Propensity score matching – each of the 957 patients in the age ≥80 group was matched to one patient in each of the <70 and 70-79 age groups based on BMI,	Based on lack of evidence for the safety of revision TKA in octogenerians	N/A	Baseline characteristics that differed significantly (p<0.05) on univariate comparison between the age groups	Included in the propensity score matching procedure:<br>BMI, functional status prior to surgery, ASA class, diabetes mellitus, smoking status, anaesthesia type	N/A

	functional status prior to surgery, ASA class, diabetes mellitus, smoking status, and anaesthesia type. After this, at the author's specified α level of 0.05, there were no statistically significant differences between the age groups in terms of baseline characteristics.					
Bovonratwet 2019	N/A	Based on lack of evidence for safety of rapid hospital discharge in octogenarian TKA patients	Poisson regression with robust error variance	The final multivariate model was chosen based on a backward stepwise method, where variables with highest p-values were eliminated one by one until only variables with $p<0.05$ remained in the model. The following variables (from Table 1 in the paper) were included in the backward stepwise method: age (continuous), sex, BMI (categorical), functional status, ASA class, diabetes	Final set of covariates, determined by the backward stepwise method, was not reported	Not reported

				mellitus, smoking status, anaesthesia type, operative duration		
Bovonratwet 2020	N/A	Broad range of patient-related variables selected in order to gain a broad understanding of risk factors for readmission	Poisson regression	All predictor variables were included in the model, and backward stepwise variable selection was used with a p-value threshold of 0.05	Included in the model for backward stepwise selection: age, sex, BMI, functional status prior to surgery, ASA class, smoker, anaemia, diabetes mellitus, chronic steroid use, hypertension, dyspnoea on exertion, COPD, anaesthesia type, operative duration, preoperative INR, bleeding disorder<br><br>Included in the final model after backward stepwise selection: functional status prior to surgery, hypertension, COPD, operative duration	Not reported
Buitagro 2020	N/A	Based on prior literature and clinical reasoning	Logistic regression	Unclear	Multilevel logistic regression analysis with health provider clusters was carried out. Otherwise, it is not clear what the model was adjusted for	Not reported
Bullock 2003	N/A	Based on prior literature indicating benefits of simultaneous bilateral TKA, the authors hypothesised there would be no difference in postoperative complications between unilateral and simultaneous bilateral TKA patients	N/A	N/A	N/A	N/A
Charette 2019	N/A	Based on authors' determination that more research is warranted to ascertain the potential impact of performing TKA	Logistic regression	Not reported	Unclear	Not reported

		surgery in younger patients				
Courtney 2018	N/A	Based on authors' concerns around gap in the literature pertaining to risk adjustment for infective revision TKA cases considering their potentially higher risk for postoperative complications compared to other revision TKA patients with different aetiologies	Logistic regression	Selected a priori to identify independent risk factors for readmission	All predictor variables included in the multivariate model were also treated as covariates in order to calculate the independent risk conferred by each variable:<br>Male sex<br>Minority ethnicity<br>Age >70<br>BMI >35<br>Diabetes mellitus<br>Smoking history<br>CHF<br>Hypertension<br>Dialysis<br>Preoperative creatinine >1.5 mg/dL<br>Preoperative albumin <3.5 g/dL<br>ASA ≥4	Not reported
Curtis 2018	N/A	Based on a gap in the literature pertaining to TKA outcomes in chronically immunosuppressed patients who do not have inflammatory arthropathies or osteonecrosis	Logistic regression	A range of demographic, comorbidity, and laboratory variables were compared between the immunosuppressed patients and controls. Those that were found to be significantly different (at $p < 0.05$) on univariate analysis were subsequently included in the multivariate logistic regression model	Age, sex, BMI, ASA, current smoker, COPD, CHF, cancer, wound infection, bleeding disorder, BUN, creatinine, WBC count, haematocrit, platelets	Not reported
Curtis 2019	N/A	Functional status has been investigated as a predictor for postoperative complications in other surgical	Logistic regression	A range of demographic, comorbidity, and laboratory variables were compared between the immunosuppressed	age (continuous)<br>age >75<br>sex<br>BMI<br>BMI >= 40<br>ASA class >= 3<br>general anaesthesia	Not reported

		procedures but not TKA. The authors sought to fill this gap.		patients and controls. Those that were found to be significantly different (at $p < 0.05$) on univariate analysis were subsequently included in the multivariate logistic regression model	current smoker<br>diabetes<br>hypertension<br>CHF<br>dyspnoea<br>COPD<br>dialysis<br><br>sepsis<br>preoperative sodium (mEq/L)<br>preoperative BUN (mg/dL)<br>preoperative creatinine (mg/dL)<br>preoperative WBC (cells/mcL)<br>haematocrit (%)<br><br>Categorical and numerical (continuous) age and BMI were each significantly different between the two groups, and it is unclear whether the multivariate logistic regression model adjusted for age as a continuous or categorical variable	
D'Apuzzo 2017	N/A	Selected a priori based on aim to identify risk factors for readmission broadly	Logistic regression	Unclear	Unclear	C-statistic:<br>All-cause readmission model = 0.69<br>TKA-specific model = 0.66<br>Expanded TKA-specific model = 0.66
George 2018	N/A	Based on a gap in the literature pertaining to the apparent non-linear relationship between BMI and TKA complications	Logistic regression (for BMI analysed as a categorical variable) and restricted cubic spline (for BMI analysed as a continuous variable)	Demographics and preoperative comorbidities of all patients were compared between the different weight categories (chi-square for categorical variables, ANOVA for continuous variables), and univariate logistic	Age, smoking, CHF, COPD, diabetes, disseminated cancer, dialysis, corticosteroid use, recent weight loss<br>Of these variables, the following had missing data and were therefore excluded from the multivariate logistic regression model: sex, race, anaesthesia, ASA class, and functional status	Not reported

				regression analyses to compare 30-day readmission rates and complications between the various weight categories (reference category = normal weight), multivariate models were developed to adjust for different confounding and mediating variables - to avoid overfitting, only baseline variables p <0.01 different between weight categories were included in the multivariate analysis; all the outcomes which showed significant difference between the weight categories on multivariate analysis were further analysed using BMI as a continuous variable - BMI was included as a restricted cubic spline term with four knots in the logistic regression analysis - Wald statistic was used to assess whether the relationship between BMI and an outcome was nonlinear - predictive plots of the spline regression		

				models were created to graphically assess the relationship between BMI and outcome - for ease of interpretation, OR (odds of having an outcome at a specified BMI compared to the median BMI) were used in the predictive plots - some patients had BMI greater than 60 and these were not plotted - significance level was set at $p < 0.05$		
Gwam 2020	Propensity score matching on age, BMI, sex, race, Hispanic ethnicity, history of chronic dyspnoea, history of disseminated cancer, weight loss ≥10% of total body weight in the six months prior to index procedure, preoperative INR, preoperative platelet count, preoperative haematocrit count,	Based on prior literature and clinical reasoning	Generalised regression modelling, with penalised regression models used to account for incidences (of readmission, for a given predictor variable) less than 15	Selected a priori	Preoperative haematocrit count remained unbalanced after matching and was subsequently adjusted for.	Not reported

	preoperative functional status. A 1:1 nearest neighbour match was conducted, with a standardised mean difference threshold of 0.1 set as the threshold to determine residual imbalances.					
Hanly 2017	N/A	Based on literature suggesting worse outcomes for patients with obesity undergoing TKA, the authors sought to analyse the impact of morbid obesity	N/A	N/A	N/A	N/A
Hart 2016	Simultaneous bilateral TKA patients matched 1:4 to unilateral TKA patients during the same timeframe based on age, gender, and ASA scores. Approximate string matching, which determines approximate matches according to patterns in	Based on a gap in the literature pertaining to the difference between unilateral TKA and simultaneous bilateral TKA in terms of readmission rates, in part due to prior studies lacking sufficient sample size<br><br>Variables with a medical record completion rate below 80% were	Logistic regression.	Selected a prior for a broad range of covariates thought to be important by the authors. Interaction and non-linear terms were added to future models while variables believed to be unlikely true confounders were excluded from the final model.	The covariates in Table 1 (listed below) were included in the baseline model, but some of these may have been removed from future models:<br>Age, sex, race, BMI, smoking status, hypertension, diabetes, COPD, CHF and dyspnoea, history of steroid use, preoperative creatinine, preoperative BUN, preoperative haematocrit, platelets, ASA class, anaesthesia type, length of stay, transfusion within 72 hours, discharge destination, length of stay	Stability of models was assessed by comparing the chi-square likelihood ratio, -2 Log L, and Akaike Information Criterion (AIC) to each previous model. The final model was chosen based upon its ability to best predict a readmission or major complication following bilateral and unilateral TKA

	variables when an exact match is possible, was chosen. A nearest neighbour approach was used to locate the matches based on a greedy matching algorithm.	excluded from statistical analysis.				
Jauregui 2015	N/A	Selected a priori	N/A	N/A	N/A	N/A
Jorgensen 2013	N/A	Based on a gap in the literature pertaining to the impact of smoking and alcohol use on surgical outcomes in the fast-track arthroplasty population	N/A<br>The multivariate logistic regression model reported in the paper used data from the overall TKA + THA cohort.	N/A	N/A	N/A
Jorgensen 2017	N/A	Several fast-track arthroplasty centres in Denmark introduced high-dose glucocorticoids as part of postoperative multimodal analgesia, and the authors sought to analyse the impact of MP on surgical outcomes. A range of patient-related variables were selected for the adjusted analysis.	Logistic regression	Selected a priori	Age group, BMI group, smoking, alcohol use >24g/day, use of walking aids, living alone or in an institution, preoperative anaemia, pharmacologically treated cardiac disease, pharmacologically treated pulmonary disease, pharmacologically treated psychiatric disorder, use of anticoagulants, and NIDDM.<br>Place of surgery was included as a random effect	Model fit was evaluated using SPSS (v20) Model Viewer, and varied from 91% to 97% correct classifications

Keeney 2015	N/A	Based on prior literature and clinical insight	N/A	N/A	N/A	N/A
Kester 2016	N/A	Based on prior literature and clinical insight	Logistic regression	Variables that were significantly different (at p<0.2 and with at least 5 incidences) on univariate analysis – variables included for univariate comparison were selected a prior to give a broad ranges of pertinent clinical factors	Sex, age, BMI, current smoker, insulin-dependent diabetes mellitus, hypertension requiring medications, dialysis, ASA class	Hosmer-Lemeshow for calibration and c-statistic for goodness of fit (results not reported)
Kheir 2014	N/A	A priori clinical reasoning	Logistic regression	Selected a priori	Sex and race	Not reported
Kim 2019	N/A	Based on prior literature pertaining to adverse surgical patients in patients using opioids preoperatively	Cox proportional hazards	Selected a priori	Model 1 = age, sex, race/ethnicity, region of residence<br><br>Model 2 = age, sex, race/ethnicity, region of residence, combined comorbidity score, frailty score, number of unique prescription drugs	Not reported
Kuo 2017	CKD patients were case-matched 1:2 with non-CKD patients based on age, sex, BMI, and ASA grade. After matching, there was no statistically significant (p<0.05) difference between CKD group and non-CKD group for	Minimally invasive TKA is associated with reduced blood loss, among other things, and the authors sought to analyse the post-TKA outcomes in CKD patients given their aberrant haemostatic response compared to non-CKD individuals	Logistic regression	Selected a priori	The authors stated that the model incorporated “all possible factors” (page 1631), including: age, sex, BMI, ASA class, diagnosis (% osteoarthritis), preoperative Hb, PT/INR, and platelet count, preoperative knee deformity by mechanical axis, range of knee motion, CAD, CHF, DM, liver disease, lung disease, gout	Not reported

	any of these characteristics					
Kurtz 2016	N/A	A range of patient-related factors were selected in order to determine which of those factors increased risk of readmission.	Multilevel (individual-level and hospital-level factors) logistic regression, clustered by the hospital	Unclear	Unclear	Not reported
Nowak and Schemitsch 2019	Propensity score matching (between LOS groups) on age, BMI, sex, preoperative haematocrit, ASA class, operation length, CHF, COPD, bleeding disorder, smoking status, anaesthesia type, hypertension, steroid use, disseminated cancer, and functional status	Not stated – seems to be selected based on clinical/a priori reasoning	Poisson regression	Not stated – seems to be selected based on clinical/a priori reasoning	Unclear	C-statistic and Hosmer-Lemeshow tests<br><br>Multicollinearity was assessed using tolerance and variance inflation factors
Lehtonen 2018	N/A	Broad range of patient-related variables selected a priori and compared between readmitted and non-readmitted groups on univariate analysis. Those that were significantly different on univariate analysis ($p<0.0001$)	Logistic regression	Variables that achieved statistically significant ($p<0.0001$) difference on univariate comparison were included as covariates in the multivariate logistic regression model, including a dichotomous variable indicating	All predictor variables included in the multivariate model were also treated as covariates in order to calculate the independent risk conferred by each variable:<br>Age (continuous)<br>Sex<br>Race<br>BMI category<br>Comorbidity (dichotomous – presence or absence of ≥1 comorbidity)<br>ASA class<br>Operative time	Not reported

				presence or absence of at least one comorbidity		
Liao 2016	N/A	Based on prior literature, the authors identified the growing population of COPD patients receiving TKA as a group potentially at higher risk of adverse surgical outcomes.<br><br>Variables included in the multivariate Cox regression analysis were those demographic and comorbidity variables that were found to be statistically significant in univariate analysis	Cox regression	Variables included in the multivariate Cox regression analysis were those demographic and comorbidity variables that were found to be statistically significant in univariate analysis	COPD, CVD, CKD, DM, sex	N/A
Lovecchio 2014	N/A	The authors were only interested in the impact of their primary predictors (NIDDM and IDDM, each compared to no diabetes)	Logistic regression	Authors appear to have used a variable selection method based on clinical (rather than statistical) reasoning	ASA classification, age, sex, race, BMI, hypertension, steroid use, history of COPD, preoperative lab values, anaesthetic type, total work relative value unit (RVU)	Not reported
Miric 2014	N/A	Based on prior literature, the authors identified the growing population of nonagenarians receiving TKA as a group potentially at higher risk of	N/A	N/A	N/A	N/A

		adverse surgical outcomes				
Mudumbai 2019	N/A	Based on prior literature and clinical insight, the primary predictor variable of interest was preoperative opioid use. The authors also selected a comprehensive range of other patient-related characteristics a priori in order to determine their potential impact on readmission risk	N/A	N/A	N/A	N/A
Ottesen 2018	Coarsened exact matching (CEM), with one-to-many matching on categorical age, gender, BMI, functional status, and ASA class	Dialysis dependence was identified as a potential independent risk factor for postoperative adverse events, based on prior literature and clinical insight	Logistic regression	Based on a priori selection of patient-related variables	Categorical age, gender, BMI, functional status, and ASA class	To check the accuracy of the model, the authors compared the L1 statistic before and after CEM. CEM reduced the L1 value from 0.54 to 0.48, indicating CEM reduced total covariance of the variables about the multivariate mean.
Patterson 2018	N/A	Dialysis dependence was identified as a potential independent risk factor for postoperative adverse events, based on prior literature and clinical insight	Poisson regression with robust error variance for nonparametrically distributed data	A range of patient-related characteristics were tested on univariate comparison between dialysis-dependent and non-dialysis-dependent patients. Those which achieved p-value <0.2 were included	Male sex, facility dweller, functionally dependent, BMI (categorical), anaemia, uraemia, hypoalbuminaemia, history of smoking, history of cardiovascular disease, history of pulmonary disease, anaesthesia type	Not reported

				in the multivariate model as covariates		
Patel 2020	N/A	Based on prior literature and clinical reasoning	Logistic regression	The multivariate logistic regression model used to analyse sex as a readmission risk factor in the TKA cohort was adjusted for those variables which differed significantly (p<0.05) on univariate comparison between male and female patients in the overall TJA (THA and TKA combined) cohort	Age, BMI, current smoker, chronic steroid use, ASA class, race, functional status, preoperative living environment, diabetes, hypertension, COPD, CHF, anaemia, bleeding disorder, dyspnoea, CKD, diagnosis (primary OA vs inflammatory arthritis vs post-traumatic arthritis), bilateral procedure, procedure length >100 minutes	Not reported
Peskun 2012	Matched for age, gender, anaesthetic type, and medical comorbidities	Not stated – seems to be selected based on clinical/a priori reasoning	Logistic regression	Not stated – seems to be selected based on clinical/a priori reasoning	Anaesthetic type	Not reported
Pugely 2013	N/A	The authors selected an initial list of patient-related predictor variables from the 200+ available NSQIP variables. The selection method appears to be based on clinical importance determined a priori. Patient-related variables were compared on univariate analysis (Table 3a of the article) and then	Logistic regression	Forward selection multivariate logistic regression analysis was used for all eligible predictor variables	Listed below are those variables which were eligible for inclusion in the multivariate logistic regression model based on p-value <0.1 in Table 3a of the article:<br>Age, sex, COPD, CHF, hypertension, diabetes, PVD, disseminated cancer, bleeding disorder, dialysis, chemotherapy within 30 days, mean pre-op BUN, mean pre-op albumin, mean pre-op platelets, mean pre-op INR, mean pre-op creatinine, ASA class	C-statistic for discrimination, and Hosmer-Lemeshow chi-square statistic for calibration. Results not reported.

		forward multivariate logistic regression analysis (p<0.05) was used to identify readmission risk factors, with a p-value of <0.1 used to defined variables eligible for inclusion in the multivariate model				
Ramos 2014	N/A	The predictor of interest was discharge destination, which is not considered a patient-related risk factor for the purpose of this review. Age, sex, and comorbidity were selected a priori as covariates, but estimates of their impact on readmission	Logistic regression	Method of selection not reported, but they were selected a priori	Each of the following were selected as covariates in the multivariate logistic regression model. However, they are also the only patient-related risk factors included in the analysis and therefore they are also the predictor variables included in this review.<br>Age, sex, comorbidity	Not reported
Ricciardi 2017	Readmitted patients were matched 1:2 to non-readmitted patients on a set of predefined covariates to control for confounding	After the matching procedure, univariate analysis was conducted to identify candidate variables to include in a multivariate analysis to identify independent predictors of readmission	Conditional logistic regression	Matching procedure = predefined set of covariates<br><br>Multivariate analysis = variables that were statistically significantly different between readmitted and non-readmitted patients in the univariate analysis were included as covariates in the subsequent multivariate analysis	Controlled for in the matching procedure:<br>Age (+/- 5)<br>Sex (exact)<br>Deyo-Charlson comorbidity index (exact)<br>Date of surgery (+/- 30 days)<br><br>Included in the multivariate regression model:<br>Procedure time (mins)<br>Tourniquet time (mins)<br>Length of stay >3 days<br>Depression	Akaike information criterion was used to select the most parsimonious multivariate conditional logistic regression model, given the low sample size of the study

Robinson 2017	N/A	Based on vascular surgery literature showing differences in surgical outcomes based on patient sex, the authors sought to determine the impact of sex on readmission post-TKA	N/A	N/A	N/A	N/A
Ross 2020	N/A	Not stated – seems to be selected based on clinical/a priori reasoning	Logistic regression	Not stated – seems to be selected based on clinical/a priori reasoning	Age, gender, CCI (based on a 1-year lookback in the Discharge Abstract Database (DAD), year of surgery, LOS, urgency status (elective vs urgent procedure), revision status (primary vs revision procedure), teaching hospital status (academic vs nonacademic hospital), discharge disposition (discharge home vs discharge to an inpatient rehabilitation facility)	Not reported
Roth 2019	N/A	Based on prior literature on the relationship between BMI and surgical outcomes, the authors selected BMI (analysed as a continuous variable, compared to being analyse as a categorical variable) to be correlated with post-revision TKA outcomes as this had not been done before	Restricted cubic spline with four knots in the logistic regression analysis	Univariate logistic regression was used to compare readmission rates between the various weight categories, and only those baseline variables demonstrating p<0.01 on univariate analysis were included in the multivariate model in order to reduce overfitting.	Age, male sex, race, ASA class, general anaesthesia, and comorbidities (i.e. number of comorbidities: 0, 1, or >1)	Not reported
Rudasill 2019	N/A	The authors were only interested in the impact of their	Logistic regression	Not stated – seems to be selected based	Age, sex, race, BMI, preoperative creatinine level, preoperative WBC count, ASA class, CCI, DM, smoking,	Not reported

		primary predictor (INR level)		on clinical/a priori reasoning	preoperative dialysis, COPD, CHF, bleeding disorders, use of antihypertensive medications, elective procedure, year of surgery	
Runner 2017	N/A	Based on prior literature and the need to risk-stratify patients in TKA for complications, the authors proposed the MFI as a potential method by which this can be done.<br>The rationale for other included predictors was not specifically stated	Logistic regression	Multivariate logistic regression with backward elimination was used to assess predictors of 30-day	Backward elimination was used to assess each individual predictor of 30-day readmission (listed below) while controlling for the others:<br>MFI<br>ASA class (1)<br>Age ≥60 (unclear reference category considering only patients aged ≥60 were included in the study)<br>Wound class (class I)<br>Male sex<br>BMI >40<br>Any occurrence (of adverse events other than mortality, during hospital stay)	N/A
Saucedo 2014	N/A	Not stated for the initial selection of candidate predictors. Of these, variables with p<0.10 in unadjusted logistic regression analyses were used in the subsequent multivariable analysis	Logistic regression	Variables with p<0.10 in unadjusted logistic regression analyses were used in the subsequent multivariable analysis.	Coronary artery disease, age (20-49 and ≥80), BMI (>30). There may have been others, but see 'evidence of selective reporting' column of Results table in this review	N/A
Schaeffer 2015	N/A	Based on their review of the literature pertaining to adverse surgical outcomes related to high ASA class, the authors hypothesised that ASA rating system would help identify patients at risk of readmission	N/A	N/A	N/A	N/A

Schairer 2014	N/A	Based on prior literature pertaining to different rates of complications in primary TKA compared to revision TKA	N/A	N/A	N/A	N/A
Singh 2013	N/A	The primary predictor of interest was sex. Rationale = (direct quote from paper) “Despite several studies examining the effect of sex on pain and functional outcomes after TJA, sex has largely gone underrecognized as it relates to surgical complications.”	Mixed-effects logistic regression, accounting for clustering at the hospital level with random hospital intercepts and including patient age, race/ethnicity/ surgical risk of mortality, insurance status, and hospital teaching status and procedure volume as fixed effects	Not reported	Age, race or ethnicity, hospital teaching status (teaching or nonteaching), insurance status (categorized as none or unknown, Medicaid, Medicare/government, or private), and hospital procedure volume.	Not reported
Siracuse 2017	N/A	Demographic and clinicopathalogic factors associated with 30-day post-total joint arthroplasty readmission were identified and selected from prior literature	Logistic regression	Factors determined to be statistically significant in the univariate analysis (at p<0.01) were included in the multivariate logistic regression model	All of the variables in Table II:<br>Age, sex, race, income quartile, primary payer, comorbidities	R^2 = 0.96726
Sloan 2020	N/A	Based on prior literature and clinical reasoning	Logistic regression	Selected a priori	Preoperative albumin, age, sex, and comorbidity status (ASA class >2, diabetes mellitus, hypertension requiring medication, tobacco use, or surgery under general anaesthesia)	Not reported. Receiver operator characteristic (ROC) analysis was carried out, but for a different purpose.

Sodhi and Anis et al 2019	N/A	The authors were only interested in the impact of their primary predictor (amount of planning, as indicated by elective vs non-elective status)	Logistic regression	Not stated – seems to be selected based on clinical/a priori reasoning	Age, sex, BMI, ASA, year of surgery	No model performance criteria reported, but variance inflation factors were used to assess multicollinearity, with 3 as the cut-off (there were no such variables)
Sodhi and Mont et al 2019	N/A	Direct quote: “Based on previous literature, we hypothesized that HCAHPS scores would have a relationship to readmissions.” And “ Survey questions that were found to be significantly different between cohorts (P < .05) were then analyzed with multivariable logistic regression”	Logistic regression	Selected patient-level demographic and preoperative covariates were either: 1) significantly different between readmission and non-readmission cohorts, or 2) identified as potential clinically significant predictors of patient experience scores	Age, hospital location, length of stay, revision surgery status, and baseline VR-12 MCS and PCS	Not reported
Suleiman 2015	Propensity score matching using logistic regression with a 1:4 variable ratio, parallel, balanced nearest neighbours approach. After matching, there were no statistically significant (p<0.05)	The authors sought to compare outcomes and potential risks/benefits of SB TKA compared to unilateral TKA, given the controversary around SB TKA in the orthopaedic community	N/A – a multivariate matching procedure was carried out, and then readmission was compared between the two groups (unilateral TKA vs SB TKA) using generalised linear models with logit link	Not specifically reported, but the authors appear to have selected a range of demographic, comorbidity, and laboratory values based on a priori reasoning in order to reduce confounding	The following covariates were included in the propensity score matching procedure: Age, ASA class, bleeding disorder, race, general anaesthesia, diabetes, current smoker, functional status independent, history COPD, hypertension medication, steroid use, BMI (categorical)	There was no statistically significant difference detected between the matched cohorts on any of the variables used in the matching procedure

	differences between the matched cohorts for any baseline variables included.					
Sutton 2016	N/A	Unclear. Based on a priori criteria.	Logistic regression	Different models were compared, excluding variables thought to be unlikely true confounders of the relationship between length of stay and readmission that also had nonsignificant (p<0.05) likelihood estimates. Model stability was assessed by comparing chi-square likelihood ratio, -2 log(L), and Akaike Information Criterion (AIC) with those of previous models. The final model was chosen according to its ability to best predict readmission. The following covariates (i.e. all the variables listed in Table II) were initially considered:<br>Age, sex, BMI, race, smoking status, hypertension, diabetes, COPD, CHF & dyspnoea,	All the variables listed in table V:<br>Length of stay, age, sex, BMI, smoking status, hypertension, diabetes, COPD, CHF & dyspnoea, corticosteroid use, BUN, ASA classification, operative time, transfusion, discharge destination	Model stability was assessed by comparing chi-square likelihood ratio, -2 log(L), and Akaike Information Criterion (AIC) with those of previous models

				history of corticosteroid use, preoperative laboratory values (creatinine, BUN, haematocrit, platelets), ASA classification, anaesthesia type, operative time, transfusion within 72 hours after surgery, discharge destination		
Tang 2019	1:3 propensity score matching using a nearest neighbour matching algorithm and logistic regression estimation algorithm, based on sex, age, BMI, and procedure description (unilateral, bilateral, or revision). 0.02 was the chosen value of the matching caliper	Broad range selected a priori to provide comprehensive analysis of risk factors for readmissions (caused by complications).	Logistic regression	Covariates in the propensity score matching procedure: sex, age, BMI, procedure description<br><br>Selected a priori	Age, race, BMI, sex, procedure description, type of anaesthesia, duration of operation, perioperative blood transfusion, repeat operation within hospital stay, length of stay, day of week of operation, DM, anaemia, smoking, previous CVA or TIA, IHD, CHF, ASA-PS	PSM:<br>Relative multivariate imbalance L1 decreased from 0.933 to 0.417 and no covariate retained high degree of covariance (|d| > 0.25), and standard mean difference between all four covariates decreased below 0.1<br><br>Logistic regression model:<br>Not reported
Tay 2017	Direct quote: “The OGs were matched in a 1:1 ratio with younger controls (YGs) aged between 60 and 79 years, based	Based on prior research indicating the impact of older age and comorbidity burden on readmission risk, the authors were interested in differentiating the	Logistic regression	Not reported	Not reported	No

	on gender, body mass index, primary diagnosis, surgeon, side of surgery, history of contralateral TKA, and year of surgery. If there were multiple patients in the registry satisfying the matching criteria, the patient with the closest date of surgery to the subject was chosen to be the matched control. If there were no patients in the registry satisfying all the matching criteria, a best-fit patient who met the largest number of criteria was chosen to be the matched control.”	impact of comorbidity burden from that of older age. It is not clear why the YG group was restricted to patients ≥60y				
Urish 2018	N/A	Unclear	Logistic regression	Unclear	Age (categorical)<br>Sex<br>Median household income<br>Comorbidities<br>Index admission length of stay<br>Primary payer (Medicare or non-Medicare)<br>Hospital type (teaching or nonteaching)<br>Urban vs rural location	N/A

					Hospital size	
Webb 2017	N/A	The prognostic factor of interest was diabetes mellitus (insulin-dependent and non-insulin-dependent)	Poisson regression with robust error variance	Not specified	Age (categorical)<br>Sex<br>BMI (categorical)<br>CCI<br>Smoking status (yes or no)	N/A
Weick 2018	N/A	The prognostic factor of interest was preoperative opioid use	Logistic regression	Not specified	Age<br>Sex<br>CCI	N/A
Welsh 2017	N/A	The primary predictor of interest was discharge setting (skilled nursing facility, inpatient rehabilitation facility, community), with covariates selected seemingly based on clinical reasoning – each covariate was analysed for its independent impact on readmission risk and therefore was analysed as a predictor	Logistic regression	Based on a priori reasoning	Discharge setting<br>Age<br>Gender<br>Race/ethnicity<br>Disability entitlement<br>Surgery type<br>Admission type<br>Number of previous admissions<br>Hospital length of stay<br>Number of days in ICU<br>Charlson comorbidities<br>Hospital length of stay<br>Number of days in ICU<br>Charlson comorbidities<br>Hospital TKA volume	N/A
Workman 2019	N/A	Unclear method used to select predictor variables for univariate analysis, but only those which achieved statistical significance at p<0.05 (see Table 4 and Table 6) were included in	Logistic regression	Not specified, but it appears as though only variables that were statistically significantly different (at the p<0.05) level were included as covariates in the logistic regression model (i.e. all variables included in	Ethnicity (unclear reference group)<br>Sex (female)<br>Age (<65 years)<br>CKD<br>Chronic airway obstruction disease<br>Obesity and morbid obesity<br>Atrial fibrillation<br>Coronary atherosclerosis<br>Non-patient-related variables: discharge to rehabilitation, length of stay >3 days	N/A

		the multivariate analysis		the model were both predictors and covariates)		
Yohe 2018	N/A	Unclear – seems to be based on clinical reasoning	Logistic regression	The multivariate logistic regression model was used to calculate the adjusted odds of readmission for each predictive factor, therefore each predictive factor was also used as a covariate	All predictive factors were also used as covariates:<br>ASA<br>Functional status partially or totally dependent<br>COPD<br>CHF<br>Age<br>Sex<br>Ethnicity<br>BMI category<br>Anaesthesia (non-patient-related)	N/A
Zusmanovic 2018	N/A	N/A (only the primary predictor of interest was analysed)	(binary) Logistic regression	Direct quote from the paper: "Candidate preoperative variables for each regression were screened from those with P < .2 and at least 5 incidences in each of the cohorts from our previous univariate analysis" (page 859)	Unclear. The authors do not provide a reference for this 'previous univariate analysis', and they do not list the covariates in this study	N/A

S8 –Summary of Findings: Revision-only studies

Narrative Synthesis																
**Prognostic factor**	**Number of participants (study ID); number of studies; number of cohorts**	**Univariate**			**Multivariate**			**Phase**	**Study limitations**	**Inconsistency**	**Indirectness**	**Imprecision**	**Publication bias**	**Moderate /large effect size**	**Dose effect**	**Overall quality**
		+	0	-	+	0	-									
**ASA class ≥4 (reference category <4)**	10,844 (Courtney); 1; 1	-	-	-	-	1	-	2	✓	N/A	✓	X	X	X	X	3 (++)
**ASA class ≥3 (reference category 1-2)**	1754 (Belmont); 1; 1	1	-	-	-	-	-	1	✓	N/A	✓	X	X	X	X	2 (+)
**Total number of comorbidities**	1754 (Belmont); 1; 1	-	1	-	-	-	-	1	✓	N/A	X	X	X	X	X	1 (+)
**Age (continuous)**	1754 (Belmont); 1; 1	-	1	-	-	-	-	1	✓	N/A	✓	✓	X	X	X	3 (++)
**Age 60-69 (reference category ≤59)**	1754 (Belmont); 1; 1	-	1	-	-	-	-	1	✓	N/A	✓	X	X	X	X	2 (+)
**Age 70-79 (reference category ≤59)**	1754 (Belmont); 1; 1	-	1	-	-	-	-	1	✓	N/A	✓	X	X	X	X	2 (+)
**Age ≥80 (reference category ≤59)**	1754 (Belmont); 1; 1	-	1	-	-	-	-	1	✓	N/A	✓	X	X	X	X	2 (+)
**Hypertension**	12,598 (Belmont, Courtney); 2; 1	-	-	-	1	-	1	2	✓	✓	✓	✓	X	X	X	5 (+++)
**CHF**	10,844 (Courtney); 1; 1	-	-	-	1	-	-	2	✓	N/A	✓	X	X	X	X	3 (++)
**History of percutaneous intervention**	1754 (Belmont); 1; 1	-	1	-	-	-	-	1	✓	N/A	X	X	X	X	X	1 (+)

**Previous cardiac surgery**	1754 (Belmont); 1; 1	-	1	-	-	-	-	1	✓	N/A	X	X	X	X	X	1 (+)
**History revascularisation/ amputation for peripheral vascular disease/rest pain/ gangrene**	1754 (Belmont); 1; 1	-	1	-	-	-	-	1	✓	N/A	X	X	X	X	X	1 (+)
**BMI (continuous)**	9773 (Roth); 1; 1	-	-	-	1	-	-	2	✓	N/A	✓	✓	X	X	X	4 (+++)
**BMI 30.0-39.9 (reference <30)**	1754 (Belmont); 1; 1	-	1	-	-	-	-	1	✓	N/A	X	X	X	X	X	1 (+)
**BMI ≥40 (reference <30)**	1754 (Belmont); 1; 1	-	1	-	-	-	-	1	✓	N/A	X	X	X	X	X	1 (+)
**BMI >35**	10,844 (Courtney); 1; 1	-	-	-	1	-	-	2	✓	N/A	X	✓	X	X	X	3 (++)
**Overweight**	9773 (Roth); 1; 1	-	-	-	-	1	-	2	✓	N/A	X	✓	X	X	X	3 (++)
**Obese**	9773 (Roth); 1; 1	-	-	-	-	1	-	2	✓	N/A	X	✓	X	X	X	3 (++)
**Morbidly obese**	9773 (Roth); 1; 1	-	-	-	1	-	-	2	✓	N/A	X	✓	X	X	X	3 (++)
**Recent weight loss**	1754 (Belmont); 1; 1	1	-	-	-	-	-	1	✓	N/A	X	X	X	X	X	1 (+)
**Diabetes**	12,598 (Belmont, Courtney); 2; 1	-	1	-	-	1	-	1, 2	✓	N/A	X	X	X	X	X	2 (+)
**IDDM**	1754 (Belmont); 1; 1	-	1	-	-	-	-	1	✓	N/A	X	X	X	X	X	1 (+)
**NIDDM**	1754 (Belmont); 1; 1	-	1	-	-	-	-	1	✓	N/A	X	X	X	X	X	1 (+)

**Preoperative creatinine >1.5 mg/dL**	10,844 (Courtney); 1; 1	-	-	-	-	1	-	2	✓	N/A	✓	✓	X	X	X	4 (+++)
**Preoperative creatinine ≥2 g/dL**	10,844 (Courtney); 1; 1	-	1	-	-	-	-	1	✓	N/A	✓	X	X	X	X	2 (+)
**Preoperative albumin <3.5 g/dL**	10,844 (Courtney); 1; 1	-	-	-	-	1	-	2	✓	N/A	✓	✓	X	X	X	4 (+++)
**Preoperative serum albumin**	1754 (Belmont); 1; 1	-	-	1	-	-	-	1	✓	N/A	✓	✓	X	X	X	3 (++)
**Preoperative prealbumin ≤3.5 g/dL**	1754 (Belmont); 1; 1	-	1	-	-	-	-	1	✓	N/A	✓	X	X	X	X	2 (+)
**Preoperative INR**	1754 (Belmont); 1; 1	-	1	-	-	-	-	1	✓	N/A	✓	X	X	X	X	2 (+)
**Preoperative WBC count**	1754 (Belmont); 1; 1	-	1	-	-	-	-	1	✓	N/A	✓	✓	X	X	X	3 (++)
**Preoperative haematocrit**	1754 (Belmont); 1; 1	-	1	-	-	-	-	1	✓	N/A	✓	✓	X	X	X	3 (++)
**Preoperative platelets**	1754 (Belmont); 1; 1	-	1	-	-	-	-	1	✓	N/A	✓	✓	X	X	X	3 (++)
**Preoperative creatinine (continuous)**	1754 (Belmont); 1; 1	-	1	-	-	-	-	1	✓	N/A	✓	X	X	X	X	1 (+)
**Smoking history**	10,844 (Courtney); 1; 1	-	-	-	-	1	-	2	✓	N/A	X	✓	X	X	X	2 (+)
**COPD and chronic airways disease**	1754 (Belmont); 1; 1	-	1	-	-	-	-	1	✓	N/A	X	✓	X	X	X	2 (+)
**Dyspnoea**	1754 (Belmont); 1; 1	-	1	-	-	-	-	1	✓	N/A	X	✓	X	X	X	2 (+)
**Previous TIA/CVA/stroke with neurologic**	1754 (Belmont); 1; 1	-	-	-	1	-	-	2	✓	N/A	X	✓	X	X	X	3 (++)

**deficit/CVA/stroke without neurologic deficit**																
**Overall complications**	1754 (Belmont); 1; 1	1	-	-	-	-	-	2	✓	N/A	X	✓	X	✓	X	4 (+++)
**Mortality or major complication**	1754 (Belmont); 1; 1	1	-	-	-	-	-	2	✓	N/A	X	✓	X	✓	X	4 (+++)
**Major systemic complications**	1754 (Belmont); 1; 1	1	-	-	-	-	-	2	✓	N/A	X	✓	X	✓	X	4 (+++)
**Minor systemic complications**	1754 (Belmont); 1; 1	1	-	-	-	-	-	2	✓	N/A	X	✓	X	✓	X	4 (+++)
**Major local complications**	1754 (Belmont); 1; 1	1	-	-	-	-	-	2	✓	N/A	X	✓	X	✓	X	4 (+++)
**Minor local complications**	1754 (Belmont); 1; 1	1	-	-	-	-	-	2	✓	N/A	X	✓	X	✓	X	4 (+++)
**Complication - DVT**	1754 (Belmont); 1; 1	-	-	-	1	-	-	2	✓	N/A	✓	✓	X	✓	X	5 (+++)
**Complication - PE**	1754 (Belmont); 1; 1	1	-	-	-	-	-	2	✓	N/A	✓	✓	X	✓	X	5 (+++)
**Complication - UTI**	1754 (Belmont); 1; 1	-	-	-	1	-	-	2	✓	N/A	✓	✓	X	✓	X	5 (+++)
**Complication - Deep wound infection/organ or space SSI combined**	1754 (Belmont); 1; 1	-	-	-	1	-	-	2	✓	N/A	✓	✓	X	✓	X	5 (+++)
**Complication – superficial wound infection**	1754 (Belmont); 1; 1	-	-	-	1	-	-	2	✓	N/A	✓	✓	X	✓	X	5 (+++)
**Complication – Wound disruption**	1754 (Belmont); 1; 1	1	-	-	-	-	-	2	✓	N/A	✓	✓	X	✓	X	5 (+++)

**Major systemic complications: Post-operative sepsis/septic shock combined**	1754 (Belmont); 1; 1	1	-	-	-	-	-	2	✓	N/A	✓	✓	X	✓	X	5 (+++)
**Major systemic complications: Cardiac arrest requiring CPR**	1754 (Belmont); 1; 1	-	1	-	-	-	-	2	✓	N/A	✓	✓	X	✓	X	5 (+++)
**Major systemic complications: Unplanned intubation**	1754 (Belmont); 1; 1	-	1	-	-	-	-	2	✓	N/A	✓	✓	X	✓	X	5 (+++)
**Major systemic complications: Ventilator >48hrs**	1754 (Belmont); 1; 1	-	1	-	-	-	-	2	✓	N/A	✓	✓	X	✓	X	5 (+++)
**Minor systemic complications: Pneumonia**	1754 (Belmont); 1; 1	-	1	-	-	-	-	2	✓	N/A	✓	✓	X	✓	X	5 (+++)
**Minor systemic complications: Progressive renal insufficiency**	1754 (Belmont); 1; 1	1	-	-	-	-	-	2	✓	N/A	✓	✓	X	✓	X	5 (+++)
**Major systemic complications: Stroke/CVA**	1754 (Belmont); 1; 1	1	-	-	-	-	-	2	✓	N/A	✓	✓	X	✓	X	5 (+++)
**Major local complications: Peripheral nerve injury**	1754 (Belmont); 1; 1	-	1	-	-	-	-	2	✓	N/A	✓	✓	X	✓	X	5 (+++)
**Dialysis (including dialysis use/renal failure)**	1754 (Belmont); 1; 1	-	-	-	-	1	-	2	✓	N/A	✓	✓	X	X	X	4 (+++)
**Age >70**	1754 (Belmont); 1; 1	-	-	-	-	1	-	2	✓	N/A	X	✓	X	X	X	3 (++)
**Age <70 (reference category ≥80)**	1754 (Belmont); 1; 1	-	-	-	-	1	-	2	✓	N/A	X	✓	X	X	X	3 (++)

**Age 70-79 (reference category ≥80)**	1754 (Belmont); 1; 1	-	-	-	-	1	-	2	✓	N/A	X	✓	X	X	X	3 (++)
**Female sex**	1754 (Belmont); 1; 1	-	-	-	1	-	-	2	✓	N/A	✓	✓	X	X	X	4 (+++)
**Male sex**	10,844 (Courtney); 1; 1	-	-	-	1	-	-	2	✓	N/A	✓	✓	X	X	X	4 (+++)
**Infectious indication for revision TKA**	10,844 (Courtney); 1; 1	-	-	-	1	-	-	2	✓	N/A	✓	✓	X	X	X	4 (+++)
**Bleeding disorder**	1754 (Belmont); 1; 1	-	1	-	-	-	-	2	✓	N/A	✓	✓	X	X	X	4 (+++)
**Regular alcohol use**	1754 (Belmont); 1; 1	-	1	-	-	-	-	2	✓	N/A	✓	✓	X	X	X	4 (+++)
**Steroid use for chronic condition**	1754 (Belmont); 1; 1	-	1	-	-	-	-	2	✓	N/A	✓	✓	X	X	X	4 (+++)
**Wound classification other than clean (reference clean)**	1754 (Belmont); 1; 1	-	1	-	-	-	-	2	✓	N/A	✓	✓	X	X	X	4 (+++)
**Preoperative open wound**	1754 (Belmont); 1; 1	-	1	-	-	-	-	2	✓	N/A	✓	✓	X	X	X	4 (+++)
**Sepsis within 48 hours prior to surgery**	1754 (Belmont); 1; 1	-	1	-	-	-	-	2	✓	N/A	✓	✓	X	X	X	4 (+++)
**Partially/totally dependent functional status (reference independent)**	1754 (Belmont); 1; 1	-	1	-	-	-	-	2	✓	N/A	✓	✓	X	X	X	4 (+++)
**Prior operation of <30 days**	1754 (Belmont); 1; 1	-	1	-	-	-	-	2	✓	N/A	✓	✓	X	X	X	4 (+++)

✓ = No serious limitation in this criterion among the studies which analysed the given risk factor; X = serious limitation in this criterion among the studies which analysed the given risk factor; N/A given a score of zero, same as X; + very low quality = very little confidence in the effect estimate: true effect likely to be substantially different from the estimate of effect; ++ low quality = confidence in the effect estimate is limited: the true effect may be substantially different from the estimate of the study; +++ moderate quality = moderately confident in the effect estimate: true effect is likely to be close to the estimate of the effect, but there is a possibility that it is substantially different; ++++ high quality = very confident that the true effect lies close to that of the estimate of the effect

S9 - Results for all-cause readmission, adjusted analysis

Comorbidities				
**Study ID (overall risk of bias quartile – arranged in descended order from lowest to highest)**	**Effect size estimates:**		**Significance tests on matched cohorts:**	
	**Effect measure calculated (measure of confidence):**	**Result** (in **bold =** confidence intervals do not include null value**):**	**Method:**	**Result:**
Composite comorbidity indices				
Charlson Comorbidity Index (CCI):				
Kurtz 2016 (1st)	F-statistic*	CCI (F-statistic) = 15.5<br><br>CCI 1-2 = 37.5% overall TKA cohort, 42.1% readmitted cohort<br>CCI 3-4 = 6.7% overall TKA cohort, 11.6% readmitted cohort<br>CCI 5+ = 1.3% overall TKA cohort, 3.4% readmitted cohort	N/A	N/A
Ross 2020** (2nd)	OR (95% CI)	Reference category = 0<br><br>CCI:<br>**1 = 1.239 (1.167-1.317)**<br>**2 = 1.562 (1.43-1.706)**<br>**3+ = 2.12 (1.887-2.381)**	N/A	N/A
Welsh 2017 (2nd)	OR (95% CI)	Reference category = 0<br><br>CCI:<br>1 = **1.28 (1.25-1.31)**<br>>= 2 = **1.72 (1.66-1.78)**	N/A	N/A
Buitagro 2020 (3rd)	OR (95% CI)	CCI (reference = 0):<br>**1-2 = 1.24 (1.02-1.51)**<br>**≥3 = 1.64 (1.26-2.12)**	N/A	N/A

Tay 2017 (4th)	N/A	N/A	Pearson chi-square or Fisher exact	CCI: (0.050 Spearman Rho, p = 0.591) – i.e. no statistically significant (at p≤0.05) increase in readmission risk with increasing CCI
American Society of Anaesthesiologists (ASA) Classification:				
Pugely 2013 (1st)	OR (95% CI)	Reference category = 1 or 2<br><br>ASA Class 4 = **1.42 (1.15-1.74)**	N/A	N/A
Lehtonen 2018 (1st)	OR (95% CI)	Reference category = 2<br><br>ASA class:<br>1 = 1.19 (0.93 to 1.53)<br>3 = **1.43 (1.34-1.53)**<br>4 = **2.06 (1.73-2.44)**<br>Non Assigned = 1.75 (0.71 to 4.32)	N/A	N/A
Courtney 2018*** (1st)	OR (95% CI)	Reference category <4<br><br>ASA ≥4 = 1.223 (0.797-1.878)	N/A	N/A
Abola 2018 (1st)	OR (99% CI)	Reference category = 1<br><br>ASA class 2 = **0.73 (0.64–0.84)**<br>ASA class 3 = 1.09 (0.96–1.25)<br>ASA class 4/5 = **1.47 (1.17–1.86)**	N/A	N/A
Hart 2016 (2nd)	OR (95% CI)	Reference category = 1<br><br>ASA class:<br>2 = 0.74 (0.35-1.56)<br>3 = 1.13 (0.53-2.41)<br>4 = 0.67 (0.96-1.80)	N/A	N/A
Sutton 2016 (2nd)	OR (95% CI)	Reference category = 1<br><br>ASA class:<br>2 = **0.62 (0.39-0.99)**<br>3 = 0.93 (0.58-1.49)<br>4 = 1.08 (0.58-1.99)	N/A	N/A
Runner 2017 (2nd)	OR (95% CI)	Reference category = 1<br><br>ASA class:<br>2 = 0.84 (0.35-2.01)<br>3 = 1.22 (0.51-2.95)<br>4-5 = 0.75 (0.24-2.30)	N/A	N/A

Yohe 2018 (3rd)	OR (95% CI)	Reference category = 1 or 2<br><br>ASA 3 or 4 or 5 = **1.547 (1.135-2.220)**	N/A	N/A
Tang 2019** (3rd)	OR (95% CI)	Reference category = 1<br><br>Bivariate logistic regression (matched cohorts):<br>ASA-PS:<br>2 = 1.58 (0.43-5.82)<br>3 = 2.67 (0.46-15.35)	Chi-square for categorical variables and Mann-Whitney U test for continuous variables	Unless specified otherwise, results are presented as proportion in complication (leading to readmission) group vs proportion in no complication group = p-value.<br><br>ASA-PS (p-value = 0.54):<br>1 = 6.8% vs 10.8%<br>2 = 84.1% vs 83.8%<br>3 = 9.1% vs 5.4%
Ricciardi 2017 (3rd)	N/A	N/A	Chi-square test, and Fisher Exact test (when expected values n<5 in any field)	Results below are presented as proportion (%) in non-readmission group vs proportion (%) in readmission group (p-value).<br>ASA class:<br>1-2 = 69.6% vs 82.6% (0.24)<br>3-4 = 30.4% vs 17.4% (0.24)
Presence of any comorbidity:				
Lehtonen 2018 (1st)	OR (95% CI)	Comorbidity (presence vs absence):<br>Comorbidity = **1.29 (1.25-1.34)**	N/A	N/A
Ramos 2014 (3rd)	OR (95% CI)	Comorbidity = **3.829 (1.6-9.162)**	N/A	N/A
Elixhauser Index:				
Arroyo 2019 (2nd)	OR (95% CI)	Reference category = first tertile<br><br>Elixhauser Index:<br>Second tertile = **0.84 (0.81-0.87)**<br>Third tertile = **1.24 (1.20-1.28)**, missing = 0.96 (0.88-1.05)	N/A	N/A
		Cardiovascular		
Hypertension:				
Courtney 2018*** (1st)	OR (95% CI)	Hypertension = **1.230 (1.025-1.475)**	N/A	N/A
Belmont 2016*** (1st)	OR (95% CI)	Hypertension = **0.61 (0.39-0.96)**	N/A	N/A
Ali 2019 (1st)	OR (95% CI)	Hypertension = 1.02 (1.00-1.04; p = 0 .0697)	N/A	N/A
Abola 2018 (1st)	OR (99% CI)	Hypertension = **1.15 (1.09–1.22)**	N/A	N/A

Sutton 2016 (2nd)	OR (95% CI)	Hypertension = **1.36 (1.16-1.60)**	N/A	N/A
Hart 2016 (2nd)	OR (95% CI)	Hypertension = 1.26 (0.95-1.68)	N/A	N/A
Bovonratwet 2020 (2nd)	RR (95% CI)	**Hypertension = 2.5 (1.47-4.25)**	N/A	N/A
Peskun 2012 (3rd)	OR (95% CI)	Hypertension = 0	N/A	N/A
Nowak 2019 (3rd)	OR (95% CI)	Hypertension = **1.258 (1.145-1.382)**	N/A	N/A
Siracuse 2017 (3rd)	OR (95% CI)	Hypertension = **1.10 (1.07-1.14)**	N/A	N/A
D'Apuzzo 2017 (3rd)	OR (95% CI)	Hypertension (uncomplicated and complicated combined) = **1.06 (1.02-1.09)**	N/A	N/A
Ricciardi 2017 (3rd)	N/A	N/A	Chi-square test, and Fisher Exact test (when expected values n<5 in any field)	Results below are presented as proportion (%) in non-readmission group vs proportion (%) in readmission group (p-value).<br>Hypertension = 58.7% vs 56.5% (1.00)
Coronary artery disease (CAD), ischaemic heart disease (IHD), and previous acute myocardial infarction (AMI):				
Ali 2019 (1st)	OR (95% CI)	Previous AMI = 0.91 (0.71-1.17)	N/A	N/A
Peskun 2012 (3rd)	OR (95% CI)	MI/CAD = 5.73 (0.18-181.4)	N/A	N/A
Workman 2019 (3rd)	OR (95% CI)	Coronary atherosclerosis = 1.06 (0.662-1.71)	N/A	N/A
Saucedo 2014 (3rd)	OR (95% CI)	CAD = **1.79 (1.16-2.78)**	N/A	N/A
Tang 2019** (3rd)	OR (95% CI)	Bivariate logistic regression (matched cohorts):<br>IHD = 0.66 (0.18-2.43)<br><br>Multivariate logistic regression:<br>IHD = 0.44 (0.08-2.42)	N/A	N/A
Congestive Cardiac/Heart Failure (CCF/CHF):				
Urish 2018 (1st)	OR (95% CI)	CHF = **1.59 (1.43-1.78)**	N/A	N/A
Ali 2019 (1st)	OR (95% CI)	CHF = **1.08 (1.01-1.17)**	N/A	N/A
Abola 2018 (1st)	OR (99% CI)	CHF = 1.33 (0.99–1.79)	N/A	N/A
Courtney 2018*** (1st)	OR (95% CI)	CHF = **2.358 (1.381-4.026)**	N/A	N/A
Sutton 2016 (2nd)	OR (95% CI)	CHF and dyspnoea = **1.27 (1.03-1.57)**	N/A	N/A

Nowak 2019 (3rd)	OR (95% CI)	CHF = 0.890 (0.215-3.681)	N/A	N/A
Yohe 2018 (3rd)	OR (95% CI)	CHF = **3.030 (1.121-8.192)**	N/A	N/A
Siracuse 2017 (3rd)	OR (95% CI)	Congestive heart failure = **1.64 (1.53-1.76)**	N/A	N/A
Tang 2019** (3rd)	OR (95% CI)	Bivariate logistic regression (matched cohorts):<br>CHF = 1.49 (0.13-16.83)<br><br>Multivariate logistic regression:<br>CCF = 1.10 (0.06-21 .17)	Chi-square for categorical variables and Mann-Whitney U test for continuous variables	Unless specified otherwise, results are presented as proportion in complication (leading to readmission) group vs proportion in no complication group = p-value.<br><br>CHF = 2.3% vs 1.5% = 0.75
D'Apuzzo 2017 (3rd)	OR (95% CI)	Congestive heart failure = **1.48 (1.36-1.60)**	N/A	N/A
Arrhythmias:				
Ali 2019 (1st)	OR (95% CI)	Arrhythmias = **1.14 (1.10-1.18)**	N/A	N/A
Workman 2019 (3rd)	OR (95% CI)	AF = 1.03 (0.571-1.86)	N/A	N/A
Valvular disease:				
Ali 2019 (1st)	OR (95% CI)	Valvular heart disease = **1.09 (1.02-1.16)**	N/A	N/A
D'Apuzzo 2017 (3rd)	OR (95% CI)	Valvular disease = **1.21 (1.15, 1.29)**	N/A	N/A
Ricciardi 2017 (3rd)	N/A	N/A	Chi-square test, and Fisher Exact test (when expected values n<5 in any field)	Results below are presented as proportion (%) in non-readmission group vs proportion (%) in readmission group (p-value).<br>Valvular disease = 8.7% vs 17.4% (0.43)
Peripheral vascular disease:				
Ali 2019 (1st)	OR (95% CI)	Peripheral vascular disease = **1.17 (1.08-1.26)**	N/A	N/A
D'Apuzzo 2017 (3rd)	OR (95% CI)	Peripheral vascular disorder = **1.16 (1.05-1.27)**	N/A	N/A
Ricciardi 2017 (3rd)	N/A	N/A	Chi-square test, and Fisher Exact test (when expected values n<5 in any field)	Results below are presented as proportion (%) in non-readmission group vs proportion (%) in readmission group (p-value).<br>PVD = 2.2% vs 0 (1.00)
Cardiac disease:				

Kurtz 2016 (1st)	F-statistic	Heart disease (F-statistic) = 1343<br><br>Proportion in overall TKA cohort vs readmitted cohort unavailable	N/A	N/A
Jorgensen 2017 (1st)	OR (95% CI)	Pharmacologically treated cardiac disease = 1.41 (0.95–2.09)	N/A	N/A
BMI, obesity, and weight loss				
BMI continuous:				
George 2018 (2nd)	OR (95% CI)	N/A	N/A	Figure 1 shows that BMI and readmission had a U-shaped relationship with the lowest rate of readmission seen for patients with BMI closer to 30 and progressively higher rates seen for patients with lower and higher BMIs
Tang 2019** (3rd)	OR (95% CI)	Multivariate logistic regression:<br>BMI (no numbers given, but no difference detected on multivariate analysis between readmitted and non-readmitted patients) = 0.98 (0 .91-1 .07)	N/A	N/A
BMI categories:				
Lehtonen 2018 (1st)	OR (95% CI)	Reference category = overweight<br><br>BMI category:<br>Underweight = 1.06 (0.56 to 1.99)<br>Normal = 1.11 (0.99 to 1.23)<br>Obese = 0.96 (0.88 to 1.04)<br>Very obese = 0.92 (0.84 to 1.01)<br>Morbidly obese = **1.20 (1.08-1.32)**	N/A	N/A
Jorgensen 2017 (1st)	OR (95% CI)	Reference category = normal weight (18.5-25)<br><br>BMI:<br><18.5 = 0.42 (0.02–8.63)<br>25.1-29.9 = 0.82 (0.58–1.18)<br>30.0-34.9 = 0.89 (0.60–1.33)<br>35.0-39.9 = 1.09 (0.65–1.83)<br>≥40 = 0.70 (0.29–1.69)	N/A	N/A
Courtney 2018*** (1st)	OR (95% CI)	Reference category not reported<br><br>BMI >35 = **1.307 (1.108-1.542)**	N/A	N/A
Abola 2018 (1st)	OR (99% CI)	Reference category = 20-35<br><br>BMI <20 = 0.98 (0.67–1.44)<br>BMI >35 = 1.03 (0.84–1.26)	N/A	N/A

Sloan 2020 (2nd)	OR (95% CI)	Results from main logistic regression model (controlling for the full range of covariates):<br>Weight categories (reference = normal weight):<br>Underweight = 1.28 (0.62-2.63)<br>Overweight = 0.94 (0.82-1.08)<br>Obese class I = 0.92 (0.80-1.05)<br>**Obese class II = 0.82 (0.71-0.96)**<br>Obese class III = 1.08 (0.93-1.26)	N/A	N/A
Abdulla 2020 (2nd)	OR (95% CI)	Weight class (reference category = normal weight):<br>Overweight = 0.84 (0.62-1.14)<br>Obese class I = 0.82 (0.61-1.11)<br>Obese class II = 0.78 (0.56-1.09)<br>Obese class III = 1.07 (0.76-1.50)	N/A	N/A
Alvi 2015 (2nd)	OR (95% CI)	Reference category = normal weight (18.5-25)<br><br>BMI:<br>25-30 = 0.98 (0.45-2.14)<br>30-35 = 1.01 (0.46-2.22)<br>35-40 = 0.94 (0.41-2.15)<br>40+ = 0.97 (0.41-2.31)	N/A	N/A
Zusmanovic 2018 (2nd)	OR (95% CI)	Reference category = normal weight (18.5-25)<br><br>BMI categories:<br>Overweight = **0.87 (0.780-0.972)**<br>O1 = **0.909 (0.860-0.960)**<br>O2 = **0.912 (0.876-0.949)**<br>O3 = 0.981 (0.950-1.013)	N/A	N/A
George 2018 (2nd)	OR (95% CI)	Reference category = normal weight (18.5-25)<br><br>Overweight = 0.93 (0.84-1.03)<br>Obese = 0.92 (0.83-1.02)<br>Morbidly obese = **1.19 (1.06-1.34)**	N/A	N/A
Runner 2017 (2nd)	OR (95% CI)	Reference category = <40<br><br>BMI >40 = 1.10 (0.80-1.51)	N/A	N/A
Yohe 2018 (3rd)	OR (95% CI)	Reference category = normal weight (18.5-25)<br><br>Underweight = 0.839 (0.109-6.468)<br>Overweight = 1.091 (0.756-1.576)<br>Obese = 0.877 (0.590-1.302)	N/A	N/A
Saucedo 2014 (3rd)	OR (95% CI)	Reference category = 18.5-24.9<br><br>BMI >30 = **1.53 (1.07-2.30)**	N/A	N/A

Tang 2019** (3rd)	OR (95% CI)	Reference category <25<br><br>Bivariate logistic regression (matched cohorts):<br>BMI (<25):<br>25-29.9 = 1.46 (0.62-3.44)<br>30-34.9 = 0.78 (0.30-2.01)<br>≥35 = 1.29 (0.42-3.94)	Chi-square for categorical variables and Mann-Whitney U test for continuous variables	Unless specified otherwise, results are presented as proportion in complication (leading to readmission) group vs proportion in no complication group = p-value.<br><br>BMI (p-value = 0.59):<br><25 = 31.8% vs 34.6%<br>25-29.9 = 34.1% vs 25.4%<br>30-34.9 = 20.5% vs 28.5%<br>≥35 = 13.6% vs 11.5%
Obesity:				
Ali 2019 (1st)	OR (95% CI)	Obesity = **1.08 (1.04-1.12)**	N/A	N/A
Urish 2018 (1st)	OR (95% CI)	Obesity = **1.07 (1.01-1.30)**	N/A	N/A
Roth 2019*** (1st)	OR (95% CI)	Morbidly obese = **1.85 (1.27-2.69)**<br>Overweight 1.17 (0.81-1.69)<br>Obese = 1.32 (0.93-1.86)	N/A	N/A
Kurtz 2016 (1st)	F-statistic	Obesity (F-statistic) = 74.5<br><br>Proportion in overall TKA cohort vs readmitted cohort unavailable	N/A	N/A
Workman 2019 (3rd)	OR (95% CI)	Obesity and morbid obesity = **1.454 (1.006-2.10)**	N/A	N/A
D'Apuzzo 2017 (3rd)	OR (95% CI)	Obesity = 1.03 (0.99-1.07)	N/A	N/A
Ricciardi 2017 (3rd)	N/A	N/A	Chi-square test, and Fisher Exact test (when expected values n<5 in any field)	Results below are presented as proportion (%) in non-readmission group vs proportion (%) in readmission group (p-value).<br>Obesity = 26.1% vs 17.4% (0.42)
Weight loss:				
Ali 2019 (1st)	OR (95% CI)	Recent weight loss = 1.04 (0.87-1.25)	N/A	N/A
Abola 2018 (1st)	OR (99% CI)	Weight loss = 1.12 (0.64–1.95)	N/A	N/A
D'Apuzzo 2017 (3rd)	OR (95% CI)	Weight loss = 0.76 (0.48-1.21)	N/A	N/A
Endocrine				
Diabetes:				
Urish 2018 (1st)	OR (95% CI)	DM without complications = **1.17 (1.10-1.23)**<br>DM with complications = **1.22 (1.07-1.40)**	N/A	N/A

Kurtz 2016 (1st)	F-statistic	Diabetic (F-statistic) = 132<br><br>Proportion in overall TKA cohort vs readmitted cohort unavailable	N/A	N/A
Jorgensen 2017 (1st)	OR (95% CI)	NIDDM = 1.24 (0.79–1.93)	N/A	N/A
Courtney 2018*** (1st)	OR (95% CI)	Diabetes mellitus = 0.927 (0.767-1.121)	N/A	N/A
Ali 2019 (1st)	OR (95% CI)	Diabetes mellitus = **1.10 (1.07-1.14)**	N/A	N/A
Abola 2018 (1st)	OR (99% CI)	Diabetes = 1.03 (0.97–1.10)	N/A	N/A
Lovecchio 2014 (2nd)	OR (95% CI)	NIDDM = 0.98 (0.69-1.39)<br>IDDM = 1.37 (0.84-2.22)	N/A	N/A
Webb 2017 (2nd)	RR (99.7% CI)	IDDM vs non-DM = **1.65 (1.35-2.01)**<br>NIDDM vs non-DM = 1.12 (0.97-1.29)	N/A	N/A
Sutton 2016 (2nd)	OR (95% CI)	Diabetes = 1.06 (0.9-1.24)	N/A	N/A
Liao 2016** (2nd)	Hazard ratio (p-value)	DM = **1.65 (0.004)**	N/A	N/A
Peskun 2012 (3rd)	OR (95% CI)	T2DM = 2.2 (0.07-62.7)	N/A	N/A
Siracuse 2017 (3rd)	OR (95% CI)	Diabetes (includes complicated and uncomplicated diabetes) = **1.19 (1.15-1.23)**	N/A	N/A
Ricciardi 2017 (3rd)	N/A	N/A	Chi-square test, and Fisher Exact test (when expected values n<5 in any field)	Results below are presented as proportion (%) in non-readmission group vs proportion (%) in readmission group (p-value).<br>Diabetes without long-term complications = 10.9% vs 17.4% (0.47)<br>Diabetes without long-term complications = 10.9% vs 17.4% (0.47)
D'Apuzzo 2017 (3rd)	OR (95% CI)	Diabetes = **1.10 (1.06-1.14)**	N/A	N/A
Tang 2019** (3rd)	OR (95% CI)	Bivariate logistic regression (matched cohorts):<br>Diabetes mellitus = 0.89 (0.37-2.14)<br><br>Multivariate logistic regression:<br>Diabetes mellitus = 1.27 (0.42-3.87)	Chi-square for categorical variables and Mann-Whitney U test for continuous variables	Unless specified otherwise, results are presented as proportion in complication (leading to readmission) group vs proportion in no complication group = p-value.

				Diabetes mellitus = 18.2% vs 20.0% = 0.79
Hypothyroidism:				
Ali 2019 (1st)	OR (95% CI)	Hypothyroidism = 1.03 (0.99-1.07)	N/A	N/A
Gastrointestinal				
Peptic ulcer disease:				
Ali 2019 (1st)	OR (95% CI)	Peptic ulcer disease = 0.98 (0.85-1.13)	N/A	N/A
D'Apuzzo 2017 (3rd)	OR (95% CI)	Peptic ulcer disease excluding bleeding = 0.89 (0.74-1.08)	N/A	N/A
Liver disease:				
Ali 2019 (1st)	OR (95% CI)	Liver disease = **1.32 (1.17-1.49)**	N/A	N/A
D'Apuzzo 2017 (3rd)	OR (95% CI)	Liver disease = **1.28 (1.11-1.49)**	N/A	N/A
Siracuse 2017 (3rd)	OR (95% CI)	Liver disease = **1.27 (1.13-1.43)**	N/A	N/A
Ricciardi 2017 (3rd)	N/A	N/A	Chi-square test, and Fisher Exact test (when expected values n<5 in any field)	Results below are presented as proportion (%) in non-readmission group vs proportion (%) in readmission group (p-value).<br>Liver disease = 0 vs 4.3% (0.33)
Haematological				
Anaemia:				
Ali 2019 (1st)	OR (95% CI)	Anaemia due to blood loss = 0.98 (0.65-1.49)<br>Iron deficiency anaemia = 1.06 (0.98-1.14)	N/A	N/A
Kurtz 2016 (1st)	F-statistic	Anaemia (F-statistic) = 429.9<br><br>Proportion in overall TKA cohort vs readmitted cohort unavailable	N/A	N/A
Jorgensen 2017 (1st)	OR (95% CI)	Anaemia = 1.21 (0.81–1.81)	N/A	N/A
Tang 2019** (3rd)	OR (95% CI)	Bivariate logistic regression (matched cohorts):<br>Mild anaemia = 0.99 (0.49-2.04)<br>Moderate/severe anaemia = 1.83 (0.41-8.21)	Chi-square for categorical variables and Mann-Whitney U test for continuous variables	Unless specified otherwise, results are presented as proportion in complication (leading to readmission) group vs proportion in no complication group = p-value.<br><br>Anaemia (p-value = 0.72):<br>None = 54.5% vs 56.1%

				Mild = 38.6% vs 40.0%<br>Moderate/severe = 6.8% vs 3.8%
Siracuse 2017 (3rd)	OR (95% CI)	Anaemia = **1.19 (1.14-1.23)**	N/A	N/A
D'Apuzzo 2017 (3rd)	OR (95% CI)	Deficiency anaemias = **1.06 (1.00-1.12; p = 0.036)**	N/A	N/A
Ricciardi 2017 (3rd)	N/A	N/A	Chi-square test, and Fisher Exact test (when expected values n<5 in any field)	Results below are presented as proportion (%) in non-readmission group vs proportion (%) in readmission group (p-value).<br>Deficiency anaemias = 8.7% vs 13.0% (0.68)
Bleeding disorders:				
Ali 2019 (1st)	OR (95% CI)	Coagulopathy = **1.36 (1.19-1.55)**	N/A	N/A
Pugely 2013 (1st)	OR (95% CI)	History of bleeding disorder = **2.01 (1.34-3.01)**	N/A	N/A
Jorgensen 2017 (1st)	OR (95% CI)	Anticoagulant therapy = 0.97 (0.53–1.78)	N/A	N/A
Abola 2018 (1st)	OR (99% CI)	Bleeding disorders = **1.30 (1.17–1.45)**	N/A	N/A
Rudasill 2019 (2nd)	OR (95% CI)	Overall readmission:<br>INR >1-1.25 = unable to calculate<br>**INR >1.25-1.5 = 1.62 (1.22-2.15)**<br>**INR > 1.5 = 1.86 (1.09-3.17)**	N/A	N/A
D'Apuzzo 2017 (3rd)	OR (95% CI)	Coagulopathy = **1.19 (1.05-1.35)**	N/A	N/A
Siracuse 2017 (3rd)	OR (95% CI)	Coagulopathy = **1.22 (1.11-1.34)**	N/A	N/A
Nowak 2019 (3rd)	OR (95% CI)	Bleeding disorder = **1.739 (1.401-2.160)**	N/A	N/A
Blood dyscrazias:				
Courtney 2018*** (1st)	OR (95% CI)	Preoperative creatinine >1.5 mg/dL = 1.207 (0.949-1.536)<br>Preoperative albumin <3.5 g/dL = 1.267 (0.911-1.762)	N/A	N/A
Ali 2019 (1st)	OR (95% CI)	Fluid balance abnormality = 1.07 (0.99-1.14)	N/A	N/A
Pugely 2013 (1st)	OR (95% CI)	Serum BUN (continuous) = **1.02 (1.01-1.03)**	N/A	N/A
Abola 2018 (1st)	OR (99% CI)	Hyponatraemia: primary (imputed) analysis = 1.12 (1-1.24; p-value not given, but authors reported in-text that there was no significant association between hyponatraemia and readmission risk)	N/A	N/A
Sloan 2020 (2nd)	OR (95% CI)	**Hypoalbuminaemia = 1.62 (1.41-1.86)**	N/A	N/A
Sutton 2016 (2nd)	OR (95% CI)	Preop BUN per mg/dL = **1.01 (1.01-1.02)**	N/A	N/A

Hart 2016 (2nd)	OR (95% CI)	BUN = **1.02 (1.004-1.03)**	N/A	N/A
D'Apuzzo 2017 (3rd)	OR (95% CI)	Fluid and electrolyte disorders = 1.03 (0.94-1.12)	N/A	N/A
Siracuse 2017 (3rd)	OR (95% CI)	Fluid and electrolyte disorder = **1.25 (1.19-1.32)**	N/A	N/A
Ricciardi 2017 (3rd)	N/A	N/A	Chi-square test, and Fisher Exact test (when expected values n<5 in any field)	Results below are presented as proportion (%) in non-readmission group vs proportion (%) in readmission group (p-value). Fluid and electrolyte disorders = 4.3% vs 8.7% (0.60)
Respiratory				
COPD and chronic airways disease:				
Abola 2018 (1st)	OR (99% CI)	COPD = **1.28 (1.16–1.41)**	N/A	N/A
Hart 2016 (2nd)	OR (95% CI)	COPD = **1.86 (1.12-3.10)**	N/A	N/A
Bovonratwet 2020 (2nd)	RR (95% CI)	**COPD = 2.4 (1.01-5.62)**	N/A	N/A
Sutton 2016 (2nd)	OR (95% CI)	COPD = **1.37 (1.04-1.80)**	N/A	N/A
Liao 2016** (2nd)	Hazard ratio (p-value)	COPD = **1.63 (0.028)**	N/A	N/A
Peskun 2012 (3rd)	OR (95% CI)	COPD = 0	N/A	N/A
Nowak 2019 (3rd)	OR (95% CI)	COPD = **1.839 (1.562-2.166)**	N/A	N/A
Yohe 2018 (3rd)	OR (95% CI)	COPD = **1.694 (1.007-2.850)**	N/A	N/A
Workman 2019 (3rd)	OR (95% CI)	Chronic airway obstructive disease = **2.81 (1.535-5.14)**	N/A	N/A
Siracuse 2017 (3rd)	OR (95% CI)	Chronic obstructive pulmonary disease = **1.29 (1.24-1.34)**	N/A	N/A
Pulmonary disease:				
Ali 2019 (1st)	OR (95% CI)	Chronic pulmonary disease = **1.26 (1.23-1.30)**	N/A	N/A
Urish 2018 (1st)	OR (95% CI)	Chronic pulmonary disease = **1.35 (1.28-1.43)**	N/A	N/A
Kurtz 2016 (1st)	F-statistic	Pulmonary disease (F-statistic) = 510.7<br><br>Proportion in overall TKA cohort vs readmitted cohort unavailable	N/A	N/A
Jorgensen 2017 (1st)	OR (95% CI)	Pharmacologically treated pulmonary disease = 1.42 (0.93–2.17)	N/A	N/A
D'Apuzzo 2017 (3rd)	OR (95% CI)	Chronic pulmonary disease = **1.24 (1.19-1.29)**	N/A	N/A

Ricciardi 2017 (3rd)	N/A	N/A	Chi-square test, and Fisher Exact test (when expected values n<5 in any field)	Results below are presented as proportion (%) in non-readmission group vs proportion (%) in readmission group (p-value). Long-term pulmonary disease = 13.0% vs 8.7% (0.71)
Smoking:				
Abola 2018 (1st)	OR (99% CI)	Smoking = **1.10 (1.02–1.20)**	N/A	N/A
Courtney 2018*** (1st)	OR (95% CI)	Smoking history = 1.087 (0.855-1.383)	N/A	N/A
Jorgensen 2017 (1st)	OR (95% CI)	Smoking = 1.00 (0.65–1.55)	N/A	N/A
Hart 2016 (2nd)	OR (95% CI)	Current smoker = 1.35 (0.92-1.96)	N/A	N/A
Sutton 2016 (2nd)	OR (95% CI)	Smoking status - current smoker = **1.53 (1.24-1.89)**	N/A	N/A
Tang 2019** (3rd)	OR (95% CI)	Bivariate logistic regression (matched cohorts):<br>Smoking = 1.35 (0.52-3.53)<br><br>Multivariate logistic regression:<br>Smoking = 2.28 (0.65-7.97)	Chi-square for categorical variables and Mann-Whitney U test for continuous variables	Unless specified otherwise, results are presented as proportion in complication (leading to readmission) group vs proportion in no complication group = p-value.<br><br>Smoking = 15.9% vs 12.3% = 0.54
Pulmonary circulation disorder:				
Ali 2019 (1st)	OR (95% CI)	Pulmonary circulatory disease = **1.30 (1.17-1.44)**	N/A	N/A
D'Apuzzo 2017 (3rd)	OR (95% CI)	Pulmonary circulation disorder = 1.09 (0.93-1.26)	N/A	N/A
Dyspnoea:				
Abola 2018 (1st)	OR (99% CI)	Dyspnoea = 1.09 (1–1.19; p-value not reported)	N/A	N/A
Previous pneumonia:				
Ali 2019 (1st)	OR (95% CI)	Previous pneumonia = 1.00 (0.87-1.14)	N/A	N/A
Psychiatric				
Depression and mental health disorder:				
Ali 2019 (1st)	OR (95% CI)	Depression = **1.28 (1.21-1.36)**<br>Other mental health disorder = **1.30 (1.17-1.45)**	N/A	N/A
Kurtz 2016 (1st)	F-statistic	Depression (F-statistic) = 202.5<br><br>Proportion in overall TKA cohort vs readmitted cohort unavailable	N/A	N/A

Ricciardi 2017 (3rd)	OR (95% CI)	Depression = 3.35 (0.72-15.59)	Chi-square test, and Fisher Exact test (when expected values n<5 in any field)	Results below are presented as proportion (%) in non-readmission group vs proportion (%) in readmission group (p-value). Depression = **10.9% vs 34.8% (0.02)**
D'Apuzzo 2017 (3rd)	OR (95% CI)	Depression = **1.13 (1.08-1.19)**	N/A	N/A
Substance use:				
Jorgensen 2017 (1st)	OR (95% CI)	Alcohol use = 1.32 (0.80–2.17)	N/A	N/A
Ali 2019 (1st)	OR (95% CI)	Alcohol abuse = 1.06 (0.97-1.17)<br>Drug abuse = 1.17 (0.80-1.70)	N/A	N/A
Kurtz 2016 (1st)	F-statistic	Drug/alcohol abuse = 71.6	N/A	N/A
D'Apuzzo 2017 (3rd)	OR (95% CI)	Alcohol abuse = 1.08 (0.91- 1.27)<br>Drug abuse = **1.27 (1.02- 1.58)**	N/A	N/A
Psychoses and psychiatric disorder:				
Ali 2019 (1st)	OR (95% CI)	Psychoses = **1.69 (1.37-2.08)**	N/A	N/A
Jorgensen 2017 (1st)	OR (95% CI)	Psychiatric disorder = 1.33 (0.92–1.92)	N/A	N/A
D'Apuzzo 2017 (3rd)	OR (95% CI)	Psychoses = **1.32 (1.17-1.48)**	N/A	N/A
Neoplastic				
History of cancer including metastases:				
Ali 2019 (1st)	OR (95% CI)	Metastases = 0.92 (0.71-1.20)<br>Cancer diagnosis = 0.98 (0.90-1.06)	N/A	N/A
Abola 2018 (1st)	OR (99% CI)	Disseminated cancer = 1.45 (0.86–2.44)	N/A	N/A
Pugely 2013 (1st)	OR (95% CI)	History of cancer (includes planned readmissions for chemotherapy) = **11.73 (1.93-71.30)**	N/A	N/A
Kurtz 2016 (1st)	F-statistic	Secondary tumour (F-statistic) = 43.6<br><br>Proportion in overall TKA cohort vs readmitted cohort unavailable	N/A	N/A
D'Apuzzo 2017 (3rd)	OR (95% CI)	Solid tumor without metastasis = **1.15 (1.06-1.25)**	N/A	N/A
Lymphoma:				
Kurtz 2016 (1st)	F-statistic	Lymphoma (F-statistic) = 12.5<br><br>Proportion in overall TKA cohort vs readmitted cohort unavailable	N/A	N/A
D'Apuzzo 2017 (3rd)	OR (95% CI)	Lymphoma = **1.72 (1.23-2.40)**	N/A	N/A

Neurological				
Previous stroke:				
Belmont 2016*** (1st)	OR (95% CI)	Previous TIA/CVA/stroke with neurologic deficit/CVA/stroke without neurologic deficit = **3.47 (1.30-9.25)**	N/A	N/A
Ali 2019 (1st)	OR (95% CI)	Previous stroke = **0.76 (0.60-0.97)**	N/A	N/A
Tang 2019** (3rd)	OR (95% CI)	Bivariate logistic regression (matched cohorts):<br>Previous CVA or TIA = 4.23 (0.91-19.72)<br><br>Multivariate logistic regression:<br>Previous CVA or TIA = **11.59 (1.53-87 .53)**	Chi-square for categorical variables and Mann-Whitney U test for continuous variables	Unless specified otherwise, results are presented as proportion in complication (leading to readmission) group vs proportion in no complication group = p-value.<br><br>Previous CVA or TIA = 9.1% vs 2.3% = 0.05
Dementia:				
Ali 2019 (1st)	OR (95% CI)	Dementia = **1.24 (1.06-1.44)**	N/A	N/A
Paralysis and other neurological disorder:				
Ali 2019 (1st)	OR (95% CI)	Paraplegia = 1.11 (0.94-1.32)<br>Other neurological disorder = **1.40 (1.32-1.49)**	N/A	N/A
D'Apuzzo 2017 (3rd)	OR (95% CI)	Paralysis = 1.20 (0.89-1.63)<br>Other neurological disorder = **1.22 (1.12-1.33)**	N/A	N/A
Ricciardi 2017 (3rd)	N/A	N/A	Chi-square test, and Fisher Exact test (when expected values n<5 in any field)	Results below are presented as proportion (%) in non-readmission group vs proportion (%) in readmission group (p-value).<br>Other neurological disorder = 6.5% vs 4.3% (1.00)
Renal				
Chronic kidney disease (CKD):				
Liao 2016** (2nd)	Hazard ratio (p-value)	CKD = **1.98 (0.003)**	N/A	N/A
Workman 2019 (3rd)	OR (95% CI)	CKD = 1.956 (0.916-4.17)	N/A	N/A
Kuo 2017 (4th)	OR (95% CI)	CKD = **6.2 (1.98-12.8)**	N/A	N/A
Dialysis dependence:				
Courtney 2018*** (1st)	OR (95% CI)	Dialysis = 1.049 (0.469-2.346)	N/A	N/A
Abola 2018 (1st)	OR (99% CI)	Dialysis - current = **1.57 (1.11–2.23)**	N/A	N/A
Gwam 2020 (1st)	OR (95% CI)	Dialysis dependence = 1.99 (0.84-3.12)	N/A	N/A

Patterson 2018 (2nd)	RR (95% CI)	Dialysis dependence = **1.8 (1.2-2.6)**	N/A	N/A
Ottesen 2018 (2nd)	OR (95% CI)	Multivariable logistic regression following coarsened exact matching:<br>Dialysis dependence = **2.32 (1.47-3.66)**<br><br>Multivariable logistic regression analysis without coarsened exact matching:<br>Dialysis dependence = **2.33 (1.49-3.65)**	N/A	N/A
Renal failure – acute, preoperative:				
Abola 2018 (1st)	OR (99% CI)	Renal failure (acute, preoperative) = 1.30 (0.59–2.87)	N/A	N/A
Renal failure/disease – chronicity unspecified:				
Urish 2018 (1st)	OR (95% CI)	Renal disease = **1.55 (1.42-1.68)**	N/A	N/A
Ali 2019 (1st)	OR (95% CI)	Renal disease = **1.09 (1.04-1.15)**	N/A	N/A
Kurtz 2016 (1st)	F-statistic	Renal failure (F-statistic) = 1005<br><br>Proportion in overall TKA cohort vs readmitted cohort unavailable	N/A	N/A
Siracuse 2017 (3rd)	OR (95% CI)	Renal failure = **1.33 (1.25-1.42)**	N/A	N/A
D'Apuzzo 2017 (3rd)	OR (95% CI)	Renal failure = **1.35 (1.24-1.47)**	N/A	N/A
Rheumatological and autoimmune				
Rheumatoid arthritis:				
Siracuse 2017 (3rd)	OR (95% CI)	Rheumatoid arthritis = **1.14 (1.06-1.23)**	N/A	N/A
D'Apuzzo 2017 (3rd)	OR (95% CI)	Rheumatoid arthritis/collagen vascular disease = 1.09 (0.91-1.29)	N/A	N/A
Rheumatological disorder:				
Urish 2018 (1st)	OR (95% CI)	Rheumatologic disease = **1.20 (1.07-1.33)**	N/A	N/A
Ali 2019 (1st)	OR (95% CI)	Rheumatological disorder = 1.05 (1.00-1.09; p-value = 0.0668)	N/A	N/A
Steroid or other immunosuppressant use for chronic condition:				
Abola 2018 (1st)	OR (99% CI)	Steroids for chronic condition = **1.19 (1.07–1.32)**	N/A	N/A
Curtis 2018 (2nd)	OR (95% CI)	Immunosuppressant use = **1.26 (1.05-1.51)**	N/A	N/A
Hart 2016 (2nd)	OR (95% CI)	Steroid use = **2.33 (1.44-3.74)**	N/A	N/A
Other				
Preoperative opioid use:				
Kim 2019** (1st)	HR (95% CI)	Continuous opioid users<br>Model 1 = **1.57 (1.45-1.71)**<br>Model 2 = 1.06 (0.97-1.16)<br><br>Intermittent opioid use:<br>Model 1 = **1.21 (1.15-1.26)**<br>Model 2 = 0.99 (0.94-1.04)	N/A	N/A

Weick 2018 (3rd)	OR (95% CI)	>60 preoperative opioid use (compared to 0-60 days) = **1.13 (1.08-1.18)**	N/A	N/A
Preoperative medication utilisation :				
Anderson 2020 (2nd)	OR (95% CI)	Beers 0 (i.e. Medications from the 2015 Updated Beers List to be Used with Caution. Model was further adjusted for admitted Beers 1 and Beers 2 counts) = 1.01 (0.92-1.11)<br><br>Beers 1 (i.e. Medications from 2015 Updated Beers List to Avoid. Model was further adjusted for admitted Beers 0 and Beers 2 counts):<br>**At one-unit dose intervals = 1.03 (1.00-1.05; p=0.0154)**<br>At five-unit dose intervals = 1.14 (1.00-1.29; p-value not reported)<br>**At 10-unit dose intervals = 1.30 (1.01-1.66)**	N/A	N/A
Wound-related:				
Abola 2018 (1st)	OR (99% CI)	Wound infection/open wound (preoperative) = 1.34 (1–1.79; p-value not reported)	N/A	N/A
Runner 2017 (2nd)	OR (95% CI)	Wound class:<br>II = 0.08 (0.01-1.32)<br>III-IV = 2.66 (0.64-10.98)	N/A	N/A
Demographics				
Age				
Continuous:				
Lehtonen 2018 (1st)	OR (95% CI)	Age (non-readmitted = 66.53 vs readmitted = 68.49) = **1.021 (1.018-1.025)**	N/A	N/A
Hart 2016 (1st)	OR (95% CI)	Age (ages not reported separately for non-readmitted and readmitted patients) = 1.00 (0.99-1.02)	N/A	N/A
Ross 2020** (2nd)	OR (95% CI)	Age per 10y (total cohort = 67.59 (9.69) vs readmitted = 70.38 (10.16)) = **1.293 (1.26-1.327)**	N/A	N/A
Arroyo 2019 (2nd)	OR (95% CI)	Age (non-readmitted = 68.34 (10.85) vs readmitted = 69.23 (10.81)) = **1.01 (1.01-1.01)**	N/A	N/A
Welsh 2017 (2nd)	OR (95% CI)	Age (readmitted patients had advanced age compared to non-readmitted patients, but numbers were not given) = **1.04 (1.03-1.04)**	N/A	N/A
Sutton 2016 (2nd)	OR (95% CI)	Age (no numbers given, but authors found that 'age per year' increased risk of readmission – presumably this means increasing age) = 1.01 **(1.00-1.02, p-value 0.03)**	N/A	N/A
Ramos 2014 (3rd)	OR (95% CI)	Age (no numbers given, but no difference detected on multivariate analysis between readmitted and non-readmitted patients) = 0.978 (0.952-1.005)	N/A	N/A
Peskun 2012 (3rd)	OR (95% CI)	Age (no numbers given, but no difference detected on multivariate analysis between readmitted and non-readmitted patients) = 1 (0.8-1.17)	N/A	N/A
Tang 2019** (3rd)	OR (95% CI)	Multivariate logistic regression:<br>Age (non-readmitted = 66.3 (7.7) vs readmitted = 66.8 (8.9)) = 1.01 (0 .95-1.07)	N/A	N/A

Categorised:				
Kurtz 2016 (1st)	F-statistic*	Age (F-statistic) = 352.1<br><br>65-69 = 32.2% overall TKA cohort, 24.9% readmitted cohort<br>70-74 = 29.0% overall TKA cohort, 26.3% readmitted cohort<br>75-79 = 21.9% overall TKA cohort, 24.4% readmitted cohort<br>80-84 = 12.4% overall TKA cohort, 16.8% readmitted cohort<br>85+ = 4.4% overall TKA cohort, 7.5% readmitted cohort	N/A	N/A
Urish 2018 (1st)	OR (95% CI)	Reference category: 45-54<br><br>55-64 = 0.94 (0.08-1.03)<br>65-74 = **0.9 (0.81-1.00, p < 0.04)**<br>75-85 = **1.29 (1.16-1.44)**<br>85+ = **1.91 (1.66-2.20)**	N/A	N/A
Ali 2019 (1st)	OR (95% CI)	Reference category: 60-64<br><br>0-39 = **1.25 (1.05-1.50)**<br>40-44 = **1.15 (1.01-1.32)**<br>45-49 = **1.10 (1.00-1.20)**<br>50-54 = 0.99 (0.93-1.06)<br>55-59 = 1.00 (0.95-1.05)<br>65-69 = **1.08 (1.04-1.13)**<br>70-74 = **1.20 (1.15-1.24)**<br>75-79 = **1.41 (1.35-1.46)**<br>80-84 = **1.55 (1.49-1.62)**<br>85-89 = **1.80 (1.70-1.90)**<br>90+ = **1.72 (1.52-1.94)**	N/A	N/A
Abola 2018 (1st)	OR (99% CI)	Reference category: <40<br><br>Age 80+ = **1.33 (1.09–1.61)**<br>Age 40-59 = **0.74 (0.62–0.89)**<br>Age 60-79 = **0.82 (0.69–0.98)**	N/A	N/A
Pugely 2013 (1st)	OR (95% CI)	Reference category: 56-65<br><br><45 = **2.59 (1.44-4.67)**<br>76-85 = **1.42 (1.08-1.85)**<br>>85 = **1.79 (1.09-2.97)**	N/A	N/A
Jorgensen 2017 (1st)	OR (95% CI)	Reference category: 66-70<br><br><50 = 0.72 (0.29–1.80)<br>50-60 = 1.00 (0.61–1.65)<br>61-65 = 1.17 (0.72–1.91)<br>71-75 = 1.18 (0.75–1.84)	N/A	N/A

		**76-80 = 1.59 (1.00–2.51; p = 0.049)**<br>81-85 = 1.29 (0.71–2.37)<br>>85 = **2.27 (1.06–4.87)**		
Courtney 2018*** (1st)	OR (95% CI)	Reference category: not stated<br><br>Age >70 = 1.041 (0.878-1.235)	N/A	N/A
Bovonratwet 2018*** (1st)	N/A	N/A	Fisher's exact or chi-square	After propensity score matching:<br><br>Reference category: ≥80<br><br>Age <70 (5.45%) vs age ≥80 (6.23%): p = 0.499<br><br>Age 70-79 (5.61%) vs age ≥80 (6.23%): p = 0.594
Bovonratwet 2019 (1st)	RR (99.60% CI, due to Bonferroni correction)	Reference category: <80<br><br>Age ≥80 = 1.83 (1.15-2.92)	N/A	N/A
Runner 2017 (2nd)	OR (95% CI)	Reference category: unclear, considering only patients aged ≥60 were included in the study<br><br>Age ≥60 = **1.02 (1.01-1.03)**	N/A	N/A
Buitagro 2020 (3rd)	OR (95% CI)	Age (reference = ≥80):<br>≤49 = 0.50 (0.28-0.90)<br>**50-59 = 0.45 (0.32-0.63)**<br>**60-69 = 0.45 (0.34-0.60)**<br>**70-79 = 0.60 (0.46-0.78)**	N/A	N/A
Yohe 2018 (3rd)	OR (95% CI)	Reference category: 81-84<br><br>Age 85+ = 1.068 (0.787-1.451)	N/A	N/A
Workman 2019 (3rd)	OR (95% CI)	Reference category: <65<br><br>Age 65+ = **1.639 (1.21-2.213)**	N/A	N/A
Siracuse 2017 (3rd)	OR (95% CI)	Reference category: 51-60<br><br>Age:<br>41-50 = **1.13 (1.05-1.22)**<br>61-70 = 1.01 (0.97-1.06)<br>71-80 = **1.21 (1.15-1.28)**<br>81-90 = **1.70 (1.61-1.81)**	N/A	N/A

Saucedo 2014 (3rd)	OR (95% CI)	Reference category: 50-79<br><br>Age 20-49 vs 50-79 = **2.18 (1.09-4.35)**<br>Age >=80 vs 50-79 = **1.84 (1.20-2.81)**	N/A	N/A
D'Apuzzo 2017 (3rd)	OR (95% CI)	Reference category: <65<br><br>65-75 yr = 0.98 (0.94, 1.03)<br>76-85 yr = **1.25 (1.19, 1.31)**<br>>85 yr = **1.67 (1.54, 1.80)**	N/A	N/A
Tang 2019** (3rd)	OR (95% CI)	Bivariate logistic regression:<br>Reference category: <60<br><br>60-64 = 0.96 (0.33 2.80)<br>65-69 = 0.66 (0.22 1.95)<br>≥70 = 1 .33 (0.54-3 .25)	Chi-square for categorical variables and Mann-Whitney U test for continuous variables	Unless specified otherwise, results are presented as proportion in complication (leading to readmission) group vs proportion in no complication group = p-value.<br>Age (mean (SD)):<br>66.3 (7.7) vs 66.8 (8.9) = 0.70
Charette 2019 (4th)	OR (confidence interval not reported)	Reference category: ≥55<br><br>Age <55 = 0.9 (p = 0.391)	N/A	N/A
Tay 2017 (4th)	OR (95% CI)	Reference category: YG = <80<br><br>OG vs YG = 2.58 (0.8-8.35)	N/A	N/A
Sex				
Female:				
Belmont 2016*** (1st)	OR (95% CI)	Female sex = **1.75 (1.15-2.68)**	N/A	N/A
Lehtonen 2018 (1st)	OR (95% CI)	Female sex = **0.78 (0.73- 0.83)**	N/A	N/A
Abola 2018 (1st)	OR (99% CI)	Female sex = **0.87 (0.83–0.92)**	N/A	N/A
Patel 2020	OR (95% CI)	**Female sex = 0.83 (0.79-0.88)**	N/A	N/A
Ross 2020** (2nd)	OR (95% CI)	Female = **0.769 (0.733-0.807)**	N/A	N/A
Arroyo 2019 (2nd)	OR (95% CI)	Female = **0.74 (0.73-0.76)**	N/A	N/A
Liao 2016** (2nd)	Hazard ratio (p-value)	Sex = **0.64 (0.011)**<br><br>6.1% male patients readmitted vs 3.7% female patients readmitted	N/A	N/A
Tang 2019** (3rd)	OR (95% CI)	Bivariate logistic regression:<br>Female sex = 1.06 (0.52-2.15)	Chi-square for categorical variables and Mann-	Unless specified otherwise, results are presented as

		Multivariate logistic regression:<br>Female sex = 1.77 (0.68-4.63)	Whitney U test for continuous variables	proportion in complication (leading to readmission) group vs proportion in no complication group = p-value.<br><br>Sex (p-value = 0.88):<br>Male = 36.4% vs 37.7%<br>Female = 63.6% vs 62.3%
Male:				
Kurtz 2016 (1st)	F-statistic	Sex (F-statistic) = 1024<br><br>Female = 63.4% overall TKA cohort, 58.0% readmitted cohort<br>Male = 36.6% overall TKA cohort, 42.0% readmitted cohort	N/A	N/A
Courtney 2018*** (1st)	OR (95% CI)	Male sex = **1.456 (1.242-1.707)**	N/A	N/A
Ali 2019 (1st)	OR (95% CI)	Male sex = **1.23 (1.20-1.25)**	N/A	N/A
Pugely 2013 (1st)	OR (95% CI)	Male sex = **1.25 (1.03-1.53)**	N/A	N/A
Urish 2018 (1st)	OR (95% CI)	Male sex = **1.36 (1.30-1.42)**	N/A	N/A
Welsh 2017 (2nd)	OR (95% CI)	Male sex = **1.24 (1.22-1.27)**	N/A	N/A
Hart 2016 (2nd)	OR (95% CI)	Male sex = **1.48 (1.16-1.90)**	N/A	N/A
Sutton 2016 (2nd)	OR (95% CI)	Male sex = **1.52 (1.34-1.74)**	N/A	N/A
Runner 2017 (2nd)	OR (95% CI)	Male sex = **1.46 (1.19-1.78)**	N/A	N/A
Singh 2013 (3rd)	OR (95% CI)	Male sex = **1.25 (1.10-1.43)**	N/A	N/A
Yohe 2018 (3rd)	OR (95% CI)	Male sex = 1.050 (0.784-1.405)	N/A	N/A
Workman 2019 (3rd)	OR (95% CI)	Male sex = **1.372 (1.03-1.827)**	N/A	N/A
D'Apuzzo 2017 (3rd)	OR (95% CI)	Male sex = **1.26 (1.22-1.30)**	N/A	N/A
Siracuse 2017 (3rd)	OR (95% CI)	Male sex = **1.19 (1.16-1.23)**	N/A	N/A
Undefined reference category:				
Peskun 2012 (3rd)	OR (95% CI)	Gender = 1.4 (0.04-42.3)	N/A	N/A
Ramos 2014 (3rd)	OR (95% CI)	Sex = 1.454 (0.815-2.592)	N/A	N/A
Race and ethnicity				
Combined effect of race:				
Kurtz 2016 (1st)	F-statistic	Race (F-statistic) = 6.1	N/A	N/A

		Black = 5.2% overall TKA cohort, 6.3% readmitted cohort<br>Other/unknown = 3.7% overall TKA cohort, 3.7% readmitted cohort<br>White = 91.1% overall TKA cohort, 90.0% readmitted cohort		
Black:				
Ali 2019 (1st)	OR (95% CI)	Reference category = White<br><br>Black/Black British = 1.08 (0.99-1.19)	N/A	N/A
Lehtonen 2018 (1st)	OR (95% CI)	Reference category = White<br><br>Black = **1.24 (1.11- 1.37)**	N/A	N/A
Arroyo 2019 (2nd)	OR (95% CI)	Reference category = White<br><br>Black = **1.20 (1.15-1.25)**	N/A	N/A
Welsh 2017 (2nd)	OR (95% CI)	Reference category = White<br><br>Black race = **1.12 (1.07-1.18)**	N/A	N/A
Workman 2019 (3rd)	OR (95% CI)	Reference category unclear<br><br>Black ethnicity = 1.949 (0.639-5.94)	N/A	N/A
Siracuse 2017 (3rd)	OR (95% CI)	Reference category = White<br><br>African-American = **1.37 (1.30-1.44)**	N/A	N/A
D'Apuzzo 2017 (3rd)	OR (95% CI)	Reference category = White<br><br>Black = 0.99 (0.94-1.04)	N/A	N/A
Hispanic:				
Arroyo 2019 (2nd)	OR (95% CI)	Reference category = White<br><br>Hispanic = 1.01 (0.97-1.06)	N/A	N/A
Welsh 2017 (2nd)	OR (95% CI)	Reference category = White<br><br>Hispanic race = 0.99 (0.93-1.05)	N/A	N/A
Yohe 2018 (3rd)	OR (95% CI)	Reference category = non-Hispanic<br><br>Hispanic ethnicity = 0.413 (0.151-1.131)	N/A	N/A
D'Apuzzo 2017 (3rd)	OR (95% CI)	Reference category = White<br><br>Hispanic = **0.93 (0.87-0.99)**	N/A	N/A
Asian:				
Lehtonen 2018 (1st)	OR (95% CI)	Reference category = White<br><br>Asian **= 0.62 (0.48-0.80)**	N/A	N/A

Ali 2019 (1st)	OR (95% CI)	Reference category = White<br><br>Asian/Asian British = 1.01 (0.95-1.07)	N/A	N/A
D'Apuzzo 2017 (3rd)	OR (95% CI)	Reference category = White<br><br>Asian = **0.65 (0.56-0.75)**	N/A	N/A
Native Hawaiian:				
Lehtonen 2018 (1st)	OR (95% CI)	Reference category = White<br><br>Native Hawaiian = 0.58 (0.30 to 1.13)	N/A	N/A
American Indian:				
Lehtonen 2018 (1st)	OR (95% CI)	Reference category = White<br><br>American Indian = 0.60 (0.36 to 1.00; p = 0.0514))	N/A	N/A
White:				
Workman 2019 (3rd)	OR (95% CI)	Reference category = Non-white<br><br>White ethnicity = 0.859 (0.544-1.356)	N/A	N/A
Indian:				
Tang 2019** (3rd)	OR (95% CI)	Reference category = Chinese<br><br>Bivariate logistic regression:<br>Indian = 2.00 (0.62-6.53)	Chi-square for categorical variables and Mann-Whitney U test for continuous variables	Unless specified otherwise, results are presented as proportion in complication (leading to readmission) group vs proportion in no complication group = p-value.<br><br>Race (p-value = 0.70):<br>Chinese = 77.3% vs 83.8%<br>Indian = 11% vs 6.2%
Malay:				
Tang 2019** (3rd)	OR (95% CI)	Reference category = Chinese<br><br>Bivariate logistic regression:<br>Malay = 1 .28 (0.38-4.35)	Chi-square for categorical variables and Mann-Whitney U test for continuous variables	Unless specified otherwise, results are presented as proportion in complication (leading to readmission) group vs proportion in no complication group = p-value.<br><br>Race (p-value = 0.70):

				Chinese = 77.3% vs 83.8%<br>Malay = 9.1% vs 7.7%
Mixed race:				
Ali 2019 (1st)	OR (95% CI)	Reference category = White<br><br>Mixed ethnicity = 1.14 (0.94-1.37)	N/A	N/A
Other:				
Ali 2019 (1st)	OR (95% CI)	Reference category = White<br><br>Other ethnic group = 1.02 (0.90-1.17)	N/A	N/A
Abola 2018 (1st)	OR (99% CI)	Reference category = White<br><br>Non-white race = 1.05 (0.98–1.14)	N/A	N/A
Arroyo 2019 (2nd)	OR (95% CI)	Reference category = White<br><br>Other = **0.90 (0.85-0.95)**	N/A	N/A
Welsh 2017 (2nd)	OR (95% CI)	Reference category = White<br><br>Other race = 0.95 (0.88-1.03)	N/A	N/A
Siracuse 2017 (3rd)	OR (95% CI)	Reference category = White<br><br>Other = **1.08 (1.04-1.13)**	N/A	N/A
D'Apuzzo 2017 (3rd)	OR (95% CI)	Reference category = White<br><br>Other = **0.82 (0.76-0.88)**<br>Minority ethnicity = **1.293 (1.025-1.632)**	N/A	N/A
Tang 2019** (3rd)	OR (95% CI)	Reference category = Chinese<br><br>Bivariate logistic regression:<br>Others = 1.07 (0.11-10.61)	Chi-square for categorical variables and Mann-Whitney U test for continuous variables	Unless specified otherwise, results are presented as proportion in complication (leading to readmission) group vs proportion in no complication group = p-value.<br><br>Race (p-value = 0.70):<br>Chinese = 77.3% vs 83.8%<br>Others = 2.3% vs 2.3%
Missing:				
Ali 2019 (1st)	OR (95% CI)	Reference category = White<br><br>Not known or stated race = **0.68 (0.65-0.72)**	N/A	N/A

Lehtonen 2018 (1st)	OR (95% CI)	Reference category = White<br><br>Unknown or unreported = **0.87 (0.78-0.96)**	N/A	N/A
Arroyo 2019 (2nd)	OR (95% CI)	Reference category = White<br><br>Missing = **0.77 (0.70-0.84)**	N/A	N/A
D'Apuzzo 2017 (3rd)	OR (95% CI)	Reference category = White<br><br>Missing = **1.62 (1.52-1.72)**	N/A	N/A
Socioeconomic				
Income:				
Urish 2018 (1st)	OR (95% CI)	Household income (reference category = 37,999 or less):<br><br>38000-47999 = **0.9 (0.84-0.96)**<br>48000-63999 = **0.91 (0.85-0.97)**<br>64000+ = **0.85 (0.8-0.91)**	N/A	N/A
Ross 2020** (2nd)	OR (95% CI)	Income quintile (reference category = 5 (highest)):<br><br>**1 = 1.292 (1.199-1.392)**<br>**2 = 1.092 (1.013-1.177)**<br>3 = 1.054 (0.977-1.137)<br>4 = 1.072 (0.994-1.156)	N/A	N/A
Arroyo 2019 (2nd)	OR (95% CI)	Median income level (reference category = first):<br><br>Second quartile = **0.96 (0.93-1.00, p <= 0.05)**<br>Third quartile = **0.94 (0.91-0.98)**<br>Fourth quartile = **0.94 (0.91-0.98)**	N/A	N/A
Siracuse 2017 (3rd)	OR (95% CI)	Income quartile (reference category = 4th):<br><br>1st = 0.99 (0.95-1.04)<br>2nd = 0.98 (0.94-1.02)<br>3rd = 0.97 (0.94-1.01)	N/A	N/A
Insurance status:				
Urish 2018 (1st)	OR (95% CI)	Reference category = non-Medicare<br><br>Medicare = **1.27 (1.18-1.37)**	N/A	N/A
Arroyo 2019 (2nd)	OR (95% CI)	Payer status (reference category = private insurance):<br><br>Medicare = **1.23 (1.17-1.28)**<br>Medicaid = **1.58 (1.46-1.71)**<br>Self-pay or no charge = 1.10 (0.91-1.32)<br>Other = 1.03 (0.97-1.11)	N/A	N/A

Welsh 2017 (2nd)	OR (95% CI)	Disability entitlement = **1.41 (1.36-1.46)**	N/A	N/A
Siracuse 2017 (3rd)	OR (95% CI)	Primary payer (reference category = private insurance):<br><br>Medicare = **1.27 (1.22-1.32)**<br>Medicaid = **1.43 (1.33-1.54)**<br>Self-pay = **0.70 (0.52-0.93)**	N/A	N/A
D'Apuzzo 2017 (3rd)	OR (95% CI)	Insurance status (reference category = private insurance):<br><br>Medicare = **1.10 (1.05-1.15)**<br>Medicaid = 0.94 (0.87-1.02)<br>Workers' Compensation = **0.90 (0.83-0.98)**<br>Other = **0.86 (0.77-0.96)**	N/A	N/A
Socioeconomic status indices:				
Ali 2019 (1st)	OR (95% CI)	SES quintile (reference category = 1 (least deprived)):<br><br>2 = 1.02 (0.99-1.06)<br>3 = **1.05 (1.01-1.09)**<br>4 = **1.11 (1.07-1.15)**<br>5 = **1.18 (1.13-1.22)**	N/A	N/A
Functional status, living situation, and frailty				
Functional status:				
Jorgensen 2017 (1st)	OR (95% CI)	Use of walking aids = 1.37 (0.98–1.93)	N/A	N/A
Abola 2018 (1st)	OR (99% CI)	Functional dependence = **1.25 (1.07–1.46)**	N/A	N/A
Curtis 2019 (2nd)	OR (95% CI)	Dependent functional status (DEP) = **1.68 (1.39-2.02)**	N/A	N/A
Bovonratwet 2020 (2nd)	RR (95% CI)	Functional status prior to surgery (reference category = independent):<br>**Dependent = 6.4 (1.91-21.67)**	N/A	N/A
Yohe 2018 (3rd)	OR (95% CI)	Functional status partially or totally dependent = **3.231 (1.212-8.1613)**	N/A	N/A
Living situation:				
Jorgensen 2017 (1st)	OR (95% CI)	Reference category = living with spouse or relatives<br><br>Living alone = 1.02 (0.75–1.38)<br>Living in an institution = **6.00 (2.10–17.21)**	N/A	N/A
Ali 2019 (1st)	OR (95% CI)	Reference category not reported<br><br>Living alone = 1.00 (0.92-1.09; p-value = 0.9595)	N/A	N/A
Frailty:				
Runner 2017 (2nd)	OR (95% CI)	MFI = **8.71 (2.11-35.98)**	N/A	N/A
Miscellaneous				
Operative variables				
Elective or non-elective procedure:				

Sodhi and Anis et al 2019 (1st)	OR (95% CI)	Elective procedure = 0.846 (0.655-1.091)	N/A	N/A
Abola 2018 (1st)	OR (99% CI)	Nonelective operation = 0.96 (0.78–1.19)<br>Emergency operation = 1.18 (0.75–1.87)	N/A	N/A
Ross 2020** (2nd)	OR (95% CI)	Elective admission = **0.811 (0.682-0.965)**	N/A	N/A
Traumatic indication for TKA:				
Kester 2016 (1st)	OR (95% CI)	Post-traumatic TKA = **1.46 (1.02-2.08)**	N/A	N/A
Welsh 2017 (2nd)	OR (95% CI)	Traumatic admission type = 1.05 (1.00-1.10; p-value not reported)	N/A	N/A
Bilateral procedure:				
Hart 2016 (2nd)	OR (95% CI)	Bilateral TKA = 0.81 (0.58-1.12)	Fisher's exact test	Comparison of readmission rates:<br>Unilateral TKA (3.5%) vs bilateral TKA (3.6%): p-value = 0.89
Suleiman 2015 (2nd)	OR (95% CI)	SB TKA = 0.86 (0.41-1.81)	N/A	N/A
Welsh 2017 (2nd)	OR (95% CI)	Surgery type bilateral = **1.10 (1.03-1.16)**	N/A	N/A
D'Apuzzo 2017 (3rd)	OR (95% CI)	Bilateral TKA = **1.63 (1.55-1.71)**	N/A	N/A
Tang 2019** (3rd)	OR (95% CI)	Bivariate logistic regression:<br>Bilateral procedure = 1.11 (0.28-4.37)<br><br>Multivariate:<br>Bilateral procedure = 0.24 (0.03-1.80)	Chi-square for categorical variables and Mann-Whitney U test for continuous variables	Unless specified otherwise, results are presented as proportion in complication (leading to readmission) group vs proportion in no complication group = p-value.<br><br>Procedure type (p-value = 0.84):<br>Unilateral = 93.2% vs 93.1%<br>Bilateral = 6.8% vs 6.2%
Revision surgery:				
Courtney 2018*** (1st)	OR (95% CI)	TKA revision for infection = **1.455 (1.207-1.755)**	N/A	N/A
Ross 2020** (2nd)	OR (95% CI)	Revision index surgery = **1.424 (1.29-1.574)**	N/A	N/A
In-hospital complications				
Venous thromboembolism:				
Lehtonen 2018 (1st)	OR (95% CI)	Deep venous thrombosis = **10.32 (9.02 to 11.82)**<br>Pulmonary embolism = **16.45 (14.27 to 18.96)**	N/A	N/A

Belmont 2016*** (1st)	OR (95% CI)	DVT = **8.59 (2.36-31.24)**	N/A	N/A
Any medical or surgical complication:				
Runner 2017 (2nd)	OR (95% CI)	Any occurrence = **3.21 (2.63-3.92)**	N/A	N/A
D'Apuzzo 2017 (3rd)	OR (95% CI)	Medical = **1.67 (1.57-1.78)**<br>Surgical = **1.41 (1.30-1.53)**	N/A	N/A
Urinary tract infection (UTI):				
Belmont 2016*** (1st)	OR (95% CI)	UTI = **3.41 (1.04-11.22)**	N/A	N/A
Lehtonen 2018 (1st)	OR (95% CI)	Urinary tract infection = **6.22 (5.34 to 7.23)**	N/A	N/A
Surgical site infection:				
Lehtonen 2018 (1st)	OR (95% CI)	Superficial surgical site infection = **13.53 (11.46 to 15.98)**	N/A	N/A
Belmont 2016*** (1st)	OR (95% CI)	Deep wound infection/organ or space SSI combined = **15.09 (5.57-40.91)**<br>Superficial wound infection = **16.57 (5.82-47.22)**	N/A	N/A
Sepsis:				
Abola 2018 (1st)	OR (99% CI)	SIRS/sepsis/septic shock = 1.18 (0.76–1.81)	N/A	N/A
Cardiac:				
Lehtonen 2018 (1st)	OR (95% CI)	Cardiac arrest = **7.68 (5.00 to 11.81)**<br>Myocardial infarction = **18.48 (14.47 to 23.58)**	N/A	N/A
Pneumonia:				
Lehtonen 2018 (1st)	OR (95% CI)	Pneumonia = **12.15 (10.04 to 14.71)**	N/A	N/A
Acute renal failure:				
Lehtonen 2018 (1st)	OR (95% CI)	Acute renal failure = **15.26 (9.50 to 24.52)**	N/A	N/A
Healthcare utilisation				
Number of previous admissions:				
Ali 2019 (1st)	OR (95% CI)	Number of prior emergency admissions (0):<br>1 = **1.43 (1.38-1.48)**<br>2 = **1.78 (1.66-1.91)**<br>3+ = **2.38 (2.16-2.64)**	N/A	N/A
Welsh 2017 (2nd)	OR (95% CI)	Number of previous admissions = **1.24 (1.22-1.25)**	N/A	N/A
Number of prior knee procedures:				
Arroyo 2019 (2nd)	OR (95% CI)	Number of knee procedures = **0.76 (0.70-0.82)**	N/A	N/A
GP visit between surgery and readmission:				

Ross 2020** (2nd)	OR (95% CI)	Visit with GP from surgery to readmission = **0.636 (0.605-0.669)**	N/A	N/A
Patient-reported outcome measures (PROMs)				
Hospital Consumer Assessment of Healthcare Providers and Systems (HCAHPS) scores:				
Sodhi and Mont et al 2019** (3rd)	OR (95% CI)	Doctor explain = **0.29 (0.09-0.97)**<br>Discharge information = **0.034 (0.00-0.53)**<br>Good understanding = **0.28 (0.08-0.97)**<br>Doctor respect = 0.28 (0.07-1.14)<br>Definitely recommend = 0.35 (0.10-1.16)	N/A	N/A
Patient location				
Buitagro 2020 (3rd)	OR (95% CI)	Geographical region (reference = Bogota):<br>Atlantic = 0.84 (0.57-1.23)<br>Central = 1.09 (0.78-1.51)<br>Eastern = 0.94 (0.61-1.46)<br>Pacific = 0.86 (0.56-1.32)<br>**Other Departments = 2.74 (1.01-7.40)**	N/A	N/A
Most factors were found to be statistically significant (p-value often <0.0001) due to very large sample size, therefore type III F-statistic was reported. Type III F-statistic measures the additional reduction in error variance after all other factors had been included, thus reflecting the factor's independent contribution toward accounting for the variations in the dependent variable – it is valid for unbalanced data**<br><br>*Mixed cohort – revision and primary TKA combined**<br><br>*****Revision-only cohort**				

S10 - Results for all-cause readmission, unadjusted and secondary analyses

Comorbidities				
**Study ID (overall risk of bias quartile – arranged in descended order from lowest to highest)**	**Methods used**	**Unadjusted effect estimates**	**Univariate significance test**	**Additional analyses**
		**Result** (in **bold =** confidence intervals do not include null value**)**	**Result** (in **bold =** significant p-value**)**	**Result** (in **bold =** confidence intervals do not include null value or significant p-value (whichever applicable)**)**
Composite comorbidity indices				
Charlson Comorbidity Index (CCI):				
Mudumbai 2019* (4th)	Chi-square test comparing the readmitted patients (n=531 of 5514) with the overall cohort (n=5514), with two-sided p-value of ≤0.05 considered statistically significant	N/A	Overall % vs readmitted % (p-value) presented below:<br><br>CCI:<br>0 = 61.48 vs 54.43 **(<0.001)**<br>1 = 25.12 vs 26.74 **(<0.001)**<br>2+ = 13.40 vs 18.83 (0.393)	Multivariate, extended Cox regression analysis (Hazard Ratio (95% CI)) for readmission from postdischarge days 0 to 30 - all independent variables were included simultaneously in the model:<br><br>CCI:<br>1 = 1.06 (0.86-1.30)<br>2+ = 1.23 (0.97-1.57)<br>0 = reference range
American Society of Anaesthesiologists (ASA) Classification:				
Lehtonen 2018 (1st)	t-test to compare continuous variables; chi-square to compare categorical variables	N/A	Authors chose p<0.0001 to indicate statistical significance. Results are presented as non-readmitted group (%) vs readmitted group (%) = p-value<br><br>ASA class: **p<0.0001**<br>1: 2.1 vs 1.4<br>2: 50.6 vs 36.1<br>3: 45.6 vs 55.8	N/A

			4: 1.5 vs 3.3<br>5: 0.00 vs 0.00<br>Not assigned: 0.08 vs 0.11	
Belmont 2016** (1st)	Bivariate logistic regression (OR (95% CI))	Reference category = 1-2<br><br>ASA class ≥3 = **1.69 (1.12-2.56)**	N/A	N/A
Schaeffer 2015 (4th)	Chi-square test	N/A	ASA 3-4 vs ASA 1-2 (p = 0.2914)	N/A
Jauregui 2015 (4th)	Z-test – p<0.05 considered to be statistically significant	N/A	Within NSQIP, the following factors were associated with increased risk of readmission (only the p-values were reported in the paper, and not the readmission rates):<br>ASA class 3 **(p = 0.001)**	N/A
Presence of any comorbidity:				
Urish 2018 (1st)	t test for parametric continuous variables; Wilcoxon rank-sum test for nonparametric continuous variables; chi-square test for proportions.	N/A	**All of the following were significantly different (at p <0.05)** between readmitted and non-readmitted patients: Comorbidities	N/A
Belmont 2016** (1st)	Bivariate logistic regression (OR (95% CI))	Total number of comorbidities:<br>1 vs 0 = 1.37 (0.88-2.13)<br>2 vs 0 = 1.55 (0.85-2.81)<br>3 vs 0 = 2.25 (0.92-5.49)<br>≥4 vs 0 = 0.97 (0.13-7.36)	N/A	N/A
Elixhauser Index:				
Arroyo 2019 (2nd)	Continuous variables analysed using analysis of variance; categorical variables analysed using Pearson chi-square test or Fisher exact test.	N/A	**Achieved statistical significance with p<0.001:**<br>Elixhauser Index - the Van Walraven Score: Median (Q1; Q3) = 0 (-1; 2) readmitted vs 0 (-1; 0) non-readmitted	See section S10 - Arroyo 2019 – Results from Exploratory Stratified Analysis’
Diagnosis-related groups (DRGs):				

Mudumbai 2019* (4th)	Chi-square test comparing the readmitted patients (n=531 of 5514) with the overall cohort (n=5514), with two-sided p-value of ≤0.05 considered statistically significant		Overall % vs readmitted % (p-value) presented below:<br><br>DRG weight:<br>≤2 = 3.75 vs 1.69 **(0.012)**<br>2-3 = 91.78 vs 90.21 (0.190)<br>3+ = 4.46 vs 8.10 **(<0.001)**	Multivariate, extended Cox regression analysis (Hazard Ratio (95% CI)) for readmission from postdischarge days 0 to 30 - all independent variables were included simultaneously in the model:<br><br>DRG weight:<br>0-2 = **0.23 (0.11-0.47)**<br>2-3 = **0.64 (0.45-0.91)**<br>3+ = reference range
Cardiovascular				
Hypertension:				
Lehtonen 2018 (1st)	t-test to compare continuous variables; chi-square to compare categorical variables	N/A	Authors chose p<0.0001 to indicate statistical significance. Results are presented as non-readmitted group (%) vs readmitted group (%) = p-value<br><br>Comorbidities:<br>Hypertension: 65.2 vs 74.1 = **p<0.0001**	
Belmont 2016** (1st)	Bivariate logistic regression (OR (95% CI))	Hypertension = 0.68 (0.46-1.01)	N/A	N/A
Liao 2016* (2nd)	Chi-square test – statistical significance was indicated by two-sided p-value of 0.05	N/A	Not statistically significant on univariate analysis (% readmission rate; individual p-values not reported):<br>Hypertension: yes = 4.1 vs no = 4.7	
Arroyo 2019 (2nd)	Continuous variables analysed using analysis of variance; categorical variables analysed using Pearson chi-square test or Fisher exact test.	N/A	**Achieved statistical significance with p<0.001:**<br>Hypertension (uncomplicated)<br>Hypertension (complicated)	See section S10 - Arroyo 2019 – Results from Exploratory Stratified Analysis
Workman 2019 (3rd)	Student's t test for continuous variables; Fisher's exact test for categorical variables	N/A	Hypertension not otherwise specified (0.9163)	N/A

			Primary hypertension (0.0890)	
Siracuse 2017 (3rd)	Chi-square test; Student t tests; binary logistic regression	Hypertension = **1.31 (1.27-1.35)**	Not reported	N/A
Jauregui 2015 (4th)	Z-test – $p<0.05$ considered to be statistically significant	N/A	Within NSQIP, the following factors were associated with increased risk of readmission (only the p-values were reported in the paper, and not the readmission rates):<br>Hypertension requiring medication **(p = 0.001)**	N/A
Hyperlipidaemia:				
Workman 2019 (3rd)	Student’s t test for continuous variables; Fisher’s exact test for categorical variables	N/A	Hyperlipidemia (0.1457)	N/A
Pugely 2013 (1st)	Student’s t-test for continuous variables; chi-square test for categorical variables	N/A	Statistical significance was set at $p < 0.05$. Values presented as: variable (p-value) = non-readmitted proportion (%) vs readmitted proportion (%)<br><br>Hypertension **(0.0005)** = 66.62 vs 73.85	N/A
Coronary artery disease (CAD), cardiovascular disease (CVD) and ischaemic heart disease (IHD):				
Liao 2016* (2nd)	Chi-square test – statistical significance was indicated by two-sided p-value of 0.05	N/A	Not statistically significant on univariate analysis (% readmission rate; individual p-values not reported):<br>CVD: yes = 7.3 vs no = 4	
Workman 2019 (3rd)	Student’s t test for continuous variables; Fisher’s exact test for categorical variables	N/A	Coronary atherosclerosis **(0.0249)**	N/A
Congestive Cardiac/Heart Failure (CCF/CHF):				

Pugely 2013 (1st)	Student's t-test for continuous variables; chi-square test for categorical variables	N/A	Statistical significance was set at $p < 0.05$. Values presented as: variable (p-value) = non-readmitted proportion (%) vs readmitted proportion (%)<br><br>CHF **(0.0181)** = 0.22 vs 0.74	N/A
Lehtonen 2018 (1st)	t-test to compare continuous variables; chi-square to compare categorical variables	N/A	Authors chose p<0.0001 to indicate statistical significance. Results are presented as non-readmitted group (%) vs readmitted group (%) = p-value<br><br>Comorbidities:<br>CHF: 0.3 vs 0.9 = **p<0.0001**	N/A
Belmont 2016** (1st)	Bivariate logistic regression (OR (95% CI))	CHF = 3.09 (0.67-14.27)	N/A	N/A
Arroyo 2019 (2nd)	Continuous variables analysed using analysis of variance; categorical variables analysed using Pearson chi-square test or Fisher exact test.	N/A	**Achieved statistical significance with p<0.001:**<br>Congestive heart failure	See section S10 - Arroyo 2019 – Results from Exploratory Stratified Analysis
Siracuse 2017 (3rd)	Chi-square test; Student t tests; binary logistic regression	Congestive heart failure = **2.27 (2.13-2.43)**	Not reported	N/A
Arrhythmias:				
Workman 2019 (3rd)	Student's t test for continuous variables; Fisher's exact test for categorical variables	N/A	Atrial fibrillation **(0.0263)**	N/A
Valvular disease:				
Arroyo 2019 (2nd)	Continuous variables analysed using analysis of variance; categorical variables analysed using	N/A	**Achieved statistical significance with p<0.001:**<br>Valvular disease	See section S10 - Arroyo 2019 – Results from Exploratory Stratified Analysis

	Pearson chi-square test or Fisher exact test.			
Peripheral vascular disease:				
Pugely 2013 (1st)	Student's t-test for continuous variables; chi-square test for categorical variables	N/A	Statistical significance was set at p < 0.05. Values presented as: variable (p-value) = non-readmitted proportion (%) vs readmitted proportion (%)<br><br>PVD (0.086) = 0.46 vs 1.46	N/A
Arroyo 2019 (2nd)	Continuous variables analysed using analysis of variance; categorical variables analysed using Pearson chi-square test or Fisher exact test.	N/A	**Achieved statistical significance with p<0.001:**<br>Peripheral vascular disorders	See section S10 - Arroyo 2019 – Results from Exploratory Stratified Analysis
Cardiac disease:				
Belmont 2016** (1st)	Bivariate logistic regression (OR (95% CI))	Cardiac disease = 1.68 (0.79-3.57)<br>Previous myocardial infarction = N/A<br>History of angina within 1 month = 7.68 (0.69-85.41)	N/A	N/A
History of percutaneous coronary intervention or cardiac surgery:				
Belmont 2016** (1st)	Bivariate logistic regression (OR (95% CI))	PCI = 1.62 (0.63-4.15)<br>Previous cardiac surgery = 1.39 (0.32-6.01)<br>History revascularisation/amputation for peripheral vascular disease/rest pain/gangrene = 2.17 (0.07-66.60)	N/A	N/A
Jauregui 2015 (4th)	Z-test – p<0.05 considered to be statistically significant	N/A	Within NSQIP, the following factors were associated with increased risk of readmission (only the p-values were reported in the paper, and not the readmission rates):<br>History of percutaneous coronary intervention or cardiac surgery **(p = 0.0001)**	N/A
BMI, obesity, and weight loss				

BMI continuous:				
Belmont 2016** (1st)	Bivariate logistic regression (OR (95% CI))	BMI (continuous) = 1.02 (0.99-1.04)	N/A	N/A
Kheir 2014 (4th)	Bivariate logistic regression analysis for unadjusted effect estimates<br><br>Categorical variables compared using chi-square test; continuous non-parametric variables compared using Mann-Whitney U-test	OR (95%)<br><br>BMI (continuous) = 1.01 (0.99-1.03)	Results are presented as non-readmitted group (% or mean (SD)) vs readmitted group (% or mean (SD)) = p-value. P-value <0.05 was considered statistically significant.<br><br>BMI: 32.7 (7.6) vs 33.4 (8.2) = 0.329	N/A
BMI categories:				
Pugely 2013 (1st)	Student's t-test for continuous variables; chi-square test for categorical variables	N/A	Statistical significance was set at p < 0.05. Values presented as: variable (p-value) = non-readmitted proportion (%) vs readmitted proportion (%)<br><br>BMI (0.7761):<br><35 = 68.42 vs 69.00<br>>35 = 31.58 vs 31.00	N/A
Lehtonen 2018 (1st)	t-test to compare continuous variables; chi-square to compare categorical variables	N/A	Authors chose p<0.0001 to indicate statistical significance. Results are presented as non-readmitted group (%) vs readmitted group (%) = p-value<br><br>BMI category: = **p<0.0001**<br><18.5: 0.2 vs 0.2<br>18.5-25: 9.8 vs 10.0<br>25-30: 27.0 vs 26.0<br>30-35: 28.6 vs 26.8<br>35-40: 19.2 vs 17.7<br>≥40: 15.3 vs 19.2	N/A

Belmont 2016** (1st)	Bivariate logistic regression (OR (95% CI))	BMI 30.0-39.9 (reference <30) = 1.03 (0.67-1.59)<br>BMI ≥40 (reference <30) = 1.49 (0.87-2.55)	N/A	N/A
George 2018 (2nd)	Chi-square; logistic regression	Overweight = 0.93 (0.84-1.03)<br>Obese = 0.9 (0.82-1; p = 0.051)<br>Morbidly obese = **1.2 (1.07-1.33)**	Readmission rates differed significantly between the BMI categories:<br>Normal weight (3.54%) vs overweight (3.32%) vs obese (3.23%) vs morbidly obese (4.23%) = **p<0.001**	N/A
Zusmanovic 2018 (2nd)	Fisher exact test	N/A	O2 vs O3 **(p < 0.001)**<br>O1 vs O2 (p = 0.685)	N/A
Sloan 2020 (2nd)	Chi-square	N/A	Readmission were more common<br>among patients categorized as Obesity Class III (4.15%<br>(n = 670)) compared with Normal Weight (3.59% (n = 337),<br>p < 0.001)	From the restricted model (adjusted only for preoperative continuous albumin level):<br>Underweight = 1.07 (0.52-2.19)<br>Overweight = 0.95 (0.84-1.08)<br>Obese I = 0.94 (0.82-1.06)<br>Obese II = **0.85 (0.74-0.97)**<br>Obese III = 1.11 (0.97-1.27)
Kheir 2014 (4th)	Bivariate logistic regression analysis for unadjusted effect estimates	OR (95%)<br><br>BMI (categorical – reference = <25):<br>25 to <30 = 0.7 (0.41-1.21)<br>30 to <35 = 1.08 (0.64-1.81)<br>≥35 = 1.00 (0.61-1.65)	N/A	N/A
Obesity:				
Roth 2019** (1st)	Chi-square test was used to compare readmission rates between BMI categories; univariate logistic regression was used to obtain an effect size between these categories	Morbidly obese = **2.06 (1.33-2.96)**<br>Obese = 1.38 (0.99-1.94)<br>Overweight = 1.22 (0.85-1.75)	Readmission rates increased with increased BMI **(p<0.001)**:<br>Normal weight = 4.0%<br>Overweight = 4.9%<br>Obese = 5.5%<br>Morbidly obese = 8.1%	Readmission rate increased linearly with increasing BMI when measured as a continuous variable using restricted cubic spline analysis with four knots in the logistic regression model **(see Figure 1 in the study)**
Arroyo 2019 (2nd)	Continuous variables analysed using analysis of variance; categorical variables analysed using Pearson chi-square test or Fisher exact test.	N/A	**Achieved statistical significance with p<0.001:**<br>Obesity	See section S10 - Arroyo 2019 – Results from Exploratory Stratified Analysis

Workman 2019 (3rd)	Student's t test for continuous variables; Fisher's exact test for categorical variables	N/A	Obesity/morbid obesity **(0.0301**	N/A
Siracuse 2017 (3rd)	Chi-square test; Student t tests; binary logistic regression	Obesity = 1.00 (0.97-1.04)	Not reported	N/A
Hanly 2017 (4th)	Chi-square, p-value for significance = 0.0166 according to Bonferroni's correction	N/A	Morbidly obese (14.5%) vs normal weight (8.5%): p-value = 0.179	N/A
Weight loss:				
Pugely 2013 (1st)	Student's t-test for continuous variables; chi-square test for categorical variables	N/A	Statistical significance was set at p < 0.05. Values presented as: variable (p-value) = non-readmitted proportion (%) vs readmitted proportion (%)<br><br>Recent Wt Loss (0.5895) = 0.25 vs 0.37	N/A
Lehtonen 2018 (1st)	t-test to compare continuous variables; chi-square to compare categorical variables	N/A	Authors chose p<0.0001 to indicate statistical significance. Results are presented as non-readmitted group (%) vs readmitted group (%) = p-value<br><br>Recent weight loss: 0.1 vs 0.1 = 0.711	N/A
Belmont 2016** (1st)	Bivariate logistic regression (OR (95% CI))	Comorbidities:<br>Recent weight loss = **5.16 (1.03-25.86)**	N/A	N/A
Endocrine				
Diabetes:				
Pugely 2013 (1st)	Student's t-test for continuous variables; chi-square test for categorical variables	N/A	Statistical significance was set at p < 0.05. Values presented as: variable (p-value) = non-readmitted proportion (%) vs readmitted proportion (%)	N/A

			Diabetes **(0.0073)** = 17.25 vs 21.73	
Lehtonen 2018 (1st)	t-test to compare continuous variables; chi-square to compare categorical variables	N/A	Authors chose p<0.0001 to indicate statistical significance. Results are presented as non-readmitted group (%) vs readmitted group (%) = p-value<br><br>Comorbidities:<br>Diabetes: **p<0.0001**<br>Insulin-dependent diabetes: 4.4 vs 7.4<br>Non-insulin-dependent diabetes: 3.5 vs 15.1	N/A
Belmont 2016** (1st)	Bivariate logistic regression (OR (95% CI))	All diabetes = 1.10 (0.70-1.75)<br>IDDM = 0.88 (0.40-1.94)<br>NIDDM = 1.21 (0.72-2.05)	N/A	N/A
Liao 2016* (2nd)	Chi-square test – statistical significance was indicated by two-sided p-value of 0.05	N/A	Statistically significant on univariate analysis (% readmission rate; individual p-values not reported):<br>DM: **yes = 6 vs no = 3.7**	Sensitivity analysis (HR (p-value)).<br><br>Age <75y group:<br>DM = **1.69 (0.019)**<br><br>Age > 80y group:<br>DM = **1.66 (0.019)**
Lovecchio 2014 (2nd)	Logistic regression for unadjusted effect estimate, chi-square for univariate significance test	NIDDM = 1.14 (0.83-1.57)<br>IDDM = **1.83 (1.18-2.81)**	4.1% no diabetes, 4.6% NIDDM, 7.2% IDDM: **p = 0.003**	N/A
Arroyo 2019 (2nd)	Continuous variables analysed using analysis of variance; categorical variables analysed using Pearson chi-square test or Fisher exact test.	N/A	**Achieved statistical significance with p<0.001:**<br>Diabetes (uncomplicated)<br>Diabetes (complicated)	See section S10 - Arroyo 2019 – Results from Exploratory Stratified Analysis
Workman 2019 (3rd)	Student's t test for continuous variables; Fisher's exact test for categorical variables	N/A	Diabetes (0.5714)	N/A

Siracuse 2017 (3rd)	Chi-square test; Student t tests; binary logistic regression	Diabetes (includes complicated and uncomplicated diabetes) = **1.32 (1.28-1.36)**	Not reported	N/A
Hypothyroidism:				
Workman 2019 (3rd)	Student's t test for continuous variables; Fisher's exact test for categorical variables	N/A	Hypothyroid (0.7838)	N/A
Gastrointestinal				
Gastroesophageal reflux:				
Workman 2019 (3rd)	Student's t test for continuous variables; Fisher's exact test for categorical variables	N/A	Gastroesophageal reflux (0.2945)	N/A
Liver disease:				
Ali 2019 (1st)	N/A	N/A	N/A	Population attributable fraction was calculated for each of the strongest predictors for readmission. This is the proportion of the incidence rate in the whole population that is due to have that particular comorbidity.<br><br>Liver disease = 0.3%
Arroyo 2019 (2nd)	Continuous variables analysed using analysis of variance; categorical variables analysed using Pearson chi-square test or Fisher exact test.	N/A	**Achieved statistical significance with p<0.001:**<br>Liver disease	See section S10 - Arroyo 2019 – Results from Exploratory Stratified Analysis
Siracuse 2017 (3rd)	Chi-square test; Student t tests; binary logistic regression	Liver disease = **1.34 (1.19-1.50)**	Not reported	N/A
Haematological				
Anaemia:				
Arroyo 2019 (2nd)	Continuous variables analysed using analysis of variance; categorical variables analysed using Pearson chi-square test or Fisher exact test.	N/A	**Achieved statistical significance with p<0.001:**<br>Blood loss anemia<br>Deficiency anemia	See section S10 - Arroyo 2019 – Results from Exploratory Stratified Analysis
Workman 2019 (3rd)	Student's t test for continuous variables;	N/A	Anaemia **(0.0285)**	N/A

	Fisher's exact test for categorical variables			
Siracuse 2017 (3rd)	Chi-square test; Student t tests; binary logistic regression	Anaemia = **1.35 (1.31-1.40)**	Not reported	N/A
Bleeding disorders:				
Pugely 2013 (1st)	Student's t-test for continuous variables; chi-square test for categorical variables	N/A	Statistical significance was set at p < 0.05. Values presented as: variable (p-value) = non-readmitted proportion (%) vs readmitted proportion (%)<br><br>Bleeding Disorder **(<.0001)** = 2.49 vs 6.45<br>INR **(0.0326)** = 1.04 vs 1.06	N/A
Lehtonen 2018 (1st)	t-test to compare continuous variables; chi-square to compare categorical variables	N/A	Authors chose p<0.0001 to indicate statistical significance. Results are presented as non-readmitted group (%) vs readmitted group (%) = p-value<br><br>Comorbidities:<br>Bleeding disorder: 2.3 vs 5.2 = **p<0.0001**<br>Preoperative lab values:<br>INR: 1.02 (0.52-1.52) non-readmitted group vs 1.06 (0.56-1.56) readmitted = **p<0.0001**	N/A
Belmont 2016** (1st)	Bivariate logistic regression (OR (95% CI))	Bleeding disorder = 1.56 (0.70-3.48)<br>INR = 1.90 (0.96-3.77)	N/A	N/A
Ali 2019 (1st)	N/A	N/A	N/A	Population attributable fraction was calculated for each of the strongest predictors for readmission. This is the proportion of the incidence rate in the whole population that is due to have that particular comorbidity.<br><br>Coagulopathy = 0.2%

Arroyo 2019 (2nd)	Continuous variables analysed using analysis of variance; categorical variables analysed using Pearson chi-square test or Fisher exact test.	N/A	**Achieved statistical significance with p<0.001:**<br>Coagulopathy	See section S10 - Arroyo 2019 – Results from Exploratory Stratified Analysis
Rudasill 2019 (2nd)	Chi-square	N/A	Any readmission (increases with increasing INR class: **p <0.001**)	N/A
Siracuse 2017 (3rd)	Chi-square test; Student t tests; binary logistic regression	Coagulopathy = **1.54 (1.41-1.68)**	Not reported	N/A
Jauregui 2015 (4th)	Z-test – p<0.05 considered to be statistically significant	N/A	Within NSQIP, the following factors were associated with increased risk of readmission (only the p-values were reported in the paper, and not the readmission rates):<br>Bleeding disorders **(p 0.001)**	N/A
Blood dyscrazias:				
Pugely 2013 (1st)	Student's t-test for continuous variables; chi-square test for categorical variables	N/A	Statistical significance was set at p < 0.05. Values presented as: variable (p-value) = non-readmitted proportion (Mean (SD)) vs readmitted proportion (Mean (SD))<br><br>Sodium (0.2426) = 139.4 vs 139.3<br>BUN **(<.0001)** = 18.21 vs 20.18<br>Albumin **(0.04)** = 4.09 vs 4.04<br>WBC (0.1898) = 7.06 vs 7.18<br>HCT (0.7169) = 40.49 vs 40.42	N/A

			Platelets **(0.0389)** = 246.0 vs 238.9<br>Creatinine **(0.0005)** = 0.92 vs 1.02	
Abola 2018 (1st)	N/A	N/A	N/A	Sensitivity analyses on account of missing data (for the effect of hyponatraemia):<br><br>Complete case analysis = **1.26 (1.01–1.59)**<br><br>Sensitivity analysis assuming all missing cases had hyponatraemia = 1.05 (0.97–1.13)<br><br>Sensitivity analysis assuming all missing cases had normonatraemia = **1.12 (1.01–1.25)**
Lehtonen 2018 (1st)	t-test to compare continuous variables; chi-square to compare categorical variables	N/A	Authors chose p<0.0001 to indicate statistical significance. Results are presented as non-readmitted group (%) vs readmitted group (%) = p-value<br><br>Comorbidities:<br><br>Preoperative lab values:<br>WBC count: 7.05 (2.75-11.35) non-readmitted group vs 7.26 (1.64-12.88) readmitted group = **p<0.0001**<br>Haematocrit: 40.82 (32.74-48.9) non-readmitted group vs 40.29 (31.51-49.07) readmitted group = **p<0.0001**<br>Platelets: 244.12 (111.52-376.72) non-readmitted group vs 239.44 (97.78-381.1) readmitted group = **p<0.0001**<br>Creatinine: 0.91 (0.11-1.71) non-readmitted	N/A

			group vs 1.01 (9.21-1.81) readmitted group = **p<0.0001**<br>Serum albumin: 4.10 (3.34-4.86) non-readmitted group vs 4.02 (3.2-4.84) readmitted group = **p<0.0001**	
Belmont 2016** (1st)	Bivariate logistic regression (OR (95% CI))	Preoperative laboratory values (continuous unless specified otherwise):<br>White blood cell count = 1.02 (0.96-1.09)<br>Haematocrit = 0.99 (0.95-1.03)<br>Platelets = 0.999 (0.996-1.002)<br>Creatinine = 1.16 (0.92-1.45)<br>Creatinine ≥2 g/dL = 1.08 (0.25-4.61)<br>Serum albumin = 0.61 (0.40-0.94)<br>Prealbumin ≤3.5 g/dL = 1.56 (0.86-2.82)	N/A	N/A
Arroyo 2019 (2nd)	Continuous variables analysed using analysis of variance; categorical variables analysed using Pearson chi-square test or Fisher exact test.	N/A	**Achieved statistical significance with p<0.001:**<br>Fluid and electrolyte disorders	See section S10 - Arroyo 2019 – Results from Exploratory Stratified Analysis
Sloan 2020 (2nd)	Chi-square	N/A	Readmission were higher among patients with hypoalbuminaemia: 6.10% (n = 254) readmitted vs 3.34% (n=3254) non-readmitted = p<0.001	From the restricted model (adjusted only for preoperative continuous albumin level):<br>Normal albumin (and underweight) = 0.84 (0.34-2.06)<br>Hypoalbuminaemia (and underweight) = 1.96 (0.55-6.95)<br><br>Normal albumin (and overweight) = 0.95 (0.83-1.08)<br>Hypoalbuminaemia (and overweight) = 0.96 (0.59-1.54)<br><br>Normal Albumin (and obese I) = 0.93 (0.81-1.06)<br>Hypoalbuminaemia (and obese I) = 1.09 (0.68-1.74)<br><br>Normal albumin (and obese II) = **0.85 (0.74-0.98)**

				Hypoalbuminaemia (and obese II) = 1.00 (0.62-1.62)<br><br>Normal albumin (and obese III) = 1.18 (1.03-1.36)<br>Hypoalbuminaemia (and obese III) = 0.85 (0.53-1.35)
Siracuse 2017 (3rd)	Chi-square test; Student t tests; binary logistic regression	Fluid and electrolyte disorder = **1.50 (1.43-1.57)**	Not reported	N/A
Respiratory				
COPD and chronic airways disease:				
Lehtonen 2018 (1st)	t-test to compare continuous variables; chi-square to compare categorical variables	N/A	Authors chose p<0.0001 to indicate statistical significance. Results are presented as non-readmitted group (%) vs readmitted group (%) = p-value<br><br>Comorbidities:<br>COPD: 3.5 vs 8.0 = **p<0.0001**	N/A
Belmont 2016** (1st)	Bivariate logistic regression (OR (95% CI))	COPD = 1.68 (0.79-3.57)	N/A	N/A
Pugely 2013 (1st)	Student's t-test for continuous variables; chi-square test for categorical variables	N/A	Statistical significance was set at p < 0.05. Values presented as: variable (p-value) = non-readmitted proportion (%) vs readmitted proportion (%)<br><br>COPD **(0.013)** = 3.35 vs 5.34	N/A
Liao 2016* (2nd)	Chi-square test – statistical significance was indicated by two-sided p-value of 0.05	N/A	Statistically significant on univariate analysis (% readmission rate; individual p-values not reported):<br>COPD: **yes = 7.0 vs no = 4.0**	Sensitivity analysis (HR (p-value)).<br><br>Age <75y group:<br>COPD = **2.10 (0.011)**<br><br>Age > 80y group:<br>COPD = **1.72 (0.027)**

Workman 2019 (3rd)	Student's t test for continuous variables; Fisher's exact test for categorical variables	N/A	Chronic airway obstructive disorder **(0.0002)**	N/A
Siracuse 2017 (3rd)	Chi-square test; Student t tests; binary logistic regression	Chronic obstructive pulmonary disease = **1.34 (1.30-1.39)**	Not reported	N/A
Jauregui 2015 (4th)	Z-test – $p<0.05$ considered to be statistically significant	N/A	Within NSQIP, the following factors were associated with increased risk of readmission (only the p-values were reported in the paper, and not the readmission rates):<br>COPD **(p = 0.01)**	N/A
Pulmonary disease:				
Arroyo 2019 (2nd)	Continuous variables analysed using analysis of variance; categorical variables analysed using Pearson chi-square test or Fisher exact test.	N/A	**Achieved statistical significance with p<0.001:**<br>Chronic pulmonary disease	See section S10 - Arroyo 2019 – Results from Exploratory Stratified Analysis
Smoking:				
Lehtonen 2018 (1st)	t-test to compare continuous variables; chi-square to compare categorical variables	N/A	Authors chose p<0.0001 to indicate statistical significance. Results are presented as non-readmitted group (%) vs readmitted group (%) = p-value<br><br>Comorbidities:<br>Smoking: 8.6 vs 10.3 = **p<0.0001**	N/A
Pugely 2013 (1st)	Student's t-test for continuous variables; chi-square test for categorical variables	N/A	Statistical significance was set at $p < 0.05$. Values presented as: variable (p-value) = non-readmitted proportion (%) vs readmitted proportion (%)	N/A

			Current Smoker (0.1934) = 8.19 vs 9.76	
Belmont 2016** (1st)	Bivariate logistic regression (OR (95% CI))	Comorbidities:<br>Smoking = 1.12 (0.60-2.09)	N/A	N/A
Workman 2019 (3rd)	Student's t test for continuous variables; Fisher's exact test for categorical variables	N/A	History of tobacco use (0.6726)	N/A
Jorgensen 2013* (3rd)	N/A	N/A	N/A	Data were obtained upon request from the corresponding author and OR (95% CI) were calculated using Medcalc (https://www.medcalc.org/calc/odds_ratio.php):<br><br>Smoking = 1.53 (0.99-2.36)
Mudumbai 2019* (4th)	Chi-square test comparing the readmitted patients (n=531 of 5514) with the overall cohort (n=5514), with two-sided p-value of ≤0.05 considered statistically significant		Overall % vs readmitted % (p-value) presented below:<br><br>Diagnoses in the 365 d before admission:<br>nicotine = 9.94 vs 10.55 (0.677)	Multivariate, extended Cox regression analysis (Hazard Ratio (95% CI)) for readmission from postdischarge days 0 to 30 - all independent variables were included simultaneously in the model:<br><br>Diagnoses in the 365d before admission:<br>nicotine = 0.98 (0.73-1.31)
Asthma:				
Workman 2019 (3rd)	Student's t test for continuous variables; Fisher's exact test for categorical variables	N/A	Asthma (0.7842)	N/A
Dyspnoea:				
Pugely 2013 (1st)	Student's t-test for continuous variables; chi-square test for categorical variables	N/A	Statistical significance was set at p < 0.05. Values presented as: variable (p-value) = non-readmitted proportion (%) vs readmitted proportion (%)<br><br>Dyspnoea (0.1044) = 7.50 vs 9.39	N/A
Lehtonen 2018 (1st)	t-test to compare continuous variables; chi-square to compare categorical variables	N/A	Authors chose p<0.0001 to indicate statistical significance. Results are presented as non-	N/A

			readmitted group (%) vs readmitted group (%) = p-value<br><br>Comorbidities:<br>Dyspnoea: 5.9 vs 9.8 = **p<0.0001**	
Belmont 2016** (1st)	Bivariate logistic regression (OR (95% CI))	Dyspnoea = 1.01 (0.50-2.04)	N/A	N/A
Obstructive sleep apnoea:				
Workman 2019 (3rd)	Student's t test for continuous variables; Fisher's exact test for categorical variables	N/A	Obstructive sleep apnea (0.5485)	N/A
Cardiopulmonary disease				
Jorgensen 2013* (3rd)	N/A	N/A	N/A	Data were obtained upon request from the corresponding author and OR (95% CI) were calculated using Medcalc (https://www.medcalc.org/calc/odds_ratio.php):<br><br>**Cardiopulmonary disease = 1.83 (1.15-2.92)**
Psychiatric				
Depression and mental health disorder:				
Ali 2019 (1st)	N/A	N/A	N/A	Population attributable fraction was calculated for each of the strongest predictors for readmission. This is the proportion of the incidence rate in the whole population that is due to have that particular comorbidity.<br><br>Other mental health disorder = 0.3%
Arroyo 2019 (2nd)	Continuous variables analysed using analysis of variance; categorical variables analysed using Pearson chi-square test or Fisher exact test.	N/A	**Achieved statistical significance with p<0.001:**<br>Depression	See section S10 - Arroyo 2019 – Results from Exploratory Stratified Analysis
Workman 2019 (3rd)	Student's t test for continuous variables; Fisher's exact test for categorical variables	N/A	Depressive Disorder (0.9369)<br>Anxiety disorder (0.3434)	N/A

Mudumbai 2019* (4th)	Chi-square test comparing the readmitted patients (n=531 of 5514) with the overall cohort (n=5514), with two-sided p-value of ≤0.05 considered statistically significant	N/A	Overall % vs readmitted % (p-value) presented below:<br><br>Diagnoses in the 365 d before admission:<br>bipolar disorder = 2.29 vs 3.20 (0.182)<br>major depression = 7.18 vs 7.34 (0.949)<br>PTSD = 14.25 vs 18.83 **(0.002)**<br>generalised anxiety disorder = 1.27 vs 1.51 (0.757)	Multivariate, extended Cox regression analysis (Hazard Ratio (95% CI)) for readmission from postdischarge days 0 to 30 - all independent variables were included simultaneously in the model:<br><br>Diagnoses in the 365d before admission:<br>bipolar disorder = 1.03 (0.62-1.71)<br>major depression = 0.84 (0.59-1.19)<br>PTSD = 1.26 (0.99-1.61)<br>generalised anxiety disorder = 1.07 (0.52-2.17)
Substance use:				
Belmont 2016** (1st)	Bivariate logistic regression (OR (95% CI))	Regular alcohol use = 0.46 (0.03-8.33)	N/A	N/A
Pugely 2013 (1st)	Student's t-test for continuous variables; chi-square test for categorical variables	N/A	Statistical significance was set at p < 0.05. Values presented as: variable (p-value) = non-readmitted proportion (%) vs readmitted proportion (%)<br><br>Current Alcohol Abuse (0.4376) = 2.86 vs 1.94	N/A
Arroyo 2019 (2nd)	Continuous variables analysed using analysis of variance; categorical variables analysed using Pearson chi-square test or Fisher exact test.	N/A	**Achieved statistical significance with p<0.001:**<br>Alcohol abuse<br>Drug abuse	See section S10 - Arroyo 2019 – Results from Exploratory Stratified Analysis
Jorgensen 2013* (3rd)	N/A	N/A	N/A	Data were obtained upon request from the corresponding author and OR (95% CI) were calculated using Medcalc (https://www.medcalc.org/calc/odds_ratio.php):<br><br>Alcohol use = 0.96 (0.41-2.26)
Mudumbai 2019* (4th)	Chi-square test comparing the readmitted patients (n=531 of 5514)		Overall % vs readmitted % (p-value) presented below:	Multivariate, extended Cox regression analysis (Hazard Ratio (95% CI)) for readmission from postdischarge days 0 to 30 - all independent

	with the overall cohort (n=5514), with two-sided p-value of ≤0.05 considered statistically significant		Diagnoses in the 365 d before admission: substance use disorder (excludes nicotine) = 7.40 vs 9.42 (0.075)	variables were included simultaneously in the model:<br><br>Diagnoses in the 365d before admission: substance use disorder (excludes nicotine) = 1.00 (0.72-1.40)
Psychoses and psychiatric disorder:				
Ali 2019 (1st)	N/A	N/A	N/A	Population attributable fraction was calculated for each of the strongest predictors for readmission. This is the proportion of the incidence rate in the whole population that is due to have that particular comorbidity.<br><br>Pyschoses = 0.2%
Arroyo 2019 (2nd)	Continuous variables analysed using analysis of variance; categorical variables analysed using Pearson chi-square test or Fisher exact test.	N/A	**Achieved statistical significance with p<0.001:**<br>Psychoses	See section S10 - Arroyo 2019 – Results from Exploratory Stratified Analysis
Neoplastic				
History of cancer including metastases:				
Pugely 2013 (1st)	Student's t-test for continuous variables; chi-square test for categorical variables	N/A	Statistical significance was set at p < 0.05. Values presented as: variable (p-value) = non-readmitted proportion (%) vs readmitted proportion (%)<br><br>Disseminated Cancer **(0.0381)** = 0.04 vs 0.37	N/A
Arroyo 2019 (2nd)	Continuous variables analysed using analysis of variance; categorical variables analysed using Pearson chi-square test or Fisher exact test.	N/A	**Achieved statistical significance with p<0.001:**<br>Solid tumor without metastasis	See section S10 - Arroyo 2019 – Results from Exploratory Stratified Analysis
Neurological				
Previous stroke:				
Belmont 2016** (1st)	Bivariate logistic regression (OR (95% CI))	Previous TIA/CVA/stroke with neurologic deficit/CVA/stroke without neurologic deficit = **3.17 (1.29-7.79)**	N/A	N/A

Liao 2016* (2nd)	Chi-square test – statistical significance was indicated by two-sided p-value of 0.05	N/A	Not statistically significant on univariate analysis (% readmission rate; individual p-values not reported):<br>CVA: yes = 5.5% vs no = 4.1	
Paralysis and other neurological disorder:				
Ali 2019 (1st)	N/A	N/A	N/A	Population attributable fraction was calculated for each of the strongest predictors for readmission. This is the proportion of the incidence rate in the whole population that is due to have that particular comorbidity.<br><br>Other neurological disorder = 1.2%
Arroyo 2019 (2nd)	Continuous variables analysed using analysis of variance; categorical variables analysed using Pearson chi-square test or Fisher exact test.	N/A	**Achieved statistical significance with p<0.001:**<br>Other neurological disorders	See section S10 - Arroyo 2019 – Results from Exploratory Stratified Analysis
Renal				
Chronic kidney disease (CKD) and renal disease/failure - unspecified:				
Liao 2016* (2nd)	Chi-square test – statistical significance was indicated by two-sided p-value of 0.05	N/A	Statistically significant on univariate analysis (% readmission rate; individual p-values not reported):<br>CKD: **yes = 6.4 vs no = 3.9**	Sensitivity analysis (HR (p-value)).<br><br>Age <75y group:<br>CKD = **2.36 (0.003)**<br><br>Age > 80y group:<br>CKD = **2.29 (0.001)**
Workman 2019 (3rd)	Student's t test for continuous variables; Fisher's exact test for categorical variables	N/A	Chronic kidney disease **(0.0082)**	N/A
Antoniak 2020 (4th)	Mantel-Haenszel chi-squared	N/A	**Risk increased with increasing CKD stage (p <0.0001)**:<br>No CKD (2.7% of 18,771 patients)<br>Stage 2 (3.2% of 74,912 patients)	N/A for readmission (only applicable for composite outcome, 'major complications')

			Stage 3a (4.1% of 21,977 patients)<br>Stage 3b (5.7% of 8225 patients)<br>Stage 4 (7.1% of 1438 patients)	
Renal failure/disease – chronicity unspecified:				
Arroyo 2019 (2nd)	Continuous variables analysed using analysis of variance; categorical variables analysed using Pearson chi-square test or Fisher exact test.	N/A	**Achieved statistical significance with p<0.001:**<br>Renal failure	See section S10 - Arroyo 2019 – Results from Exploratory Stratified Analysis
Siracuse 2017 (3rd)	Chi-square test; Student t tests; binary logistic regression	Renal failure = **1.87 (1.77-1.98)**	Not reported	N/A
Dialysis dependence:				
Pugely 2013 (1st)	Student's t-test for continuous variables; chi-square test for categorical variables	N/A	Statistical significance was set at p < 0.05. Values presented as: variable (p-value) = non-readmitted proportion (%) vs readmitted proportion (%)<br><br>Dialysis **(0.0156) =** 0.08 vs 0.55	N/A
Lehtonen 2018 (1st)	t-test to compare continuous variables; chi-square to compare categorical variables	N/A	Authors chose p<0.0001 to indicate statistical significance. Results are presented as non-readmitted group (%) vs readmitted group (%) = p-value<br><br>Comorbidities:<br>Dialysis use: 0.2 vs 0.5 = **p<0.0001**	N/A
Belmont 2016** (1st)	Bivariate logistic regression (OR (95% CI))	Dialysis use/renal failure = 0.89 (0.04-18.41)	N/A	N/A
Patterson 2018 (2nd)	Bivariate Poisson regression with robust error variance for	Dialysis dependence (RR (95% CI)) = **2.6 (1.8-3.8)**	N/A	N/A

	nonparametrically distributed data			
Ottesen 2018 (2nd)	Univariate logistic regression	Dialysis dependence (OR (95% CI)) = **3.65 (2.34-5.67)**	N/A	N/A
Rheumatological and autoimmune				
Rheumatoid arthritis:				
Siracuse 2017 (3rd)	Chi-square test; Student t tests; binary logistic regression	Rheumatoid arthritis = **1.17 (1.09-1.25)**	Not reported	N/A
Rheumatological disorder:				
Arroyo 2019 (2nd)	Continuous variables analysed using analysis of variance; categorical variables analysed using Pearson chi-square test or Fisher exact test.	N/A	**Achieved statistical significance with p<0.001:** Rheumatoid arthritis/collagen vascular diseases	See section S10 - Arroyo 2019 – Results from Exploratory Stratified Analysis
Steroid or other immunosuppressant use for chronic condition:				
Lehtonen 2018 (1st)	t-test to compare continuous variables; chi-square to compare categorical variables	N/A	Authors chose p<0.0001 to indicate statistical significance. Results are presented as non-readmitted group (%) vs readmitted group (%) = p-value<br><br>Comorbidities:<br>Corticosteroid use: 3.5 vs 5.6 = **p<0.0001**	N/A
Belmont 2016** (1st)	Bivariate logistic regression (OR (95% CI))	Steroid use for chronic condition = 0.93 (0.37-2.33)	N/A	N/A
Pugely 2013 (1st)	Student's t-test for continuous variables; chi-square test for categorical variables	N/A	Statistical significance was set at p < 0.05. Values presented as: variable (p-value) = non-readmitted proportion (%) vs readmitted proportion (%)<br><br>Steroid Use (0.5883) = 2.57 vs 2.95	N/A

Curtis 2018 (2nd)	Chi square or Fisher exact test was used for categorical outcomes in the study – it is unclear which was used in this particular instance	N/A	Control (3.5%) vs immunosuppressed (5.2%) = **p < 0.001**	N/A
Other				
Preoperative opioid use:				
Kim 2019* (1st)	Cox proportional hazards for unadjusted effect estimates	HR (95% CI):<br>Continuous opioid used (reference = opioid naïve) = **1.47 (1.36-1.60)**<br>Intermittent opioid use (reference = opioid naïve) = **1.18 (1.13-1.23)**	N/A	Sensitivity analysis (continuous opioid users vs opioid-naive patients, excluding patients with malignant tumours):<br>Unadjusted = **1.48 (1.36-1.62)**<br>Model 1 = **1.57 (1.44-1.72)**<br>Model 2 = 1.05 (0.96-1.16)
Weick 2018 (3rd)	ANOVA for univariate analysis; logistic regression for unadjusted effect estimate	>60 days preoperative opioid use vs 0 to 60 days = **1.24 (1.19-1.30)**		N/A
Mudumbai 2019* (4th)	Chi-square test comparing the readmitted patients (n=531 of 5514) with the overall cohort (n=5514), with two-sided p-value of ≤0.05 considered statistically significant		Overall % vs readmitted % (p-value) presented below:<br><br>Preoperative outpatient opioids – status for the 180 d preoperatively:<br>tramadol only = 10.07 vs 10.36 (0.873)<br>short-acting acute = 24.90 vs 27.31 (0.195)<br>short-acting chronic = 21.51 vs 21.09 (0.849)<br>any long-acting = 4.23 vs 3.95 (0.831)<br>no opioids = 39.30 vs 37.29 (0.341)	Multivariate, extended Cox regression analysis (Hazard Ratio (95% CI)) for readmission from postdischarge days 0 to 30 - all independent variables were included simultaneously in the model:<br><br>Preoperative outpatient opioids - status for the 180d preoperatively:<br>tramadol only = 0.93 (0.68-1.27)<br>short-acting acute = 1.03 (0.83-1.29)<br>short-acting chronic = 0.84 (0.65-1.09)<br>any long-acting = 0.76 (0.4-1.43)<br>no opioids = reference range
Various preoperative medication use:				
Mudumbai 2019* (4th)	Chi-square test comparing the readmitted patients (n=531 of 5514) with the overall cohort (n=5514), with two-sided p-value of ≤0.05		Overall % vs readmitted % (p-value) presented below:	Multivariate, extended Cox regression analysis (Hazard Ratio (95% CI)) for readmission from postdischarge days 0 to 30 - all independent variables were included simultaneously in the model:

	considered statistically significant		Preoperative adjunctive pharmacotherapy for the 180 d preoperatively:<br>analgesics = 47.03 vs 48.02 (0.661)<br>SNRIs = 10.39 vs 12.24 (0.163)<br>anticonvulsants = 16.78 vs 22.41 **(<0.001)**<br>TCAs = 7.69 vs 7.16 (0.690)<br>sedatives = 18.44 vs 21.28 (0.087)	Preoperative adjunctive pharmacotherapy for the 180d preoperatively:<br>analgesics = 1.03 (0.87-1.23)<br>anticonvulsants = **1.32 (1.06-1.64)**<br>SNRIs = 1.08 (0.82-1.43)<br>TCAs = 0.82 (0.58-1.15)<br>sedatives = 1.08 (0.86-1.36)
Post-discharge opioid use:				
Mudumbai 2019* (4th)	Chi-square test comparing the readmitted patients (n=531 of 5514) with the overall cohort (n=5514), with two-sided p-value of ≤0.05 considered statistically significant		Overall % vs readmitted % (p-value) presented below:<br><br>Post-discharge opioid status as of 30 d:<br>tramadol only = 1.72 vs 3.01 **(0.026)**<br>short-acting only = 83.55 vs 58.57 **(<0.001)**<br>any long-acting = 4.03 vs 3.39 (0.504)<br>no opioids = 10.70 vs 35.03 **(<0.001)**	Multivariate, extended Cox regression analysis (Hazard Ratio (95% CI)) for readmission from postdischarge days 0 to 30 - all independent variables were included simultaneously in the model:<br><br>Postdischarge opioid status as of 30d:<br>tramadol only = 1.12 (0.72-1.74)<br>short-acting only = **1.38 (1.14-1.67)**<br>any long-acting = 1.28 (0.63-2.61)<br>no opioids = reference range
Wound class:				
Belmont 2016** (1st)	Bivariate logistic regression (OR (95% CI))	Wound classification other than clean (reference clean) = 1.90 (0.74-4.91)	N/A	N/A
Open wound:				
Lehtonen 2018 (1st)	t-test to compare continuous variables; chi-square to compare categorical variables	N/A	Authors chose p<0.0001 to indicate statistical significance. Results are presented as non-readmitted group (%) vs readmitted group (%) = p-value<br><br>Comorbidities:	N/A

			Open/infected wound: 0.3 vs 0.8 = **p<0.0001**	
Belmont 2016** (1st)	Bivariate logistic regression (OR (95% CI))	Preoperative open wound = 1.09 (0.26-4.64)	N/A	N/A
Sepsis and related:				
Lehtonen 2018 (1st)	t-test to compare continuous variables; chi-square to compare categorical variables	N/A	Authors chose p<0.0001 to indicate statistical significance. Results are presented as non-readmitted group (%) vs readmitted group (%) = p-value<br><br>Comorbidities:<br>Sepsis: 0.0 vs 0.0 = **p<0.0001**<br>Septic shock: 0.0 vs 0.0 = **p<0.0001**<br>SIRS: 0.2 vs 0.3 = **p<0.0001**	N/A
Belmont 2016** (1st)	Bivariate logistic regression (OR (95% CI))	Sepsis within 48 hours prior to surgery = 2.37 (0.53-10.64)	N/A	N/A
Chronic pain:				
Mudumbai 2019* (4th)	Chi-square test comparing the readmitted patients (n=531 of 5514) with the overall cohort (n=5514), with two-sided p-value of ≤0.05 considered statistically significant		Overall % vs readmitted % (p-value) presented below:<br><br>Had chronic pain in the 365 d before admission = 96.41 vs 95.10 (0.115)	Multivariate, extended Cox regression analysis (Hazard Ratio (95% CI)) for readmission from postdischarge days 0 to 30 - all independent variables were included simultaneously in the model:<br><br>Had chronic pain in the 365d before admission = **0.65 (0.43-0.97)**
Demographics				
Age				
Continuous:				
Urish 2018 (1st)	t test for parametric continuous variables; Wilcoxon rank-sum test for nonparametric continuous variables; chi-square test for proportions.	N/A	**All of the following were significantly different (at p <0.05)** between readmitted and non-readmitted patients:<br>Mean age (non-readmitted = 67 (9) vs readmitted = 66 (10))	N/A

Lehtonen 2018 (1st)	t-test to compare continuous variables; chi-square to compare categorical variables	N/A	Authors chose p<0.0001 to indicate statistical significance. Results are presented as non-readmitted group (%) vs readmitted group (%) = p-value<br><br>Age: 66.53 years old vs 68.49 years old = **p<0.0001**	N/A
Belmont 2016** (1st)	Bivariate logistic regression (OR (95% CI))	Age (continuous) = 1.01 (1.00-1.03; p-value non-significant)	N/A	N/A
Arroyo 2019 (2nd)	Continuous variables analysed using analysis of variance; categorical variables analysed using Pearson chi-square test or Fisher exact test.	N/A	**Achieved statistical significance with p<0.001:**<br>Age = 69.23 (10.81) readmitted vs 67.21 (10.05) non-readmitted	See section S10 - Arroyo 2019 – Results from Exploratory Stratified Analysis
Workman 2019 (3rd)	Student's t test for continuous variables; Fisher's exact test for categorical variables	N/A	Age (continuous; **< 0.0001**):<br>68.5 (10.9) readmitted vs 64.9 (9.8)	N/A
Kheir 2014 (4th)	Bivariate logistic regression analysis for unadjusted effect estimates<br><br>Categorical variables compared using chi-square test; continuous non-parametric variables compared using Mann-Whitney U-test	OR (95%)<br><br>Age (continuous) = 1.01 (0.99-1.02)	Results are presented as non-readmitted group (% or mean (SD)) vs readmitted group (% or mean (SD)) = p-value. P-value <0.05 was considered statistically significant.<br><br>Age: 62.9 (10.8) vs 63.9 (12.6) = 0.100	N/A
Categorised:				
Urish 2018 (1st)	t test for parametric continuous variables; Wilcoxon rank-sum test for nonparametric continuous variables; chi-square test for proportions.	N/A	**All of the following were significantly different (at p <0.05)** between readmitted and non-readmitted patients:<br>Age category (55-64; 65-74; 75-85; 85+)	N/A

Pugely 2013 (1st)	Student's t-test for continuous variables; chi-square test for categorical variables	N/A	Statistical significance was set at $p < 0.05$. Values presented as: variable (p-value) = non-readmitted proportion (%) vs readmitted proportion (%)<br><br>Age **(0.0036)**:<br><45 = 1.66 vs 2.95<br>46–55 = 11.57 vs 10.50<br>56–65 = 31.96 vs 24.49<br>66–75 = 34.23 vs 32.41<br>76–85 = 18.27 vs 25.41<br>>85 = 2.30 vs 4.24	N/A
Bovonratwet 2018** (1st)	Fisher's exact or chi-square	N/A	Before propensity score matching:<br><br>Age <70 (5.20%) vs age ≥80 (6.23%): p = 0.220<br><br>Age 70-79 (5.55%) vs age ≥80 (6.23%): p = 0.475	N/A
Belmont 2016** (1st)	Bivariate logistic regression (OR (95% CI))	Reference category: ≤59<br><br>Age 60-69 = 1.38 (0.80-2.38)<br>Age 70-79 = 1.65 (0.96-2.85)<br>Age ≥80 = 1.39 (0.69-2.81)	N/A	N/A
Siracuse 2017 (3rd)	Chi-square test; Student t tests; binary logistic regression	Age (reference category: 51-60):<br>41-50 = 1.05 (0.97-1.13)<br>61-70 = **1.12 (1.08-1.17)**<br>71-80 = **1.47 (1.41-1.53)**<br>81-90 = **2.13 (2.03-2.23)**	Not reported	N/A
Mudumbai 2019* (4th)	Chi-square test comparing the readmitted patients (n=531 of 5514) with the overall cohort (n=5514), with two-sided p-value of ≤0.05 considered statistically significant	N/A	Overall % vs readmitted % (p-value) presented below:<br><br>Age:<br>≤54 = 14.96 vs 10.36 **(0.002)**	Multivariate, extended Cox regression analysis (Hazard Ratio (95% CI)) for readmission from postdischarge days 0 to 30 - all independent variables were included simultaneously in the model:<br><br>Age <= 54 = **0.44 (0.32-0.61)**<br>age 55-65 = **0.7 (0.58-0.86)**

			55-65 = 52.14 vs 50.09 (0.344)<br>≥66 = 32.90 vs 39.55 **(<0.001)**	age >= 66 = reference range
Charette 2019 (4th)	Chi square or Fisher exact test	N/A	Age <55 group (6.1%) vs age ≥55 group (7.2%): p = 0.723	N/A
Miric 2014 (4th)	Chi-square test – alpha = 0.05 was used as the threshold for statistical significance	N/A	Readmission rate increased with increasing age:<br><80 = 3.2% vs 80-89 = 7.1% vs 90+ = 9.5% **(p<0.001)**	N/A
Kheir 2014 (4th)	Bivariate logistic regression analysis for unadjusted effect estimates	OR (95%)<br><br>Age (reference category = ≤55):<br>56-65 = 0.97 (0.63-1.49)<br>66-75 = 1.26 (0.82-1.94)<br>≥76 = 1.44 (0.87-2.39)		N/A
Sex				
Female:				
Belmont 2016** (1st)	Bivariate logistic regression (OR (95% CI))	Female sex (male) = 1.66 (1.12-2.45)	N/A	N/A
Mudumbai 2019* (4th)	Chi-square test comparing the readmitted patients (n=531 of 5514) with the overall cohort (n=5514), with two-sided p-value of ≤0.05 considered statistically significant	N/A	Overall % vs readmitted % (p-value) presented below:<br><br>Female sex = 5.75 vs 6.59 (0.436)	Multivariate, extended Cox regression analysis (Hazard Ratio (95% CI)) for readmission from postdischarge days 0 to 30 - all independent variables were included simultaneously in the model:<br><br>Female sex = 1.13 (0.79-1.62)
Male:				
Urish 2018 (1st)	t test for parametric continuous variables; Wilcoxon rank-sum test for nonparametric continuous variables; chi-square test for proportions.	N/A	**Significantly different (at p <0.0001)** between readmitted and non-readmitted patients:<br>Female = 63% non-readmitted vs 57% readmitted<br>Male = 37% non-readmitted vs 43% readmitted	N/A

Lehtonen 2018 (1st)	t-test to compare continuous variables; chi-square to compare categorical variables	N/A	Authors chose p<0.0001 to indicate statistical significance. Results are presented as non-readmitted group (%) vs readmitted group (%) = p-value<br><br>Sex: **p<0.0001**<br>Male: 37.6 vs 43.7<br>Female: 62.4 vs 56.3	N/A
Pugely 2013 (1st)	Student's t-test for continuous variables; chi-square test for categorical variables	N/A	Statistical significance was set at p < 0.05. Values presented as: variable (p-value) = non-readmitted proportion (%) vs readmitted proportion (%)<br><br>Sex **(0.0014)**:<br>Male = 37.20 vs 43.99<br>Female = 62.80 vs 56.01	N/A
Arroyo 2019 (2nd)	Continuous variables analysed using analysis of variance; categorical variables analysed using Pearson chi-square test or Fisher exact test.	N/A	**Achieved statistical significance with p<0.001:**<br>Sex:<br>Male = 36.8% non-readmitted vs 41.7% readmitted<br>Female = 63.2% non-readmitted vs 58.3% readmitted	See section S10 - Arroyo 2019 – Results from Exploratory Stratified Analysis
Liao 2016* (2nd)	Chi-square test – statistical significance was indicated by two-sided p-value of 0.05	N/A	Statistically significant on univariate analysis (% readmission rate; individual p-values not reported):<br>Sex: **male = 6.1 vs female = 3.7**	Sensitivity analysis (HR (p-value)).<br><br>Age <75y group:<br>Sex (reference category unclear) = 0.73 (0.190)<br><br>Age > 80y group: Sex (reference category unclear) = 0.76 (0.168)
Workman 2019 (3rd)	Student's t test for continuous variables;	N/A	Sex (p = 0.0820):	N/A

	Fisher's exact test for categorical variables		Male = 35.13% non-readmitted vs 40.48% readmitted	
Siracuse 2017 (3rd)	Chi-square test; Student t tests; binary logistic regression	Male sex = **1.13 (1.09-1.16)**	Not reported	N/A
Singh 2013 (3rd)	Chi-square test	N/A	Men had higher readmission rate than women **(6.74% vs 5.44%, p = 0.0004)**	N/A
Jorgensen 2013* (3rd)	N/A	N/A	N/A	Data were obtained upon request from the corresponding author and OR (95% CI) were calculated using Medcalc (https://www.medcalc.org/calc/odds_ratio.php):<br><br>Male sex = 1.49 (0.96-2.30)
Robinson 2017 (4th)	Chi-square test	N/A	Readmission rate was higher in males than females **(p<0.001)**:<br>Males = 4.0%<br>Females = 3.1%	N/A
Kheir 2014 (4th)	Bivariate logistic regression analysis for unadjusted effect estimates<br><br>Categorical variables compared using chi-square test; continuous non-parametric variables compared using Mann-Whitney U-test	OR (95%)<br><br>Male sex = 0.94 (0.68-1.31)	Results are presented as non-readmitted group (% or mean (SD)) vs readmitted group (% or mean (SD)) = p-value. P-value <0.05 was considered statistically significant.<br><br>Sex:<br>Female: 65.9 vs 67.9 = 0.608<br>Male: 34.1 vs 32.1 = - (not reported)	N/A
Race and ethnicity				
Black:				
Workman 2019 (3rd)	Student's t test for continuous variables; Fisher's exact test for categorical variables	N/A	African American race (0.1137)	N/A

Siracuse 2017 (3rd)	Chi-square test; Student t tests; binary logistic regression	African-American = **1.37 (1.30-1.44)**	Not reported	N/A
Mudumbai 2019* (4th)	Chi-square test comparing the readmitted patients (n=531 of 5514) with the overall cohort (n=5514), with two-sided p-value of ≤0.05 considered statistically significant		Overall % vs readmitted % (p-value) presented below:<br><br>Race: African American/black = 16.85 vs 20.72 **(0.015)**	Multivariate, extended Cox regression analysis (Hazard Ratio (95% CI)) for readmission from postdischarge days 0 to 30 - all independent variables were included simultaneously in the model:<br><br>Race:<br>African American/black = 0.84 (0.43-1.62)
Kheir 2014 (4th)	Bivariate logistic regression analysis for unadjusted effect estimates<br><br>Categorical variables compared using chi-square test; continuous non-parametric variables compared using Mann-Whitney U-test	OR (95%)<br><br>Race (reference = white):<br>Black = 1.28 (0.92-1.80)<br>Other = 1.11 (0.47-2.57)	Results are presented as non-readmitted group (% or mean (SD)) vs readmitted group (% or mean (SD)) = p-value. P-value <0.05 was considered statistically significant.<br><br>Race:<br>Black: 27.9 vs 33.3 = 0.129	N/A
Hispanic:				
Mudumbai 2019* (4th)	Chi-square test comparing the readmitted patients (n=531 of 5514) with the overall cohort (n=5514), with two-sided p-value of ≤0.05 considered statistically significant			Multivariate, extended Cox regression analysis (Hazard Ratio (95% CI)) for readmission from postdischarge days 0 to 30 - all independent variables were included simultaneously in the model:<br><br>Race: Hispanic = 0.92 (0.63-1.35)
Asian:				
Mudumbai 2019* (4th)	Chi-square test comparing the readmitted patients (n=531 of 5514) with the overall cohort (n=5514), with two-sided p-value of ≤0.05 considered statistically significant		Overall % vs readmitted % (p-value) presented below:<br><br>Race:<br>Asian = 0.80 vs 0.38 (0.373)	Multivariate, extended Cox regression analysis (Hazard Ratio (95% CI)) for readmission from postdischarge days 0 to 30 - all independent variables were included simultaneously in the model:<br><br>Race:<br>Asian = 0.42 (0.1-1.82)
Kheir 2014 (4th)	Bivariate logistic regression analysis for		Results are presented as non-readmitted group (%	N/A

	unadjusted effect estimates<br><br>Categorical variables compared using chi-square test; continuous non-parametric variables compared using Mann-Whitney U-test		or mean (SD)) vs readmitted group (% or mean (SD)) = p-value. P-value <0.05 was considered statistically significant.<br><br>Race:<br>Asian: 1.5 vs 1.2 = 0.761	
American Indian:				
Mudumbai 2019* (4th)	Chi-square test comparing the readmitted patients (n=531 of 5514) with the overall cohort (n=5514), with two-sided p-value of ≤0.05 considered statistically significant		Overall % vs readmitted % (p-value) presented below:<br><br>Race:<br>American Indian = 1.18 vs 1.32 (0.919)	Multivariate, extended Cox regression analysis (Hazard Ratio (95% CI)) for readmission from postdischarge days 0 to 30 - all independent variables were included simultaneously in the model:<br><br>Race:<br>American Indian = 0.92 (0.38-2.24)
Kheir 2014 (4th)	Bivariate logistic regression analysis for unadjusted effect estimates<br><br>Categorical variables compared using chi-square test; continuous non-parametric variables compared using Mann-Whitney U-test		Results are presented as non-readmitted group (% or mean (SD)) vs readmitted group (% or mean (SD)) = p-value. P-value <0.05 was considered statistically significant.<br><br>Race:<br>Native American: 0.1 vs 0.1 = 0.687	N/A
White:				
Workman 2019 (3rd)	Student's t test for continuous variables; Fisher's exact test for categorical variables	N/A	Caucasian race (0.3468)	N/A
Mudumbai 2019* (4th)	Chi-square test comparing the readmitted patients (n=531 of 5514) with the overall cohort (n=5514), with two-sided p-value of ≤0.05 considered statistically significant		Overall % vs readmitted % (p-value) presented below:<br><br>Race:<br>Caucasian/white = 80.70 vs 76.65 **(0.012)**	Multivariate, extended Cox regression analysis (Hazard Ratio (95% CI)) for readmission from postdischarge days 0 to 30 - all independent variables were included simultaneously in the model:<br><br>Race:<br>Caucasian/white = 0.7 (0.37-1.32)

Kheir 2014 (4th)	Bivariate logistic regression analysis for unadjusted effect estimates<br><br>Categorical variables compared using chi-square test; continuous non-parametric variables compared using Mann-Whitney U-test		Results are presented as non-readmitted group (% or mean (SD)) vs readmitted group (% or mean (SD)) = p-value. P-value <0.05 was considered statistically significant.<br><br>Race:<br>White: 67.1 vs 60.6 = 0.084	N/A
Biracial:				
Workman 2019 (3rd)	Student's t test for continuous variables; Fisher's exact test for categorical variables	N/A	Biracial **(0.0353)**	N/A
Other:				
Siracuse 2017 (3rd)	Chi-square test; Student t tests; binary logistic regression	Other = **1.12 (1.08-1.16)**	Not reported	N/A
Kheir 2014 (4th)	Bivariate logistic regression analysis for unadjusted effect estimates<br><br>Categorical variables compared using chi-square test; continuous non-parametric variables compared using Mann-Whitney U-test	OR (95%)<br><br>Race (reference = white):<br>Other = 1.11 (0.47-2.57)	Results are presented as non-readmitted group (% or mean (SD)) vs readmitted group (% or mean (SD)) = p-value. P-value <0.05 was considered statistically significant.<br><br>Race:<br>Other: 1.9 vs 2.4 = 0.633	N/A
Missing:				
Kheir 2014 (4th)	Bivariate logistic regression analysis for unadjusted effect estimates<br><br>Categorical variables compared using chi-square test; continuous non-parametric variables	OR	Results are presented as non-readmitted group (% or mean (SD)) vs readmitted group (% or mean (SD)) = p-value. P-value <0.05 was considered statistically significant.<br><br>Race:	N/A

	compared using Mann-Whitney U-test		Unknown: 1.5 vs 2.4 = 0.353	
Race – various categories combined:				
Pugely 2013 (1st)	Student's t-test for continuous variables; chi-square test for categorical variables	N/A	Statistical significance was set at p < 0.05. Values presented as: variable (p-value) = non-readmitted proportion (%) vs readmitted proportion (%)<br><br>Race (0.5627):<br>Black = 6.23 vs 5.77<br>White = 80.84 vs 82.68<br>Other =12.93 vs 11.55	N/A
Lehtonen 2018 (1st)	t-test to compare continuous variables; chi-square to compare categorical variables	N/A	Authors chose p<0.0001 to indicate statistical significance. Results are presented as non-readmitted group (%) vs readmitted group (%) = p-value<br><br>Race: = **p<0.0001**<br>White: 78.2 vs 79.1<br>Black: 7.2 vs 9.4<br>Asian: 2.2 vs 1.4<br>American Indian: 0.5 vs 0.3<br>Native Hawaiian: 0.3 vs 0.2<br>Unreported: 11.6 vs 9.6	N/A
Arroyo 2019 (2nd)	Continuous variables analysed using analysis of variance; categorical variables analysed using Pearson chi-square test or Fisher exact test.	N/A	**Achieved statistical significance with p<0.001:**<br>Race	See section S10 - Arroyo 2019 – Results from Exploratory Stratified Analysis
Socioeconomic				
Income:				
Urish 2018 (1st)	t test for parametric continuous variables; Wilcoxon rank-sum test	N/A	**All of the following were significantly different (at p <0.0001)**	N/A

	for nonparametric continuous variables; chi-square test for proportions.		between readmitted and non-readmitted patients:<br>Median household income<br>37,999 or less = 20% of non-readmitted cohort, 23% of readmitted cohort<br>38,000-47,999 = 26% of non-readmitted cohort, 27% of readmitted cohort<br>48,000-63999 = 27% of non-readmitted cohort, 27% of readmitted cohort<br>64,000 or more = 23 of non-readmitted cohort, 23% of readmitted cohort	
Arroyo 2019 (2nd)	Continuous variables analysed using analysis of variance; categorical variables analysed using Pearson chi-square test or Fisher exact test.	N/A	**Achieved statistical significance with p<0.001:**<br>Median income quartile of postal (ZIP) code<br>First quartile = 19.8% of readmitted cohort, 21.7% of non-readmitted cohort<br>Second quartile = 25.3% of non-readmitted cohort, 25.0% of readmitted cohort<br>Third quartile = 27.0% of non-readmitted cohort, 26.4% of readmitted cohort<br>Fourth quartile = 26.1% of non-readmitted cohort, 25.0% readmitted cohort<br>Missing = 1.8% of non-readmitted cohort, 1.9% of readmitted cohort	See section S10 - Arroyo 2019 – Results from Exploratory Stratified Analysis
Siracuse 2017 (3rd)	Chi-square test; Student t tests; binary logistic regression	Income quartile (reference category = 4th):<br>1st = **1.07 (1.03-1.12)**<br>2nd = 1.00 (0.96-1.04)<br>3rd = 0.99 (0.95-1.03)	Not reported	N/A
Insurance status:				

Arroyo 2019 (2nd)	Continuous variables analysed using analysis of variance; categorical variables analysed using Pearson chi-square test or Fisher exact test.	N/A	**Achieved statistical significance with p<0.0001:**<br>Payer status<br>Medicare = 59.7% of non-readmitted cohort, 68.8% of readmitted cohort<br>Medicaid = 3.1% of non-readmitted cohort, 4.1% of readmitted cohort<br>Private insurance (other) = 4.6% of non-readmitted cohort, 3.8% of readmitted cohort<br>Private insurance (self-pay/no-charge) = 0.5% of non-readmitted cohort, 0.4% of readmitted cohort	See section S10 - Arroyo 2019 – Results from Exploratory Stratified Analysis
Siracuse 2017 (3rd)	Chi-square test; Student t tests; binary logistic regression	Primary payer:<br>Medicare = **1.58 (1.53-1.63)**<br>Medicaid = **1.62 (1.50-1.74)**<br>Self-pay = 0.74 (0.56-0.99)	Not reported	N/A
Socioeconomic status indices:				
Keeney 2015 (4th)	Two-tailed Fisher's exact test	N/A	P<0.05 considered statistically significant.<br><br>Socioeconomically disadvantaged TKA patients had higher 30-day readmission risks both before and after risk reduction protocols were introduced:<br>Before introduction of risk reduction protocols = 8.0%, disadvantaged group vs 3.4%, non-disadvantaged group (p < 0.01)<br>After introduction of risk reduction protocols = 4.6%, disadvantaged	N/A

			group vs 1.8%, non-disadvantaged group (p = 0.02)<br><br>A significant decline in 30-day readmission was only noted among non-minority TKA patients: 4.9% disadvantaged (prior to introduction of risk reduction protocols) vs 2.8% disadvantaged (after introduction of risk reduction protocols), P < 0.01	
Functional status, living situation, and frailty				
Functional status:				
Belmont 2016** (1st)	Bivariate logistic regression (OR (95% CI))	Partially/totally dependent functional status (reference independent) = 0.73 (0.26-2.02)	N/A	N/A
Jorgensen 2013* (3rd)	N/A	N/A	N/A	Data were obtained upon request from the corresponding author and OR (95% CI) were calculated using Medcalc (https://www.medcalc.org/calc/odds_ratio.php):<br><br>Use of walking aids = **2.23 (1.46-3.42)**
Living situation:				
Jorgensen 2013* (3rd)	N/A	N/A	N/A	Data were obtained upon request from the corresponding author and OR (95% CI) were calculated using Medcalc (https://www.medcalc.org/calc/odds_ratio.php):<br><br>Living alone (reference category = living with others) = 1.32 (0.87-2.02)<br><br>Living in nursing home = no readmissions
Mudumbai 2019* (4th)	Chi-square test comparing the readmitted patients (n=531 of 5514) with the overall cohort (n=5514), with two-sided p-value of ≤0.05 considered statistically significant		Overall % vs readmitted % (p-value) presented below:<br><br>Homeless = 3.66 vs 6.03 **(0.003)**	Multivariate, extended Cox regression analysis (Hazard Ratio (95% CI)) for readmission from postdischarge days 0 to 30 - all independent variables were included simultaneously in the model:<br><br>Homeless = 1.41 (0.94-2.11)

Marital status:				
Mudumbai 2019* (4th)	Chi-square test comparing the readmitted patients (n=531 of 5514) with the overall cohort (n=5514), with two-sided p-value of ≤0.05 considered statistically significant		Overall % vs readmitted % (p-value) presented below:<br><br>Currently married = 61.55 vs 57.63 (0.056)	Multivariate, extended Cox regression analysis (Hazard Ratio (95% CI)) for readmission from postdischarge days 0 to 30 - all independent variables were included simultaneously in the model:<br><br>Currently married = 0.92 (0.76-1.10)
Frailty:				
Runner 2017 (2nd)	N/A	N/A	N/A	Bivariate logistic regression analysis and chi-square test to determine the relationship between readmission and MFI score with adjustments for BMI<br><br>Incidence of readmission increased with increasing MFI level (OR (95% CI) = 1.41 (1.25-1.59)).<br>MFI = 0.0: readmission incidence = 3.05%<br>MFI = 0.09: readmission incidence = 4.66%<br>MFI = 0.18: readmission incidence = 6.46%<br>MFI = 0.27: readmission incidence = 5.88%<br>MFI ≥0.36: readmission incidence = 12.5%
Miscellaneous				
Operative variables				
Elective or non-elective procedure:				
Sodhi and Anis et al 2019 (1st)	Pearson's Chi-square	N/A	4514 (2.3%) elective vs 64 (2.8%) nonelective: p = 0.110	N/A
Traumatic indication for TKA:				
Kester 2016 (1st)	Fisher's exact test	N/A	Readmission rate: Non-traumatic (0.792%) vs post-traumatic (1.484%) = p-value 0.132	N/A
Bilateral procedure:				
Bullock 2003 (4th)	Relative risk (95% CI); chi-square	Simultaneous bilateral (3.6%) vs unilateral (2.3%) = 1.57 (0.65-3.55)	Simultaneous bilateral (3.6%) vs unilateral (2.3%) = p>0.25	N/A
Revision surgery:				
Courtney 2018** (1st)	Chi square, p<0.05 considered statistically significant	N/A	Infection group (10%) vs Aseptic group (6%) = **p<0.001**	N/A

Mudumbai 2019* (4th)	Chi-square test comparing the readmitted patients (n=531 of 5514) with the overall cohort (n=5514), with two-sided p-value of ≤0.05 considered statistically significant	N/A	Overall % vs readmitted % (p-value) presented below:<br><br>Surgery type:<br>primary = 89.86 vs 88.32 (0.246)<br>revision = 10.14 vs 11.68 (0.246)	Multivariate, extended Cox regression analysis (Hazard Ratio (95% CI)) for readmission from postdischarge days 0 to 30 - all independent variables were included simultaneously in the model:<br><br>Surgery type:<br>primary = 0.96 (0.71-1.62)<br>revision = reference range
Schairer 2014* (4th)	Chi-square, with p<0.05 considered statistically significant	N/A	The 30-day readmission rate showed significant (p= 0.047) differences across groups for primary TKA (3.4%), revision TKA (5.7%), and revision for infected TKA (6.2%).	N/A
In-hospital complications				
Venous thromboembolism:				
Lehtonen 2018 (1st)	t-test to compare continuous variables; chi-square to compare categorical variables	N/A	Authors chose p<0.0001 to indicate statistical significance. Results are presented as non-readmitted group (%) vs readmitted group (%) = p-value<br><br>Pulmonary embolism: 0.5 vs 6.8 = **p<0.0001**<br>Deep vein thrombosis: 0.7 vs 6.6 = **p<0.0001**	N/A
Belmont 2016** (1st)	Bivariate logistic regression (OR (95% CI))	Major systemic complications:<br>Pulmonary embolism = **15.65 (3.12-78.47)**<br><br>Minor systemic complications:<br>DVT = **7.88 (2.33-26.58)**	N/A	N/A
Any medical or surgical complication:				
Lehtonen 2018 (1st)	t-test to compare continuous variables; chi-square to compare categorical variables	N/A	Authors chose p<0.0001 to indicate statistical significance. Results are presented as non-readmitted group (%) vs readmitted group (%) = p-value	N/A

			Overall complications: 10.4 vs 58.0 = **p<0.0001**<br><br>Medical complications (8.9 vs 32.4 = **p<0.0001**)<br><br>Surgical complications (1.6 vs 25.6 = **p<0.0001**)	
Belmont 2016** (1st)	Bivariate logistic regression (OR (95% CI))	Overall complications = **14.42 (9.11-22.82)**<br><br>Major systemic complications = **12.75 (6.20-26.23)**<br><br>Minor systemic complications = **6.11 (2.97-12.56)**<br><br>Major local complications = **11.90 (5.15-27.47)**<br><br>Minor local complications = **24.37 (10.54-56.31)**<br><br>Mortality or major complication = **12.13 (6.80-21.63)**	N/A	N/A
Urinary tract infection (UTI):				
Lehtonen 2018 (1st)	t-test to compare continuous variables; chi-square to compare categorical variables	N/A	Authors chose p<0.0001 to indicate statistical significance. Results are presented as non-readmitted group (%) vs readmitted group (%) = p-value<br><br>Urinary tract infection: 0.7 vs 4.8 = **p<0.0001**	N/A
Belmont 2016** (1st)	Bivariate logistic regression (OR (95% CI))	Minor systemic complications:<br>UTI = **3.92 (1.29-11.93)**	N/A	N/A
Surgical site infection and wound complications:				
Lehtonen 2018 (1st)	t-test to compare continuous variables; chi-square to compare categorical variables	N/A	Authors chose p<0.0001 to indicate statistical significance. Results are presented as non-readmitted group (%) vs	N/A

			readmitted group (%) = p-value<br><br>Superficial surgical site infection: 0.4 vs 4.8 = **p<0.0001**<br>Deep or incisional surgical site infection: 0.0 vs 3.5 = **p<0.0001**<br>Organ or space surgical site infections: 0.0 vs 3.8 = **p<0.0001**	
Belmont 2016** (1st)	Bivariate logistic regression (OR (95% CI))	Major local complications:<br>Deep wound infection/organ or space SSI combined = **13.90 (5.86-32.96)**<br><br>Minor local complications:<br>Superficial wound infection = **18.61 (7.03-49.28)**<br>Wound disruption = **39.90 (7.65-208.11)**		
Sepsis and related :				
Lehtonen 2018 (1st)	t-test to compare continuous variables; chi-square to compare categorical variables	N/A	Authors chose p<0.0001 to indicate statistical significance. Results are presented as non-readmitted group (%) vs readmitted group (%) = p-value<br><br>Septic shock: 0.0 vs 1.2 = **p<0.0001**<br>Sepsis: 0.1 vs 4.1 = **p<0.0001**	N/A
Belmont 2016** (1st)	Bivariate logistic regression (OR (95% CI))	Major systemic complications:<br>Post-operative sepsis/septic shock combined = **7.94 (2.67-23.66)**	N/A	N/A
Cardiac:				
Lehtonen 2018 (1st)	t-test to compare continuous variables; chi-square to compare categorical variables	N/A	Authors chose p<0.0001 to indicate statistical significance. Results are presented as non-readmitted group (%) vs readmitted group (%) = p-value	N/A

			Cardiac arrest: 0.1 vs 0.6 = **p<0.0001**<br>Myocardial infarction: 0.1 vs 2.7 = **p<0.0001**	
Belmont 2016** (1st)	Bivariate logistic regression (OR (95% CI))	Major systemic complications:<br>Cardiac arrest requiring CPR = 2.17 (0.07-66.60)<br>Myocardial infarction = N/A	N/A	N/A
Pneumonia and respiratory:				
Lehtonen 2018 (1st)	t-test to compare continuous variables; chi-square to compare categorical variables	N/A	Authors chose p<0.0001 to indicate statistical significance. Results are presented as non-readmitted group (%) vs readmitted group (%) = p-value<br><br>Pneumonia: 0.3 vs 3.8 = **p<0.0001**<br>Unplanned intubation: 0.1 vs 1.4 = **p<0.0001**<br>Ventilator for more than 48 hours: 0.1 vs 0.8 = **p<0.0001**	N/A
Belmont 2016** (1st)	Bivariate logistic regression (OR (95% CI))	Major systemic complications:<br>Unplanned intubation = 1.68 (0.06-44.39)<br>Ventilator >48hrs = 5.16 (0.06-468.05)<br><br>Minor systemic complications:<br>Pneumonia = 3.84 (0.43-34.63)	N/A	N/A
Renal:				
Lehtonen 2018 (1st)	t-test to compare continuous variables; chi-square to compare categorical variables	N/A	Authors chose p<0.0001 to indicate statistical significance. Results are presented as non-readmitted group (%) vs readmitted group (%) = p-value<br><br>Acute renal failure: 0.0 vs 0.7 = **p<0.0001**	N/A
Belmont 2016** (1st)	Bivariate logistic regression (OR (95% CI))	Major systemic complications:<br>Acute renal failure = N/A	N/A	N/A

		Minor systemic complications:<br>Progressive renal insufficiency = **23.49 (2.67-23.66)**		
CVA or TIA:				
Lehtonen 2018 (1st)	t-test to compare continuous variables; chi-square to compare categorical variables	N/A	Authors chose p<0.0001 to indicate statistical significance. Results are presented as non-readmitted group (%) vs readmitted group (%) = p-value<br><br>Stroke or CVA: 0.1 vs 0.8 = **p<0.0001**	N/A
Belmont 2016** (1st)	Bivariate logistic regression (OR (95% CI))	Major systemic complications:<br>Stroke/CVA = **46.97 (4.85-455.21)**		
Other:				
Belmont 2016** (1st)	Bivariate logistic regression (OR (95% CI))	Major systemic complications:<br>Coma = N/A<br><br>Major local complications:<br>Peripheral nerve injury = 3.03 (0.07-125.62)<br>Graft/prosthesis failure = N/A		
Healthcare utilisation				
Chemotherapy within 30 days prior to operation:				
Pugely 2013 (1st)	Student's t-test for continuous variables; chi-square test for categorical variables	N/A	Statistical significance was set at p < 0.05. Values presented as: variable (p-value) = non-readmitted proportion (%) vs readmitted proportion (%)<br><br>Chemo w/in 30 Days (0.0579) = 0.15 vs 0.97	N/A
Radiation therapy within 90 days prior to operation:				
Pugely 2013 (1st)	Student's t-test for continuous variables; chi-square test for categorical variables	N/A	Statistical significance was set at p < 0.05. Values presented as: variable (p-value) = non-readmitted proportion (%)	N/A

			vs readmitted proportion (%)<br><br>Radiation Therapy w/in 90 Days (1) = 0.03 vs 0.00	
Prior operation:				
Pugely 2013 (1st)	Student's t-test for continuous variables; chi-square test for categorical variables	N/A	Statistical significance was set at p < 0.05. Values presented as: variable (p-value) = non-readmitted proportion (%) vs readmitted proportion (%)<br><br>Prior Operation w/in 30 Days (1) = 0.37 vs 0.00	N/A
Belmont 2016** (1st)	Bivariate logistic regression (OR (95% CI))	Prior operation of <30 days = 1.01 (0.05-21.62)	N/A	N/A
Mudumbai 2019* (4th)	Chi-square test comparing the readmitted patients (n=531 of 5514) with the overall cohort (n=5514), with two-sided p-value of ≤0.05 considered statistically significant		Overall % vs readmitted % (p-value) presented below:<br><br>Had a prior inpatient surgery in the 180 d before admission = 1.96 vs 1.88 (1.000)	Multivariate, extended Cox regression analysis (Hazard Ratio (95% CI)) for readmission from postdischarge days 0 to 30 - all independent variables were included simultaneously in the model:<br><br>Had a prior inpatient surgery in the 180d before admission = 0.82 (0.43-1.55)
Outpatient visits:				
Mudumbai 2019* (4th)	Chi-square test comparing the readmitted patients (n=531 of 5514) with the overall cohort (n=5514), with two-sided p-value of ≤0.05 considered statistically significant		Overall % vs readmitted % (p-value) presented below:<br><br>Had >30 outpatient visits in the 365 d before admission = 30.69 vs 43.31 **(<0.001)**	Multivariate, extended Cox regression analysis (Hazard Ratio (95% CI)) for readmission from postdischarge days 0 to 30 - all independent variables were included simultaneously in the model:<br><br>Had >30 outpatient visits in the 365d before admission = **1.56 (1.28-1.91)**
Patient-reported outcome measures (PROMs)				
Hospital Consumer Assessment of Healthcare Providers and Systems (HCAHPS) scores :				
Sodhi and Mont et al 2019* (3rd)	Categorical variables = Pearson's chi-squared test; continuous variables = Student's t test	N/A	P-value for comparison between readmitted and non-readmitted patients for each survey	N/A

			component is reported below.<br>Communication with nurses:<br>Nurses “always” treated you with courtesy and respect = .991<br>Nurses “always” listened carefully to you = .946<br>Nurses “always” explained things in a way you could understand = .376<br><br>Communication with doctors:<br>Doctors “always” treated you with courtesy and respect = .034<br>Doctors “always” listened carefully to you = .096<br>Doctors “always” explained things in a way you could understand = **.021**<br><br>Cleanliness and quietness of the hospital environment:<br>Your room and bathroom were “always” kept clean = .583<br>The area around your room was “always” quiet at night = .057<br><br>Responsiveness of hospital staff:<br>You “always” got help in getting to the bathroom or using a bedpan as soon as you wanted it = .318	

			After you pressed the call button, you "always" got help as soon as you wanted it = .216<br><br>Pain management:<br>Your pain was "always" well controlled = .590<br>The hospital staff "always" did everything they could to help you with your pain = .580<br><br>Communication about medicines:<br>Before giving you any new medicine, hospital staff "always" described possible side effects in a way you could understand = .145<br>Before giving you any new medicine, hospital staff "always" told you what the medicine was for = .566<br><br>Discharge information:<br>Hospital staff talked with you about whether you would have the help you needed when you left the hospital = -<br>You got information in writing about what symptoms or health problems to look out for after you left the hospital = **.032**<br>Staff took your preferences and those of your family into account in deciding what your	

			health care needs would be when you left the hospital = .054<br><br>Transition of care:<br>You had a good understanding of the things you were responsible for in managing your health = **.014**<br>You clearly understood the purpose for taking each of your medications = .897<br><br>Overall rating of a hospital:<br>Rated this hospital as a "9 or 10" out of 10 overall = 0.190<br>Would "definitely" recommend this hospital to friends and family = **0.017**	
Patient location:				
Mudumbai 2019* (4th)	Chi-square test comparing the readmitted patients (n=531 of 5514) with the overall cohort (n=5514), with two-sided p-value of ≤0.05 considered statistically significant		Overall % vs readmitted % (p-value) presented below:<br><br>Urban/rural status:<br>highly rural = 2.19 vs 1.32 (0.196)<br>rural = 56.29 vs 67.61 **(<0.001)**<br>urban = 41.51 vs 31.07 **(<0.001)**	Multivariate, extended Cox regression analysis (Hazard Ratio (95% CI)) for readmission from postdischarge days 0 to 30 - all independent variables were included simultaneously in the model:<br><br>Urban/rural status:<br>highly rural = 0.53 (0.25-1.13)<br>rural = **0.64 (0.53-0.78)**<br>urban = reference range
*Mixed cohort – primary and revision TKA combined<br><br>**Revision-only cohort				

S11 - Arroyo 2019 - Results from Exploratory Stratified Analysis

Arroyo 2019 – Results from Exploratory Stratified Analysis		
**Stratification:**	**Results - Associated with 30-day readmission (OR (95% CI)):**	Results - Not associated with 30-day readmission (OR (95% CI)):
By Primary Payer status	**Medicare only:**<br>Race (reference = White):<br>Black = 1.18 (1.12-1.24)<br>Other = 0.89 (0.83-0.96)<br>Missing = 0.74 (0.66-0.83)<br><br>Median income level (reference = first quartile):<br>Second quartile = 0.96 (0.92-1.00)<br>Third quartile = 0.94 (0.90-0.98)<br>Fourth quartile = 0.93 (0.89-0.97)<br><br>**Medicaid only:**<br>Race (reference = White):<br>Other = 0.80 (0.67-0.97)<br>Missing = 0.58 (0.40-0.82)<br><br>**Private only:**<br>Race (reference = White):<br>Black = 1.22 (1.13-1.33)<br>Missing = 0.82 (0.67-0.99)<br><br>**Other only:**<br>Race (reference = White):<br>Black = 1.30 (1.09-1.54)	**Medicare only:**<br>Race (reference = White):<br>Hispanic = 0.98 (0.93-1.03)<br><br>Median income level (reference = first quartile):<br>Missing = 0.91 (0.81-1.01)<br><br>**Medicaid only:**<br>Race (reference = White):<br>Black = 1.14 (0.98-1.33),<br>Hispanic = 0.92 (0.79-1.07);<br><br>Median income level (reference = first quartile):<br>Second quartile = 1.01 (0.88-1.17)<br>Third quartile = 0.98 (0.84-1.14)<br>Fourth quartile = 1.11 (0.91-1.35)<br>Missing = 1.18 (0.91-1.53)<br><br>**Private only:**<br>Race (reference = White):<br>Hispanic = 1.08 (0.96-1.20)<br>Other = 0.95 (0.85-1.06)<br><br>Median income level (reference = First quartile)<br>Second quartile = 0.97 (0.90-1.04)<br>Third quartile = 0.98 (0.91-1.05)<br>Fourth quartile = 0.99 (0.91-1.08)<br>Missing = 1.11 (0.94-1.31)<br><br>**Other only:**<br>Race (reference = White):<br>Hispanic = 1.11 (0.94-1.33)<br>Other = 1.18 (0.94-1.49)<br>Missing = 0.71 (0.48-1.07)<br><br>Median income level (reference = First quartile):<br>Second quartile = 1.08 (0.91-1.28)<br>Third quartile = 1.04 (0.88-1.24)<br>Fourth quartile = 1.07 (0.89-1.30)

		Missing = 1.41 (0.98-2.03)
By Race/Ethnicity	**White:**<br>Payer status (references = Private insurance):<br>Medicare = 1.19 (1.13-1.25)<br>Medicaid = 1.83 (1.65-2.02)<br><br>Median income level (reference = first quartile)<br>Second quartile = 0.94 (0.91-0.98)<br>Third quartile = 0.93 (0.89-0.97)<br>Fourth quartile = 0.92 (0.88-0.96)<br><br>**Black:**<br>Payer status (reference = Private insurance):<br>Medicare = 1.39 (1.23-1.56)<br>Medicaid = 1.59 (1.36-1.85)<br><br>Median income level (reference = First quartile):<br>Second quartile = 1.14 (1.03-1.26)<br><br>**Hispanic:**<br>Payer status (reference = Private insurance):<br>Medicare = 1.28 (1.15-1.43)<br>Medicaid = 1.48 (1.26-1.73)<br><br>**Other:**<br>Payer status (reference = Private insurance):<br>Medicare = 1.22 (1.06-1.40)<br>Medicaid = 1.28 (1.06-1.55)	**White:**<br>Payer status (reference = Private insurance):<br>Self-pay or no charge = 1.11 (0.89-1.39)<br>Other = 1.02 (0.95-1.10)<br><br>Median income level (reference = First quartile):<br>Missing = 0.94 (0.86-1.04)<br><br>**Black:**<br>Payer status (reference = Private insurance):<br>Self-pay or no charge = 1.22 (0.77-1.93)<br>Other = 1.01 (0.84-1.22)<br><br>Median income level (reference = First quartile):<br>Third quartile = 1.05 (0.95-1.16)<br>Fourth quartile = 1.00 (0.89-1.13)<br>Missing = 0.90 (0.70-1.15)<br><br>**Hispanic:**<br>Payer status (reference = Private insurance):<br>Self-pay or no charge = 0.94 (0.58-1.55)<br>Other = 1.09 (0.91-1.30)<br><br>Median income level (reference = First quartile):<br>Second quartile = 0.93 (0.83-1.03)<br>Third quartile = 0.90 (0.81-1.00)<br>Fourth quartile = 0.99 (0.88-1.12)<br>Missing = 1.10 (0.82-1.47)<br><br>**Other:**<br>Payer status (reference = Private insurance):<br>Self-pay or no charge = 1.00 (0.60-1.67)<br>Other = 1.26 (0.99-1.62)<br><br>Median income level (reference = First quartile):<br>Second quartile = 0.91 (0.79-1.06)<br>Third quartile = 0.87 (0.76-1.01)

		Fourth quartile = 0.86 (0.72-1.03)<br>Missing = 1.05 (0.71-1.56)
By Neighbourhood Income Quartile	**Quartile 1:**<br>Payer status (reference = Private insurance):<br>Medicare = 1.34 (1.24-1.45)<br>Medicaid = 1.64 (1.47-1.84)<br><br>Race (reference = White)<br>Black = 1.11 (1.04-1.20)<br>Missing = 0.68 (0.56-0.82)<br><br>**Quartile 2:**<br>Payer status (reference = Private insurance):<br>Medicare = 1.24 (1.17-1.33)<br>Medicaid = 1.68 (1.48-1.92)<br><br>Race (reference = White):<br>Black = 1.32 (1.21-1.44)<br>Missing = 0.79 (0.65-0.95)<br><br>**Quartile 3:**<br>Payer status (reference = Private insurance):<br>Medicare = 1.20 (1.12-1.29)<br>Medicaid = 1.50 (1.29-1.76)<br><br>Race (reference = White):<br>Black = 1.25 (1.15-1.36)<br>Other = 0.90 (0.82-0.99)<br><br>**Quartile 4:**<br>Payer status (reference = Private insurance):<br>Medicare = 1.17 (1.09-1.26)<br>Medicaid = 1.66 (1.39-1.97)<br><br>Race (reference = White):<br>Black = 1.21 (1.10-1.34)<br>Other = 0.87 (0.78-0.97)<br>Missing = 0.70 (0.58-0.86)	**Quartile 1:**<br>Payer status (reference = Private insurance):<br>Self-pay or no charge = 1.28 (0.82-2.02)<br>Other = 0.99 (0.87-1.14)<br><br>Race (reference = White):<br>Hispanic = 1.01 (0.93-1.09)<br>Other = 0.93 (0.81-1.06)<br><br>**Quartile 2:**<br>Payer status (reference = Private insurance):<br>Self-pay or no charge = 1.15 (0.79-1.69)<br>Other = 1.11 (0.99-1.25)<br><br>Race (reference = White):<br>Hispanic = 0.97 (0.89-1.06)<br>Other = 0.90 (0.80-1.02)<br><br>**Quartile 3:**<br>Payer status (reference = Private insurance):<br>Self-pay or no charge = 0.96 (0.58-1.58)<br>Other = 1.02 (0.91-1.15)<br><br>Race (reference = White):<br>Hispanic = 0.98 (0.89-1.07)<br>Missing = 0.84 (0.71-1.01)<br><br>**Quartile 4:**<br>Payer status (reference = Private insurance):<br>Self-pay or no charge = 1.30 (0.90-1.87)<br>Other = 1.02 (0.89-1.18);<br><br>Race (reference = White):<br>Hispanic = 1.08 (0.99-1.17)<br><br>**Missing:**<br>Payer status (reference = Private insurance):

		Medicare = 0.98 (0.78-1.24)<br>Medicaid = 1.37 (0.97-1.93)<br>Self-pay or no charge = 0.58 (0.32-1.05)<br>other = 1.25 (0.86-1.81)<br><br>Race (reference = White):<br>Black = 1.16 (0.88-1.54)<br>Hispanic = 1.19 (0.92-1.53)<br>Other = 1.07 (0.76-1.50)<br>Missing = 0.77 (0.40-1.45)
S	**Quartile 1:**<br>Payer status (reference = Private insurance):<br>Self-pay or no charge = 1.28 (0.82-2.02)<br>Other = 0.99 (0.87-1.14)<br><br>Race (reference = White):<br>Hispanic = 1.01 (0.93-1.09)<br>Other = 0.93 (0.81-1.06)<br><br>**Quartile 2:**<br>Payer status (reference = Private insurance):<br>Self-pay or no charge = 1.15 (0.79-1.69)<br>Other = 1.11 (0.99-1.25)<br><br>Race (reference = White):<br>Hispanic = 0.97 (0.89-1.06)<br>Other = 0.90 (0.80-1.02)<br><br>**Quartile 3:**<br>Payer status (reference = Private insurance):<br>Self-pay or no charge = 0.96 (0.58-1.58)<br>Other = 1.02 (0.91-1.15)<br><br>Race (reference = White):<br>Hispanic = 0.98 (0.89-1.07)<br>Missing = 0.84 (0.71-1.01)<br><br>**Quartile 4:**<br>Payer status (reference = Private insurance):<br>Self-pay or no charge = 1.30 (0.90-1.87)<br>Other = 1.02 (0.89-1.18)<br><br>Race (reference = White):	**Quartile 1:**<br>Payer status (reference = Private insurance):<br>Self-pay or no charge = 1.03 (0.77-1.37)<br>Other = 1.07 (0.96-1.19)<br><br>Race (reference = White):<br>Hispanic = 1.01 (0.94-1.09)<br>Other = 0.90 (0.82-1.00)<br><br>Median income level (reference = First quartile):<br>second quartile = 0.99 (0.93-1.05)<br>Third quartile = 0.99 (0.93–1.06)<br>Fourth quartile = 1.00 (0.93–1.07)<br>Missing 1.07 (0.92–1.23)<br><br>**Quartile 2:**<br>Payer status (reference = Private insurance):<br>Self-pay or no charge = 1.23 (0.75–2.00)<br>Other = 1.01 (0.90–1.14)<br><br>Race (reference = White):<br>Hispanic = 0.98 (0.91–1.05)<br>Other = 0.88 (0.77–1.01)<br><br>Median income level (reference = First quartile):<br>second quartile = 0.99 (0.92–1.06)<br>Third quartile = 0.95 (0.89–1.02)<br>Missing = 0.97 (0.81–1.16)<br><br>**Quartile 3:**<br>Payer status (reference = Private insurance):<br>Self-pay or no charge = 0.90 (0.58–1.39)<br>Other = 1.08 (0.94–1.24)

	Hispanic = 1.08 (0.99-1.17)<br><br>**Missing:**<br>Payer status (reference = Private insurance):<br>Medicare = 0.98 (0.78-1.24)<br>Medicaid = 1.37 (0.97-1.93)<br>Self-pay or no charge = 0.58 (0.32-1.05)<br>Other = 1.25 (0.86-1.81)<br><br>Race (reference = White):<br>Black = 1.16 (0.88-1.54)<br>Hispanic = 1.19 (0.92-1.53)<br>Other = 1.07 (0.76-1.50)<br>Missing = 0.77 (0.40-1.45)	Race (reference = White):<br>Hispanic = 1.05 (0.93–1.18)<br>Other = 0.98 (0.87–1.10)<br>Missing = 0.87 (0.72–1.04)<br><br>Median income level (reference = First quartile):<br>Second quartile = 0.94 (0.88–1.01)<br>Third quartile = 0.96 (0.89–1.03)<br>Fourth quartile = 0.94 (0.86–1.01)<br>Missing = 0.87 (0.71–1.05)
By Year (2007-2010)	**2007:**<br>Payer status (reference = Private insurance):<br>Medicare = 1.20 (1.09–1.32)<br>Medicaid = 1.67 (1.39–1.99)<br>Other = 1.22 (1.04–1.44)<br><br>Race (reference = White):<br>Black = 1.18 (1.05–1.33)<br>Other = 0.81 (0.69–0.96)<br>Missing = 0.57 (0.44–0.74)<br><br>Median income level (reference = First quartile):<br>Third quartile = 0.90 (0.83–0.99)<br><br>**2008:**<br>Payer status (reference = Private insurance):<br>Medicare = 1.35 (1.23–1.47)<br>Medicaid = 1.60 (1.33–1.93)<br><br>Race (reference = White):<br>Black = 1.30 (1.16–1.45)<br><br>Median income level (reference = First quartile):<br>Third quartile = 0.92 (0.85–1.00)<br><br>**2009:**<br>Payer status (reference = Private insurance):<br>Medicare = 1.16 (1.07–1.27)<br>Medicaid = 1.63 (1.36–1.95)	**2007:**<br>Payer status (reference = Private insurance):<br>Self-pay or no charge = 1.10 (0.69–1.75)<br><br>Race (reference = White):<br>Hispanic = 1.01 (0.89–1.13)<br><br>Median income level (reference = First quartile):<br>Second quartile = 0.93 (0.85–1.02)<br>Fourth quartile = 0.93 (0.84–1.02)<br>Missing = 0.84 (0.67–1.06)<br><br>**2008:**<br>Payer status (reference = Private insurance):<br>Self-pay or no charge = 1.30 (0.87–1.94)<br>Other = 1.06 (0.91–1.24)<br><br>Race (reference = White):<br>Hispanic = 1.08 (0.96–1.21)<br>Other = 0.93 (0.80–1.09)<br>Missing = 0.79 (0.62–1.01)<br><br>Median income level (reference = First quartile):<br>Second quartile = 0.96 (0.88–1.05)<br>Fourth quartile = 0.91 (0.82–1.01)<br>Missing = 1.02 (0.83–1.26)<br><br>**2009:**<br>Payer status (reference = Private insurance):

	Race (reference = White):<br>Black = 1.22 (1.09–1.38)<br>Missing = 0.76 (0.60–0.97)<br><br>**2010:**<br>Payer status (reference = Private insurance):<br>Medicare = 1.17 (1.07–1.27)<br>Medicaid = 1.57 (1.34–1.84)<br><br>Race (reference = White):<br>Black = 1.16 (1.04–1.29)<br>Other = 0.83 (0.71–0.97)<br><br>Median income level (reference = First quartile):<br>Second quartile = 0.90 (0.83–0.98)<br>Third quartile = 0.89 (0.81–0.98)<br>Fourth quartile = 0.89 (0.82–0.98)	Self-pay or no charge = 1.18 (0.79–1.77)<br>Other = 0.98 (0.84–1.14)<br><br>Race (reference = White):<br>Hispanic = 1.05 (0.95–1.17)<br>Other = 0.89 (0.77–1.03)<br><br>Median income level (reference = First quartile):<br>Second quartile = 1.04 (0.96–1.13)<br>Third quartile = 0.97 (0.90–1.05)<br>Fourth quartile = 0.96 (0.88–1.04)<br>Missing = 1.08 (0.90–1.30)<br><br>**2010:**<br>Payer status (reference = Private insurance):<br>Self-pay or no charge = 0.97 (0.69–1.35)<br>Other = 1.03 (0.89–1.19)<br><br>Race (reference = White):<br>Hispanic = 0.98 (0.89–1.08)<br><br>Median income level (reference = First quartile):<br>Missing = 1.05 (0.86–1.29)
By Year (2011-2014)	**2011:**<br>Payer status (reference = Private insurance):<br>Medicare = 1.25 (1.14–1.37)<br>Medicaid = 1.59 (1.35–1.88)<br><br>Race (reference = White)<br>Black = 1.32 (1.17–1.50)<br><br>**2012:**<br>Payer status (reference = Private insurance):<br>Medicare = 1.25 (1.11–1.40)<br>Medicaid = 1.61 (1.30–2.00)<br><br>**2013:**<br>Payer status (reference = Private insurance):<br>Medicare = 1.24 (1.10–1.39)<br>Medicaid = 1.68 (1.38–2.04)<br>Self-pay or no charge = 1.49 (1.05–2.11)<br><br>Race (reference = White):	**2011:**<br>Payer status (reference = Private insurance):<br>Self-pay or no charge = 1.03 (0.55–1.90)<br>Other = 0.98 (0.84–1.13)<br><br>Race (reference = White):<br>Hispanic = 1.02 (0.91–1.13)<br>Other = 0.92 (0.79–1.08)<br>Missing = 0.79 (0.62–1.00)<br><br>Median income level (reference = First quartile):<br>Second quartile = 0.96 (0.89–1.04)<br>Third quartile = 0.98 (0.90–1.06)<br>Fourth quartile = 0.94 (0.86–1.03)<br>Missing = 0.96 (0.78–1.20)<br><br>**2012:**<br>Payer status (reference = Private insurance):<br>Self-pay or no charge = 0.78 (0.40–1.51)<br>Other = 0.92 (0.75–1.13)

	Black = 1.19 (1.07–1.33)<br><br>**2014:**<br>Payer status (reference = Private insurance):<br>Medicare = 1.21 (1.11–1.32)<br>Medicaid = 1.55 (1.36–1.77)<br><br>Race (reference - White):<br>Black = 1.20 (1.05–1.37)<br>Other = 0.78 (0.65–0.93)	Race (reference = White):<br>Black = 1.07 (0.95–1.20)<br>Hispanic = 1.11 (0.96–1.27)<br>Other = 0.95 (0.81–1.11)<br>Missing = 0.99 (0.66–1.48)<br><br>Median income level (reference = First quartile):<br>Second quartile = 0.99 (0.66–1.48)<br>Third quartile = 1.01 (0.90–1.13)<br>Fourth quartile = 1.00 (0.90–1.11)<br>Missing = 0.94 (0.71–1.25)<br><br>**2013:**<br>Payer status (reference = Private insurance):<br>Other = 0.98 (0.81–1.17)<br><br>Race (reference = White):<br>Hispanic = 1.01 (0.86–1.18)<br>Other = 1.07 (0.91–1.25)<br>Missing = 0.79 (0.54–1.13)<br><br>Median income level (reference = First quartile):<br>Second quartile = 1.02 (0.92–1.13)<br>Third quartile = 0.96 (0.86–1.07)<br>Fourth quartile = 0.96 (0.85–1.07)<br>Missing = 0.85 (0.62–1.17)<br><br>**2014:**<br>Payer status (reference = Private insurance):<br>Self-pay or no charge = 1.16 (0.71–1.89)<br>Other = 1.18 (0.94–1.47)<br><br>Race (reference = White):<br>Hispanic = 0.98 (0.84–1.13)<br>Missing = 0.89 (0.59–1.33)<br><br>Median income level (reference = First quartile):<br>Second quartile = 1.01 (0.91–1.12)<br>Third quartile = 1.00 (0.90–1.12)<br>Fourth quartile = 1.07 (0.95–1.20)<br>Missing = 1.00 (0.75–1.34)

By State	**California:**<br>Payer status (reference = Private insurance):<br>Medicare = 1.24 (1.14–1.34)<br>Medicaid = 1.66 (1.48–1.86)<br><br>Race (reference = White):<br>Black = 1.40 (1.26–1.55)<br>Missing = 0.69 (0.60–0.79)<br><br>**Florida:**<br>Payer status (reference = Private insurance):<br>Medicare = 1.14 (1.09–1.20)<br>Medicaid = 1.75 (1.58–1.93)<br>Self-pay or no charge = 1.27 (1.06–1.53)<br><br>Race (reference = White):<br>Other = 0.91 (0.84–0.99)<br>missing = 0.79 (0.69–0.91)<br><br>Median income level (reference = First quartile):<br>Second quartile = 0.94 (0.91–0.97)<br>Third quartile = 0.94 (0.90–0.97)<br>Fourth quartile = 0.89 (0.85–0.92)<br><br>**Maryland:**<br>Payer status (reference = Private insurance):<br>Other = 0.72 (0.54–0.96)<br><br>Race (reference = White):<br>Black = 1.38 (1.13–1.69)<br><br>Median income level (reference = First quartile):<br>Fourth quartile = 1.23 (1.02–1.47)<br><br>**New York:**<br>Payer status (reference = Private insurance):<br>Medicare = 1.27 (1.19–1.35)<br>Medicaid = 1.49 (1.34–1.66)<br><br>Race (reference = White):<br>Black = 1.17 (1.09–1.25)<br>Other = 0.92 (0.85–0.99)	**California:**<br>Payer status (reference = Private insurance):<br>Self-pay or no charge = 0.70 (0.37–1.32)<br>Other = 1.02 (0.92–1.14)<br><br>Race (reference = White):<br>Hispanic = 0.97 (0.91–1.04)<br>Other = 0.89 (0.78–1.01)<br><br>Median income level (reference = First quartile):<br>Second quartile = 0.98 (0.91–1.05)<br>Third = quartile = 0.95 (0.89–1.02)<br>Fourth quartile = 0.96 (0.89–1.03)<br>Missing = 1.11 (0.94–1.30)<br><br>**Florida:**<br>Payer status (reference = Private insurance):<br>Other = 1.06 (0.94–1.20)<br><br>Race (reference = White):<br>Hispanic = 1.00 (0.92–1.07)<br><br>Median income level (reference = First quartile):<br>Missing = 0.90 (0.80–1.01)<br><br>**Maryland:**<br>Payer status (reference = Private insurance):<br>Medicare = 1.16 (0.99–1.37)<br>Medicaid = 1.33 (0.96–1.85)<br>Self-pay or no charge = 0.79 (0.23–2.66)<br><br>Race (reference = White):<br>Hispanic = 1.10 (0.62–1.93)<br>Other = 0.96 (0.62–1.48)<br>Missing = 0.89 (0.74–1.08)<br><br>Median income level (reference = First quartile):<br>Second quartile = 1.14 (0.95–1.36)<br>Third quartile = 0.97 (0.80–1.17)<br>Missing = 0.99 (0.46–2.12)<br><br>**New York:**<br>Payer status (reference = Private insurance):

		Self-pay or no charge = 0.96 (0.75–1.21)<br>Other = 1.09 (0.99–1.19)<br>Race (reference = White):<br>Hispanic = 1.02 (0.94–1.12)<br>Missing = 0.96 (0.74–1.25)<br><br>Median income level (reference = First quartile):<br>Second quartile = 0.97 (0.91–1.04)<br>Third quartile = 0.93 (0.86–1.00)<br>Fourth quartile = 0.95 (0.88–1.02)<br>Missing = 0.95 (0.79–1.14)

S12 – Results for other types of readmission, adjusted analysis

Other types of readmission				
**Study ID**	**Effect size estimates:**		**Significance tests on matched cohorts:**	
	**Effect measure calculated (measure of confidence):**	**Result (**in **bold =** confidence intervals do not include null value**):**	**Method:**	**Result:**
Surgical readmission**				
Ali 2019 (1st)	OR (95% CI)	**Demographics:**<br><br>Age group (60-64):<br>0-39 = **1.31 (1.04-1.63)**<br>40-44 = **1.24 (1.05-1.46)**<br>45-49 = **1.18 (1.06-1.32)**<br>50-54 = 1.04 (0.96-1.13)<br>55-59 = 1.04 (0.98-1.11)<br>65-69 = 1.02 (0.97-1.07)<br>70-74 = 1.05 (1.00-1.11; p-value = 0.0622)<br>75-79 = **1.17 (1.11-1.23)**<br>80-84 = **1.22 (1.15-1.29)**<br>85-89 = **1.40 (1.29-1.51)**<br>90+ = **1.25 (1.04-1.49)**<br><br>Male sex = **1.21 (1.17-1.24)**<br><br>SES quintile (1 (least deprived)):<br>2 = 1.02 (0.98-1.07)<br>3 = **1.06 (1.01-1.11)**<br>4 = **1.12 (1.06-1.17)**<br>5 = **1.24 (1.18-1.31)**<br><br>Ethnicity (white):<br>Mixed ethnicity = 1.04 (0.80-1.35)<br>Asian/Asian British = 0.95 (0.88-1.03)<br>Black/Black British = **1.19 (1.06-1.33)**<br>Other ethnic group = 0.91 (0.76-1.10)<br>Not known or stated race = **0.66 (0.62-0.70)**<br><br>**Comorbidities:**<br><br>Diabetes mellitus = 1.02 (0.97-1.06)<br>Hypertension = 1.00 (0.97-1.03)	N/A	N/A

		Arrhythmias = 1.05 (1.00-1.10; p-value = 0.0676)<br>Valvular heart disease = **1.11 (1.02-1.21)**<br>Congestive heart failure = 1.00 (0.90-1.11)<br>Peripheral vascular disease = **1.15 (1.04-1.28)**<br>Chronic pulmonary disease = **1.29 (1.24-1.34)**<br>Pulmonary circulatory disease = 1.16 (1.00-1.35; p-value = 0.053)<br>Metastases = 0.88 (0.61-1.29)<br>Renal disease = **0.91 (0.85-0.98)**<br>Dementia = 1.22 (0.98-1.52)<br>Psychoses = **1.51 (1.14-2.00)**<br>Alcohol abuse = 1.07 (0.95-1.20)<br>Drug abuse = 0.96 (0.58-1.59)<br>Depression = **1.27 (1.19-1.37)**<br>Other mental health disorder = **1.39 (1.22-1.59)**<br>Living alone = 1.05 (0.94-1.18)<br>Liver disease = **1.46 (1.26-1.70)**<br>Peptic ulcer disease = 0.93 (0.76-1.13)<br>Paraplegia = **1.40 (1.13-1.73)**<br>Anaemia due to blood loss = 0.98 (0.55-1.74)<br>Iron deficiency anaemia = 1.02 (0.92-1.14)<br>Coagulopathy = **1.28 (1.07-1.52)**<br>Recent weight loss = 0.95 (0.73-1.23)<br>Fluid balance abnormality = 0.96 (0.86-1.06)<br>Hypothyroidism = **1.07 (1.01-1.13)**<br>Obesity = **1.18 (1.12-1.23)**<br>Other neurological disorder = **1.40 (1.29-1.52)**<br>Rheumatological disorder = 1.00 (0.94-1.07)<br>Previous pneumonia = 0.88 (0.72-1.06)<br>Previous stroke = 0.74 (0.53-1.03)<br>Previous AMI = 0.67 (0.45-1.01)<br>Cancer diagnosis = **0.87 (0.77-0.98)**<br><br>**Miscellaneous:**<br><br>Number of prior emergency admissions (0):<br>1 = **1.41 (1.35-1.48)**<br>2 = **1.63 (1.48-1.79)**<br>3+ = **2.09 (1.82-2.40)**		
		Return-to-theatre readmission***		

Ali 2019 (1st)	OR (95% CI)	**Demographics:**<br><br>Age group (60-64):<br>0-39 = **2.04 (1.31-3.19)**<br>40-44 = 1.22 (0.80-1.85)<br>45-49 = **1.48 (1.15-1.91)**<br>50-54 = **1.22 (1.00-1.49; p-value = 0.046)**<br>55-59 = **1.21 (1.03-1.42)**<br>65-69 = 1.00 (0.87-1.14)<br>70-74 = 1.06 (0.93-1.21)<br>75-79 = 1.05 (0.91-1.20)<br>80-84 = 1.14 (0.98-1.33)<br>85-89 = 1.15 (0.94-1.41)<br>90+ = 0.59 (0.32-1.09)<br><br>Male sex = **1.66 (1.54-1.79)**<br><br>SES quintile (1 (least deprived)):<br>2 = 0.97 (0.87-1.09)<br>3 = 0.99 (0.89-1.12)<br>4 = 1.07 (0.95-1.21)<br>5 = 1.06 (0.92-1.21)<br><br>Ethnicity (white):<br>Mixed ethnicity = 1.19 (0.63-2.22)<br>Asian/Asian British = 1.04 (0.85-1.27)<br>Black/Black British = 0.99 (0.71-1.38)<br>Other ethnic group = 0.65 (0.37-1.16)<br>Not known or stated race = **0.70 (0.60-0.83)**<br><br>**Comorbidities:**<br><br>Diabetes mellitus = 0.99 (0.89-1.10)<br>Hypertension = 0.98 (0.91-1.06)<br>Arrhythmias = 1.10 (0.97-1.24)<br>Valvular heart disease = **1.29 (1.05-1.58)**<br>Congestive heart failure = 0.81 (0.61-1.07)<br>Peripheral vascular disease = 0.91 (0.68-1.22)<br>Chronic pulmonary disease = **1.14 (1.03-1.26)**<br>Pulmonary circulatory disease = 1.14 (0.80-1.62)<br>Metastases = 1.59 (0.76-3.33)	N/A	N/A

		Renal disease = 0.99 (0.83-1.18)<br>Dementia = **1.71 (1.10-2.65)**<br>Psychoses = **2.52 (1.49-4.24)**<br>Alcohol abuse = 1.18 (0.89-1.56)<br>Drug abuse = 1.46 (0.54-3.96)<br>Depression = **1.34 (1.12-1.60)**<br>Other mental health disorder = 1.31 (0.92-1.85)<br>Living alone = 1.01 (0.75-1.36)<br>Liver disease = 1.30 (0.89-1.91)<br>Peptic ulcer disease = 0.89 (0.53-1.48)<br>Paraplegia = 1.11 (0.64-1.93)<br>Anaemia due to blood loss = 1.03 (0.25-4.17)<br>Iron deficiency anaemia = 1.12 (0.86-1.46)<br>Coagulopathy = 0.95 (0.59-1.55)<br>Recent weight loss = 0.63 (0.28-1.41)<br>Fluid balance abnormality = 0.91 (0.70-1.17)<br>Hypothyroidism = 1.08 (0.94-1.25)<br>Obesity = **1.36 (1.22-1.53)**<br>Other neurological disorder = **1.57 (1.30-1.89)**<br>Rheumatological disorder = 1.04 (0.88-1.22)<br>Previous pneumonia = 1.05 (0.67-1.64)<br>Previous stroke = 0.65 (0.27-1.58)<br>Previous AMI = 0.37 (0.09-1.46)<br>Cancer diagnosis = 0.79 (0.58-1.06)<br><br>**Miscellaneous:**<br><br>Number of prior emergency admissions (0):<br>1 = **1.35 (1.19-1.52)**<br>2 = **1.32 (1.02-1.70)**<br>3+ = **2.11 (1.54-2.90)**		
TKA-specific readmission****				
D'Apuzzo 2017 (3rd)	OR (95% CI)	**Demographics:**<br><br>Age group (vs. <65 yr):<br>65-75 yr = **0.78 (0.69-0.89)**<br>76-85 yr = 0.94 (0.82-1.08)<br>>85 yr = **1.28 (1.02-1.61)**<br><br>Sex:<br>Male vs. female = **1.34 (1.23, 1.46)**	N/A	N/A

		Race/ethnicity (vs. white):<br>Black = **1.20 (1.05-1.37)**<br>Hispanic = 1.00 (0.84-1.20)<br>Asian = 0.65 (0.42-1.02)<br>Other = 1.03 (0.85-1.23)<br>Missing = 1.15 (0.92-1.44)<br><br>Insurance status (vs. private):<br>Medicare = **1.31 (1.16-1.49)**<br>Medicaid = **1.40 (1.16-1.69)**<br>Workers' Compensation = 1.07 (0.86-1.33)<br>Other = 1.20 (0.90-1.58)<br><br>In-hospital complications:<br>Medical = **2.31 (1.99-2.68)**<br>Surgical = **2.70 (2.28-3.20)**<br><br>**Comorbidities:**<br><br>Congestive heart failure = **1.41 (1.14-1.75)**<br>Valvular disease = 1.07 (0.89-1.28)<br>Pulmonary circulation disorder = **1.74 (1.24-2.44)**<br>Peripheral vascular disorder = 1.12 (0.86-1.46)<br>Paralysis = 1.64 (0.77-3.49)<br>Other neurological disorder = 1.17 (0.92-1.48)<br>Chronic pulmonary disease = **1.16 (1.04-1.29)**<br>Diabetes = **1.11 (1.01-1.23)**<br>Renal failure = **1.47 (1.20-1.81)**<br>Liver disease = 0.99 (0.65-1.50)<br>Peptic ulcer disease excluding bleeding = 0.92 (0.48-1.79)<br>Lymphoma = 1.94 (0.80-4.73)<br>Solid tumour without metastasis = 1.04 (0.78-1.38)<br>Rheumatoid arthritis/collagen vascular disease = 1.11 (0.68-1.81)<br>Coagulopathy = **1.56 (1.14-2.13)**<br>Obesity = **1.25 (1.12-1.38)**<br>Weight loss = 1.29 (0.53-3.19)<br>Fluid and electrolyte disorders = 1.07 (0.84-1.37)		

		Deficiency anaemias = 1.01 (0.87-1.19)<br>Alcohol abuse = 0.93 (0.59-1.45)<br>Drug abuse = 1.36 (0.83-2.23)<br>Psychoses = 1.09 (0.78-1.52)<br>Depression = **1.35 (1.19-1.53)**<br>Hypertension (uncomplicated and complicated combined) = 1.09 (1.00-1.19; p = 0.065)<br><br>**Miscellaneous:**<br>Bilateral TKA = **1.22 (1.02-1.45)**		
Rudasill 2019 (2nd)	OR (95% CI)	TKA-related readmission<br>INR class:<br>INR >1-1.25 = unable to calculate<br>**INR >1.25-1.5 = 1.59 (1.14-2.22)**<br>**INR > 1.5 = 1.99 (1.07-3.70)**	N/A	N/A
Expanded TKA-specific readmission*****				
D'Apuzzo 2017 (3rd)	OR (95% CI)	**Demographics:**<br><br>All-cause:<br>Age group (vs. <65 yr):<br>65-75 yr = **0.84 (0.78-0.91)**<br>76-85 yr = 1.01 (0.93-1.10)<br>>85 yr = **1.32 (1.15-1.52)**<br><br>Sex:<br>Male vs. female = **1.41 (1.34-1.49)**<br><br>Race/ethnicity (vs. white):<br>Black = **1.24 (1.14-1.34)**<br>Hispanic = **1.18 (1.07-1.30)**<br>Asian = 0.86 (0.68-1.09)<br>Other = 1.02 (0.91-1.14)<br>Missing = 1.08 (0.93-1.24)<br><br>Insurance status (vs. private):<br>Medicare = **1.26 (1.17-1.36)**<br>Medicaid = **1.40 (1.26-1.57)**<br>Workers' Compensation = 1.10 (0.97-1.25)<br>Other = 1.09 (0.92-1.30)<br><br>In-hospital complications:<br>Medical = **1.85 (1.67-2.05)**	N/A	N/A

		Surgical = **2.16 (1.92-2.43)**<br><br>**Comorbidities:**<br><br>Congestive heart failure = **1.53 (1.34-1.74)**<br>Valvular disease = 1.05 (0.93-1.17)<br>Pulmonary circulation disorder = **1.29 (1.01-1.66)**<br>Peripheral vascular disorder = 1.17 (0.99-1.37)<br>Paralysis = 1.30 (0.77-2.19)<br>Other neurological disorder = **1.28 (1.12-1.47)**<br>Chronic pulmonary disease = **1.32 (1.24-1.41)**<br>Diabetes = **1.08 (1.02-1.15)**<br>Renal failure = **1.32 (1.16-1.50)**<br>Liver disease = **1.40 (1.12-1.74)**<br>Peptic ulcer disease excluding bleeding = 1.12 (0.77-1.63)<br>Lymphoma = **1.90 (1.09-3.34)**<br>Solid tumour without metastasis = 0.96 (0.80-1.15)<br>Rheumatoid arthritis/collagen vascular disease = 1.22 (0.89-1.67)<br>Coagulopathy = **1.48 (1.21-1.80)**<br>Obesity = **1.14 (1.07-1.21)**<br>Weight loss = 0.99 (0.52-1.89)<br>Fluid and electrolyte disorders = 1.07 (0.92-1.24)<br>Deficiency anaemias = 1.06 (0.96-1.16)<br>Alcohol abuse = 1.03 (0.79-1.33)<br>Drug abuse = 1.34 (1.00-1.79; p = 0.054)<br>Psychoses = **1.31 (1.09-1.58)**<br>Depression = **1.27 (1.17-1.37)**<br>Hypertension (uncomplicated and complicated combined) = 1.03 (0.98-1.09)<br><br>**Miscellaneous:**<br><br>Bilateral TKA = 0.89 (0.79-1.01)		
Readmission Due To Surgical Site Infection******				
Anthony 2018* (2nd)	OR (95% CI)	**Demographics:**<br><br>Age (Reference category: <18):	N/A	N/A

		[18, 30) = **0.114 (0.02–0.63)**<br>[30, 40) = 1.121 (0.5–2.53)<br>[40, 50) = 1.076 (0.5–2.32)<br>[50, 60) = 0.977 (0.46–2.1)<br>[60, 70) = 0.771 (0.36–1.66)<br>[70, 80) = 0.69 (0.32–1.49)<br>80+ = 0.633 (0.29–1.37)<br><br>Female sex = **0.559 (0.53–0.59)**<br><br>Primary Payer (reference category = Medicare):<br>Self-pay = 1.009 (0.69–1.47)<br>No charge = 0.613 (0.21–1.76)<br>Other = 0.961 (0.84–1.1)<br>Private insurance = **0.679 (0.63–0.73)**<br>Medicaid = **1.489 (1.32-1.68)**<br><br>Patient location:<br>Rural (vs urban) = **0.868 (0.8–0.94**)<br><br>**Comorbidities:**<br><br>Hypertension = **1.189 (1.11-1.27)**<br>Obesity = **1.182 (1.11-1.26)**<br>Diabetes = **1.122 (1.05-1.2)**		
***Mixed cohort – revision and primary TKA combined; **Defined in Ali 2019 as a readmission with a primary ICD-10 code related to the surgical site; ***defined in Ali 2019 as a readmission where the patient returned to theatre during the readmission; ****defined in D'Apuzzo 2017 as readmission due to any of the eight diagnoses the CMS (Centers for Medicare & Medicaid Services) considers to be TKA-specific complications (acute myocardial infarction, pneumonia, sepsis/septicaemia/shock, surgical site bleeding, pulmonary embolism, death, mechanical complications, periprosthetic joint infection/wound infection); Rudasil 2019 did not explicitly define 'TKA-related readmission'; *****defined in D'Apuzzo 2017 as the list of eight diagnoses defined as TKA-specific by the CMS, plus an additional 22 diagnoses (arterial embolism, bleeding complication, cardiac complication, central nervous system complication other than stroke, deep vein thrombosis, genitourinary complication, haematological complication, iatrogenic complication other than periprosthetic, mechanical complication, other complications, nosocomial infection other than periprosthetic, periprosthetic fracture or dislocation, periprosthetic infection, postoperative fever, postoperative pain, postoperative stiffness, pulmonary complication other than pulmonary embolism, soft-tissue complication, stroke, unspecified periprosthetic infection, wound complication, revision); ******defined in Anthony as readmission with a primary diagnosis of postoperative surgical site infection, identified using ICD-9_CM codes**				

S13 – Results for other types of readmission, unadjusted and secondary analyses

Other types of readmission				
**Study ID (overall risk of bias quartile – arranged in descended order from lowest to highest)**	**Methods used**	**Unadjusted effect estimates**	**Univariate significance test**	**Additional analyses**
		**Result (**in **bold =** confidence intervals do not include null value**)**	**Result (**in **bold =** significant p-value**)**	**Result (**in **bold =** confidence intervals do not include null value or significant p-value (whichever applicable)**)**
Surgical readmission***				
Ali 2019 (1st)	N/A	N/A	N/A	Population attributable fraction was calculated for each of the strongest predictors for readmission. This is the proportion of the incidence rate in the whole population that is due to have that particular comorbidity.<br><br>Pyschoses = 0.2%<br>Liver disease = 0.5%<br>Other neurological disorder = 0.6%<br>Paraplegia = 0.2%<br>Other mental health disorder = 0.5%
Return-to-theatre readmission****				
Ali 2019 (1st)	N/A	N/A	N/A	Population attributable fraction was calculated for each of the strongest predictors for readmission. This is the proportion of the incidence rate in the whole population that is due to have that particular comorbidity.<br><br>Pyschoses = 0.7%<br>Dementia = 0.5%<br>Other neurological disorder = 2.2%<br>Obesity = 4.7%<br>Depression = 1.5%
TKA-specific readmission*****				
Rudasill 2019 (2nd)	Chi-square	N/A	TKA-related readmission (increases with increasing INR class: **p <0.001**)	N/A

*Mixed cohort – primary and revision TKA combined; **Revision-only cohort; ***Defined in Ali 2019 as a readmission with a primary ICD-10 code related to the surgical site; ****defined in Ali 2019 as a readmission where the patient returned to theatre during the readmission; *****Rudasil 2019 did not explicitly define 'TKA-related readmission'

S14 - Meta-analysis R code

```
# # # # # # # # # # # # # # # # # # # # # # # # # # # # #
# R-script: meta_analysis_code.R
# Project: Meta-analysis
#
# Data used: adjusted effects estimates
# Data created: N/A (Forest plots only)
#
# Date: 01/09/2020
# Author: D. Gould
# R version: R version 3.5.3 (2019-03-11) -- "Great Truth"
#
# Purpose: Random effects meta-analysis and Forest plot generation
# # # # # # # # # # # # # # # # # # # # # # # # # # # # #
# Install tidyverse and meta packages, if not yet installed:
# install(tidyverse)
# install(meta)

# Load Packages
library(tidyverse)
library(meta)
```

```
#########################################
# >insert prognostic factor name<
#########################################
df <- read.csv("prognostic_factor.csv")
df <- df %>%
  rename(
    study_id = ï..study_id
  )

# Log transform all effect size data, so it can be used in metagen() function
df$effect_size <- log(df$effect_size)
df$confidence_interval_lower <- log(df$confidence_interval_lower)
df$confidence_interval_upper <- log(df$confidence_interval_upper)

# Calculate standard error
df$seTE <- (df$confidence_interval_upper - df$confidence_interval_lower)/3.92
output <- metagen(effect_size,
                  seTE,
                  studlab = study_id,
                  method.tau = "SJ",
                  sm = "OR",
                  data = df)
```

```
# Generate Forest plot
forest(output,
       layout = "JAMA")
```

S15 - Critical Appraisal

Critical Appraisal												
Study ID	***JBI 1.**	**JBI 2.**	**JBI 3.**	**JBI 4.**	**JBI 5.**	**JBI 7.**	**JBI 8.**	**JBI 9.**	**JBI 10.**	**JBI 11.**	**Overall risk of bias score (quartile; arranged in descending order)**	**Evidence of selective reporting**
Abola 2018	Y	Y	Y	Y	Y	Y	Y	N	U	Y	15% (1st)	No
Ali 2019	Y	Y	Y	Y	Y	Y	Y	N	Y	U	15% (1st)	No
Belmont 2016	Y	Y	Y	Y	Y	Y	Y	N	Y	U	15% (1st)	No
Bovonratwet 2019	Y	Y	Y	Y	Y	Y	Y	N	Y	U	15% (1st)	Yes
Bovonratwet 2018	Y	Y	Y	Y	Y	Y	Y	N	Y	U	15% (1st)	No
Courtney 2018	Y	Y	Y	Y	Y	Y	Y	N	Y	U	15% (1st)	No
Jorgensen 2017	Y	Y	Y	Y	Y	Y	Y	Y	Y	Y	0% (1st)	No
Kester 2016	Y	Y	Y	Y	Y	Y	Y	N	Y	U	15% (1st)	Yes
Kim 2019	Y	Y	Y	Y	Y	Y	Y	N	Y		10% (1st)	No
Kurtz 2016	Y	Y	Y	Y	Y	Y	Y	U	Y	U	10% (1st)	No

Lehtonen 2018	Y	Y	Y	Y	Y	Y	Y	N	Y	U	15% (1st)	Yes
Pugely 2013	Y	Y	Y	Y	Y	Y	Y	N	Y	U	15% (1st)	Yes
Roth 2019	Y	Y	Y	Y	Y	Y	Y	N	U	Y	15% (1st)	No
Urish 2018	Y	Y	Y	Y	Y	Y	Y	U	Y	U	10% (1st)	No
Sodhi and Anis et al 2019	Y	Y	Y	Y	Y	Y	Y	N	U	Y	15% (1st)	Yes
Gwam 2020	Y	Y	Y	Y	Y	Y	Y	N	U	Y	15% (1st)	No
Alvi 2015	Y	Y	Y	Y	Y	Y	Y	N	U	U	20% (2nd)	Yes
Anthony 2018	Y	U	U	U	Y	Y	Y	U	Y	Y	20% (2nd)	No
Curtis 2019	Y	Y	Y	Y	Y	Y	Y	N	U	U	20% (2nd)	Yes
Curtis 2018	Y	Y	Y	Y	Y	Y	Y	N	U	U	20% (2nd)	No
George 2018	Y	Y	Y	Y	Y	Y	Y	N	U	U	20% (2nd)	No
Hart 2016	Y	Y	Y	Y	Y	Y	Y	N	U	U	20% (2nd)	Yes
Liao 2016	Y	Y	Y	Y	Y	U	Y	N	Y	U	20% (2nd)	Yes
Ottesen 2018	Y	Y	Y	Y	Y	Y	Y	N	U	U	20% (2nd)	Yes

Patterson 2018	Y	Y	Y	Y	Y	Y	Y	N	U	U	20% (2nd)	No
Runner 2017	Y	Y	Y	Y	Y	Y	Y	N	U	U	20% (2nd)	Yes
Suleiman 2015	Y	Y	Y	Y	Y	Y	Y	N	U	U	20% (2nd)	Yes
Sutton 2016	Y	Y	Y	Y	Y	Y	Y	N	U	U	20% (2nd)	Yes
Webb 2017	Y	Y	Y	Y	Y	Y	Y	N	U	U	20% (2nd)	Yes
Welsh 2017	Y	Y	Y	Y	Y	Y	Y	N	U	U	20% (2nd)	No
Zusmanovic 2018	Y	Y	Y	Y	Y	Y	Y	N	U	U	20% (2nd)	Yes
Arroyo 2019	Y	Y	Y	Y	Y	Y	Y	N	U	U	20% (2nd)	Yes
Lovecchio 2014	Y	Y	Y	Y	Y	Y	Y	N	U	U	20% (2nd)	No
Ross 2020	Y	Y	Y	Y	Y	Y	Y	N	U	U	20% (2nd)	Yes
Rudasill 2019	Y	Y	Y	Y	Y	Y	Y	N	N	Y	20% (2nd)	Yes
Abdulla 2020	Y	Y	Y	Y	Y	Y	Y	N	U	U	20% (2nd)	No
Anderson 2020	Y	Y	Y	Y	Y	Y	Y	N	U	U	20% (2nd)	No
Bovonratwet 2020	Y	Y	Y	Y	Y	Y	Y	N	U	U	20% (2nd)	No

Patel 2020	Y	Y	Y	Y	Y	Y	Y	N	U	U	20% (2nd)	No
Sloan 2020	Y	Y	Y	Y	Y	Y	Y	N	U	U	20% (2nd)	No
Tang 2019	Y	U	U	Y	Y	U	Y	N	N	Y	35% (3rd)	Yes
D'Apuzzo 2017	Y	Y	Y	U	U	Y	Y	N	U	U	30% (3rd)	Yes
Jorgensen 2013	Y	Y	Y	Y	N	Y	Y	Y	U	N	25% (3rd)	No
Ramos 2014	Y	Y	Y	Y	Y	Y	Y	N	N	U	25% (3rd)	No
Ricciardi 2017	Y	Y	Y	Y	Y	Y	Y	N	N	U	25% (3rd)	No
Saucedo 2014	Y	Y	Y	Y	Y	U	Y	N	U	U	25% (3rd)	Yes
Singh 2013	Y	Y	Y	Y	Y	U	Y	N	U	U	25% (3rd)	No
Siracuse 2017	Y	Y	Y	Y	Y	U	Y	N	U	U	25% (3rd)	Yes
Sodhi and Mont et al 2019	Y	Y	Y	Y	Y	U	Y	N	U	U	25% (3rd)	No
Weick 2018	Y	Y	Y	Y	Y	Y	Y	N	N	U	25% (3rd)	Yes
Workman 2019	Y	Y	Y	Y	Y	Y	Y	N	N	U	25% (3rd)	Yes
Yohe 2018	Y	Y	Y	Y	Y	U	Y	N	U	U	25% (3rd)	No

Nowak and Schemitsch 2019	Y	Y	Y	U	U	Y	Y	N	U	U	30% (3rd)	Yes
Peskun 2012	Y	Y	Y	U	U	Y	Y	N	N	U	35% (3rd)	No
Buitagro 2020	Y	Y	Y	U	U	Y	Y	N	U	U	30% (3rd)	No
Schairer 2014	U	U	U	N	N	Y	Y	N	N	N	65% (4th)	No
Bullock 2003	Y	Y	Y	N	N	Y	Y	N	N	N	50% (4th)	No
Charette 2019	Y	Y	Y	U	U	U	Y	N	N	U	50% (4th)	Yes
Hanly 2017	Y	U	U	N	N	N	Y	N	N	N	70% (4th)	No
Jauregui 2015	Y	U	U	N	N	U	Y	N	N	N	65% (4th)	Yes
Keeney 2015	Y	U	U	N	N	U	Y	N	U	U	55% (4th)	No
Kheir 2014	Y	Y	Y	U	N	Y	Y	N	N		45% (4th)	Yes
Kuo 2017	Y	U	U	Y	Y	U	Y	N	N	U	40% (4th)	No
Miric 2014	Y	Y	Y	N	N	U	Y	N	N	N	55% (4th)	No
Mudumbai 2019	Y	Y	Y	N	N	Y	Y	N	U	N	45% (4th)	No
Robinson 2017	Y	Y	Y	N	N	Y	Y	N	U	N	45% (4th)	No

Schaeffer 2015	Y	Y	Y	N	N	U	Y	N	N	N	55% (4th)	Yes
Tay 2017	Y	Y	Y	N	N	Y	Y	N	N	N	50% (4th)	Yes
Antoniak 2020	Y	Y	Y	N	N	Y	Y	N	U	N	45% (4th)	Yes

*Joanna Briggs Institute Critical Appraisal Checklist for Cohort Studies – components:

JBI 1. Were the groups similar and recruited from the same population?

JBI 2. Were the exposures measured similarly to assign people to both exposed and unexposed groups?

JBI 3. Was the exposure measured in a valid and reliable way?

JBI 4. Were confounding factors identified?

JBI 5. Were strategies to deal with confounding factors stated?

JBI 6. (omitted because it is not relevant) Were the groups/participants free of the outcome at the start of the study (or at the moment of exposure)?

JBI 7. Were strategies to deal with confounding factors stated?

JBI 8. Was the follow up time reported and sufficient to be long enough for outcomes to occur?

JBI 9. Was follow up complete, and if not, were the reasons for loss to follow up described and explored?

JBI 10. Were strategies to address incomplete follow up utilized?

JBI 11. Was appropriate statistical analysis used?

Y = yes; U = unclear; N = no

S16 - Summary of Findings – Comorbidities

Meta-analysis											
Prognostic factor	**Number of participants; number of studies; number of cohorts**	**OR (95% CI)**	**Phase**	**Study limitations**	**Inconsistency (I^2) – 50% threshold for serious limitation**	**Indirectness**	**Imprecision**	**Publication bias**	**Moderate /large effect size**	**Dose effect**	**Overall quality**
**Alcohol abuse**	947,326; 3; 3	1.08 (0.96-1.20)	2	✓	0%	✓	✓	✓	X	X	6 (++++)
**BMI <18.5 vs normal**	105,401; 2; 2	1.15 (0.45-2.98)	2	✓	0%	✓	X	X	X	X	4 (+++)
**BMI 25-30 vs normal**	121,886; 3; 3	0.91 (0.80-1.03)	2	✓	0%	✓	✓	✓	X	X	6 (++++)
**BMI 30-35 vs normal**	121,886; 3; 3	0.90 (0.80-1.02)	2	✓	0%	✓	✓	✓	X	X	6 (++++)
**BMI 35-40 vs normal**	121,886; 3; 3	0.84 (0.69-1.02)	2	✓	0%	✓	✓	✓	X	X	6 (++++)
**BMI >40 vs normal**	121,886; 3; 3	1.05 (0.84-1.31)	2	✓	0%	✓	✓	✓	X	X	6 (++++)
**Obesity**	1,168,493; 3; 3	1.06 (1.02-1.09)	2	✓	34%	X	✓	X	X	X	4 (+++)
**Weight loss**	944,028; 2; 2	0.95 (0.71-1.27)	2	✓	35%	X	X	X	X	X	3 (++)
**Arrhythmias and AF combined**	573,805; 2; 2	1.14 (1.09-1.19)	2	✓	0%	X	✓	X	X	X	4 (+++)
**IHD + CAD + cardiac disease**	17,920; 2; 2	1.29 (0.79-2.09)	2	X	30%	X	✓	X	X	X	3 (++)
**Peripheral vascular disease**	944,028; 2; 2	1.17 (1.10-1.24)	2	✓	0%	✓	✓	X	X	X	5 (+++)

**Previous myocardial infarction, or coronary artery disease**	566,479; 2; 2	1.18 (0.29-4.88)	2	✓	8%	X	X	X	X	X	3 (++)
**NIDDM**	31,988; 2; 2	1.08 (0.80-1.45)	2	✓	0%	✓	X	X	X	X	4 (+++)
**Liver disease**	1,377,666; 3; 3	1.29 (1.20-1.39)	2	X	0%	✓	✓	X	X	X	4 (+++)
**Peptic ulcer disease**	944,028; 2; 2	0.94 (0.84-1.07)	2	✓	0%	✓	X	X	X	X	4 (+++)
**Anaemia**	437,565; 2; 2	1.19 (1.15-1.24)	2	✓	0%	✓	✓	✓	X	X	6 (++++)
**Deficiency anaemias**	944,028; 2; 2	1.06 (1.01-1.11)	2	✓	0%	X	✓	X	X	X	4 (+++)
**Coagulopath y**	1,377,666; 3; 3	1.25 (1.15-1.36)	2	X	16%	✓	✓	X	X	X	4 (+++)
**Fluid and electrolyte disorder**	944,028; 2; 2	1.05 (1.00-1.12)	2	✓	0%	X	✓	X	X	X	4 (+++)
**Chronic pulmonary disease**	1,172,420; 4; 4	1.28 (1.22-1.34)	2	✓	54%	X	✓	X	X	X	3 (++)
**Paralysis**	944,028; 2; 2	1.13 (0.97-1.31)	2	✓	0%	X	✓	X	X	X	4 (+++)
**Psychiatric disorder**	947,326; 3; 3	1.43 (1.12-1.70)	2	✓	52%	X	✓	X	X	X	3 (++)
**Smoking**	390,193; 3; 3	1.25 (0.82-1.91)	2	✓	2%	X	✓	✓	X	X	5 (+++)
**Rheumatolo gic disorder**	1,602,131; 4; 4	1.11 (1.04-1.18)	2	✓	58%	X	✓	X	X	X	3 (++)

Narrative Synthesis																
Prognostic factor	Number of participants (study ID); number of studies; number of cohorts	Univariate			Multivariate			Phase	Study limitations	Inconsistency	Indirectness	Imprecision	Publication bias	Moderate /large effect size	Dose effect	Overall quality
		+	0	-	+	0	-									
Composite comorbidity indices																
**CCI 1-2 (reference category = 0)**	965,046 (Kurtz, Buitagro); 2; 2	-	-	-	2	-	-	2	✓	✓	✓	✓	X	X	✓	6 (++++)
**CCI 1 (reference category = 0)**	822,828 (Ross, Welsh, Mudumbai); 3; 3	-	-	-	2	1	-	2	✓	✓	✓	✓	X	X	✓	6 (++++)
**CCI 2 (reference category = 0)**	210,145 (Ross); 1; 1	-	-	-	1	-	-	2	✓	N/A	✓	✓	X	X	✓	5 (++++)
**CCI ≥2 (reference category = 0)**	612,683 (Welsh, Mudumbai); 1; 1	-	-	-	1	1	-	2	✓	✓	✓	✓	X	X	✓	6 (++++)
**CCI 3-4 (reference category = 0)**	952,593 (Kurtz); 1; 1	-	-	-	1	-	-	2	✓	N/A	✓	✓	X	X	✓	5 (+++)
**CCI ≥3 (reference category = 0)**	12,453 (Buitagro); 1; 1	-	-	-	1	-	-	2	X	N/A	✓	✓	X	X	✓	4 (+++)
**CCI 5+ (reference category = 0)**	952,593 (Kurtz); 1; 1	-	-	-	1	-	-	2	✓	N/A	✓	✓	X	X	✓	5 (+++)
**Increasing CCI**	418 (Tay); 1; 1	-	-	-	-	1	-	2	X	N/A	✓	X	X	X	✓	3 (++)
**Presence of any comorbidity**	363,342 (Lehtonen, Ramos, Urish); 3; 3	1	-	-	2	-	-	2	✓	✓	X	✓	X	X	X	4 (+++)

**Increasing ASA classification (reference category = 2)**	137,209 (Lehtonen); 1; 1	-	-	-	1	-	-	2	✓	N/A	✓	✓	X	X	X	4 (+++)
**Increasing ASA classification (reference category = 1)**	2621 (Tang); 1; 1	-	-	-	-	1	-	2	X	N/A	✓	X	X	X	X	2 (+)
**ASA classification (other)**	731 (Ricciardi, Shcaeffer); 2; 2	-	1	-	-	1	-	1, 2	X	X	✓	X	X	X	X	2 (+)
**Increasing Elixhauser Index**	739,857 (Arroyo); 1; 1	-	-	-	1	-	-	2	✓	N/A	✓	✓	X	X	✓	5 (+++)
**Increasing DRG (Diagnosis-related group)**	5514 (Mudumbai); 1; 1	-	-	-	1	-	-	2	X	N/A	✓	X	X	X	✓	3 (++)
Cardiovascular																
**Hypertension**	2,547,837 (Ali, Abola, Bovonratwet 2020, Nowak, Siracuse, D'Apuzzo, Ricciardi, Lehtonen, Liao, Arroyo, Workman); 11; 11	2	2	-	6	1	-	1, 2	✓	✓	✓	✓	X	X	X	5 (+++)
**Hyperlipidaemia**	19,296 (Workman, Pugely); 2; 2	1	1	-	-	-	-	2	✓	X	✓	✓	X	X	X	4 (+++)
**Cardiac disease**	952,593 (Kurtz); 1; 1	-	-	-	1	-	-	2	✓	N/A	X	✓	X	X	X	3 (++)
**Cardiovascular disease (CVD)**	3431 (Liao); 1; 1	-	1	-	-	-	-	1	✓	N/A	✓	X	X	X	X	3 (++)

**Congestive Cardiac/Heart Failure (CCF/CHF)**	3,003,728 (Urish, Ali, Nowak, Yohe, Siracuse, Tang, D'Apuzzo, Lehtonen, Arroyo, Siracuse); 10; 10	3	-	-	5	2	-	1, 2	X	✓	✓	✓	✓	X	X	5 (+++)
**Valvular disease**	1,683,954 (Ali, D'Apuzzo, Ricciardi, Arroyo); 4; 4	1	-	-	2	1	-	1, 2	X	✓	X	✓	X	X	X	3 (++)
**Peripheral vascular disease**	751,740 (Pugely, Arroyo, Ricciardi); 3; 3	1	1	-	-	1	-	1, 2	X	✓	✓	X	X	X	X	3 (++)
**History of percutaneous coronary intervention or cardiac surgery**	12,010 (Jauregui); 1; 1	1	-	-	-	-	-	1	X	N/A	✓	✓	X	X	X	2 (+)
BMI, obesity, and weight loss																
**BMI (continuous)**	156,773 (George, Tang, Kheir); 3; 3	-	1	-	1	1	-	1, 2	✓	X	✓	✓	X	X	X	4 (+++)
**BMI underweight (reference category = overweight (25-30))**	137,209 (Lehtonen); 1; 1	-	-	-	-	1	-	2	✓	N/A	✓	✓	X	X	X	4 (+++)
**BMI normal weight (reference category =**	137,209 (Lehtonen); 1; 1	-	-	-	-	1	-	2	✓	N/A	✓	✓	X	X	X	4 (+++)

**overweight (25-30))**																
**BMI obese (reference category = overweight (25-30))**	137,209 (Lehtonen); 1; 1	-	-	-	-	1	-	2	✓	N/A	✓	✓	X	X	X	4 (+++)
**BMI very obese (reference category = overweight (25-30))**	137,209 (Lehtonen); 1; 1	-	-	-	-	1	-	2	✓	N/A	✓	✓	X	X	X	4 (+++)
**BMI morbidly obese (reference category = overweight (25-30))**	137,209 (Lehtonen); 1; 1	-	-	-	1	-	-	2	✓	N/A	✓	✓	X	X	X	4 (+++)
**BMI >30 (reference category = normal weight)**	3890 (Saucedo); 1; 1	-	-	-	1	-	-	2	X	N/A	✓	X	X	X	X	2 (+)
**Increasing BMI (reference category = <25)**	3218 (Kheir); 1; 1	-	1	-	-	-	-	1	X	N/A	✓	X	X	X	X	1 (+)
**Obesity**	2,133,639 (Kurtz, Workman, Ricciardi, Arroyo, Siracuse); 4; 4	1	1	-	2	1	-	1, 2	X	X	X	✓	X	X	X	2 (+)
**Morbid obesity**	211 (Hanly); 1; 1	-	1	-	-	-	-	1	X	✓	✓	X	X	X	X	2 (+)
**Weight loss**	225,312 (Abola, Lehtonen); 2; 2	-	1	-	-	1	-	1, 2	✓	✓	X	✓	X	X	X	4 (+++)
Endocrine																
**Diabetes (general and Peskun (T2DM))**	2,463,096 (Kurtz, Ali, Abola, Sutton,	-	1	-	5	4	-	1, 2	X	X	x	✓	✓	X	X	3 (++)

	Liao, Peskun, Siracuse, D'Apuzzo, Tang, Workman); 10; 10															
**Diabetes (with complications)**	964,322 (Urish, Arroyo); 2; 2	1	-	-	1	-	-	1, 2	✓	✓	✓	✓	X	X	X	5 (+++)
**Diabetes (without complications)**	964,322 (Urish, Arroyo); 2; 2	1	-	-	1	-	-	1, 2	✓	✓	✓	✓	X	X	X	5 (+++)
**Diabetes (IDDM)**	236,717 (Webb, Lehtonen); 2; 2	1	-	-	1	-	-	1, 2	✓	✓	✓	✓	X	X	X	5 (+++)
**Diabetes (NIDDM)**	236,717 (Webb, Lehtonen); 2; 2	1	-	-	-	1	-	1, 2	✓	X	✓	✓	X	X	X	4 (+++)
**Hypothyroidism**	573,805 (Ali, Workman); 2; 2	-	1	-	-	1	-	1, 2	✓	✓	✓	✓	X	X	X	5 (+++)
Gastrointestinal																
**Liver disease**	137,278 (Lehtonen, Ricciardi); 2; 2	1	-	-	-	1	-	1, 2	✓	X	X	X	X	X	X	2 (+)
Haematological																
**Anaemia**	955,214 (Kurtz, Tang); 2; 2	-	-	-	1	1	1	2	✓	x	✓	✓	X	X	X	4 (+++)
**Anaemia (blood loss)**	1,306,180 (Ali, Arroyo); 2; 2	1	-	-	-	1	-	1, 2	✓	X	✓	✓	X	X	X	4 (+++)

**Anaemia (deficiency)**	1,306,249 (Ali, Ricciardi, Arroyo); 3; 3	1	-	-	-	2	-	1, 2	✓	X	X	✓	X	X	X	3 (++)
**Bleeding disorders**	213,455 (Lehtonen, Nowak); 2; 2	1	-	-	1	-	-	1, 2	x	✓	X	✓	X	X	X	3 (++)
**Coagulopathy**	739,857 (Arroyo); 1; 1	1	-	-	-	-	-	1	✓	N/A	✓	✓	X	X	X	3 (++)
**Anticoagulant therapy**	3927 (Jorgensen 2017); 1; 1	-	-	-	-	1	-	2	✓	N/A	X	X	X	X	X	2 (+)
**Increasing INR**	158,448 (Rudasil, Lehtonen); 2; 2	1	-	-	1	-	-	1, 2	✓	✓	X	✓	X	X	X	4 (+++)
**Fluid and electrolyte disorder**	1,173,495 (Arroyo, Siracuse); 2; 2	1	-	-	1	-	-	1, 2	X	✓	X	✓	X	X	X	3 (++)
**Elevated serum BUN**	20,375 (Hart, Pugely); 2; 2	1	-	-	1	-	-	1, 2	✓	✓	✓	X	X	X	X	4 (+++)
**Hyponatraemia**	99,917 (Abola, Pugely); 2; 2	-	1	-	-	1	-	1, 2	✓	X	✓	✓	X	X	X	4 (+++)
**Low albumin**	101,474 (Sloan); 1; 1	-	-	-	1	-	-	2	✓	N/A	✓	✓	X	X	X	4 (+++)
**Elevated creatinine**	137,209 (Lehtonen); 1; 1	1	-	-	-	-	-	1	✓	N/A	✓	✓	X	X	X	3 (++)
**Elevated WBC count**	137,209 (Lehtonen); 1; 1	1	-	-	-	-	-	1	✓	N/A	✓	✓	X	X	X	3 (++)

**Reduced haematocrit**	137,209 (Lehtonen); 1; 1	1	-	-	-	-	-	1	✓	N/A	✓	✓	X	X	X	3 (++)
**Low platelets**	137,209 (Lehtonen); 1; 1	1	-	-	-	-	-	1	✓	N/A	✓	✓	X	X	X	3 (++)
Respiratory																
**COPD and chronic airways disease (combined)**	562,325 (Bovonratwet 2020, Liao, Workman, Siracuse); 4; 4	-	-	-	4	-	-	1	✓	✓	X	✓	X	X	X	3 (++)
**Pulmonary disease**	1,692,519 (Kurtz, Ricciardi, Arroyo); 3; 3	1	-	-	1	1	-	1, 2	✓	✓	X	✓	X	X	X	4 (+++)
**Smoking**	238,308 (Abola, Mudumbai, Workman, Lehtonen); 4; 4	1	2	-	1	-	-	1, 2	✓	X	X	✓	X	X	X	3 (++)
**Pulmonary circulation disorder**	944,028 (Ali, D'Apuzzo); 2; 2	-	-	-	1	1	-	2	X	X	X	✓	X	X	X	2 (+)
**Asthma**	7482 (Workman); 1; 1	-	1	-	-	-	-	1	X	N/A	✓	X	X	X	X	1 (+)
**Dyspnoea**	225,312 (Abola, Lehtonen); 2; 2	1	-	-	-	1	-	1, 2	✓	X	X	✓	X	X	X	3 (++)
**Previous pneumonia**	566,323 (Ali); 1; 1	-	-	-	-	1	-	2	✓	N/A	X	✓	X	X	X	3 (++)
**Obstructive sleep apnoea**	7482 (Workman); 1; 1	-	1	-	-	-	-	1	X	N/A	✓	X	X	X	X	1 (+)

**Cardiopulmonary disease**	1481 (Jorgensen 2013); 1; 1	1	-	-	-	-	-	1	X	N/A	X	X	X	X	X	0 (+)
Psychiatric																
**Depression**	2,649,543 (Ali, Kurtz, Ricciardi, D’Apuzzo, Arroyo, Workman, Mudumbai); 7; 7	1	2	-	3	1	-	1, 2	✓	✓	✓	✓	X	X	X	5 (+++)
**‘Other’ mental health condition (other than depression)**	566,323 (Ali); 1; 1	-	-	-	1	-	-	2	✓	N/A	X	✓	X	X	X	3 (++)
**Bipolar disorder**	5514 (Mudumbai); 1; 1	-	1	-	-	-	-	1	X	N/A	✓	X	X	X	X	1 (+)
**PTSD**	5514 (Mudumbai); 1; 1	-	1	-	-	-	-	1	X	N/A	✓	X	X	X	X	1 (+)
**Anxiety disorder**	12,996 (Mudumbai, Workman); 2; 2	-	2	-	-	-	-	1	X	✓	X	✓	X	X	X	2 (+)
**Alcohol abuse**	753,152 (Arroyo, Jorgensen 2013, Pugely); 3; 3	1	2	-	-	-	-	1	✓	✓	X	✓	X	X	X	3 (++)
**Drug abuse (including general substance abuse designation, and drug/alcohol abuse (combined category in Kurtz))**	2,264,287 (Ali, Kurtz, Arroyo, Mudumbai); 4; 4	1	1	-	2	-	-	1, 2	✓	✓	X	✓	X	X	X	4 (+++)
**Psychoses**	739,857 (Arroyo); 1; 1	1	-	-	-	-	-	1	✓	N/A	X	✓	X	X	X	2 (+)

Neoplastic																
**History of cancer**	1,695,699 (Ali, Pugely, D'Apuzzo, Arroyo); 4; 4	1	-	-	2	1	-	1, 2	✓	✓	X	✓	X	X	X	4 (+++)
**Disseminated cancer**	1,618,833 (Ali, Abola, Kurtz, Pugely); 4; 4	1	-	-	1	2	-	1, 2	✓	X	X	✓	X	X	X	3 (++)
**Lymphoma**	1,330,298 (Kurtz, D'Apuzzo); 2; 2	-	-	-	2	-	-	2	X	✓	✓	✓	X	X	X	4 (+++)
Neurological																
**Previous stroke**	572,375 (Ali, Tang, Liao); 3; 3	-	1	-	1	-	1	1, 2	✓	X	✓	✓	X	X	X	4 (+++)
**Dementia**	566,323 (Ali); 1; 1	-	-	-	1	-	-	2	✓	N/A	✓	✓	X	X	X	4 (+++)
**Other neurological disorder**	1,683,954 (Ali, D'Apuzzo, Ricciardi, Arroyo); 4; 4	1	-	-	2	1	-	1, 2	X	✓	X	✓	X	X	X	3 (++)
In-hospital complications																
**Deep vein thrombosis**	137,209 (Lehtonen); 1; 1	-	-	-	1	-	-	2	✓	N/A	✓	✓	X	✓	X	5 (+++)
**Pulmonary embolism**	137,209 (Lehtonen); 1; 1	-	-	-	1	-	-	2	✓	N/A	✓	✓	X	✓	X	5 (+++)
**Any complication**	227,469 (Runner, Lehtonen); 2; 2	1	-	-	1	-	-	1, 2	✓	✓	✓	✓	X	✓	X	6 (++++)
**Any medical complication**	514,914 (D'Apuzzo,	1	-	-	1	-	-	1, 2	X	✓	✓	✓	X	X	X	4 (+++)

	Lehtonen); 2; 2															
**Any surgical complication**	514,914 (D'Apuzzo, Lehtonen); 2; 2	1	-	-	1	-	-	1, 2	X	✓	✓	✓	X	X	X	4 (+++)
**Urinary tract infection**	137,209 (Lehtonen); 1; 1	-	-	-	1	-	-	2	✓	N/A	✓	✓	X	✓	X	5 (+++)
**Surgical site infection**	137,209 (Lehtonen); 1; 1	-	-	-	1	-	-	2	✓	N/A	✓	✓	X	✓	X	5 (+++)
**Sepsis**	225,312 (Abola, Lehtonen); 2; 2	1	-	-	-	1	-	1, 2	✓	✓	✓	✓	X	X	X	4 (+++)
**Cardiac (including cardiac arrest and myocardial infarction)**	137,209 (Lehtonen); 1; 1	-	-	-	1	-	-	2	✓	N/A	x	✓	X	✓	X	4 (+++)
**Pneumonia**	137,209 (Lehtonen); 1; 1	-	-	-	1	-	-	2	✓	N/A	✓	✓	X	✓	X	5 (+++)
**Acute renal failure**	137,209 (Lehtonen); 1; 1	-	-	-	1	-	-	2	✓	N/A	✓	✓	X	✓	X	5 (+++)
**CVA or TIA**	137,209 (Lehtonen); 1; 1	1	-	-	-	-	-	1	✓	N/A	✓	✓	X	X	X	3 (++)
Renal																
**CKD**	116,108 (Liao, Workman, Kuo, Antoniak); 4; 4	1	-	-	2	1	-	1, 2	X	✓	✓	✓	✓	X	X	5 (+++)
**Dialysis dependence**	163,810 (Ottesen); 1; 1	-	-	-	1	-	-	2	✓	N/A	✓	✓	X	X	X	4 (++)
**Renal failure – acute, preoperative**	88,103 (Abola); 1; 1	-	-	-	-	1	-	2	✓	N/A	✓	✓	X	X	X	4 (++)

**Renal failure/disease – chronicity unspecified**	3,294,581 (Urish, Ali, Kurtz, Siracuse, D'Apuzzo, Arroyo); 6; 6	1	-	-	5	-	-	1, 2	✓	✓	✓	✓	✓	X	X	6 (++++)
Rheumatological and autoimmune																
**Rheumatoid arthritis/collagen vascular diseases**	739,857 (Arroyo); 1; 1	1	-	-	-	-	-	1	✓	N/A	✓	✓	X	X	X	3 (++)
**Steroid or other immunosuppressant use for chronic condition**	111,624 (Curtis 2018); 1; 1	-	-	-	1	-	-	2	✓	N/A	X	✓	X	X	X	3 (++)
Other																
**Preoperative opioid use**	554,801 (Kim, Weick, Mudumbai); 3; 3	-	-	-	2	1	-	2	X	✓	X	✓	X	X	X	3 (++)
**Post-discharge opioid use**	5514 (Mudumbai); 1; 1	-	-	-	1	-	-	2	X	N/A	X	X	X	X	X	1 (+)
**Preoperative medication use (general)**	12,639 (Anderson); 1; 1	-	-	-	1	-	-	2	✓	N/A	X	✓	X	X	X	3 (++)
**Preoperative medication use (analgesics)**	5514 (Mudumbai); 1; 1	-	-	-		1	-	2	X	N/A	X	X	X	X	X	1 (+)
**Preoperative medication use (anticonvulsants)**	5514 (Mudumbai); 1; 1	-	-	-	1	-	-	2	X	N/A	✓	X	X	X	X	2 (+)
**Preoperative medication use (SNRIs)**	5514 (Mudumbai); 1; 1	-	-	-	-	1	-	2	X	N/A	✓	X	X	X	X	2 (+)
**Preoperative medication use (TCAs)**	5514 (Mudumbai); 1; 1	-	-	-	-	1	-	2	X	N/A	✓	X	X	X	X	2 (+)
**Preoperative medication use (sedatives)**	5514 (Mudumbai); 1; 1	-	-	-	-	1	-	2	X	N/A	✓	X	X	X	X	2 (+)

**Wound class**	315,572 (Abola, Runner, Lehtonen); 3; 3	1	-	-	-	2	-	1, 2	✓	✓	X	✓	X	X	X	4 (+++)

✓ = No serious limitation in this criterion among the studies which analysed the given risk factor; X = serious limitation in this criterion among the studies which analysed the given risk factor; N/A given a score of zero, same as X = No serious limitation in this criterion among the studies which analysed the given risk factor; X = serious limitation in this criterion among the studies which analysed the given risk factor; N/A given a score of zero, same as X; + very low quality = very little confidence in the effect estimate: true effect likely to be substantially different from the estimate of effect; ++ low quality = confidence in the effect estimate is limited: the true effect may be substantially different from the estimate of the study; +++ moderate quality = moderately confident in the effect estimate: true effect is likely to be close to the estimate of the effect, but there is a possibility that it is substantially different; ++++ high quality = very confident that the true effect lies close to that of the estimate of the effect

S17 - Summary of Findings – Demographics

Meta-analysis											
Prognostic factor	**Number of participants; number of studies; number of cohorts**	**OR (95% CI)**	**Phase**	**Study limitations**	**Inconsistency (I^2) – 50% threshold**	**Indirectness**	**Imprecision**	**Publication bias**	**Moderate /large effect size**	**Dose effect**	**Overall quality**
**Hispanic race**	1,736,757; 4; 4	0.92 (0.68-1.25)	2	✓	58%	✓	✓	✓	X	X	5 (+++)

Narrative Synthesis															
Prognostic factor	**Number of participants (study ID); number of studies; number of cohorts**	**Univariate**			**Multivariate**			**Phase**	**Study limitations**	**Inconsistency**	**Indirectness**	**Imprecision**	**Publication bias**	**Moderate /large effect size**	**Dose effect**	**Overall quality**
		+	0	-	+	0	-									
Age (continuous variable)																
**Age**	1,933,990 (Lehtonen, Ross, Arroyo, Welsh, Ramos, Peskun, Tang, Urish, Workman, Kheir); 10; 10	3	1	-	4	3	-	1, 2	✓	X	✓	✓	✓	X	X	5 (+++)
Sex																
**Female sex**	1,380,453 (Patel, Arroyo, Ross, Liao, Tang, Mudumbai); 6; 6	-	1	-	-	1	4	1, 2	✓	X	✓	✓	✓	X	X	5 (+++)
**Male sex**	3,418,447 (Kurtz, Ali, Runner, Urish, Singh, Workman, D’Apuzzo, Siracuse, Arroyo, Liao,	2	2	-	8	-	-	1, 2	✓	✓	✓	✓	✓	X	X	6 (++++)

	Jorgensen 2013, Kheir); 12; 12															
Race																
**Black (reference = white or non-Black)**	2,878,115 (Ali, Lehtonen, Arroyo, Welsh, Workman, Siracuse, D'Apuzzo, Mudumbai, Kheir); 9; 9	1	1	-	4	3	-	1, 2	✓	X	✓	✓	X	X	X	4 (+++)
**Hispanic (reference = white or non-Hispanic)**	5514 (Mudumbai); 1; 1	-	1	-	-	-	-	1	X	N/A	✓	X	X	X	X	1 (+)
**Asian (reference = white)**	1,089,969 (Lehtonen, Ali, D'Apuzzo, Mudumbai, Kheir); 5; 5	-	2	-	-	1	2	1, 2	✓	X	✓	✓	X	X	X	4 (+++)
**Native Hawaiian (reference = white)**	137,209 (Lehtonen); 1; 1	-	1	-	-	-	-	1	✓	N/A	✓	✓	X	X	X	3 (++)
**American Indian (reference = white)**	145,941 (Lehtonen, Mudumbai, Kheir))	-	2	-	-	1	-	1, 2	✓	X	✓	✓	X	X	X	4 (+++)
**White**	16,214 (Mudumbai, Kheir, Workman); 3; 3	-	1	1	-	1	-	1, 2	X	X	✓	X	X	X	X	2 (+)
**Indian (reference = Chinese)**	2621 (Tang); 1; 1	-	-	-	-	1	-	2	X	N/A	✓	X	X	X	X	2 (+)
**Malay (reference = Chinese)**	2621 (Tang); 1; 1	-	-	-	-	1	-	2	X	N/A	✓	X	X	X	X	2 (+)
**Biracial (Workman) or**	573,805 (Ali, Workman); 2; 2	1	-	-	1	-	-	1, 2	✓	✓	✓	✓	X	X	X	5 (+++)

**mixed race (Ali)**																
**Minority ethnicity**	377,705 (D'Apuzzo); 1; 1	-	-	-	1	-	-	2	X	N/A	X	✓	X	X	X	2 (+)
**Other (Tang = Chinese; otherwise = white)**	2,818,634 (Ali, Abola, Arroyo, Welsh, Siracuse, D'Apuzzo, Tang, Kheir); 8; 8	-	1	-	1	4	2	1, 2	✓	X	X	✓	X	X	X	3 (++)
**Missing**	1,824,312 (Ali, Lehtonen, Arroyo, D'Apuzzo, Kheir); 5; 5	-	1	-	1	-	3	1, 2	✓	X	X	✓	X	X	X	3 (++)
**Race (combined analysis – i.e. racial difference exists between readmitted and non-readmitted cohorts)**	1,829,659 (Kurtz, Lehtonen, Arroyo); 3; 3	2	-	-	1	-	-	1, 2	✓	✓	X	✓	X	X	X	4 (+++)
Socioeconomic																
**Decreasing incoming**	1,608,105 (Urish, Ross, Arroyo, Siracuse); 4; 4	-	-	-	3	1	-	2	✓	✓	X	✓	X	X	✓	5 (+++)
**Low socioeconomic status**	569,441 (Ali, Keeney); 2; 2	1	-	-	1	-	-	1, 2	✓	✓	X	✓	X	X	X	4 (+++)
Insurance status																
**Medicare (reference category = private insurance or non-Medicare)**	1,775,665 (Urish, Arroyo, Siracuse, D'Apuzzo); 4; 4	-	-	-	4	-	-	2	✓	✓	X	✓	X	X	X	4 (+++)
**Medicaid (reference category = private insurance)**	1,551,200 (Arroyo, Siracuse, D'Apuzzo); 3; 3	-	-	-	3	-	-	2	x	✓	X	✓	X	X	X	3 (++)

**Self-pay, no charge, workers' compensation, or other (reference category = private insurance)**	1,551,200 (Arroyo, Siracuse, D'Apuzzo); 3; 3	-	-	-	-	2	3	2	x	X	X	✓	X	X	X	2 (+)
**Disability entitlement**	607,169 (Welsh); 1; 1	-	-	-	1	-	-	2	✓	N/A	X	✓	X	X	X	3 (++)
Functional status, living situation, and frailty																
**Dependent functional status**	117,774 (Bovonratwet 2020); 1; 1	-	-	-	1	-	-	2	✓	N/A	✓	✓	X	X	X	4 (+++)
**Use of walking aids**	5,408 (Jorgensen 2017, Jorgensen 2013); 2; 2	1	-	-	-	1	-	1, 2	✓	✓	✓	X	X	X	X	4 (+++)
**Living alone**	571,731 (Jorgensen 2017, Ali, Jorgensen 2013); 3; 3	-	1	-	-	2	-	1, 2	✓	✓	✓	✓	X	X	X	5 (+++)
**Living in an institution or nursing home**	5,408 (Jorgensen 2017, Jorgensen 2013); 2; 2	-	1	-	1	-	-	1, 2	✓	X	✓	X	X	X	X	3 (++)
**Homeless**	5514 (Mudumbai); 1; 1	-	-	-	1	-	-	2	x	N/A	✓	X	X	X	X	2 (+)
**Frailty (Modified Frailty Index)**	90,260 (Runner); 1; 1	-	-	-	1	-	-	2	✓	N/A	✓	✓	X	✓	X	5 (+++)

✓ = No serious limitation in this criterion among the studies which analysed the given risk factor; X = serious limitation in this criterion among the studies which analysed the given risk factor; N/A given a score of zero, same as X; + very low quality = very little confidence in the effect estimate: true effect likely to be substantially different from the estimate of effect; ++ low quality = confidence in the effect estimate is limited: the true effect may be substantially different from the estimate of the study; +++ moderate quality = moderately confident in the effect estimate: true effect is likely to be close to the estimate of the effect, but there is a possibility that it is substantially different; ++++ high quality = very confident that the true effect lies close to that of the estimate of the effect

S18 - Summary of Findings – Other

Meta-analysis											
**Prognostic factor**	**Number of 262articipant; number of studies; number of cohorts**	**OR (95% CI)**	**Phase**	**Study limitations**	**Inconsistency (I^2)**	**Indirectness**	**Imprecision**	**Publication bias**	**Moderate /large effect size**	**Dose effect**	**Overall quality**
**Elective vs non-elective**	419,323; 2; 2	0.82 (0.71-0.95)	2	✓	0%	✓	✓	X	X	X	5 (+++)

Narrative Synthesis																
**Prognostic factor**	**Number of participants (study ID); number of studies; number of cohorts**	**Univariate**			**Multivariate**			**Phase**	**Study limitations**	**Inconsistency**	**Indirectness**	**Imprecision**	**Publication bias**	**Moderate /large effect size**	**Dose effect**	**Overall quality**
		+	0	-	+	0	-									
**Operative variables**																
**Elective or non-elective procedure**	88,103 (Abola); 1; 1	-	-	-	-	1	-	2	✓	N/A	✓	✓	X	X	X	4 (+++)
**Emergency procedure**	88,103 (Abola); 1; 1	-	-	-	-	1	-	2	✓	N/A	✓	✓	X	X	X	4 (+++)
**Traumatic indication for TKA**	675,518 (Kester, Welsh); 2; 2	-	-	-	1	1	-	2	✓	X	✓	✓	X	X	X	4 (+++)
**Bilateral procedure**	996,823 (Hart, Welsh, D'Apuzzo, Tang, Bullock); 5; 5	-	1	-	2	2	-	1, 2	✓	X	X	✓	X	X	X	3 (++)
**Revision surgery (vs primary)**	217,066 (Ross, Mudumbai,	1	1	-	1	-	-	1, 2	X	✓	X	✓	X	X	X	3 (++)

	Schairer); 3; 3																
**Healthcare utilisation**																	
**Increasing number of previous admissions**	1,173,492 (Ali, Welsh); 2; 2	-	-	-	2	-	-	2	✓	✓	X	✓	X	X	✓	5 (+++)
**Number of prior knee procedures**	739,857 (Arroyo); 1; 1	-	-	-	-	-	1	2	✓	N/A	X	✓	X	X	✓	4 (+++)
**GP visit between surgery and readmission**	210,145 (Ross); 1; 1	1	-	-	-	-	1	2	✓	N/A	X	✓	X	X	X	3 (++)
**Radiation therapy within 90 days prior to procedure**	11,814 (Pugely); 1; 1	-	-	-	-	-	-									
**Chemotherapy within 30 days prior to procedure**	11,814 (Pugely); 1; 1	-	1	-	-	-	-	1	✓	N/A	X	✓	X	X	X	2 (+)
**Prior operation**	17,328 (Mudumbai (within 180 days), Pugely (within 30 days)); 1; 1	-	-	-	-	1	-	2	X	N/A	X	X	X	X	X	1 (+)
**>30 outpatient visits in the 365 days prior to procedure**	5514 (Mudumbai); 1; 1	-	-	-	1	-	-	2	X	N/A	X	X	X	X	X	1 (+)
**Patient-reported outcome measures**	584 (Sodhi and Mont et al); 1; 1	-	-	-	1	-	-	2	X	N/A	X	X	X	X	X	1 (+)
**Patient location**	17,967 (Buitagro,	-	-	-	-	1	1	2	X	X	X	✓	X	X	X	2 (+)

	Mudumbai); 2; 2															

✓ = No serious limitation in this criterion among the studies which analysed the given risk factor; X = serious limitation in this criterion among the studies which analysed the given risk factor; N/A given a score of zero, same as X; + very low quality = very little confidence in the effect estimate: true effect likely to be substantially different from the estimate of effect; ++ low quality = confidence in the effect estimate is limited: the true effect may be substantially different from the estimate of the study; +++ moderate quality = moderately confident in the effect estimate: true effect is likely to be close to the estimate of the effect, but there is a possibility that it is substantially different; ++++ high quality = very confident that the true effect lies close to that of the estimate of the effect

S19 – Forest plots

Alcohol abuse:

Source	OR (95% CI)
Jorgensen et al 2017	1.32 [0.80; 2.17]
D'Apuzzo et al 2017	1.08 [0.91; 1.28]
Ali et al 2019	1.06 [0.97; 1.16]
Total (fixed effect)	1.07 [0.99; 1.16]
Total (random effects)	1.08 [0.96; 1.20]

Heterogeneity: $\chi^2_2 = 0.73$ (P = .69), $I^2 = 0\%$

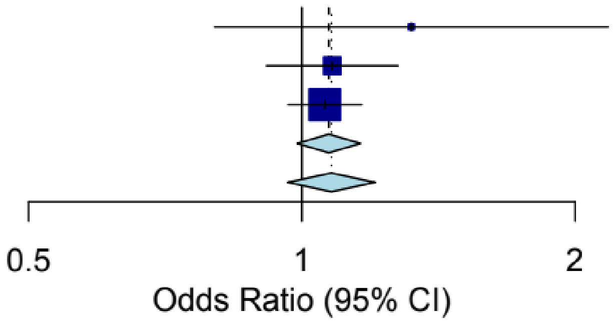

Anaemia:

Source	OR (95% CI)
Siracuse et al 2017	1.19 [1.15; 1.24]
Jorgensen et al 2017	1.21 [0.81; 1.81]
Total (fixed effect)	1.19 [1.15; 1.24]
Total (random effects)	1.19 [1.15; 1.24]

Heterogeneity: $\chi^2_1 = 0.01$ (P = .94), $I^2 = 0\%$

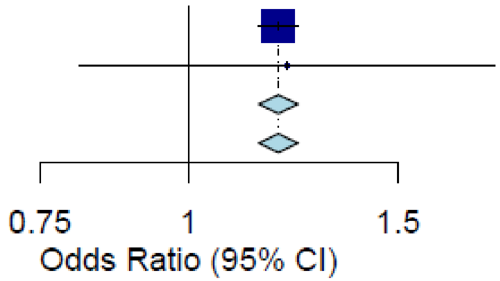

Arrhythmia and AF:

Source	OR (95% CI)
Ali et al 2019	1.14 [1.10; 1.18]
Workman et al 2019	1.03 [0.57; 1.86]
Total (fixed effect)	1.14 [1.10; 1.18]
Total (random effects)	1.14 [1.09; 1.19]

Heterogeneity: $\chi^2_1 = 0.11$ (P = .74), I^2 = 0%

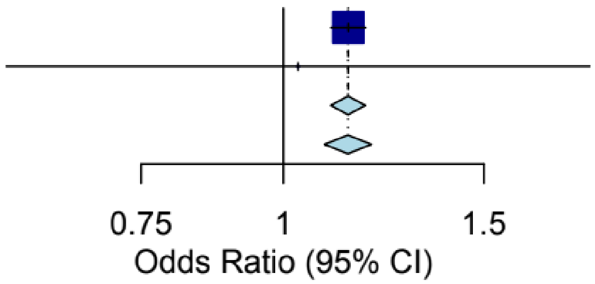

BMI 25.0 to 30 ref standard:

Source	OR (95% CI)
Jorgensen et al 2017	0.82 [0.57; 1.17]
Abdulla et al 2020	0.84 [0.62; 1.14]
Sloan et al 2020	0.94 [0.82; 1.08]
Total (fixed effect)	0.91 [0.81; 1.02]
Total (random effects)	0.91 [0.80; 1.03]

Heterogeneity: $\chi^2_2 = 0.81$ (P = .67), I^2 = 0%

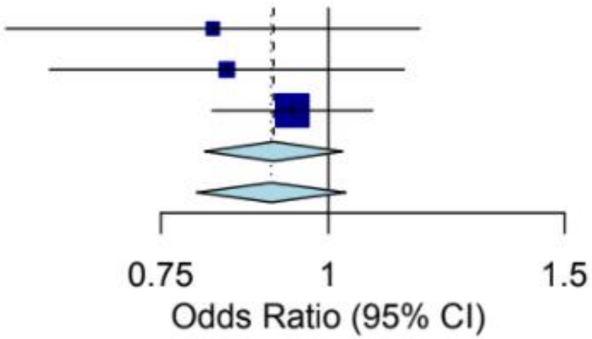

BMI 30 to 35 ref standard:

Source	OR (95% CI)
Jorgensen et al 2017	0.89 [0.60; 1.33]
Abdulla et al 2020	0.82 [0.61; 1.11]
Sloan et al 2020	0.92 [0.80; 1.05]
Total (fixed effect)	0.90 [0.80; 1.01]
Total (random effects)	0.90 [0.80; 1.02]

Heterogeneity: $\chi^2_2 = 0.47$ ($P = .79$), $I^2 = 0\%$

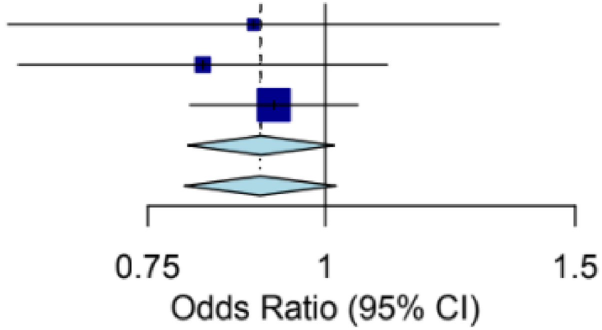

BMI 35 to 40 ref standard:

Source	OR (95% CI)
Jorgensen et al 2017	1.09 [0.65; 1.83]
Abdulla et al 2020	0.78 [0.56; 1.09]
Sloan et al 2020	0.82 [0.71; 0.95]
Total (fixed effect)	0.83 [0.73; 0.95]
Total (random effects)	0.84 [0.69; 1.02]

Heterogeneity: $\chi^2_2 = 1.22$ ($P = .54$), $I^2 = 0\%$

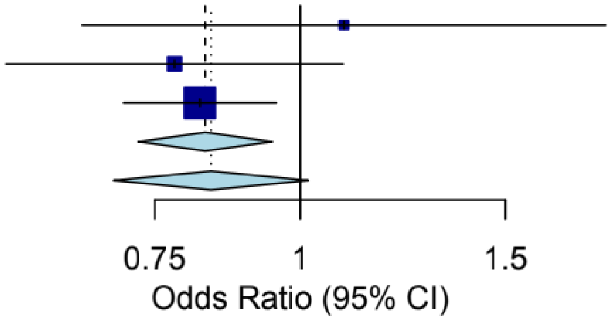

BMI over 40 ref standard:

Source	OR (95% CI)
Jorgensen et al 2017	0.70 [0.29; 1.69]
Abdulla et al 2020	1.07 [0.76; 1.50]
Sloan et al 2020	1.08 [0.93; 1.26]
Total (fixed effect)	1.07 [0.93; 1.22]
Total (random effects)	1.05 [0.84; 1.31]

Heterogeneity: $\chi^2_2 = 0.90$ (P = .64), $I^2 = 0\%$

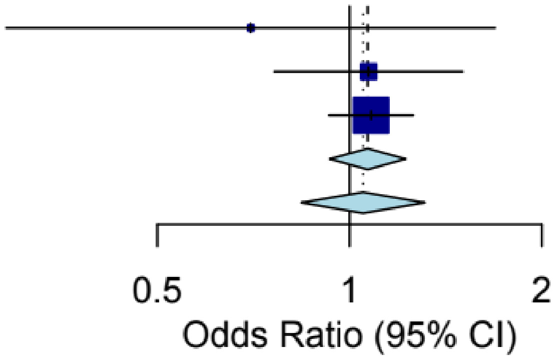

BMI under 18.5 ref standard:

Source	OR (95% CI)
Jorgensen et al 2017	0.42 [0.02; 8.72]
Sloan et al 2020	1.28 [0.62; 2.64]
Total (fixed effect)	1.21 [0.60; 2.43]
Total (random effects)	1.15 [0.45; 2.98]

Heterogeneity: $\chi^2_1 = 0.49$ (P = .48), $I^2 = 0\%$

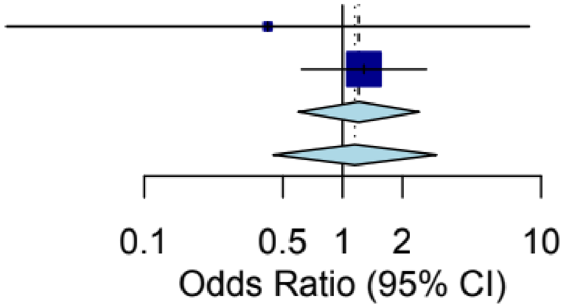

Chronic pulmonary disease:

Source	OR (95% CI)
Urish et al 2018	1.35 [1.28; 1.43]
D'Apuzzo et al 2017	1.24 [1.19; 1.29]
Ali et al 2019	1.26 [1.23; 1.30]
Jorgensen et al 2017	1.42 [0.93; 2.17]
Total (fixed effect)	1.27 [1.24; 1.29]
Total (random effects)	1.28 [1.22; 1.34]

Heterogeneity: $\chi^2_3 = 6.56$ (P = .09), I^2 = 54%

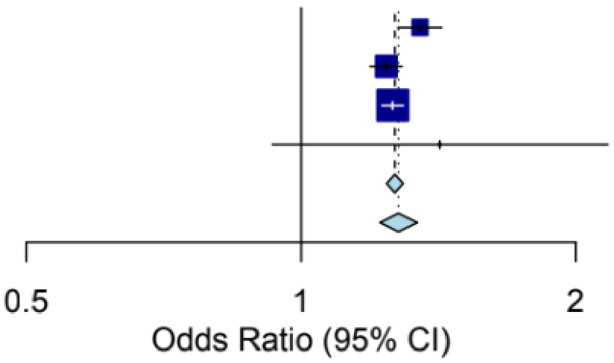

Coagulopathy:

Source	OR (95% CI)
Siracuse et al 2017	1.22 [1.11; 1.34]
D'Apuzzo et al 2017	1.19 [1.05; 1.35]
Ali et al 2019	1.36 [1.19; 1.55]
Total (fixed effect)	1.24 [1.17; 1.33]
Total (random effects)	1.25 [1.15; 1.36]

Heterogeneity: $\chi^2_2 = 2.39$ (P = .30), I^2 = 16%

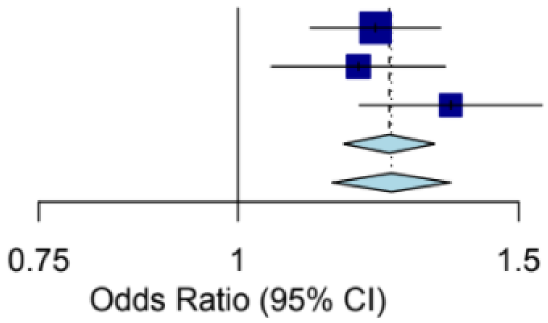

Deficiency anaemias:

Source	OR (95% CI)
D'Apuzzo et al 2017	1.06 [1.00; 1.12]
Ali et al 2019	1.06 [0.98; 1.14]
Total (fixed effect)	1.06 [1.01; 1.11]
Total (random effects)	1.06 [1.01; 1.11]

Heterogeneity: $\chi^2_1 = 0.00$ (P > .99), $I^2 = 0\%$

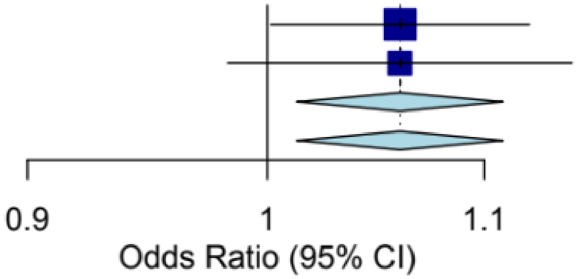

Elective procedure:

Source	OR (95% CI)
Sodhi and Anis et al 2019	0.85 [0.66; 1.09]
Ross et al 2020	0.81 [0.68; 0.96]
Total (fixed effect)	0.82 [0.71; 0.95]
Total (random effects)	0.82 [0.71; 0.95]

Heterogeneity: $\chi^2_1 = 0.07$ (P = .79), $I^2 = 0\%$

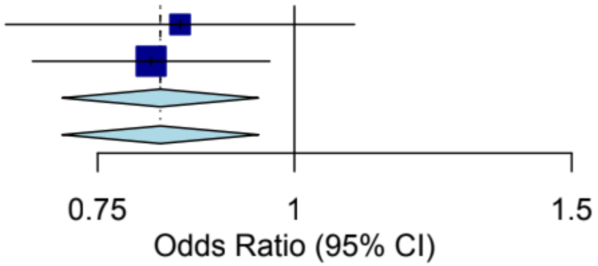

Fluid and electrolyte disorder:

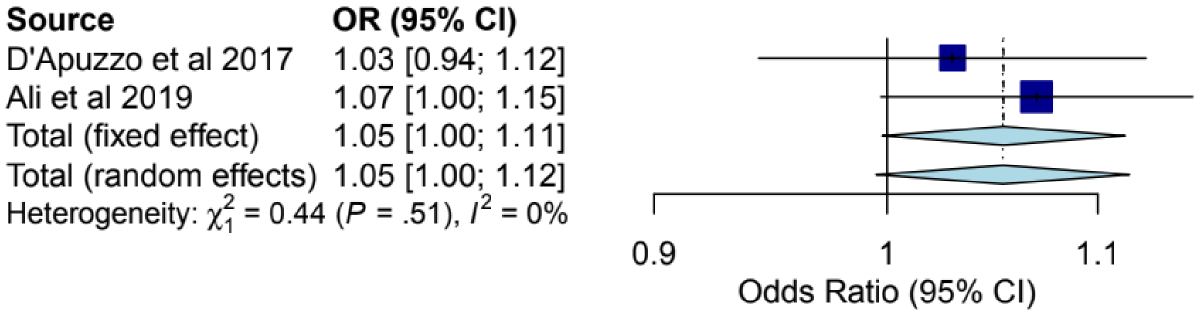

Hispanic race:

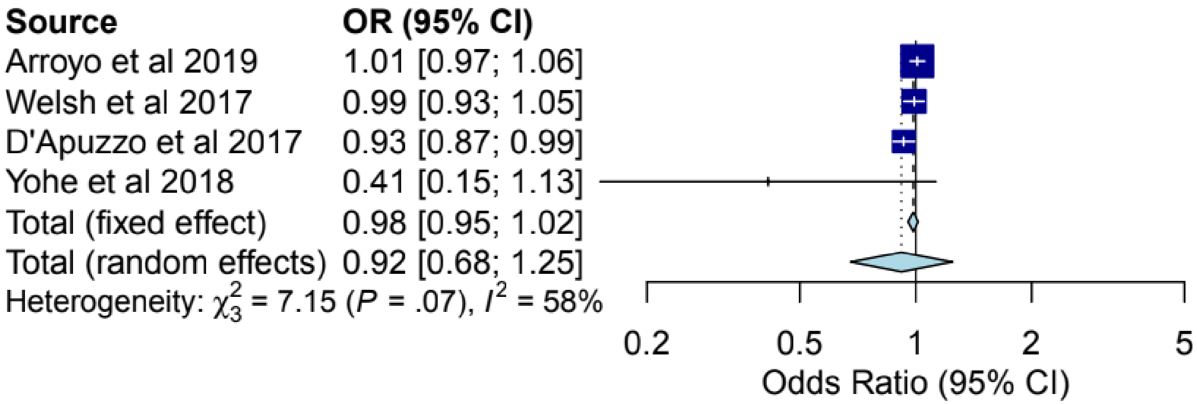

IHD and CAD and cardiac disease:

Source	OR (95% CI)
Workman et al 2019	1.06 [0.66; 1.70]
Saucedo et al 2014	1.79 [1.16; 2.77]
Tang et al 2019	0.44 [0.08; 2.42]
Jorgensen et al 2017	1.41 [0.95; 2.09]
Total (fixed effect)	1.37 [1.07; 1.76]
Total (random effects)	1.29 [0.79; 2.09]

Heterogeneity: $\chi^2_3 = 4.29$ (P = .23), I^2 = 30%

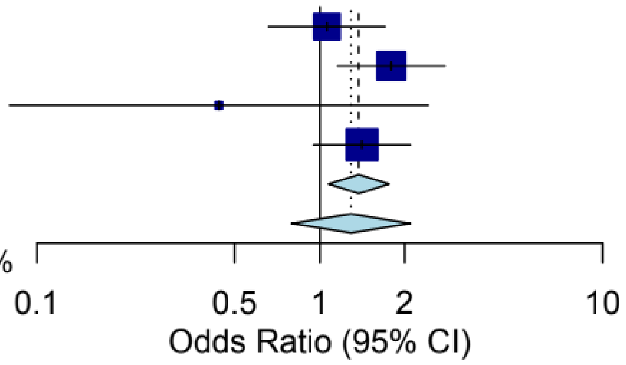

Liver disease:

Source	OR (95% CI)
Siracuse et al 2017	1.27 [1.13; 1.43]
D'Apuzzo et al 2017	1.28 [1.10; 1.48]
Ali et al 2019	1.32 [1.17; 1.49]
Total (fixed effect)	1.29 [1.20; 1.39]
Total (random effects)	1.29 [1.20; 1.39]

Heterogeneity: $\chi^2_2 = 0.22$ (P = .90), I^2 = 0%

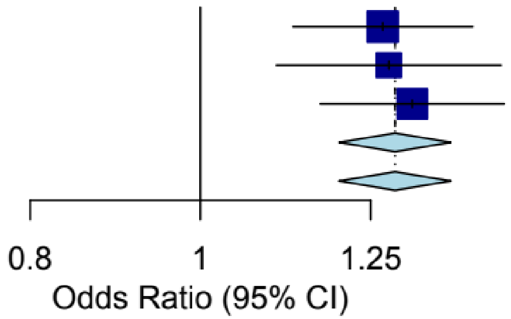

NIDDM:

Source	OR (95% CI)
Lovecchio et al 2014	0.98 [0.69; 1.39]
Jorgensen et al 2017	1.24 [0.79; 1.94]
Total (fixed effect)	1.07 [0.81; 1.41]
Total (random effects)	1.08 [0.80; 1.45]

Heterogeneity: $\chi^2_1 = 0.66$ (P = .42), I^2 = 0%

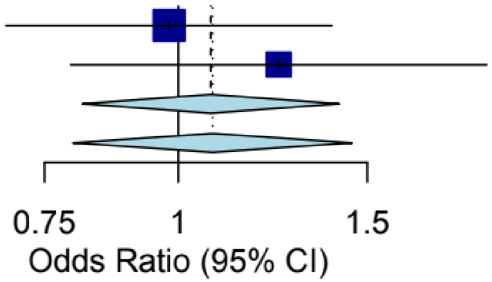

Obesity:

Source	OR (95% CI)
D'Apuzzo et al 2017	1.03 [0.99; 1.07]
Urish et al 2018	1.07 [0.94; 1.21]
Ali et al 2019	1.08 [1.04; 1.12]
Total (fixed effect)	1.06 [1.03; 1.08]
Total (random effects)	1.06 [1.02; 1.09]

Heterogeneity: $\chi^2_2 = 3.04$ (P = .22), I^2 = 34%

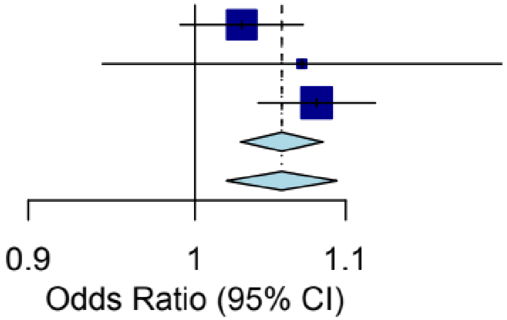

Paralysis and paraplegia:

Source	OR (95% CI)
D'Apuzzo et al 2017	1.20 [0.89; 1.62]
Ali et al 2019	1.11 [0.94; 1.32]
Total (fixed effect)	1.13 [0.98; 1.31]
Total (random effects)	1.13 [0.97; 1.31]

Heterogeneity: $\chi^2_1 = 0.19$ (P = .66), I^2 = 0%

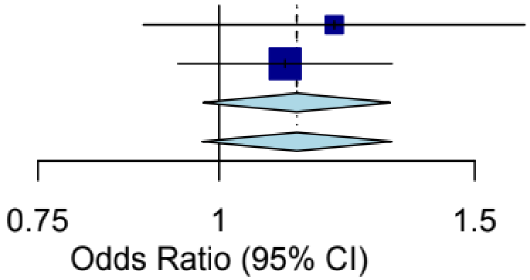

Peptic ulcer disease:

Source	OR (95% CI)
Ali et al 2019	0.98 [0.85; 1.13]
D'Apuzzo et al 2017	0.89 [0.74; 1.08]
Total (fixed effect)	0.95 [0.84; 1.06]
Total (random effects)	0.94 [0.84; 1.07]

Heterogeneity: $\chi^2_1 = 0.64$ (P = .42), I^2 = 0%

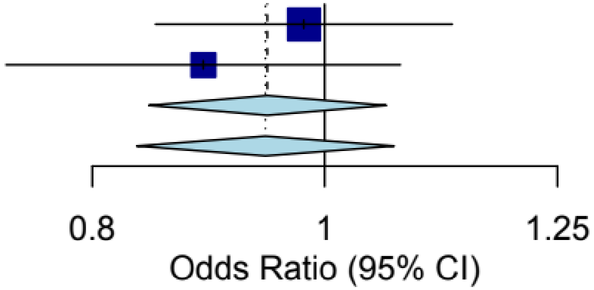

Peripheral vascular disease:

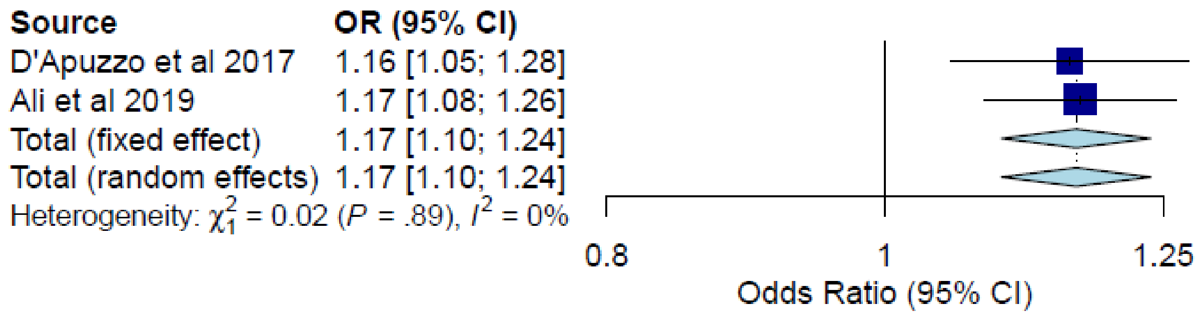

Previous myocardial infarction and coronary artery disease combined:

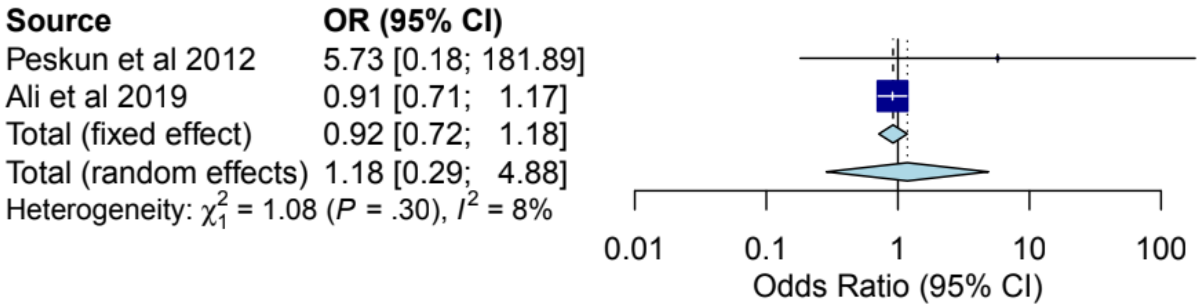

Psychiatric disorder:

Source	OR (95% CI)
Jorgensen et al 2017	1.33 [0.92; 1.92]
D'Apuzzo et al 2017	1.32 [1.17; 1.48]
Ali et al 2019	1.69 [1.37; 2.08]
Total (fixed effect)	1.40 [1.26; 1.54]
Total (random effects)	1.43 [1.21; 1.70]

Heterogeneity: $\chi^2_2 = 4.16$ ($P = .13$), $I^2 = 52\%$

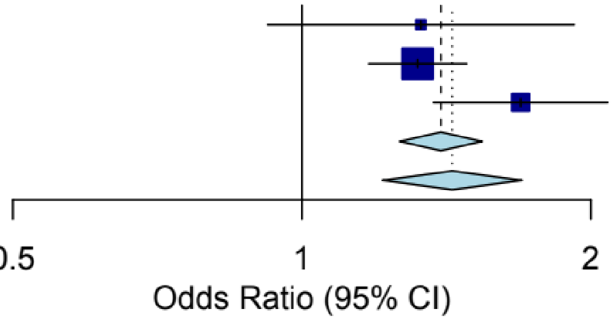

Rheumatological disease:

Source	OR (95% CI)
Urish et al 2018	1.20 [1.08; 1.34]
D'Apuzzo et al 2017	1.09 [0.92; 1.30]
Ali et al 2019	1.05 [1.01; 1.10]
Siracuse et al 2017	1.14 [1.06; 1.23]
Total (fixed effect)	1.08 [1.05; 1.12]
Total (random effects)	1.11 [1.04; 1.18]

Heterogeneity: $\chi^2_3 = 7.22$ ($P = .07$), $I^2 = 58\%$

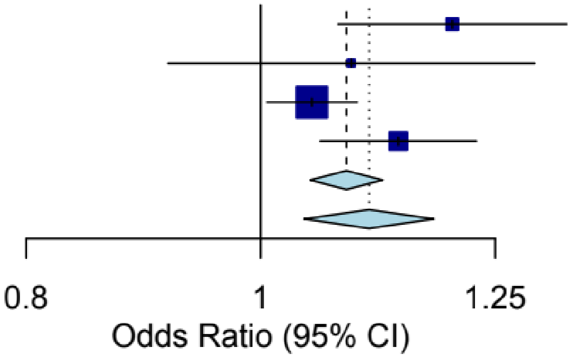

Smoking:

Source	OR (95% CI)
Jorgensen et al 2017	1.00 [0.65; 1.54]
Hart et al 2017	1.35 [0.92; 1.97]
Tang et al 2019	2.28 [0.65; 7.98]
Total (fixed effect)	1.23 [0.93; 1.62]
Total (random effects)	1.25 [0.82; 1.91]

Heterogeneity: $\chi^2_2 = 2.04$ (P = .36), I^2 = 2%

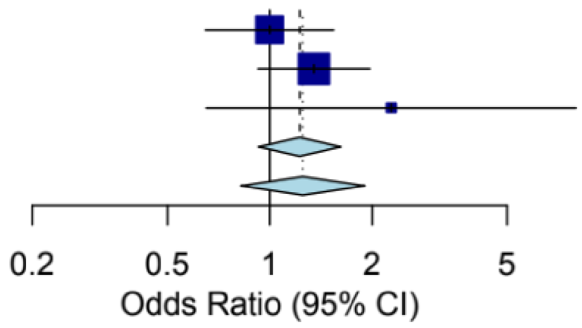

Weight loss:

Source	OR (95% CI)
D'Apuzzo et al 2017	0.76 [0.48; 1.21]
Ali et al 2019	1.04 [0.87; 1.25]
Total (fixed effect)	1.00 [0.84; 1.18]
Total (random effects)	0.95 [0.71; 1.27]

Heterogeneity: $\chi^2_1 = 1.53$ (P = .22), I^2 = 35%

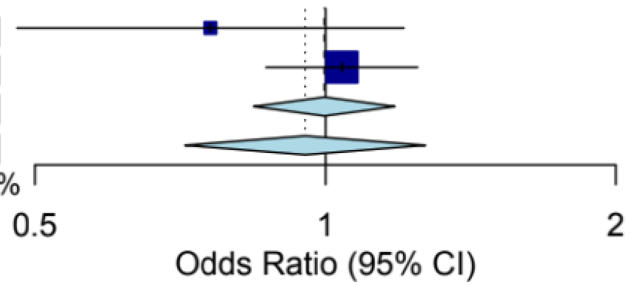

Supplement: Supplementary file 1 [file jcm-10-00134-s001.pdf]
